# Deferasirox Derivatives as Inhibitors of Kallikrein‐Related Peptidases Associated to Neurodegenerative Diseases

**DOI:** 10.1002/cmdc.202500187

**Published:** 2025-04-24

**Authors:** Rilès Boumali, Elodie David, Nancy Chaaya, Morane Lucas, Sabrina Aït Amiri, Valérie Lefort, Anthony Nina‐Diogo, Michèle Salmain, Isabelle Petropoulos, Vincent Corcé, Chahrazade El Amri, Candice Botuha

**Affiliations:** ^1^ Sorbonne Université CNRS INSERM Institut de Biologie Paris‐Seine Biological Adaptation and Ageing (B2A‐IBPS) Paris F‐75252 France; ^2^ Sorbonne Université CNRS Institut Parisien de Chimie Moléculaire (IPCM) Paris F‐75252 France

**Keywords:** 1,2,4‐triazole, deferasirox, iron chelation, kallikrein‐related peptidases, neurodegenerative diseases

## Abstract

Kallikrein‐related peptidases are a family of serine proteases whose loss of activity regulation has been particularly linked to neurodegenerative diseases. Moreover, iron overload is also a key process in some of these leading pathological conditions, particularly Alzheimer's disease. It is identified for the first time Deferasirox, a well‐known FDA‐approved iron chelator (DFX) as an initial hit for kallikrein's (KLK) inhibition and proposed here the design and synthesis of a small library of molecules using DFX as chemical scaffold. Resulting subseries of compounds are evaluated against lead central nervous system KLK's, namely, KLK1, KLK6, and KLK8 using targeted pharmacomodulations on DFX. Beyond DFX, several reversible micromolar inhibitors of these KLKs have been identified as hits and are shown to be devoid of any noticeable cytotoxicity toward neural cell lines commonly used in the field of neurodegenerative diseases. Their ability to chelate iron is also assessed in comparison to DFX and preformed iron‐compound complexes displayed slightly improved inhibition potency for some derivatives with a KLK‐dependent manner. Hence, several DFX derivatives are identified as promising starting points for the development of dual therapeutic agents in the context of neurodegenerative diseases where both deregulated KLK's proteolysis and iron dysregulation are involved.

## Graphical abstract

Dysregulated proteolysis mediated by kallikrein‐related peptidases (KLKs) and iron overload are involved in the progression of neurodegenerative diseases. Deferasirox, an clinically‐approved iron chelator, and its newly synthesized derivatives have been identified as inhibitors of major central nervous system KLKs with low cytotoxicity and effective iron chelating properties making them promising candidates as dual therapeutic agents.

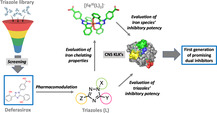

## 1 Introduction

More than one‐third of human proteases are serine proteases.^[^ ^1^, ^2^ ^]^ These proteases are involved in a wide variety of processes, such as digestion, development, blood coagulation, fibrinolysis, immune response, prohormone cleavage, signal transduction, and complement fixation.^[^ ^3^ ^]^ Among the serine proteases, tissue kallikreins or kallikrein‐related peptidases—as opposed to the plasma kallikrein from which they are distinguished—form a family of proteases present in at least six mammalian orders. In humans, tissue kallikreins (KLKs, hKLKs, or hKs for human kallikreins) are encoded by 15 structurally similar genes (KLKs) that colocalize in tandem on chromosome 19q13.4, representing the largest contiguous protease gene cluster in the human genome.^[^ ^4^ ^]^ They include human KLK1 and 14 other kallikrein‐related peptidases (KLK2‐KLK15).^[^ ^5^ ^]^

Dysregulation of KLKs has been early linked to several neurological disorders, including multiple sclerosis, Parkinson's, and Alzheimer's disease (AD).^[^ ^6^, ^7^ ^]^ KLKs have particularly emerged as promising biomarkers for AD, offering the potential for early diagnosis and disease monitoring.^[^ ^8^, ^9^, ^10^ ^]^ Among them, KLK8 (neuropsin) is expressed in the brain and has been implicated in various pathological processes associated to AD.^[^ ^11^, ^12^, ^13^, ^14^ ^]^ KLK6 (neurosin) is abundantly expressed in the peripheral nervous system and the central nervous system (CNS)^[^ ^15^, ^16^ ^]^ where it is considered as the most abundant serine protease. This enzyme has a major distribution in the brain stem and spinal cord but is also present in other major regions of the brain, like the hippocampus, frontal lobe, subthalamic nucleus, and substantia nigra. KLK6 is mostly associated to multiple sclerosis and synucleopathies.^[^ ^17^ ^]^ KLK1, primarily known for its role in regulating blood pressure and renal function, has also been reported to be implied in different CNS diseases.^[^ ^18^ ^]^ Given its roles in vascular function, neuroinflammation, and neuroprotection, KLK1 has also been suggested as a potential therapeutic target in different neuropathologic conditions such as stroke.^[^ ^19^ ^]^ The implications of KLK1, KLK6, and KLK8 in major neurological pathologies have driven substantial pharmacological efforts toward specific kallikrein inhibitors, both as potential therapeutic agents and as molecular tools for designing activity‐based probes.^[^ ^20^, ^21^, ^22^ ^]^

Despite the increasing interest in these targets, further efforts are still needed to develop inhibitors with more diverse pharmacological profiles and optimized potency and selectivity spectrum. In contrast, iron accumulation is also a hallmark of neurodegenerative diseases, such as AD, Parkinson's disease or multiple system atrophy where it is linked to oxidative stress and neuronal damage.^[^ ^23^, ^24^ ^]^ Excess iron in the brain can catalyze the production of harmful reactive oxygen species (ROS), leading to cell death and worsening disease progression.^[^ ^25^, ^26^ ^]^ For example, the impact of Fe^3+^ in amyloid beta 42 (Aβ_42_) aggregation and the translational regulation of the intracellular level of Aβ peptide precursor (APP) has been particularly studied.^[^ ^27^, ^28^, ^29^ ^]^ Iron chelators are currently being considered as potential drugs to prevent the effect of iron ions accumulation in AD and its consequences in the production of ROS and more generally oxidative stress.^[^ ^30^ ^]^ Interestingly, Deferasirox (DFX, Exjade), a FDA‐approved iron chelator used in thalassemia,^[^ ^31^ ^]^ has been shown to prevent Aβ accumulation through reduction of iron overload in AD brain.^[^ ^32^, ^33^ ^]^ Moreover, DFX and its derivatives have shown various pharmacological properties including antibacterial, antifungal, and anticancer properties^[^ ^34^, ^35^, ^36^, ^37^ ^]^ as well as enzyme inhibition.^[^ ^38^, ^39^, ^40^, ^41^, ^42^, ^43^, ^44^ ^]^

**Scheme 1:**
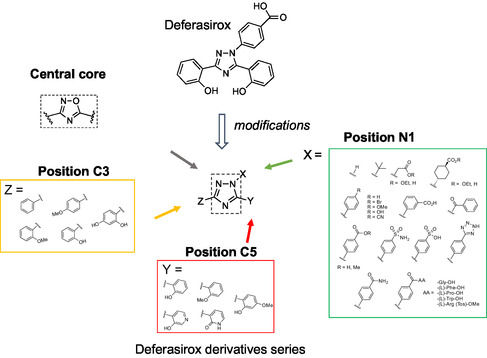
Design of DFX series analogs.

We identify DFX as a first‐hit compound acting as KLK8 micromolar inhibitor in a preliminary screening from an in‐house library including diverse chemical heterocyclic structures. We then propose DFX as a focused molecular platform to perform targeted pharmacomodulations and structure–activity relationship (SAR) studies, by varying substitutions at the N1 position of the triazole and the diphenol chelating clamp at the C3 and C5 positions (**Scheme** **1**). Inhibitory potential of DFX derivatives against lead CNS KLKs, namely, KLKs 1, 6, 8, as well as the chelating properties of the designed compounds were evaluated. Selectivity profiling, inhibition mechanisms, and docking studies were then investigated for the most potent inhibitors to better understand the structural basis of inhibition. Next, the inhibitory potency of preformed Fe^3+^ complexes for compounds retaining chelation properties within the range of DFX was assessed. Dual agents displaying both KLK's inhibitory potential and chelating properties may be indeed of great interest in neurodegenerative diseases for which both deregulated KLK's activity and iron overload constitute important hallmarks.

## 2 Results and Discussion

### 2.1 Design and Pharmacomodulations

In an initial screening of an in‐house array of 1,2,4‐triazole derivatives, DFX was shown to inhibit KLK8 in the micromolar range (IC_50_ ≈ 30 μM). To further explore the inhibition potential of DFX‐based derivatives against the relevant KLKs associated to CNS diseases, namely, KLK1, KLK6, and KLK8, we have planned to modify DFX at strategic positions using a synthetic methodology that we previously developed and mastered.^[^ ^45^, ^46^, ^47^ ^]^ While keeping the chelation ability of DFX analogs, we first explored structural diversity at the N1 position to evaluate the importance of both the phenyl ring and the carboxylic acid function on the inhibition potency.

Various substituents with different electronic properties, lengths, and shapes, including alkyl, benzoyl, phenyl, and 4‐substituted aryl groups, as well as carboxylic acid bioisosteres (tetrazole, sulfonamide, sulfonic acid), were introduced at the N1 position (see synthesis in Supporting Information). Additionally, a DFX analog with a carboxylic acid function at the N1 position was designed, replacing the phenyl group with either a carboxymethyl or carboxycyclohexyl group. Finally, different amino acids were introduced on the aromatic ring with the aim to maximize interactions with the enzymes (Scheme 1).

The benzoic acid moiety was preserved at the N1 position of the DFX series, while structural modifications were introduced to the chelating clamp at the C3 and C5 positions (see synthesis in Supporting Information). Phenols were substituted with methyl ethers to assess their impact, and additional polar substituents (OH and OMe) were incorporated to enhance electronic density and facilitate hydrogen bonding. To improve binding affinity and sustain iron chelation, heteroaromatic ring such as 3‐pyridol and 2‐pyridone were introduced at position C5 (Scheme 1).

Finally, we considered replacing the central 1,2,4‐triazole core with a 1,2,4‐oxadiazole core. This approach led to the synthesis of thirty‐six 1,2,4 triazole derivatives, twenty‐two of which are novel compounds (Scheme S1–S4, Supporting information).

### 2.2 Inhibition Properties

The synthesized 1,2,4‐triazole compounds including DFX and oxadiazole **23** were first screened at 50 and 100 μM on the selected KLKs (KLK1, KLK8, and KLK6). Compounds with inhibition above 50% were further investigated to quantify their inhibition potential. We will then give an overview of the inhibition profiles and select the hit analogs for their ability to target a given CNS KLK, e.g., KLK1, KLK6, KLK8. Then, mechanistic studies and selectivity toward a panel of representative serine proteases are presented, structure‐activity relationship on the impact of the N1 substitutions (**Table** **1**) and the disruption of the chelating clamp (**Table** **2**) on the inhibitory potency is analyzed. In a second step, chelation properties and impact of iron on inhibitory potency of best chelators are discussed. The inhibitory spectrum of the N1 substituted derivatives of DFX on the targeted CNS KLKs is summarized in Table 1.

**Table 1:** Efficiency of DFX‐based derivatives toward selected CNS KLK1, 6 and 8: effect of pharmacomodulation at the N1 site.

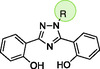				
Compoundsa)	R	KLK1b)	KLK6b)	KLK8b)
**DFX 3a**	4—COOH—C_6_H_4_—	NIc)	NI	29.2 ± 1.0
**3b**	3—COOH—C_6_H_4_—	NI	NI	21.4 ± 0.4
**3c**	H	NI	NI	NI
**3d**	*t‐*Butyl—	NI	NI	NI
**3e**	C_6_H_5_ *—*CO—	23.3 ± 0.9	NI	NI
**3fa**	*Trans*‐4—COOEt—C_6_H_10_—	NI	NI	NI
**3fb**	*Trans*‐4—COOH—C_6_H_10_—	NI	NI	NI
**3g**	4—COOEt—CH_2_—	NI	NI	NI
**3h**	4—COOH—CH_2_—	NI	NI	NI
**3i**	4—COOMe—C_6_H_4_—	NI	NI	NI
**3ja**	4*—*CO—(Gly)—(OMe)—C_6_H_4_—	NI	NI	NI
**3jb**	4*—*CO—(Gly)—(OH)—C_6_H_4_—	NI	NI	27.0 ± 0.6
**3ka**	4*—*CO—(Phe)—(OMe)—C_6_H_4_—	NI	NI	NI
**3kb**	4*—*CO—(Phe)—(OH)—C_6_H_4_—	16.4 ± 1.0	NI	24.8 ± 1.9
**3la**	4*—*CO—(Trp)—(OMe)—C_6_H_4_—	NI	NI	NI
**3lb**	4*—*CO—(Trp)—(OH)—C_6_H_4_—	NI	31.8 ± 2.9	27.1 ± 1.1
**3ma**	4*—*CO—(Pro)—(OMe)—C_6_H_4_—	NI	NI	NI
**3mb**	4*—*CO—(Pro)—(OH)—C_6_H_4_—	NI	NI	25.9 ± 0.7
**3n**	C_6_H_5_—	NI	NI	NI
**3o**	4—CN—C_6_H_4_—	NI	NI	NI
**3p**	4—Br—C_6_H_4_—	NAd)	NI	NI
**3q**	4—OMe—C_6_H_4_—	NI	NI	NI
**3r**	4—OH—C_6_H_4_—	16.4 ± 1.3	NI	NI
**3s**	4—SO_2_NH_2_—C_6_H_4_—	NI	NI	NI
**3t**	4—SO_3_H—C_6_H_4_—	NI	NI	29.5 ± 1.4
**3u**	4—CONH_2_—C_6_H_4_—	NI	NI	23.7 ± 2.3
**3v**	4—tetrazole—C_6_H_4_—	NI	NI	44.1 ± 1.7
**3w**	4*—*CO—Tos—(Arg)—(OMe)—C_6_H_4_—	NA	NI	NI
**23**	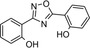	NI	NI	NI

a) For each compound, the inhibitory effect was quantified either by the percentage of inhibition at 50 μM, or by the IC_50_ for compound hits, e.g. that displayed inhibitory potency above 50% at 50 μM.

b) To determine IC_50_, compounds, at different concentrations (concentration ranges adjusted depending of the inhibitory potency) are preincubated 15 min at 37 °C in appropriate conditions. The data result from at least three independent experiments in duplicate. The IC_50_ values were calculated by fitting the experimental data to Equation (2a) or (2b) and expressed as geometric standard deviation (see Section 4). (Sub‐series 1).

c) NI: not inhibitory.

d) NA: not applicable.

**Table 2:** Efficiency of DFX‐based derivatives toward selected CNS KLK1, 6 and 8: impact of pharmacomodulations on the chelating clamp.

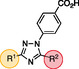					
Compoundsa)	R^1^	R^2^	KLK1b)	KLK6b)	KLK8b)
**DFX**	2—OH—C_6_H_4_—	2—OH—C_6_H_4_—	NI^c)^	NI	29.3 ± 1.0
**9**	C_6_H_5_—	2—OH—C_6_H_4_—	NI	NI	46.8 ± 0.2
**10**	4—OMe—C_6_H_4_—	2—OH—C_6_H_4_—	NI	NI	33.4 ± 1.1
**13**	2—OH—C_6_H_4_—	2—OH—4—OMe—C_6_H_3_—	NI	NI	24.8 ± 1.3
**14**	2—OH—C_6_H_4_—	2—OH—4—OH—C_6_H_3_—	NI	NI	38.3 ± 3.5
**18**	4—OMe—C_6_H_4_—	3—OH—C_5_H_3_N—	NI	NI	NI
**19**	4—OMe—C_6_H_4_—	2—OH—C_5_H_3_N—	NI	NI	NI
**21**	2—OMe—C_6_H_4_—	2—OMe—C_6_H_4_—	NI	NI	NI
**22**	2—OCOCH_3_—C_6_H_4_—	2—OCOCH_3_—C_6_H_4_—	NI	NI	NI

a) For each compound, the inhibitory effect was quantified either by the percentage of inhibition at 50 μM, or by the IC_50_ for compound hits, e.g., that displayed inhibitory potency above 50% at 50 μM.

b) To determine IC_50_, compounds, at different concentrations (concentration ranges adjusted depending of the inhibitory potency) are preincubated 15 min at 37 °C in appropriate conditions. The data result from at least three independent experiments in duplicate. The IC_50_ values were calculated by fitting the experimental data to Equation (2a) or (2b) and expressed as geometric standard deviation (see Section 4). (Subseries 2)

c) NI: not inhibitory.

The results show that six DFX derivatives bearing either acidic functional groups on the phenyl ring (carboxyl **3b**, sulfonic **3t**, tetrazole **3v**) or primary amide (**3u**) and nonhindered amino acid groups (**3jb**, **3mb)** inhibit KLK8 with almost the same potency as DFX and do not inhibit the activity of KLK1 and KLK6, demonstrating selectivity toward KLK8. In contrast, the presence of bulky amino acid groups reduces the selectivity of the compounds toward KLK8, as compound **3lb** which contains a tryptophane amino acid, inhibits both KLK8 and KLK6 within a similar IC_50_ range, while **3kb** bearing a phenylalanine residue inhibits KLK1/KLK8 in the same IC_50_ range. Two DFX derivatives (**3e**, **3r**), possessing a benzoyl group at the N1 position (**3e**) and the one possessing a phenol group (**3r**), are selective toward KLK1 with moderate activity with IC_50_ ranging from 16.4 to 23.3 μM (Table 1). Altogether, these results highlight that the presence of both phenyl and unhindered acidic groups at the N1 position is essential for maintaining activity and selectivity against KLK8.

Table 2 summarizes the inhibitory potential of DFX analogs bearing modifications at the chelating clamp on the C3 and/or C5 position of the 1,2,4‐triazole nucleus. In this SAR study, we have adopted the benzoic acid moiety as a privileged fragment at the N1 position of the DFX series.

Interestingly, modifications to the chelating clamp of DFX derivatives significantly impact their inhibitory potency. None of the screened derivatives inhibit KLK1 or KLK6, similarly to DFX itself. Retaining the phenol group at the C5 position, the substitution of the phenol at C3 with either a phenyl (**9**) or a 4‐methoxyphenyl (**10**) group preserves inhibition of KLK8. A similar trend is observed when adding a methoxy (**13**) or hydroxyl group (**14**) to the phenol at C5 while keeping the C3 phenol intact. In contrast, replacing the phenol at C5 with a 3‐pyridol (**18**) or 2‐pyridone (**19**) results in a complete loss of inhibition. Finally, analogs with protected phenols, such as methyl ethers or acetates, show no inhibition, emphasizing the critical role of the hydroxyl groups.

At this stage, these results indicate that only one phenol group is required to maintain inhibition of KLK8, and DFX derivatives with a benzoic acid moiety at the N1 position exhibit selective inhibition of KLK8 over other KLKs of the CNS. All the hit compounds (**3a, 3b, 3r, 3e, 3lb, 3mb**) that preferentially inhibit at least one of the KLK's of the CNS triad (KLK1‐KLK6‐KLK8) were found to be reversible and time‐independent inhibitors.

**Figure 1:**
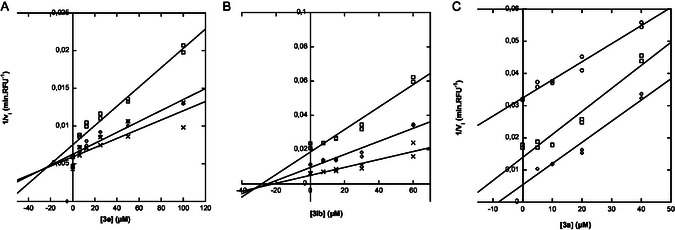
Best hit's mechanisms of inhibition. A) Dixon plots for **3e** toward **KLK1**, B) **3lb** toward **KLK6**, and C) **3a** (DFX) toward **KLK8**. Inhibitors were tested at different concentrations in presence of different fixed substrate concentrations (O: 25 μM; □: 50 μM; ◊: 100 and  ×: 200 μM) at 37 °C in optimal conditions for each enzyme (see Section 4). All data are representative of two independent experiments performed in duplicate.

To give more insights on inhibition mechanism, the types of inhibition were determined using Dixon plots. Some representative examples for each targeted KLK are given in **Figure** **1** and inhibition constants (Ki or Ki′) reported in Table 5. Interestingly, for inhibition of KLK8 with DFX (**3a**) or (**3mb**), Dixon plot gives rise to parallel lines, indicating uncompetitive inhibition with Ki′ in the low micromolar level, respectively, Ki′ = 8 μM and 25 μM. These observations indicate that these inhibitors may not strictly target the active site. In contrast, compound (**3e**) was shown to be competitive inhibitor of KLK1, which suggests its potential binding within the active site of the enzyme, whereas, the KLK6's hit (**3lb**) displays a noncompetitive profile.

### 2.3 Selectivity Profiles and Molecular Docking

The selectivity profiles of hit compounds were then determined by screening them at 50 μM against other members of representative KLK's family (KLK4, 5, 7, 11, 13, 14), as well as serine proteases implied in the blood–brain barrier physiology represented by plasmin, thrombin, tPA, and matriptase that are also involved in neuroinflammatory processes. Additional screening was also performed against the abundant and ubiquitous lysosomal cysteine protease Cathepsin L (**Table** **3**).

**Table 3:** Selectivity profiles of hit compounds 3a, 3b, 3e, 3lb, 3mb, and 3r toward other members of KLK's family and selected representative proteases.

Enzymes	3aa)	3b	3e	3lb	3mb	3r
**KLK1**	NIb)	NI	82.1	90.1	NI	79.1
**KLK6**	NI	NI	82.4	90	NI	NI
**KLK8**	70.1	62.1	NI	50.2	63.1	NI
**KLK4**	80.6	83.8	67.2	99.7	77.3	84.3
**KLK5**	85.1	98.1	98.8	98.2	95.1	85.5
**KLK7**	NI	88.1	80.1	90.5	70.1	80.4
**KLK11**	90.6	90.9	NI	81.4	76.7	NI
**KLK13**	95.1	99.2	97.1	98.1	85.2	98.3
**KLK14**	94.2	95.1	NI	98.8	89.7	62.2
**Thrombin**	NI	NI	NI	NI	61.1	NI
**Plasmin**	NI	NI	NI	60.5	NI	NI
**tPA**	NI	NI	NI	NI	NI	NI
**Matriptase**	NI	NI	NI	NI	NI	NI
**Cathepsin L**	NI	NI	NI	NI	NI	NI

a) The percentages of inhibition were determined with 50 μM of DFX‐based derivatives in duplicate, in the optimal conditions of each protease (see Section 4, biology, enzyme assays section).

b) NI: not inhibitory, percentage of inhibition below 50%.

It is noticeable that most of the hit compounds, including DFX, are devoid of any inhibitory activity toward non‐KLK serine proteases and cathepsin L. Only **3a** (DFX) hit compound of KLK8 has no inhibitory activity toward chymotrypsin‐like KLK7 while trypsin‐like KLKs are efficiently inhibited by most of the hit compounds with percentage of inhibition above 80% at 50μM. **3r** selective of KLK1 among the CNS triad, also inhibits KLK's including KLK7 but not trypsin‐like KLK11 which are poorly expressed in the CNS. In contrast, compound **3lb** inhibitor of two of targeted KLKs (KLK6 and KLK8) systematically inhibits all the investigated KLKs and plasmin, illustrating a promiscuous accommodation of serine protease's family.

Additionally, in the presence of 100 μM FeCl_3_ (Table S1, Supporting Information), very narrow modulations are observed, most of the preceding profiles of inhibition were reproduced. This selectivity analysis shows that DFX‐based hit inhibitors, while sparing the most abundant and representative serine proteases of the CNS also involved in coagulation processes, preferentially target trypsin‐like KLKs (KLK4, 5, 11, 13, 14), however, are poorly present in the CNS.

Therefore, these selectivity profiles are consistent with few potential off targets constituting thus a promising pharmacological basis for further development. Hence, **3a** (DFX) or **3r** appears as interesting candidates for further pharmacomodulations that should help to narrow selectivity profiles.

To get more insight into the structural basis of inhibition, we performed molecular docking calculations using Molegro with hit compounds to identify their putative binding site on KLK's. First, compounds **3a** (DFX), **3b**, **3lb**, and **3mb** were docked into the X‐ray structure of KLK8 (PDB: 5MS4)^[^ ^48^ ^]^ in complex with leupeptin for **3a**, **3b,** and **3mb** because of their uncompetitive mechanism of inhibition (Figure S6, Supporting Information). As expected for these compounds, the predicted putative positioning was, in most cases, consistent with the uncompetitive inhibition mechanism, e.g., accommodating pockets just outside the active site.

**Figure 2:**
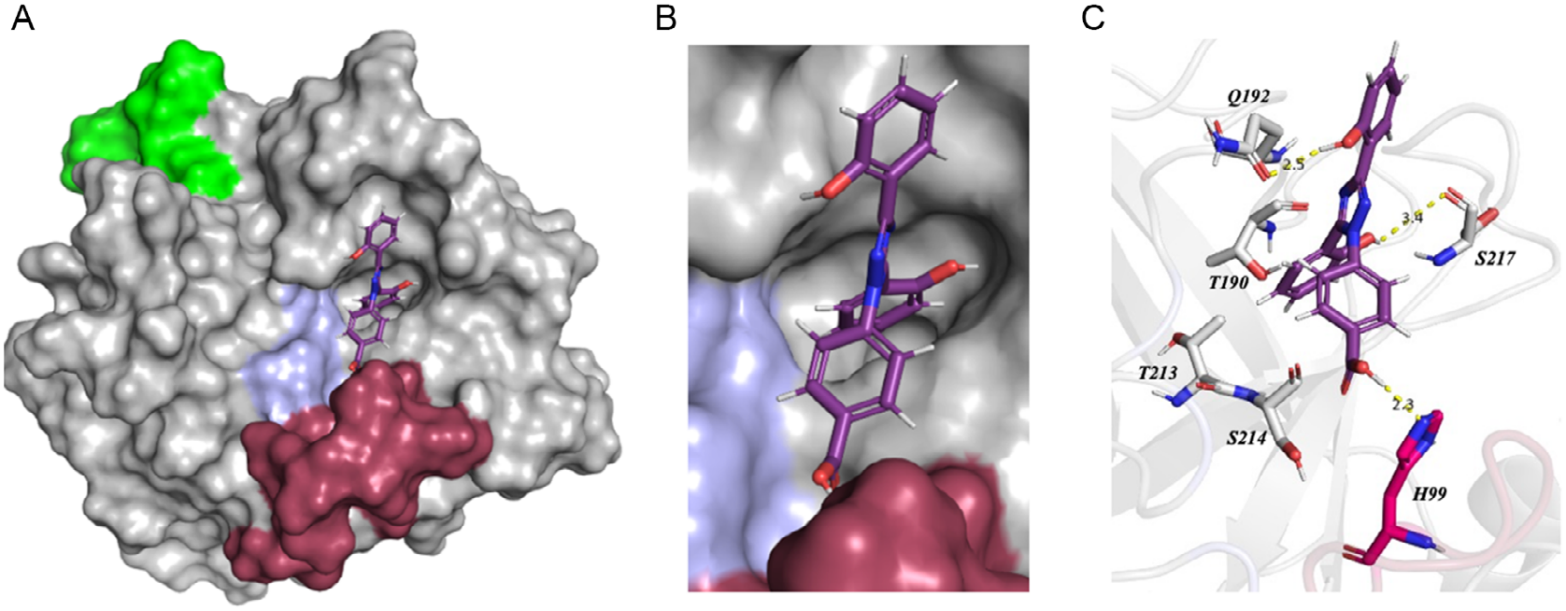
Interaction model of DFX within KLK8 (PDB: 5MS4).^[^ ^48^ ^]^ KLK8 is represented in surface mode with representation underlying the catalytic triad H57, D102, S195 (light blue), the different regulatory loops characteristic of KLK8, the calcium‐binding loop (green), the zinc‐binding loop (red). A) Representation of KLK8 with the most favorable predicted pose of DFX within the serine protease. B) First pose of DFX within a pocket at the vicinity of the KLK8 active site. C) Interaction map of DFX within the interaction pocket.

To exemplify, the best pose of DFX within KLK8 is shown in **Figure** **2**, the highest score predicted that the carboxylic group makes crucial H‐bond with His99 within the zinc‐binding 99‐loop whereas the two phenol groups are stabilized through H‐bonds with Ser217 and Gln192 residues within the S1 pocket.

This orientation within the S1 pocket is almost identical for compound **3b**, which has a carboxylic substituent at the meta position, as both phenol groups interact with the same residues, namely, Ser217 and Gln192, with an additional hydrogen bond predicted between the carboxylic acid and Asp189 specific of trypsin‐like KLKs. As for **3mb** bearing a larger amino‐acid group, its conformation differs, keeping contact with Gln192 and losing the one with Ser217 in the S1 subsite. The carboxylic group of the proline moiety forms a H‐bond with His57 of the catalytic triad.

Noncompetitive inhibitor **3lb** is predicted to interact with catalytic residues His57 and Ser195 through hydrogen bonds with its phenol groups. No interactions are observed with the tryptophan moiety, which appears to be oriented outside the catalytic pocket.

Interestingly, the specificity of compounds **3a, 3b,** and **3mb** toward KLK8 appears to depend on a hydrogen bond between their carboxylic acid group and the amine group of Ser217 within loop‐220. In KLK1 and KLK6, this position is occupied by Tyr217 and Asn217, respectively both larger residues that reduce the depth of the pocket in loop‐220, likely hindering inhibitor binding (see specific features within the KLK family in Figure S9, Supporting Information). Additionally, the topology of this pocket is relatively similar in KLK1 and KLK6 but differs significantly in KLK8.

Docking poses obtained for KLK1 and KLK6 are presented in Figure S7 and S8, Supporting Information. We have investigated the binding mode of compound **3e** bearing a benzoyl group and **3r** bearing a phenol group at the N1 position because of their selectivity toward KLK1. Both compounds are stabilized through H‐bonds with residues Gly193 and Ser195 within the S1 pocket of the active site; either through H‐bond with the two phenols of the chelating part of **3r** or through H‐bond with one phenol and the C=O of the benzoyl group of **3e**.

The specificity of compounds **3e** and **3r** toward KLK1 may be attributed to a hydrophobic interaction between their phenol groups and the side chain of Val192 in the S1 pocket of KLK1 (see interactive 2D diagram and molecular docking Figure S7, Supporting Information). In contrast, KLK6 and KLK8 have a polar residue (Asn192) at this position in their sequence, likely preventing interactions between these compounds and the S1 pocket.

### 2.4 Chelation Properties and Impact of Iron on Inhibitory Properties

DFX being a chelator of reference, we thus evaluated the iron‐chelating capacity of the newly synthesized analogs by both UV–vis spectroscopy (Figure S1, Supporting Information) and calcein transchelation assay (**Table** **4**).

**Table 4:** Calcein release assay, chelation properties, and determination of apparent affinities.

Compounda)	Apparent affinity [pM]b)
**3a (DFX)**	549 ± 7
**Modification at N1 position**	
**3b**	63 ± 11
**3c**	32 ± 12
**3d**	2150 ± 110
**3e**	410 ± 35
**3h**	220 ± 24
**3ja**	175 ± 15
**3jb**	78 ± 9
**3kb**	13.1 ± 4.2
**3la**	130 ± 20
**3lb**	74 ± 10
**3ma**	105 ± 22
**3mb**	29 ± 17
**3n**	81 ± 45
**3r**	62 ± 14
**3t**	165 ± 17
**3u**	93 ± 7
**Modification on chelation clamp**	
**9**	33 823 ± 2562
**13**	77 ± 19
**22**	27 081 ± 1871

a) Experiments were performed in duplicate and two independent experiments.

b) Apparent affinities are given in picomolar to allow comparisons (see Section 4).

First, we focused on compounds with modifications at the N1 position, while keeping the chelating clamp unchanged, along with some analogs modified in the chelating clamp for comparison. All the experiments were performed with activity buffer used in enzymatic assays.

UV–vis analysis of DFX was first performed to monitor the chelation process and the stability of the Fe^3+^ complex over time at a 1:1 ratio, typically representing the duration of inhibition assay (30 min to 4 h) (Figure S1, Supporting Information). In these conditions of pH (7.4) and ligand‐to‐iron ratio and according to Steinhauser et al. the main species in solution is assigned to [Fe(DFX)_2_]^3−^.^[^ ^49^ ^]^ Indeed, we aimed to explore not only the chelation properties but also the impact of the chelated species on enzyme inhibition.

Analogs with modified chelation clamp, some of which were devoid of inhibitory potency, were also evaluated for exemplification (compounds **9**, **13, 22**). As expected, the experimental results confirm that maintaining the integrity of the chelation clamp, as in the DFX parent compound, is essential.

UV–vis spectra recorded for DFX (**3a**) and compound **3t** containing a sulfonyl group, in the presence of FeCl_3_, both reveal the appearance of an absorption band with a maximum at 468 nm, characteristic of iron complex formation.^[^ ^50^, ^51^ ^]^ Interestingly compound **13**, a DFX analog possessing an additional methoxy group on the phenol at C5, behaves in the same way in the presence of iron (III) since a similar absorption band is observed in the UV–vis spectrum. For all these compounds, the same absorption band is observed over time (t = 0 to 240 min) indicating that the chelated species are present during the time‐course of the enzyme assays, enabling further study of their impact on the inhibitory potency. (*Vide infra*)

However, as expected, this is not the case for compounds **9** and **22** which have a disrupted chelating clamp. In the presence of FeCl_3_, no changes in the absorption spectra of **9** and **22** are observed.

The results from the calcein release assays (see Section 4 for details, Figure S3, Supporting Information and Table 4) on a selection of modified compounds reveal that N1‐substituted derivatives generally exhibit comparable or even enhanced chelation properties compared to parent DFX, except for compound **3d** substituted by a *t*‐Butyl group. Since the assay was not fully quantitative, we provided an estimation of pseudo‐affinity constants, which allowed ranking of chelation potential. The apparent affinity constants are given in picomolar to facilitate the comparison with DFX.

The best affinities (below 30 pM) were obtained for amino‐acid substituted DFX (Proline‐**3mb** and phenylalanine‐**3kb**) which is not surprising since a DFX peptide conjugate (DFX‐TAT) has been reported to efficiently remove labile iron from buffered solution into RBE4 cell line.^[^ ^52^ ^]^ The non‐N1 substituted DFX derivatives **3c** also demonstrated a strong affinity for iron, a finding that has already observed in studies of this compound as a potential anticancer complex.^[^ ^53^ ^]^ The iron‐chelating capacity of other N1‐substituted derivatives can be compared to that of similar DFX derivatives, whose chelating ability has been shown by fluorescence quenching experiments with iron(III) salts.^[^ ^51^, ^54^ ^]^

As for compounds **9** and **22** which feature a disrupted chelation clamp, respectively, possessing only one phenol group and protected phenols, both show very low affinity for Fe^3+^ ions as evidenced by their similar apparent affinity constants of up to 30 000 pM. Interestingly, compound **13**, a DFX analog possessing an additional methoxy group on the phenol at C5 shows a stronger affinity for iron almost eight times more effective than DFX. Taking advantage of the chelation properties of our DFX‐based analogs, we investigated whether resulting chelated species could influence the inhibitory potency (**Table** **5**).

**Table 5:** Mechanisms of inhibition and inhibition constants (Ki and Ki′) values of hit compounds on targeted KLKs.

KLKa)	Compound	Type of inhibition	Ki[μM]	Ki′ [μM]
**KLK1**	**3e**	Competitive	24	–
**KLK1**	**3e** + Fe^3+^	Competitive	78	–
**KLK1**	**3r**	Noncompetitive	29	–
**KLK1**	**3r** + Fe^3+^	Noncompetitive	7	–
**KLK6**	**3lb**	Noncompetitive	33	–
**KLK6**	**3lb** + Fe^3+^	Noncompetitive	9	–
**KLK8**	**3a**	Uncompetitive	–	8
**KLK8**	**3a** + Fe^3+^	Uncompetitive	–	7
**KLK8**	**3lb**	Competitive	12	–
**KLK8**	**3lb** + Fe^3+^	Competitive	10	–
**KLK8**	**3mb**	Uncompetitive	–	25
**KLK8**	**3mb** + Fe^3+^	Uncompetitive	–	21
**KLK8**	**3b**	Uncompetitive	–	18
**KLK8**	**3b** + Fe^3+^	Uncompetitive	–	18

a) Ki and Ki′ were obtained using conventional Dixon Plots. Ki′ were determined using 100 μM substrate concentration.

First, the impact of Fe^3+^ ions was first evaluated on the selected KLKs (Figure S4, Supporting Information). It was demonstrated that there is no negative effect on KLK's activity, even at high Fe^3+^ concentration.

Next, we evaluated the impact of preincubated mixtures of each compound at 50 μM and Fe^3+^ at 100 μM to model an iron overload condition on targeted KLKs (Figure S5, Supporting Information).

Distinct effects were observed. First, the effect of iron on KLK inhibition can be evaluated by comparing the mean inhibition measured in the presence and absence of iron, revealing differential effects. The mean inhibition for KLK6 is 18 ± 25%, which increases to 67 ± 30% in the presence of iron. For KLK8, the mean inhibition is 31 ± 24%, increasing to 62 ± 19% with iron. Finally, for KLK1, the inhibition increases from 31 ± 24% without iron to 53 ± 22% with iron. These results suggest that iron enhances the inhibitory effect on the targeted KLKs, with the most pronounced effect for KLK6; which is otherwise poorly inhibited.

We suppose that chelation, with or without the regulatory effect of iron on KLK's conformational state, may enhance the binding of DFX‐based analogs, especially with KLK6. We have also observed an effect dependent on the structure of the analogs. Overall, as anticipated, the analogs which possess a low affinity for iron, do not show an increased inhibitory activity in the presence of iron, i.e., **3d** and **22**. In contrast, the inhibitory potency of DFX analogs with a good affinity for iron, similar to or greater than DFX itself, is not systematically enhanced on each KLK. In addition, compound **3c** bearing no substituent at the N1 position shows a significant higher inhibitory potency in the presence of Fe^3+^ on all KLKs (Table S5, Supporting Information).

Finally, the mechanisms of inhibition for hit compounds were also determined to illustrate the effect of iron (Table 5). In most cases, the type of inhibition remains unchanged, and the determined inhibition constants are either unchanged or slightly lowered in the presence of iron, except for hit compound **3r** bearing a phenol group which exhibits a significantly lower inhibition constant on KLK1 in the presence of iron. This could be correlated to the higher affinity of **3r** for iron compared to **3e**, the other hit on KLK1.

### 2.5 Cytotoxicity Evaluation

Potential cytotoxicity of hit compounds **(3a, 3b, 3e, 3r, 3lb, and 3mb)** was assessed on two different human cell lines, a neuroblastoma cell line (SH‐SY5Y)^[^ ^55^ ^]^ and a microglial cell line (HMC3).^[^ ^56^ ^]^ These two cell lines allow to provide a first evaluation of the inhibitor's effect toward these two major cell lines that are commonly using in the context of neurodegenerative diseases for the evaluation of therapeutical compounds. SH‐SY5Y cells were used to model neuron's cell populations while HMC3 the immune cells responsible for neuro‐inflammation which an important hallmark of these diseases. The targeted proteases are secreted enzymes that act on cellular pathways by activating extracellular or membrane‐bound substrates. Additionally, the conventional pharmacological action of Deferasirox is known to occur extracellularly. Therefore, the potential effects of our compounds are expected to be extracellular and do not require cell penetration. Figure S8, Supporting Information, presents the results obtained after 24h of treatment with concentrations corresponding at least to twofold value of mean inhibition constants (25 and 50 μM), inhibitors in absence or presence of iron (prechelated species) were devoid of any noticeable cytotoxic effect on both cell lines (percentage of cell viability above 80%). This lack of cytotoxicity constitutes a favorable pharmacological feature for further development. Ongoing research in our lab aims at deciphering how iron and inflammatory signaling may be impacted by these compounds.

## 3 Conclusion

To sum up, we designed and synthesized a small library of Deferasirox‐based analogs targeting key CNS kallikrein‐related peptidases, resulting to a first generation of DFX‐based inhibitors with IC_50_ in micromolar range. Among these analogs, we found at least one hit compound that is able to preferentially target one of the CNS KLK's (KLK1‐KLK6‐KLK8) as reversible and time‐independent inhibitors. We show with SAR studies corroborated by molecular docking analysis that Deferasirox analogs exhibit marked selectivity depending on the functional groups present. Analogs bearing acidic groups or nonbulky amino acids at the N1 position of the triazole preferentially inhibit KLK8, while analogs modified at the N1 position, by a phenol or benzoyl group confer selectivity for KLK1. In addition, modifications to the chelating clamp also impact inhibitory potency, highlighting the importance of an unprotected phenol group to maintain activity and selectivity against KLK8.

Notably, we report for the first time the inhibitory potential of FDA‐approved Deferasirox and analogs against proteases, thereby expanding the spectrum of properties of this versatile FDA‐approved iron chelator. We particularly underscored that DFX is a preferential inhibitor of KLK8, a relevant serine protease in AD, dementias, and other psychiatric disorders, although its selectivity toward trypsin‐like KLKs remains to be optimized. Moreover, the uncompetitive nature of some identified inhibitors could be an advantageous feature in vivo, enhancing selectivity toward specific biological substrates involved in pathologies. Interestingly, in the presence of iron, DFX derivatives retain their inhibitory properties. Compounds with enhanced chelation abilities, compared to DFX, also exhibited slightly improved inhibition activity. However, the exact mechanism, such as the binding modes of the chelated entities, still remains to be determined. One can hypothesize, for example, that the resulting chelated entities may bind to regulatory pockets, such as the calcium pocket of KLK8, favoring inactive conformations. Hit compounds were also shown to be devoid of any cytotoxic effects toward neural cell lines used as models in the context of neurodegenerative diseases (SH‐SY5Y, HMC3). The dual ability of some analogs may serve as a basis to design a second‐generation of dual agents with improved selectivity spectrum toward CNS KLKs, where iron dyshomeostasis and proteolytic imbalance are involved.

## 4 Experimental Section

4.1

4.1.1

##### Enzyme Assays

All enzyme stocks were stored at −20 °C and purchased from R&D Systems as proenzymes (KLK6, KLK8, KLK13) or as mature and active forms (KLK5). Activation of KLK6, KLK8, and KLK13 is performed in optimized buffers. The activation reaction is initiated by Lysyl‐Endopeptidase at 2.5 mU mL^−1^ (Wako Bioproducts). The activation reaction is stopped by diluting the enzyme to 0.5 μg mL^−1^ in assay buffer (50 mM Tris, 1 m Citrate, 0.05% w/v Brij‐35, pH 8) to obtain an active enzyme stock. KLK1 was purchased from R & D Systems as pro‐KLK1. Activation of KLK1 is performed in 50 mM Tris‐HCl, 10 mM CaCl_2_, 150 mM NaCl, 0.05% Brij‐35, pH 7.5. The activation reaction is initiated by bacterial thermolysin at 0.4 μg mL^−1^ (Sigma‐Aldrich) and stopped by addition of 100 mM EDTA (Sigma‐Aldrich) to obtain an active KLK1 stock at the same concentration of 100 μg mL^−1^. The activation reactions for KLK7 are initiated bacterial thermolysin at 0.4 μg mL^−1^ (Sigma‐Aldrich) and stopped by addition of 100 mM EDTA (Sigma‐Aldrich) to obtain active stocks. All enzyme stocks were stored at −20 °C. H‐PFR‐AMC (KLK1), Boc‐VPR‐AMC (KLK5, KLK8, KLK13), Boc‐QAR‐AMC (KLK6, Plasmin), substrates were purchased from Bachem. MeO‐Suc‐RPY‐AMC (KLK7) substrate was purchased from AAT Bioquest. Plasmin (Sigma‐Aldrich) was purchased in mature and active form.

##### Kinetic Assays

Compounds were screened on KLKs using a BMG Fluostar microplate reader (black 96‐well microplates). Hit compounds were screened on the selected group of enzymes using optimized concentrations and buffers (KLK1 0.8 nM, KLK4 10 nM, KLK5 0.2 nM; KLK6 1.9 nM; KLK7 6 nM; KLK8 0.2 nM; KLK11 5.2 nM, KLK13 0.47 nM, KLK14 2.5 nM, plasmin 3.3 nM, thrombin 0.1 nM, tissue‐plasminogen activator (tPA) 0.1 nM, matriptase 1 nM, cathepsin L 2 nM) either in absence or prior preincubation in the presence of 100 μM of Fe^3+^.

The proteases are first preincubated for 15 min with each compound (50 and 100 μM) or with DMSO (<2%, negative control) in a total volume of 85 μL of 50 mM Tris buffer, 1 m citrate, 0.05% Brij‐35, pH 7, 37 °C. The reaction is triggered by the appropriate substrate at the optimized concentration in 15 μL of 50 mM Tris buffer, 1 m citrate, 0.05% Brij‐35, pH 8 (H‐PFR‐AMC 100 μM; Boc‐VPR‐AMC 100 μM; Boc‐QAR‐AMC 100 μM; Meo‐Suc‐RPY‐AMC 150 μM, Z‐LR‐AMC 100 μM, Z‐GGR‐AMC 100 μM) and followed for 30 min at 37 °C. Results are given from two independent experiments with a standard error below 10%. The release of the AMC fluorescent group is detected using the following wavelengths: λ_ex_ = 360 nm and λ_em_ = 460 nm. The percent inhibition is calculated from Equation (1) where *V*_0_ is the initial rate of the DMSO control, the initial rate in the presence of the inhibitor *V*_i_.

(1) $$\%\text{inhibition}=\left(1-\left(v_{i}/v_{0}\right)\right)\times 100$$

The selected compounds are those for which the inhibition is greater than 50% at a concentration of 50 μM on KLK1, KLK6, and KLK8 then the IC_50_ is determined. The inhibitory effect of the compound (%) as a function of its concentration generally follows Equation (2) which results in a hyperbole or a sigmoid. The equation is entered in the Kaleidagraph 4.5 software for curve fitting f([I]) = % Inhibition, where [I] is the inhibitor concentration. The concentration ranges of inhibitor were adjusted to inhibitory potency as detected in preliminary screening tests; [I] was from 0.1 to 100 μM.

The inhibitory activity of compounds was expressed as IC_50_ (inhibitor concentrations giving 50% inhibition). The values of IC_50_ were calculated by fitting the experimental data to Equation (2a,b)

(2a) $$\%\text{Inhibition}=100\times \left(1-V_{i}/V_{0}\right)=100{\left[I\right]}_{0}/\left(IC_{50}+\;{\left[I\right]}_{0}\right)$$

(2b)

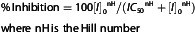

Reversibility was analyzed by diluting the reaction mixtures (dilution factor of 100) after 15 min preincubation of the enzyme with inhibitor. Aliquots of reaction mixtures (1 μL) were added to 99 μL of buffer containing the fluorogenic substrate (experimental conditions identical to the routine protocol used for a given enzyme). The mechanism of inhibition was determined by varying substrate and inhibitor concentrations, the type of inhibition and inhibition parameter (Ki or Ki′) are determined by Dixon's plots.

##### Chelation Assay

The study of the chelating propensity of 1,2,4 triazole derivatives was performed by spectrofluorimetry using the calcein transchelation assay.^[^ ^57^ ^]^ Calcein was purchased from Merck (Sigma‐Aldrich). Calcein is a fluorescent molecule (λ_ex_ = 495 nm, λ_em_ = 515 nm) that chelates Fe^3+^ ions. Following chelation, fluorescence emission of calcein is quenched. The assay consists in measuring the ability of inhibitors to transchelate Fe^3+^ from the calcein/Fe^3+^ complex, resulting in the release of calcein and restoration of fluorescence. Assays were performed both in 20 mM HEPES, 150 mM NaCl (pH = 7.3), and TCB buffers using 100 nM calcein in the presence of 1 mM FeCl_3_ for quenching of the calcein fluorescence. The solution is left for 24 h at 4 °C and then mixed with an inhibitor solution (250 μL of calcein solution for 20 μL of inhibitor solutions at different concentrations) for 4 h at room temperature. Fluorescence of solutions is finally measured at 37 °C on BMG Fluostar microplate reader. Resulting Fluorescence = f([I]) curves were analyzed using Kaleidagraph to obtain pseudo‐affinity constants (*K*_d_ in pM) using Equation (3).

(3) $$\text{Fluorescence}\left({\left[I\right]}_{0}\right)=100\times {\left[I\right]}_{0}/\left(Kd\;+\;{\left[I\right]}_{0}\right)\;\text{where }{\left[I\right]}_{0}\text{is the concentration of inhibitor}.$$

##### Molecular Docking

Molecular docking calculations were conducted to propose interaction models of hit 1,2,4‐triazole derivatives with KLK1, KLK6, and KLK8. The protonation states of the ligand were calculated using ChemSketch at pH = 7. The 3D conformations of the ligand were generated by ChemSketch and these KLKs were checked by Molegro. The coordinates of the KLK's target were retrieved from the Protein Data Bank: KLK1 (PDB 1SPJ); KLK6 (PDB 1LO6); KLK8 (PDB 5MS4). The ligand and target were prepared using Molegro before flexible molecular docking with Molegro. For KLK1, KLK6, and KLK8: the box was centered near the geometrical center of the enzyme (near the catalytic triad residues: H57, D102, and S195), with final sizes of 43.58 × 36.45 × 18.36 Å^3^ for KLK1, 30.92 × 80.89 × 72.77 Å^3^ for KLK6 and 32.79 × 10.10 × 10.72 Å^3^ for KLK8 which largely included the whole enzyme each time. 15 poses were asked ranked by their MolDock Scoring functions. Resulting poses were visualized and figures generated using PyMol. The coordinate files of selected poses are available in the online version. Interactive 2D diagram has been generated from PoseEdit from the free available ProteinsPLus webserver (https://proteins.plus).^42^

## Conflict of Interest

The authors declare no conflict of interest.

## Author Contributions

**Rilès Boumali**: formal analysis (equal); methodology (equal); validation (equal); writing—review & editing (supporting). **Elodie David**: formal analysis (equal); methodology (equal); validation (equal); writing—review & editing (supporting). **Nancy Chaaya**: formal analysis (supporting); methodology (supporting); validation (supporting). **Morane Lucas**: formal analysis (supporting); methodology (supporting); validation (supporting). **Sabrina Aït Amiri**: formal analysis (supporting); methodology (supporting); validation (supporting). **Valérie Lefort**: validation (supporting). **Anthony Nina‐Diogo**: formal analysis (supporting); methodology (supporting); validation (supporting). **Michèle Salmain**: writing—review & editing (supporting). **Isabelle Petropoulos**: supervision (supporting); writing—review & editing (supporting). **Vincent Corcé**: conceptualization (equal); supervision (supporting); writing—review & editing (supporting). **Chahrazade El Amri**: conceptualization (equal); funding acquisition (equal); investigation (equal); project administration (equal); supervision (equal); writing—original draft (equal); writing—review & editing (equal). **Candice Botuha**: conceptualization (equal); funding acquisition (equal); investigation (equal); project administration (equal); supervision (equal); writing—original draft (equal); writing—review & editing (equal). **Rilès Boumali** and **Elodie David** contributed equally to this work.

## Supporting information

Supplementary Material

## Data Availability

The data that support the findings of this study are available from the corresponding author upon reasonable request.
