# Supplementary material for: Deferasirox Derivatives as Inhibitors of Kallikrein‐Related Peptidases Associated to Neurodegenerative Diseases
Source: ChemMedChem. 2025 Apr 24;20(13):e202500187. doi: 10.1002/cmdc.202500187 (PMC12221113; doi:10.1002/cmdc.202500187)
Supplement: Supplementary file 1 — Supplementary Material [file CMDC-20-e202500187-s001.pdf]

Supplementary Information for

DEFERASIROX DERIVATIVES AS INHIBITORS OF KALLIKREIN-RELATED PEPTIDASES ASSOCIATED TO NEURODEGENERATIVE DISEASES.

Rilès Boumali[a]†, Elodie David[a],[b]†, Nancy Chaaya[a], Morane Lucas[b], Sabrina Aït Amiri[a], Valérie Lefort[a], Anthony Nina-Diogo[b], Michèle Salmain[b], Isabelle Petropoulos[a], Vincent Corcé[b], Chahrazade El Amri[a]*, Candice Botuha[b]*

[a] R. Boumali, Dr. E. David, Dr. N. Chaaya, Dr. S. Aït Amiri, V. Lefort, Pr. I. Petropoulos and Pr. C. El Amri.
Sorbonne Université, CNRS, INSERM, Institut de Biologie Paris-Seine, Biological Adaptation and Ageing (B2A-IBPS)
F-75252 Paris, France
E-mail: chahrazade.el_amri@sorbonne-universite.fr

[b] Dr. E. David, M. Lucas, Dr. A. Nina-Diogo, Dr. M. Salmain, Dr. V. Corcé and Dr. C. Botuha
Sorbonne Université, CNRS, Institut Parisien de Chimie Moléculaire (IPCM),
F-75252 Paris, France
E-mail: candice.botuha@sorbonne-universite.fr

† Rilès Boumali and Elodie David contributed equally to this work.

Table of Contents

1. Synthetic routes for deferasirox derivatives 5

Scheme S1. Synthetic route for N1-substituted DFX derivatives........ 6

Scheme S2. Synthetic route for C3-substituted DFX derivatives a 7

Scheme S3. Synthetic Route for C5-substituted DFX derivatives a 7

Scheme S4. Synthetic Route for C3 and C5-substituted DFX derivatives 8

Scheme S5. Synthesis of oxadiazole 23 8

2. Material and methods 8

2.a. Experimental procedures 8

2.b. Mechanisms of inhibition 31

Figure S1: Mechanisms of inhibition of hit compounds in absence and presence of 100 µM $FeCl_3$. Dixon plots for KLK1, KLK6 and KLK8 hits 31

2.c. Chelation properties, iron interactions, and selectivity profiles of selected compounds targeting KLK activity. 34

Figure S2: Stability of the chelated species as monitored by spectrophotometry........ 34

Figure S3 : Trans-chelation test to analyze chelation properties for the newly synthetized compounds in comparison to DFX........ 35

Figure S4 : Effect of [Fe^{3+}] on KLK's activity : A. KLK1; B. KLK6; C. KLK8. Curve fits for Pseudo-IC_{50} are shown as insert. 36

Figure S5: Effect of [Fe^{3+}] on the inhibition potential of DFX-based derivatives. A. KLK1; B. KLK6; C. KLK8. 37

Table S1 : Selectivity profiles of hit compounds towards other members of KLK's family and selected representative proteases in the presence of 100 µM 38

2.d. Molecular docking 39

Figure S6 : Molecular docking-interaction model of compound 3b, 3lb and 3mb with KLK8 (PDB: 5MS4). 39

Figure S7 : Molecular docking-interaction model of compound 3e and 3r with KLK1 (PDB: 1SPJ). 42

Figure S8 : Molecular docking-interaction model of compound 3lb with KLK6 (PDB: 1LO6). 44

Figure S9: Specific features within the KLK Family – Focus on the Topology of Regulatory 220-Loops in Human KLKs 1, 6, and 8 within active sites 45

2.e. Cytotoxicity evaluation: experimental procedure 46

Figure S10 : Cell viability assay after exposure of HMC3 47

3. ^{1}H and ^{13}C NMR spectra of new compounds 48

Figure S11 : 1H and 13C NMR spectra of compound 3d 49

Figure S12 : ^{1}H and ^{13}C NMR spectra of compound of (±)-Ethyl 4-[(tert-butyloxycarbonyl)hydrazono]cyclohexanecarboxylate 50

Figure S13 : ^{1}H and ^{13}C NMR spectra of (±)-Trans-ethyl 4-[2-(tert-: butyloxycarbonyl)hydrazino]cyclohexanecarboxylate 51

Figure S14 : ^{1}H and ^{13}C NMR spectra of (±)-Trans-ethyl-4-hydrazinylcyclohexanecarboxylate hydrochloride 52

Figure S15 : ^{1}H and ^{13}C NMR spectra of 3fa 53

Figure S16 : ^{1}H and ^{13}C NMR spectra of 3fb 54

Figure S17 : ^{1}H and ^{13}C NMR spectra of 3g 55

Figure S18 : ^{1}H and ^{13}C NMR spectra of 3h 56

Figure S19 : ^{1}H and ^{13}C NMR spectra of 3ja 57

Figure S20 : ^{1}H and ^{13}C NMR spectra of 3jb 58

Figure S21 : ^{1}H and ^{13}C NMR spectra of 3kb 59

Figure S22 : ^{1}H and ^{13}C NMR spectra of 3la 60

Figure S23 : ^{1}H and ^{13}C NMR spectra of 3lb 61

Figure S24 : ^{1}H and ^{13}C NMR spectra of 3ma 62

Figure S25 : ^{1}H and ^{13}C NMR spectra of 3mb 63

Figure S26 : ^{1}H and ^{13}C NMR spectra of 3p 64

Figure S27 : ^{1}H and ^{13}C NMR spectra of 3r 65
Figure S28 : ^{1}H and ^{13}C NMR spectra of 3v 66
Figure S29 : ^{1}H and ^{13}C NMR spectra of (S)-methyl 2-amino-5-(3-tosylguanidino)pentanoate 67
Figure S30 : ^{1}H and ^{13}C NMR spectra of 3w 68
Figure S31 : ^{1}H and ^{13}C NMR spectra of 6 69
Figure S32 : ^{1}H and ^{13}C NMR spectra of 8 70
Figure S33 : ^{1}H and ^{13}C NMR spectra of 9 71
Figure S34 : ^{1}H and ^{13}C NMR spectra of 10 72
Figure S35 : ^{1}H and ^{13}C NMR spectra of 13 73
Figure S36 : ^{1}H and ^{13}C NMR spectra of 14 74
Figure S37 : ^{1}H and ^{13}C NMR spectra of 20 75
4. HPLC chromatograms of all the tested compounds 3a, 3b, 3c, 3d, 3e, 3fa, 3fb, 3g, 3h, 3i, 3ja, 3jb, 3ka, 3kb, 3la, 3lb, 3ma, 3mb, 3n, 3o, 3q, 3r, 3s, 3t, 3u, 3v, 3w, 9, 10, 13, 14, 18, 19, 21, 22, 23. 76
Figure S38 : HPLC chromatogram of 3a 77
Figure S39 : HPLC chromatogram of 3b 78
Figure S40 : HPLC chromatogram of 3c 79
Figure S41 : HPLC chromatogram of 3d 80
Figure S42 : HPLC chromatogram of 3e 81
Figure S43 : HPLC chromatogram of 3fa 82
Figure S44 : HPLC chromatogram of 3fb 83
Figure S45 : HPLC chromatogram of 3g 84
Figure S46 : HPLC chromatogram of 3h 85
Figure S47 : HPLC chromatogram of 3i 86
Figure S48 : HPLC chromatogram of 3ja 87
Figure S49 : HPLC chromatogram of 3jb 88
Figure S50 : HPLC chromatogram of 3ka 89
Figure S51 : HPLC chromatogram of 3kb 90
Figure S52 : HPLC chromatogram of 3la 91
Figure S53 : HPLC chromatogram of 3lb 92
Figure S54 : HPLC chromatogram of 3ma 93
Figure S55 : HPLC chromatogram of 3mb 94
Figure S56 : HPLC chromatogram of 3n 95
Figure S57 : HPLC chromatogram of 3o 96

Figure S58 : HPLC chromatogram of 3p 97
Figure S59 : HPLC chromatogram of 3q 98
Figure S60 : HPLC chromatogram of 3r 99
Figure S61 : HPLC chromatogram of 3s 100
Figure S62 : HPLC chromatogram of 3t 101
Figure S63 : HPLC chromatogram of 3u 102
Figure S64 : HPLC chromatogram of 3v 103
Figure S65 : HPLC chromatogram of 3w 104
Figure S66 : HPLC chromatogram of 9 105
Figure S67 : HPLC chromatogram of 10 106
Figure S68 : HPLC chromatogram of 13 107
Figure S69 : HPLC chromatogram of 14 108
Figure S70 : HPLC chromatogram of 18 109
Figure S71 : HPLC chromatogram of 19 110
Figure S72 : HPLC chromatogram of 21 111
Figure S73 : HPLC chromatogram of 22 112
Figure S74 : HPLC chromatogram of 23 113

1. Synthetic routes for deferasirox derivatives

Scheme 1 describes the synthesis of deferasirox derivatives **3** modified at the N1 position of the 1,2,4-triazole ring. Using a previously described procedure [1], we synthesized thirteen N1-modified DFX derivatives among them five are new from 2-(2-hydroxyphenyl)-benzo-4H-[1,3]-oxazin-4-one **2**, which was prepared by condensing salicylamide **1** with salicylic acid using thionyl chloride. The reaction of **2** between the appropriate hydrazine derivatives and triethylamine led to the corresponding 1,2,4-triazoles **3a-e, 3fa, 3g and 3n-t**. Next, compounds **3fb** (R^1 = $C_6H_{10}CO_2H$) and **3h** (R^1 = CH_2CO_2H) were prepared respectively from **3fa** (R^1 = $C_6H_{10}CO_2Et$) and **3g** (R^1 = CH_2CO_2Et) by a saponification-acidification sequence and **3r** (R^4 = OH) prepared from **3q** (R^4 = OMe) using neat pyridinium chloride. Compound **3i** (R^3 = OMe) was obtained from DFX **3a** by esterification reaction conditions. Then, derivatives possessing amino esters groups **3ja-3ma** were obtained from DFX **3a** using N-acylation conditions with thionyl chloride as carboxylic activating reagent. Saponification and subsequent acidification reactions yielded to the corresponding amino acid compounds **3jb-3mb**. Notably triazole **3w** incorporating a tosylated (L)-arginine methyl ester was synthesized following the same strategy. Its deprotection being unsuccessful, this compound was used in its protected form in the enzymatic tests. Finally, hydrolysis under basic conditions of 4-benzonitrile substituted triazole **3o** (R^4 = CN) led to triazole **3u** (R^4 = $CONH_2$) whereas triazole **3v** carrying a 4-phenyl tetrazole fragment was obtained via a [2+3] cycloaddition of nitrile **3o** and sodium azide.

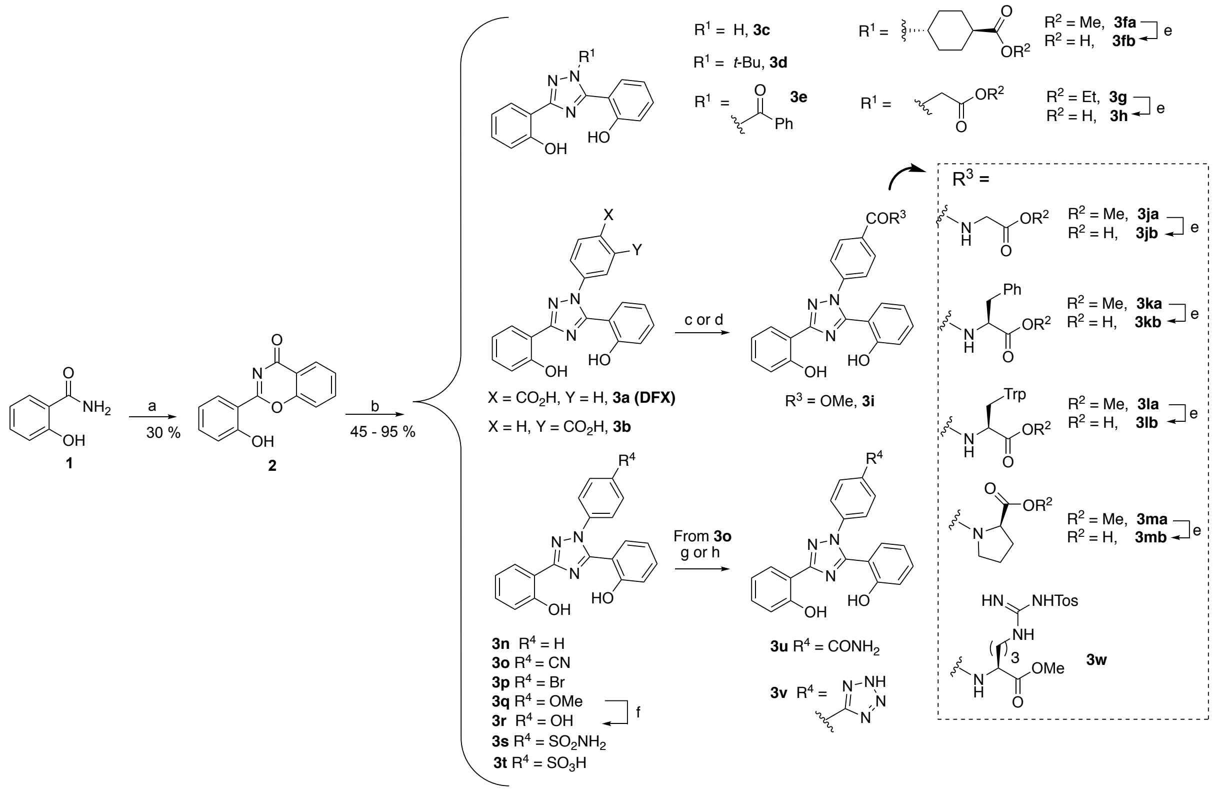

Scheme S1. Synthetic route for N1-substituted DFX derivatives

[a]Reagents and conditions : (a) (i) salicylic acid, $SOCl_2$, xylene, 10-15 °C, (ii) 35-40 °C, 1 h (iii) salicylamide **1** (iv) 120 °C, 4 h (b) 4-CO_2H-Ph-$NHNH_2$ or 3-CO_2H-Ph-$NHNH_2$ or R^1-NH-NH_2 or 4-R^4-Ph-NH-NH_2, NEt_3, EtOH, 110 °C, 4 h (c) (i) MeOH, H_3CCOCl, 0 °C, (ii) 20 °C, 12 h, (d) (i) $SOCl_2$, DCM, reflux, 5 h (ii) HCl.Ala-OMe or HCl.(L)-Phe-OMe or HCl.(L)-Trp-OMe or HCl.(L)-Pro-OMe or HCl-(L)-Tos-Arg-OMe, NEt_3, DCM, 20 °C (e) (i) KOH, MeOH, 20 °C, 3h, (ii) 1M HCl (f) (i) Pyridine.HCl, 225 °C, 2 h, (g) KOH, tBuOH, reflux, 2 h, (h) (i) NaN_3, NH_4Cl, DMF, 120 °C, 48 h.

DFX derivatives **9** and **10** modified at the C3 position of the triazole ring were obtained in three steps from 2-fluorobenzamide **4** using an adapted described procedure.[2] Sodium salts **5** (R^5 = H) and **6** (R^5 = OMe) were first isolated by condensing 2-fluorobenzamide **4** with the corresponding aroyl chloride in the presence of sodium hydride. Intramolecular O-arylation under microwave irradiation of sodium salts **5** and **6** respectively yielded benzoxazinones **7** and **8** which upon addition of 4-hydrazinobenzoic acid produced triazoles **9** and **10**. (Scheme 3)

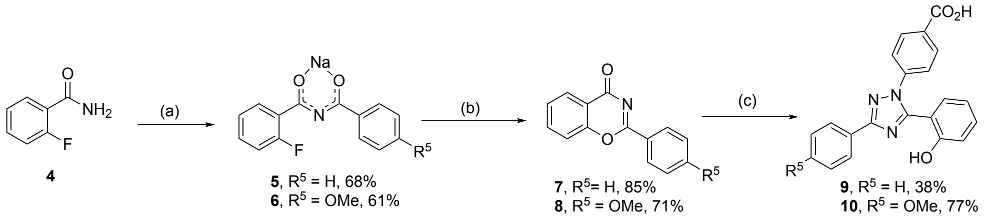

Scheme S2. Synthetic route for C3-substituted DFX derivatives [a]

[a] Reagents and conditions: (a) (i) NaH, THF, 0 °C, 1 h, (ii) PhCOCl or 4-OMe-PhCOCl, THF, -78 °C to 20 °C, 12 h, (b) DMSO, MW, 180 °C, 30 min. (c) 4-CO_2H-PhNHNH$_2$, NEt$_3$, EtOH, 110 °C, 30 min. for **10** or 4 h for **9**.

The synthesis of C5-modified triazole **14** is described in scheme 4. Condensation of salicylamide **1** with 2,4-dimethoxybenzoyl chloride afforded benzoxazinone **12**, which, upon addition of 4-hydrazinobenzoic acid yielded triazole **13**; subsequent deprotection of the phenol group of **13** provided the desired triazole **14**.

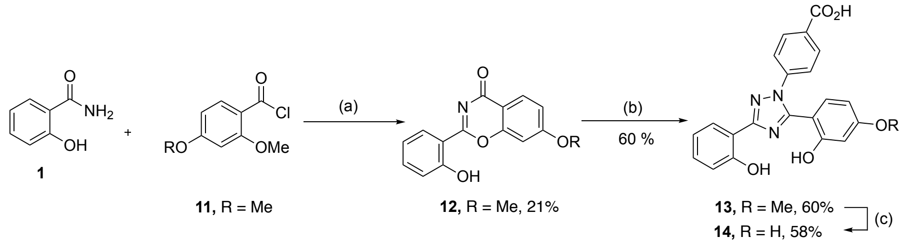

Scheme S3. Synthetic Route for C5-substituted DFX derivatives [a]

[a]Reagents and conditions: (a) xylene, 120 °C, 4 h, (b) 4-CO_2H-PhNHNH$_2$, NEt$_3$, EtOH, 110 °C, 4 h (c) Pyridine.HCl, 225 °C, 2 h.

The synthesis of triazoles **18-22** modified on both the C3 and C5 positions is depicted in Scheme 5. Triazole **18** with a 2-pyridone ring at C5 and the triazole **19** with a 3-pyridol at C5 were obtained from pyridoxazinones **15** and **16** via intramolecular O-arylation, following our reported procedure [3] [2]. Condensation of pyridoxazinone **17** with 4-hydrazinobenzoic acid afforded triazole **20**. Finally, methoxy groups (triazole **21**, R^7 = Me) and acetate groups (triazole **22**, R^7 = Ac) were added to DFX **3a** through alkylation reaction with methyl iodide and esterification reaction using acetic anhydride, respectively.

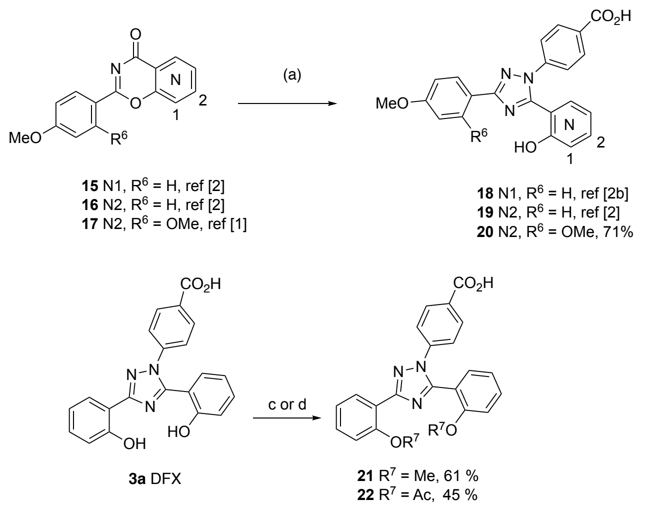

Scheme S4. Synthetic Route for C3 and C5-substituted DFX derivatives

[a] Reagents and conditions: (a) 4-CO_2H-$PhNHNH_2$, EtOH, 110 °C, 1 h AcOH (for **18**) or 4-CO_2H-$PhNHNH_2$, EtOH, 110 °C, 1 h (for **19** and **20**). (c) MeI, K_2CO_3, CH_3CN, reflux, 6h (d) Ac_2O, H_2SO_4, 80 °C, 4 h

Finally, oxadiazole **23** was prepared from benzoxazinone **2** using hydroxylamine and sodium acetate following experimental procedure previously described on pyridoxazinones (Scheme 6).[2]

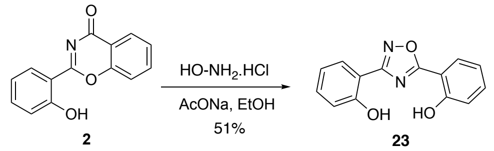

Scheme S5. Synthesis of oxadiazole 23

All reagents were analytical grade. Distilled water was filtered and deionized through a Millipore water purification system.

2. Material and methods

2.a. Experimental procedures

All reagents and solvents were purchased from commercial suppliers and used without further purification (Sigma-Aldrich, TCI, Alfa-Aesar, Fluorochem). All reactions requiring anhydrous conditions were carried out under an inert atmosphere (argon) in oven-dried

glassware (70°C). Anhydrous THF used was distilled over sodium/benzophenone before use. Each reaction was monitored by analytical thin-layer chromatography (TLC) on silica gel 60 F254 plates or on neutral alumina plates and revealed using UV short wavelength (λ= 254 nm). When it is mentioned, purification of compounds in normal phase was carried out by flash chromatography on silica gel (40-60 µm), on neutral alumina 90 or on preparative layer chromatography (PLC) on silica gel. Nuclear Magnetic Resonance spectra (NMR) were recorded on a Bruker Avance spectrometer at 300 or 400 MHz for ^{1}H and 100 MHz for ^{13}C. ^{2}D NMR experiments such as ^{1}H-^{1}H COSY and ^{1}H-^{13}C HSQC experiments were performed to enable signal attributions. Chemical shifts (δ) are given in ppm, referenced to the residual solvent peak (in deuterated dimethyl sulfoxide, DMSO-d6: ^{1}H δ 2.50 ppm and ^{13}C δ 39.5 ppm, in deuterated chloroform, $CDCl_3$: ^{1}H δ 7.26 ppm and ^{13}C δ 77.1 ppm, in CD_3CN ^{1}H 1.94 ppm and ^{13}C δ 118.3 ppm), coupling constant (J) are measured in hertz (Hz). Multiplicity is indicated as follows: s, singlet; d, doublet; t, triplet; q, quadruplet; dd, doublet of doublet; m, multiplet. The melting point were performed on electrothermal IA 91000. UV/Vis absorption spectra were measured using a Cary 50 (Varian) spectrophotometer at 20°C. The purity of compounds was evaluated by HPLC: Column Nucleodur C18 HTec 150 x 4.6 mm; 5 µm Macherey-Nagel), Solvent A, solvent B, flow rate = 1 $mL.min^{-1}$. UV detection at 300 nm. Reactions with microwave were performed using Biotage Initiator™ 2.0 apparatus in a Biotage 10-20 mL microwave vial, at the indicated temperature and period time, on very high absorption mode. Reactions of monowave were performed using Monowave 50 apparatus with 6 mL vial at the indicated temperature and period time. UV/Vis absorption spectra were measured using TECAN spectrophotometer at 37 °C.

Compounds **18**, **19** have not been synthesized during this work. These compounds are described in the literature [3] and have been used directly for the enzyme assays. Synthesis of the following compounds is described in the literature: **15**, **16** and **17**. [2] [3]

2-(2-hydroxyphenyl)-1,3(4H)-benzoxazin-4-one 2: Salicylic acid (3.02 g, 22 mmol) was dissolved in xylene (10 ml). Thionyl chloride (1.6 mL, 22 mmol) was added dropwise at 10-15 °C. The reaction mixture was stirred at 10-15°C for 30 min and heated at 35-40°C for 1 h. Salicylamide (2.99 g, 22 mmol) was added to the reaction mixture at 25-30 °C. After addition, the reaction mixture was gradually heated at 120 °C and stirred for 5 h. Excess of xylene was distilled out. The resulting residue was triturated with methanol to give, after drying under

vacuum at 60°C during 4 h, the pure product. Pale yellow powder; m= 1.90 mg; 36 % yield.^{1}H NMR (400 MHz, DMSO-d$_6$) δ 12.93 (s, 1H), 8.25 – 8.17 (m, 1H), 8.07 (dd, J = 7.8, 1.7, 1H), 7.95 (ddd, J = 8.5, 7.5, 1.7, 1H), 7.80 (dd, J = 8.5, 1.0, 1H), 7.68 – 7.56 (m, 2H), 7.14 – 7.05 (m, 2H).^{13}C NMR (100 MHz, DMSO-d$_6$) δ 164.8, 163.5, 161.8, 153.9, 136.7, 136.0, 129.0, 127.2, 126.8, 119.6, 118.0, 117.8, 117.5, 111.5. Experimental data match with the data reported in the literature [4].

General procedure A for the synthesis of N1-substituted-3,5-bis(2-hydroxyphenyl)-1,2,4-triazol-1-yl analogs: In a sealed tube and under argon atmosphere, the corresponding hydrazine derivatives (1.0 equiv.) and triethylamine (1.1 equiv.) were dissolved in absolute ethanol (0.12 M). the corresponding benzoxazinone (1.0 eq) was added and the reaction mixture was refluxed for 4h at 110 °C. The solution was then allowed to cool to room temperature and water was added. The mixture was concentrated to a total volume of 50 % under reduced pressure. A 1 M aqueous solution of HCl was added to the mixture to reach pH = 3 or pH = 5 depending of the expecting product. The precipitate was filtrated and the resulting solid was purified either by precipitation and filtration or by extraction then by column chromatography on silica gel.

4-(3,5-bis(2-hydroxyphenyl)-1,2,4-triazol-1-yl)benzoic acid DFX 3a: The compound is obtained following general procedure A from 4-hydrazinobenzoic acid (191 mg, 1.25 mmol), triethylamine (191 µL, 1.37 mmol) and 2-(2-hydroxyphenyl)-1,3(4H)-benzoxazin-4-one **2** (300 mg, 1.25 mmol). After addition of 1 M aqueous solution of HCl to reach pH = 3, the resulting solid was filtered and washed with water and dried for 12h to give the desired product. Beige powder; 430 mg; 92 %**.** HPLC (A: Water 30 % + 0.1 % TFA, B: CH_3CN 70 % + 0.08 % TFA): t_R = 3.33 min Purity > 97 %**.** ^{1}H NMR (400 MHz, DMSO-d$_6$) δ 12.79 (s, 1H), 10.79 (s, 1H), 10.05 (s, 1H), 8.05 (dd, J = 7.8, 1.7, 1H), 7.99 (m, 2H), 7.55 (m, 3H), 7.39 (m, 2H), 7.08 – 6.95 (m, 3H), 6.87 (d, J = 8.2, 1H). ^{13}C NMR (101 MHz, DMSO-d$_6$) δ 166.4, 159.8, 156.3, 155.1, 152.0, 141.1, 132.5, 131.4, 131.0, 130.6, 130.3, 126.7, 123.3, 119.7, 119.4, 117.1, 116.1, 114.4, 113.6. Experimental data matches the data reported in the literature.[5]

3-(3,5-bis(2-hydroxyphenyl)-1H-1,2,4-triazol-1-yl)benzoic acid 3b: The compound is obtained following the general procedure D from 3-hydrazinylbenzoic acid (127 mg, 0.84 mmol) and 2-(2-hydroxyphenyl)-1,3(4H)-benzoxazin-4-one **2** (200 mg, 0.84 mmol). The resulting solid was filtered and washed with water. The crude product was purified via column chromatography

on silica gel (0-15% MeOH in DCM) to give pure compound. A pale-yellow powder; 171 mg, 55%. HPLC (A: Water 30 % + 0.1 % TFA, B: CH_3CN 70 % + 0.08 % TFA) t_R = 3.18 min Purity > 96 %. ^1H NMR (400 MHz, DMSO-d_6) δ 13.28 (bs, 1H), 10.89 (s, 1H), 10.09 (s, 1H), 8.06 (dd, J = 7.8, 1.7, 1H), 8.04 – 7.94 (m, 2H), 7.63 (d, J = 7.9, 1H), 7.59 – 7.49 (m, 2H), 7.37 (m, 2H), 7.06 – 6.90 (m, 3H), 6.88 (d, J = 8.2, 1H). The spectroscopic data are in accordance with literature description.[6]

2,2'-(1H-1,2,4-triazole-3,5-diyl)diphenol 3c: In a monowave tube, under argon atmosphere, 2-(2-hydroxyphenyl)-1,3(4H)-benzoxazin-4-one **2** (50 mg, 0.21 mmol) was dissolved in absolute ethanol (3 mL). 1M hydrazine in THF (210 µL, 0.21 mmol) were added to the solution. The tube was sealed and the reaction mixture was heated to 110 °C at full power in the Monowave for 40 min (heat as fast as possible mode). The reaction mixture was cooled to room temperature. The mixture was filtered and the resulting solid was washed with ethanol and triturated in diethyl ether. After filtration of the suspension, the solid was dried under vacuum at 60°C during 4 h to provide the desired product. White powder; 31 mg; 58% yield. HPLC (A: Water 40 % + 0.1 % TFA, B: CH_3CN 60 % + 0.08 % TFA) t_R = 7.88 min Purity > 98 %. ^1H NMR (400 MHz, DMSO-d_6) δ 14.07 (s, 1H), 11.17 (s, 2H), 8.03 (dd, J = 7.9, 1.6, 2H), 7.44 – 7.28 (m, 2H), 7.14 – 6.91 (m, 4H). Experimental data match with the data reported in the literature.[4]

2,2'-(1-(tert-butyl)-1H-1,2,4-triazole-3,5-diyl)diphenol 3d: In a Monowave tube under argon atmosphere, 2-(2-hydroxyphenyl)-1,3(4H)-benzoxazin-4-one **2** (100 mg, 0.42 mmol) was dissolved in absolute ethanol (4.5 mL). Triethylamine (116 µL, 0.84 mmol) and tert-butylhydrazine hydrochloride (52 mg, 0.42 mmol) were added to the solution. The tube was sealed and the reaction mixture was heated for 1h at 110 °C (heat as fast as possible mode). The solution was cooled to room temperature and water was added. The mixture was concentrated to a total volume of 50 % under reduced pressure and acetic acid was added to reach pH = 5. The mixture was extracted three times with dichloromethane, dried over sodium sulfate, filtered and concentrated in vacuo. The resulting solid was dried under vacuum at 50 °C for 4 h to afford the desired product. White powder; 75 mg; 58 % yield. HPLC (A: Water 30 %, B: CH_3CN 70 %) t_R = 4.92 min Purity > 99 %.^1H NMR (400 MHz, DMSO-d_6) δ 10.99 (s, 1H), 10.11 (s, 1H), 7.97 (dd, J = 8.1, 1.7, 1H), 7.39 (m, 1H), 7.35 – 7.27 (m, 2H), 7.01 – 6.91 (m, 4H),

1.50 (s, 9H). ^{13}C NMR (100 MHz, DMSO-d_6) δ 157.8, 156.5, 156.1, 150.9, 132.1, 131.4, 131.2, 126.6, 119.9, 119.2, 118.6, 117.2, 116.1, 114.6, 61.8, 29.9 (3C). HRMS (ESI) m/z: Calcd $C_{18}H_{20}N_3O_2$ $[M+H]^+$ 310.1550. Found: 310.1547.

(3,5-bis(2-hydroxyphenyl)-1H-1,2,4-triazol-1-yl)(phenyl)methanone 3e: In a sealed tube and under an atmosphere of argon, benzhydrazide (127 mg, 0.84 mmol) were allowed to react with 2-(2-hydroxyphenyl)-1,3(4H)-benzoxazin-4-one **2** (200 mg, 0.84 mmol) and acetic acid (72 µL, 1.67 mmol) for about 5 hours in absolute EtOH (0.08 M) at 110 °C. After cooling to rt., water was added to the reaction mixtures before removing EtOH under reduced pressure. The aqueous solution was extracted 3 times with DCM. The combined organic layers were washed successively with a saturated aqueous $NaHCO_3$ solution and brine, dried over Na_2SO_4, filtered and concentrated under reduced pressure. The crude product was purified on a preparative layer chromatography on silica gel (EtOAc:Cyclohexane 6:4 V:V + 1% MeOH) to furnish the desired product. White powder; 89 mg; 30% yield. HPLC (A: Water 30 % + 0.1 % TFA, B: CH_3CN 70 % + 0.08 % TFA) t_R = 4.44 min, Purity > 94 %**.** ^1H NMR (400 MHz, DMSO-d_6) δ 14.21 (bs, 1H, H_{24}), 11.10 (bs, 1H, H_{25}), 8.33 – 8.12 (m, 3H), 7.78 – 7.71 (m, 1H), 7.64 – 7.54 (m, 4H), 7.50 (t, J = 7.4, 1H), 7.42 (d, J = 7.5, 1H), 7.31 – 7.22 (m, 1H), 6.95 (d, J = 8.2, 1H), 6.75 (t, J = 7.5, 1H). The analytical data are in agreement with literature report.[7]

Synthesis of (±)- (1R, 4R)-Ethyl 4-(3,5-bis(2-hydroxyphenyl)-1H-1,2,4-triazol-1-yl)cyclohexanecarboxylate trans 3fa

(±)-Ethyl 4-[(tert-butyloxycarbonyl)hydrazono]cyclohexanecarboxylate: Tert-butyl carbazate (1.4 g, 10.5 mmol) was dissolved in toluene (14.8 mL). Ethyl 4-oxocyclohexanecarboxylate (1.68 mL, 10.5 mmol) was added, and the reaction stirred overnight at rt. The mixture was dried over Na_2SO_4, filtered and the solvent was co-evaporated with MeOH to give the desired product. White solid; 3.0 g, quantitative yield. HPLC (A: Water 30 % + 0.1 % TFA, B: CH_3CN 70 % + 0.08 % TFA) t_R = 5.92 min Purity > 97%**.** ^1H NMR (400 MHz, DMSO-d_6) δ 9.55 (s, 1H), 4.07 (q, J = 7.1, 2H), 2.78 – 2.69 (m, 1H), 2.59 (tt, J = 10.3, 3.8, 1H), 2.28 (dt, J = 8.7, 4.1, 1H), 2.23 – 2.13 (m, 1H), 2.02 – 1.85 (m, 3H), 1.63 – 1.46 (m, 2H), 1.42 (s, 9H), 1.18 (t, J = 7.1, 3H). ^{13}C NMR (100 MHz, DMSO-d_6) δ 174.3, 153.6, 79.0, 60.1, 40.9, 33.2, 29.0, 28.3 (3C), 27.6, 25.2, 14.3. One of the carbons does not relax, and thus is not visible on

the ^{13}C NMR spectrum. HRMS (ESI) m/z: Calcd for $C_{14}H_{24}N_2O_4Na$ $[M+Na]^+$ 307.1628. Found 307.1628.

(±)-Ethyl 4-[2-(tert-butyloxycarbonyl)hydrazino]cyclohexanecarboxylate : To a solution of tert-butyl 2-(4-(ethoxycarbonyl)cyclohexylidene)hydrazinecarboxylate (3.0 g, 10.4 mmol) in THF (12 mL) was added sodium cyanoborohydride (830 mg, 13.0 mmol) in small portions. The mixture was stirred at rt for 1 h and then treated overnight with a solution of p-toluenesulfonic acid (1.8 g, 10.4 mmol) in THF (12 mL). The solvent was evaporated under reduced pressure and the residue was suspended in DCM. The solution was successively washed with a solution of saturated aqueous ammonium chloride, saturated aqueous sodium bicarbonate and brine. The organic layer was dried over Na_2SO_4, filtered and evaporated to a colorless oil. The two diastereoisomers were separated by column chromatography on silica gel (EtOAc:Cyclohexane (3:7 V:V)). The first compound eluted was the cis-isomer of the expecting product**.** The second compound eluted was the trans-isomer of the expecting product. Only the trans compound has been described.

(±)-Trans-ethyl 4-[2-(tert-butyloxycarbonyl)hydrazino]cyclohexanecarboxylate: White powder; 798 mg; 27% yield. ^1H NMR (400 MHz, DMSO-d_6) δ 8.16 (s, 1H), 4.24 (bs, 1H), 4.03 (q, J = 7.1, 2H), 2.65 – 2.54 (m, 1H), 2.20 (tt, J = 11.8, 3.5, 1H), 1.87 (dd, J = 13.2, 2.5, 2H), 1.78 (dd, J = 13.0, 3.0, 2H), 1.38 (m, 9H), 1.34 – 1.22 (m, 2H), 1.16 (t, J = 7.1, 3H), 1.00 (td, J = 13.2, 3.3 Hz, 2H). ^{13}C NMR (100 MHz, DMSO-d_6) δ 175.1, 156.7, 78.4, 59.8, 57.5, 42.3, 29.9 (2C), 28.4 (3C), 27.1 (2C), 14.3**.** HRMS (ESI) m/z: Calcd for $C_{14}H_{26}N_2O_4Na$ $[M+Na]^+$ 309.1785. Found 309.1784

(±)-Trans-ethyl-4-hydrazinylcyclohexanecarboxylate hydrochloride : Trans-ethyl 4-[2-(tert-butyloxycarbonyl)hydrazino]cyclohexanecarboxylate (798 mg, 2.8 mmol) was dissolved in 1,4-dioxane (10.1 mL). A hydrogen chloride solution 4.0 M in dioxane (5.7 mL) was added and the mixture stirred at r.t. overnight. The solid was filtered over a sintered glass frit and triturated 3 times with diethyl ether to give the desired product. White powder; 500 mg, 96% yield. ^1H NMR (400 MHz, DMSO-d_6) δ 7.36 (bs, 3H), 4.05 (m, 1H), 4.04 (q, J = 7.1, 2H), 2.94 – 2.81 (m, 1H), 2.28 – 2.17 (m, 1H), 2.11 – 2.02 (m, 2H), 2.01 – 1.92 (m, 2H), 1.41 – 1.21 (m, 3H), 1.18 (m,

1H), 1.15 (t, J = 7.1, 3H). ^{13}C NMR (75 MHz, DMSO-d_6) δ 174.6, 60.1, 58.2, 41.7, 27.1 (2C), 26.8 (2C), 14.5. HRMS (ESI) m/z: Calcd for $C_9H_{19}N_2O_2$ $[M+H]^+$ 187.1441. Found 187.1437

(±)-(1R, 4R)-Ethyl 4-(3,5-bis(2-hydroxyphenyl)-1H-1,2,4-triazol-1-yl)cyclohexanecarboxylate trans 3fa: The compound is obtained by the general procedure A from 2-(2-hydroxyphenyl)-1,3(4H)-benzoxazin-4-one **2** (192 mg, 0.80 mmol) and trans-ethyl -4 hydrazinylcyclohexanecarboxylate hydrochloride (224 mg, 1.20 mmol) and triethylamine (372 µL, 2.41 mmol) in absolute ethanol (9.6 mL). After cooling to rt., water was added to the mixture before removing EtOH under reduced pressure. The aqueous solution was extracted 3 times with EtOAc. The organic phases were washed with brine, dried over Na_2SO_4, filtered and concentrated in vacuo. The crude product was purified through a pad of silica (EtOAc:Cyclohexane (5:5 V:V)) to furnish the desired product. White powder; 255 mg; 78% yield. HPLC (A: Water 30 % + 0.1 % TFA, B: CH_3CN 70 % + 0.08 % TFA) t_R = 5.92 min Purity > 97%**.** ^1H NMR (300 MHz, DMSO-d_6) δ 11.03 (s, 1H), 10.34 (s, 1H), 7.98 – 7.95 (m, 1H), 7.46 – 7.40 (m, 2H), 7.33 – 7.27 (m, 1H), 7.10 – 6.92 (m, 4H), 4.07 – 4.00 (m, 3H), 2.46 – 2.35 (m, 1H), 2.03 – 1.96 (m, 6H), 1.41 – 1.34 (m, 2H), 1.16 (t, J = 7.1 Hz, 3H)**.** ^{13}C NMR (75 MHz, DMSO-d_6) δ 174.1, 158.9, 155.9, 155.1, 150.9, 131.9, 131.0, 130.6, 125.9, 119.2, 119.1, 116.6, 116.0, 113.9, 113.9, 59.5, 57.0, 40.7, 30.7 (2C), 27.2 (2C), 13.8. HRMS (ESI) m/z: Calcd for $C_{23}H_{26}N_3O_4$ $[M+H]^+$ 408.1918. Found 408.1917.

(±)-(1R, 4R)-4-(3,5-bis(2-hydroxyphenyl)-1H-1,2,4-triazol-1-yl)cyclohexanecarboxylic acid 3fb: To a solution of **3fa** (100 mg, 0.25 mmol) dissolved in dry MeOH (4.7 mL), potassium hydroxide (138 mg, 2.46 mmol) was added under an atmosphere of argon. The reaction was heated at reflux for 4 h. After cooling to rt., brine was poured into the mixture and then extracted 3 times with EtOAc. The aqueous layer was acidified with aqueous hydrochloric acid (1 M) until pH 2, and then extracted 3 times with EtOAc. The organic layers were combined, dried and concentrated under reduced pressure to furnish the desired product. Pale pink powder; 83 mg; 87% yield. HPLC (A: Water 30 % + 0.1 % TFA, B: CH_3CN 70 % + 0.08 % TFA) t_R = 2.60 min Purity > 97%**.** ^1H NMR (400 MHz, DMSO-d_6) δ 12.08 (s, 1H), 11.05 (s, 1H), 10.35 (s, 1H), 7.98 (dd, J = 7.8, 1.4, 1H), 7.48 – 7.40 (m, 2H), 7.34 – 7.28 (m, 1H), 7.06 (d, J = 8.0, 1H), 7.03 – 6.92 (m, 3H), 4.11 – 3.99 (m, 1H), 2.39 – 2.28 (m, 1H), 2.08 – 1.94 (m, 6H), 1.45 – 1.31

(m, 2H). ^{13}C NMR (100 MHz, DMSO-d_6) δ 176.1, 159.2, 156.1, 155.4, 151.2, 132.2, 131.3, 130.8, 126.2, 119.5, 119.4, 116.8, 116.2, 114.2, 114.1, 57.4, 41.1, 31.1 (2C), 27.5 (2C). HRMS (ESI) m/z: Calcd for $C_{21}H_{22}N_3O_4$ $[M+H]^+$ 380.1605. Found 380.1604.

Ethyl 2-(3,5-bis(2-hydroxyphenyl)-1H-1,2,4-triazol-1-yl) acetate 3g: The compound is obtained following the general procedure A from ethyl hydrazinoacetate hydrochloride (129 mg, 0.84 mmol), triethylamine (232 µL, 1.67 mmol) and 2-(2-hydroxyphenyl)-1,3(4H)-benzoxazin-4-one **2** (200 mg, 0.84 mmol). After addition of 1 M aqueous solution of HCl, the pH of the mixture reaches to pH = 5. The precipitate was filtered and the resulting solid washed with water and dried for 12h to give the desired product. Pink pale powder; 218 mg; 77% yield. HPLC (A: Water 30 %, B: CH_3CN 70 %) t_R = 3.43 min Purity > 99 %. ^1H NMR (400 MHz, DMSO-d_6) δ 10.91 (s, 1H), 10.55 (s, 1H), 7.96 (dd, J = 7.8, 1.7, 1H), 7.50 – 7.39 (m, 2H), 7.34 (m, 1H), 7.05-6.93 (m, 4H), 5.16 (s, 2H), 4.08 (q, J = 7.1, 2H), 1.12 (t, J = 7.1, 3H). ^{13}C NMR (100 MHz, DMSO-d_6) δ 166.8, 159.3, 156.1, 155.9, 153.4, 132.5, 131.3, 131.1, 126.3, 119.6, 119.5, 116.9, 116.3, 113.7, 113.4, 61.3, 50.6, 13.83. HRMS (ESI) m/z: Calcd for $C_{18}H_{18}N_3O_4$ $[M+H]^+$ 340.1292. Found 340.1293.

2-(3,5-bis(2-hydroxyphenyl)-1H-1,2,4-triazol-1-yl)acetic acid 3h: Ethyl 2-(3,5-bis(2-hydroxyphenyl)-1H-1,2,4-triazol-1-yl)acetate **3g** (84 mg, 0.25 mmol) and potassium hydroxide (91 mg, 1.6 mmol) were dissolved in absolute ethanol (2 mL). The reaction mixture was stirred 12 h at room temperature. A solution of brine was added the mixture. A 1 M aqueous solution of HCl was added to the mixture to reach pH =3. The resulting solid was filtered and washed with water and dried under vacuum at 50 °C for 5 h to give the desired product. White solid; 72 mg; 93 % yield. HPLC (A: Water 40 % + 0.1 % TFA, B: CH_3CN 60 % + 0.08 % TFA) t_R = 3.83 min Purity > 95 %. ^1H NMR (400 MHz, DMSO-d_6) δ 13.21 (s, 1H), 10.96 (s, 1H), 10.71 (s, 1H), 7.96 (dd, J = 7.8, 1.8, 1H), 7.45-7.39 (m, 2H), 7.35-7.31 (m, 1H), 7.14 (dd, J = 8.6, 1.2, 1H), 7.00-6.95 (m, 3H), 5.07 (s, 2H). ^{13}C NMR (100 MHz, DMSO-d_6) δ 168.2, 159.2, 156.2, 155.0, 153.4, 132.3, 131.2, 131.0, 126.3, 119.5, 119., 116.9, 116.5, 113.9, 113.6, 50.7. HRMS (ESI) m/z: Calcd for $C_{16}H_{12}N_3O_4$ [M] 310.0833. Found: 310.0831.

Methyl 4-(3,5-bis(2-hydroxyphenyl)-1H-1,2,4-triazol-1-yl)benzoate 3i: In a round-bottomed flask, 4-(3,5-bis(2-hydroxyphenyl)-1,2,4-triazol-1-yl)benzoic acid **DFX-3a** (500 mg, 1.34 mmol) was dissolved in methanol (18 mL). The solution was cooled to 0° C and acetyl chloride (4.8 mL, 0.067 mmol) was added dropwise to the mixture. The mixture was stirred overnight at room temperature. The reaction mixture was cooled to 0 °C and a saturated aqueous solution of sodium hydrogen carbonate was added dropwise. The two layers were separated in a separating funnel and the aqueous layer was extracted three times with dichloromethane. The combined organic layers were washed with brine, dried over anhydrous sodium sulfate, filtered and concentrated in vacuo. The resulting solid was dried under vacuum at 60°C during 4h to furnish the desired compound. White powder; 434 mg; 84 % yield. HPLC (A: Water 30 %, B: CH_3CN 70 %) t_R = 6.17 min Purity > 99 %. ^{1}H NMR (400 MHz, DMSO-d_6) δ 10.78 (s, 1H), 10.04 (s, 1H), 8.05 (dd, J = 7.8, 1.6, 1H), 8.02 (d, J = 8.4, 2H), 7.59 (d, J = 8.4, 2H), 7.56 (dd, J = 7.7, 1.6, 1H), 7.39 (m, 2H), 7.08 – 6.94 (m, 3H), 6.86 (d, J = 8.2, 1H), 3.86 (s, 3H). ^{13}C NMR (100 MHz, DMSO-d_6) δ 165.3, 159.9, 156.3, 155.1, 152.1, 141.5, 132.5), 131.4, 131.1, 130.1 (2C), 129.3, 126.8, 123.4 (2C), 119.7, 119.4, 117.1, 116.1, 114.3, 113.6, 52.3. Experimental data match with the data reported in the literature.[8]

General procedure B for the synthesis of deferasirox-amino esters from deferasirox 3ja, 3ka, 3la and 3ma: To a solution of 4-(3,5-bis(2-hydroxyphenyl)-1,2,4-triazol-1-yl)benzoic acid DFX **3a** (1.0 equiv.) dissolved in anhydrous DCM (0.013 M), thionyl chloride (10.0 equiv.) was added dropwise at 0 °C, and the reaction was stirred under an atmosphere of argon at reflux for 5 h. The volatiles were removed under reduced pressure. Meanwhile, the corresponding L-amino acid methyl ester hydrochloride (1.2 equiv.) was dissolved in DCM (0.036 M) under argon atmosphere, and triethylamine (2.5 equiv.) was added. The reaction was stirred for 20 min at rt. This mixture was then cooled at 0 °C and the previous formed aroyl chloride taken up in DCM (0.03 M) was added dropwise. The reaction was stirred overnight at rt. under an atmosphere of argon. The reaction was quenched with a saturated aqueous $NaHCO_3$ solution. The mixture was extracted 3 times with DCM. The combined organic layers were treated with aqueous hydrochloric acid 1 M until pH 2 and extracted 3 times with DCM. Finally, the organic layer was washed with brine, dried over Na_2SO_4 and concentrated. If necessary, the crude product was purified via column chromatography on silica gel.

General procedure C for the synthesis of deferasirox-amino acid analogs: 3jb, 3kb, 3lb and 3mb : To a solution of DFX-amino ester (1.0 equiv.) dissolved in dry MeOH (0.05 M), potassium hydroxide (10.0 equiv.) was added under an atmosphere of argon. The reaction was heated at reflux for 3 h. After cooling to r.t., brine was poured into the mixture and then extracted 3 times with DCM. The aqueous layer was acidified with aqueous hydrochloric acid (1 M) until pH 2, and then extracted 3 times with EtOAc. The organic layers were combined, dried over anhydrous sodium sulfate, filtered and evaporated under reduced pressure. The resulting solid was dried under vacuum at 50°C during 12 h to furnish the desired product.

Methyl (4-(3,5-bis(2-hydroxyphenyl)-1H-1,2,4-triazol-1-yl)benzoyl)glycinate 3ja. The product was prepared according to the general procedure B from 4-(3,5-bis(2-hydroxyphenyl)-1,2,4-triazol-1-yl)benzoic acid DFX **3a** (130 mg, 0.35 mmol), thionyl chloride (0.26 mL, 3.50 mmol) and glycine methyl ester hydrochloride (50 mg, 0.40 mmol). The product was used for the next step without further purification. Pale yellow powder; 61 mg; 41% yield. HPLC (A: Water 30 % + 0.1 % TFA , B: CH_3CN 70 % + 0.08 % TFA) t_R = 3.02 min Purity > 97%**.** ^1H NMR (400 MHz, DMSO-d_6) δ 10.81 (s, 1H), 10.05 (s, 1H), 9.06 (t, J = 5.8, 1H), 8.06 (dd, J = 7.8, 1.8, 1H), 7.97 – 7.88 (m, 2H), 7.62 – 7.47 (m, 3H), 7.38 (m, 2H), 7.09 – 6.93 (m, 3H), 6.86 (dd, J = 8.4, 1.0, 1H), 4.02 (d, J = 5.8, 2H), 3.66 (s, 3H). ^{13}C NMR (100 MHz, DMSO-d_6) δ 170.3, 165.6, 159.8, 156.3, 155.2, 152.0, 140.3, 133.3, 132.5, 131.4, 131.1, 128.3 (2C), 126.8, 123.3 (2C), 119.7, 119.4, 117.1, 116.1, 114.4, 113.7, 51.8, 41.2. HRMS (ESI) m/z: Calcd for $C_{24}H_{21}N_4O_5$ $[M+H]^+$ 445.1506 Found 445.1507.

(4-(3,5-bis(2-hydroxyphenyl)-1H-1,2,4-triazol-1-yl)benzoyl)glycine 3jb: The product was prepared according to the general procedure C from methyl (4-(3,5-bis(2-hydroxyphenyl)-1H-1,2,4-triazol-1-yl)benzoyl)glycine **3ja** (100 mg, 0.23 mmol) and potassium hydroxide (82 mg, 1.5 mmol) in 3.5 mL anhydrous methanol. White solid; 90 mg; 93 % yield. HPLC (A: Water 40 % + 0.1 % TFA, B: CH_3CN 60 % + 0.08 % TFA) t_R = 3.96 min Purity > 95 %. ^1H NMR (400 MHz, DMSO-d_6) δ 12.65 (s, 1H), 10.82 (s, 1H), 10.06 (s, 1H), 8.94 (t, J = 5.9, 1H), 8.06 (dd, J = 7.8, 1.7, 1H), 7.98 – 7.88 (m, 2H), 7.61 – 7.48 (m, 3H), 7.38 (m, 2H), 7.08 – 6.94 (m, 3H), 6.86 (dd, J = 8.3, 1.0, 1H), 3.93 (d, J = 5.9, 2H). ^{13}C NMR (101 MHz, DMSO-d_6) δ 171.2, 165.4, 159.8, 156.3, 155.2, 152.0, 140.1, 133.5, 132.5, 131.4, 131.1, 128.2 (2C), 126.8, 123.2 (2C), 119.7, 119.4,

117.1, 116.1, 114.4, 113.7, 41.3. HRMS (ESI) m/z: Calcd for $C_{23}H_{19}N_4O_5$ [M+H]$^+$ 431.1350. Found 431.1350.

(S)-methyl (4-(3,5-bis(2-hydroxyphenyl)-1H-1,2,4-triazol-1-yl)benzoyl)-L-phenylalaninate 3ka: The product was prepared according to the general procedure B from 4-(3,5-bis(2-hydroxyphenyl)-1,2,4-triazol-1-yl)benzoic acid **DFX-3a** (200 mg, 0.54 mmol), thionyl chloride (0.38 mL, 5.4 mmol) and , (L)-Phenylalanine methyl ester hydrochloride (132 mg, 0.63 mmol). The product was used for the next step without further purification. Pale yellow powder; 177 mg; 63% yield. HPLC (A: Water 30 % + 0.1 % TFA, B: CH_3CN 70 % + 0.08 % TFA) t_R = 5.56 min Purity > 96%**.** ^{1}H NMR (400 MHz, DMSO-d_6) δ 10.80 (s, 1H), 10.03 (s, 1H), 8.96 (d, J = 7.8 Hz, 1H), 8.05 (dd, J = 7.8, 1.7 Hz, 1H), 7.90 – 7.81 (m, 2H), 7.57 – 7.49 (m, 3H), 7.42 – 7.34 (m, 2H), 7.32 – 7.24 (m, 4H), 7.22 – 7.16 (m, 1H), 7.05 – 6.94 (m, 3H), 6.86 (dd, J = 8.3, 1.0 Hz, 1H), 4.70-4.65 (m, 1H), 3.64 (s, 3H), 3.20 – 3.05 (m, 2H). ^{13}C NMR (100 MHz, DMSO-d_6) δ 172.1, 165.3, 159.8, 156.3, 155.1, 151.9, 140.1, 137.6, 133.3, 132.5, 131.4, 131.1, 129.1(2C), 128.4 (2C), 128.3 (2C), 126.7, 126.5, 123.2 (2C), 119.6, 119.4, 117.1, 116.1, 114.4, 113.6, 54.3), 52.1, 36.1. Experimental data match with the data reported in the literature.[9]

(S)-(4-(3,5-bis(2-hydroxyphenyl)-1H-1,2,4-triazol-1-yl)benzoyl)phenylalanine 3kb: The product was prepared according to the general procedure C from (S)-methyl (4-(3,5-bis(2-hydroxyphenyl)-1H-1,2,4-triazol-1-yl)benzoyl)-L-phenylalaninate **3ka** (90 mg, 0.17 mmol) and potassium hydroxide (61 mg, 1.09 mmol) in 2.5 mL anhydrous methanol. Yellow powder; 72 mg; 83% yield. HPLC (A: Water 40 % + 0.1 % TFA, B: CH_3CN 60 % + 0.08 % TFA) t_R = 10.0 min Purity > 95 %. ^{1}H NMR (400 MHz, DMSO-d_6) δ 12.82 (s, 1H), 10.81 (s, 1H), 10.04 (s, 1H), 8.81 (d, J = 8.1 Hz, 1H), 8.05 (dd, J = 7.8, 1.7, 1H), 7.89 – 7.82 (m, 2H), 7.57 – 7.47 (m, 3H), 7.38 (m, 2H), 7.34 – 7.23 (m, 4H), 7.22 – 7.13 (m, 1H), 7.05 – 6.94 (m, 3H), 6.86 (dd, J = 8.3, 1.0, 1H), 4.62 (m, 1H), 3.20 (m, 1H), 3.05 (m, 1H). ^{13}C NMR (101 MHz, DMSO-d_6) δ 173.1, 165.2, 159.8, 156.3, 155.2, 152.0, 140.0, 138.1, 133.5, 132.5, 131.4, 131.1, 129.0 (2C), 128.3 (2C), 128.2 (2C), 126.7, 126.4, 123.1 (2C), 119.7, 119.4, 117.0, 116.1, 114.4, 113.7, 54.3, 36.2. HRMS (ESI) m/z: Calcd for $C_{30}H_{25}N_4O_5$ [M+H]$^+$ 521.1819. Found: 521.1801.

(S)-methyl 2-(4-(3,5-bis(2-hydroxyphenyl)-1H-1,2,4-triazol-1-yl)benzamido)-3-(1H-indol-3-yl)propanoate 3la: The product was prepared according to the general procedure B from **DFX-3a** (200 mg, 0.54 mmol) and (S)-methyl 2-amino-3-(1H-indol-3-yl)propanoate hydrochloride (156 mg, 0.64 mmol). The product was used for the next step without further purification. Orange powder; 194 mg; 63 % yield. HPLC (A: Water 30 % + 0.1 % TFA, B: CH_3CN 70 % + 0.08 % TFA) t_R = 4.74 min Purity > 97%.^{1}H NMR (400 MHz, DMSO-d_6) δ 10.83 (bs, 1H), 10.81 (s, 1H), 10.03 (s, 1H), 8.93 (d, J = 7.5, 1H), 8.05 (dd, J = 7.7, 1.7, 1), 7.93 – 7.86 (m, 2H), 7.58 – 7.49 (m, 4H) 7.42 – 7.30 (m, 3H), 7.21 (d, J = 2.3, 1H), 7.09 – 6.95 (m, 5H), 6.86 (dd, J = 8.3, 1.0, 1), 4.75 – 4.67 (m, 1H), 3.64 (s, 3H), 3.30 – 3.17 (m, 2H). ^{13}C NMR (100 MHz, DMSO-d6) δ 172.4, 165.3, 159.8, 156.3, 155.2, 152.0, 140.1, 136.1, 133.3, 132.5, 131.4, 131.1, 128.4 (2C), 127.0, 126.8, 123.6, 123.1 (2C), 121.0, 119.7, 119.4, 118.4, 118.0, 117.1, 116.1, 114.4, 113.7, 111.5, 109.9, 53.9, 52.0, 26.6. HRMS (ESI) m/z : Calcd for $C_{33}H_{28}N_5O_5$ $[M+H]^+$ 574.2085. Found 574.2084.

((S)-2-(4-(3,5-bis(2-hydroxyphenyl)-1H-1,2,4-triazol-1-yl)benzamido)-3-(1H-indol-3-yl)propanoic acid 3lb: The product was prepared according to the general procedure C from (S)-methyl 2-(4-(3,5-bis(2-hydroxyphenyl)-1H-1,2,4-triazol-1-yl)benzamido)-3-(1H-indol-3-yl)propanoate **3la** (70 mg, 0.12 mmol) and potassium hydroxide (67 mg, 1.2 mmol) in 2.5 mL anhydrous methanol. Brown-orange powder; 56.7 mg; 83%. HPLC (A: Water 30 % + 0.1 % TFA, B: CH_3CN 70 % + 0.08 % TFA) t_R = 5.35 min Purity > 97%.^{1}H NMR (400 MHz, DMSO-d_6) δ 12.69 (bs, 1H), 10.81 (s, 2H), 10.02 (s, 1H), 8.77 (d, J = 7.8, 1H), 8.04 (dd, J = 7.7, 1.8, 1H), 7.93 – 7.84 (m, 2H), 7.59 – 7.47 (m, 4H), 7.42 – 7.34 (m, 2H), 7.32 (d, J = 8.0, 1H), 7.20 (d, J = 1.8, 1H), 7.10 – 6.93 (m, 5H), 6.85 (d, J = 8.2, 1H), 4.74 – 4.63 (m, 1H), 3.26 - 3.17 (m, 2H). ^{13}C NMR (100 MHz, DMSO-d6) δ 173.5, 165.2, 159.8, 156.3, 155.2, 152.0, 140.0, 136.1, 133.6, 132.5, 131.4, 131.1, 128.4 (2C), 127.1, 126.8, 123.5, 123.1 (2C), 121.0, 119.7, 119.4, 118.4, 118.1, 117.1, 116.1, 114.4, 113.7, 111.4, 110.4, 53.7, 26.6. HRMS (ESI) m/z: Calcd for $C_{32}H_{26}N_5O_5$ $[M+H]^+$ 560.1928. Found 560.1928.

(S)-methyl 1-(4-(3,5-bis(2-hydroxyphenyl)-1H-1,2,4-triazol-1-yl)benzoyl)pyrrolidine-2-carboxylate 3ma: The product was prepared according to the general procedure B from **DFX-3a** (200 mg, 0.54 mmol) and (S)-methyl pyrrolidine-2-carboxylate hydrochloride (106.5 mg, 0.25 mmol). The crude product was purified via column chromatography on silica gel

(DCM:MeOH (98:2 V:V)) to give the desire product. Light orange powder; 162 mg; 77% yield. HPLC (A: Water 30 % + 0.1 % TFA, B: CH_3CN 70 % + 0.08 % TFA) t_R = 3.79 min Purity > 99%.^{1}H NMR (400 MHz, DMSO-d6) δ 10.83 (s, 1H), 10.08 (s, 1H), 8.06 (dd, J = 7.8, 1.5, 1H), 7.67 – 7.59 (m, 2H), 7.57 – 7.50 (m, 3H), 7.43 – 7.34 (m, 2H), 7.07 – 6.95 (m, 3H), 6.88 (d, J = 8.2, 1H), 4.52 – 4.46 (m, 1H), 3.67 (s, 3H), 3.56 – 3.48 (m, 2H), 2.36 – 2.22 (m, 1H), 2.01 – 1.80 (m, 3H). ^{13}C NMR (100 MHz, DMSO-d6) δ 172.2, 167.2, 159.8, 156.3, 155.3, 151.9, 139.0, 135.8, 132.5, 131.4, 131.0, 128.2 (2C), 126.7, 123.3 (2C), 119.7, 119.4, 117.0, 116.1, 114.4, 113.7, 58.9, 51.9, 49.5, 28.9, 25.0. HRMS (ESI) m/z: Calcd for $C_{27}H_{25}N_4O_5$ $[M+H]^+$ 485.1819. Found 485.1819

(S)-1-(4-(3,5-bis(2-hydroxyphenyl)-1H-1,2,4-triazol-1-yl)benzoyl)pyrrolidine-2-carboxylic acid-3mb: The product was prepared according to the general procedure C from (S)-methyl 1-(4-(3,5-bis(2-hydroxyphenyl)-1H-1,2,4-triazol-1-yl)benzoyl)pyrrolidine-2-carboxylate **3ma** (770 mg, 0.16 mmol) and potassium hydroxide (90 mg, 1.6 mmol) in 2.5 mL anhydrous methanol. Yellowish powder, 56.7 mg, 83% yield. HPLC (A: Water 30 % + 0.1 % TFA, B: CH_3CN 70 % + 0.08 % TFA) t_R = 3.65 min Purity > 99%. ^{1}H NMR (400 MHz, DMSO-d_6) δ 12.59 (bs, 1H, H, H_{36}), 10.84 (s, 1H), 10.07 (s, 1H), 8.05 (dd, J = 7.8, 1.5, 1H), 7.64 – 7.58 (m, 2H), 7.56 – 7.44 (m, 3H), 7.42 – 7.33 (m, 2H), 7.06 – 6.94 (m, 3H), 6.90 – 6.85 (m, 1H), 4.45 – 4.32 (m, 1H), 3.62 – 3.44 (m, 2H), 2.34 – 2.21 (m, 1H), 1.94 – 1.76 (m, 3H). ^{13}C NMR (100 MHz, DMSO-d6) δ 173.1, 167.1, 159.8, 156.3, 155.3, 151.9, 138.9, 136.1, 132.5, 131.4, 131.1, 128.2 (2C), 126.7, 123.3 (2C), 119.7, 119.4, 117.1, 116.2, 114.4, 113.7, 59.0, 49.5, 28.9, 25.0. HRMS (ESI) m/z: Calcd for $C_{26}H_{23}N_4O_5$ $[M+H]^+$ 471.1663. Found 471.1663.

2,2’-(1-phenyl-1H-1,2,4-triazole-3,5-diyl)diphenol 3n: The compound is obtained following the general procedure A from phenyl hydrazine (165 µL, 1.67 mmol) and 2-(2-hydroxyphenyl)-1,3(4H)-benzoxazin-4-one **2** (400 mg, 1.67 mmol) without triethylamine. After the addition of aqueous solution of 1M HCl to reach pH = 3, the two layers were separated in a separating funnel and the aqueous layer was extracted three times with EtOAc. The combined organic layers were washed with brine, dried over sodium sulfate, filtered and concentrated in vacuo. The resulting solid was dried under vacuum at 50 °C for 4h to give the desired product. White powder; 495 mg; 90% yield. HPLC (A: Water 30 % , B: CH_3CN 70 %) t_R = 6.88 min Purity > 99 %. ^{1}H NMR (400 MHz, DMSO-d_6) δ 10.89 (s, 1H), 10.06 (s, 1H), 8.05 (dd, J = 7.8, 1.7, 1H), 7.49 – 7.42 (m, 6H), 7.37 (m, 2H), 7.07 – 6.97 (m, 2H), 6.94 (m, 1H), 6.88 (dd, J = 8.3, 1.0, 1H). ^{13}C

NMR (100 MHz, DMSO-d_6) δ 159.5, 156.3, 155.4, 151.7, 137.7, 132.2, 131.3, 130.9, 129.1 (2C), 128.6, 126.6, 123.7 (2C), 119.6, 119.2, 117.0, 116.1, 114.5, 113.7. Experimental data matches the data reported in the literature.[1]

4-(3,5-bis(2-hydroxyphenyl)-1H-1,2,4-triazol-1-yl)benzonitrile 3o: The compound is obtained following the general procedure A from 4-cyanophenylhydrazine hydrochloride (213 mg, 1.25 mmol), triethylamine (349 µL, 2.5 mmol) and 2-(2-hydroxyphenyl)-1,3(4H)-benzoxazin-4-one **2** (300 mg, 1.25 mmol). After the addition of an aqueous solution of 1M HCl the mixture reaches to pH = 5. The two layers were separated in a separating funnel and the aqueous layer was extracted three times with ethyl acetate. The combined organic layers were washed with brine, dried over sodium sulfate, filtered and concentrated in vacuo. The crude product was purified by column chroma on silica gel using a mixture of dichloromethane/cyclohexane (80:20 then 100:0; v/v) as eluent to afford the desired product. Yellow-white powder; 306 mg; 81 % yield**.** HPLC (A: Water 30 % , B: CH_3CN 70 %) t_R = 4.84 min Purity > 99 %.^{1}H NMR (400 MHz, DMSO-d_6) δ 10.69 (s, 1H), 10.07 (s, 1H), 8.05 (dd, J = 7.8, 1.7, 1H), 8.01 – 7.92 (m, 2H), 7.68 – 7.61 (m, 2H), 7.58 (dd, J = 7.7, 1.7, 1H), 7.45 – 7.34 (m, 2H), 7.06 – 6.96 (m, 3H), 6.87 (dd, J = 8.2, 1.0, 1H)**.** ^{13}C NMR (100 MHz, DMSO-d_6) δ 160.1, 156.3, 155.1, 152.2, 141.3, 133.4 (2C), 132.7, 131.5, 131.1, 126.9, 123.9 (2C), 119.7, 119.5, 118.1, 117.1, 116.1, 114.2, 113.5, 110.9. The experimental data are in accordance with the literature.[10]

2,2'-(1-(4-bromophenyl)-1H-1,2,4-triazole-3,5-diyl)diphenol-3p. The compound is obtained following the general procedure A from 4-bromophenylhydrazine hydrochloride (187 mg, 0.84 mmol), triethylamine (232 µL, 1.67 mmol) and 2-(2-hydroxyphenyl)-1,3(4H)-benzoxazin-4-one **2** (200 mg, 0.84 mmol). After the addition of aqueous solution of 1M HCl to reach pH = 5, the two layers were separated in a separating funnel and the aqueous layer was extracted three times with ethyl acetate. The combined organic layers were washed with brine, dried over sodium sulfate, filtered and concentrated in vacuo. The crude product was purified by column chromatography on silica gel using a mixture of dichloromethane as eluent to afford0.à the desired product. Brown powder; 224 mg; 66 % yield. HPLC (A: Water 30 % + 0.1 % TFA, B: CH_3CN 70 % + 0.08 % TFA) t_R = 9.25 min Purity > 97%**.** ^{1}H NMR (400 MHz, DMSO-d_6) δ 10.81 (s, 1H), 10.06 (s, 1H), 8.04 (dd, J = 7.8, 1.7, 1H), 7.71 – 7.63 (m, 2H), 7.53 (dd, J = 7.6, 1.7, 1H), 7.48 – 7.32 (m, 4H), 7.06 – 6.93 (m, 3H), 6.87 (dd, J = 8.3, 1.0, 1H). ^{13}C NMR (100 MHz, DMSO-d6) δ 159.7, 156.3, 155.2, 151.9, 137.2, 132.5, 132.1 (2C), 131.4, 131.1, 126.7, 125.6 (2C), 121.5,

119.7, 119.4, 117.0, 116.1, 114.3, 113.7. HRMS (ESI) m/z: Calcd for $C_{20}H_{15}BrN_3O_2$ $[M+H]^+$ 408.0342. Found 408.0341.

2,2'-(1-(4-methoxyphenyl)-1H-1,2,4-triazole-3,5-diyl)diphenol 3q: The compound is obtained following the general procedure A from 4-methoxyphenylhydrazine hydrochloride (146 mg, 0.84 mmol), triethylamine (232 µL, 1.67 mmol) and 2-(2-hydroxyphenyl)-1,3(4H)-benzoxazin-4-one **2** (200 mg, 0.84 mmol). After the addition of aqueous solution of 1M HCl to reach pH = 3, the two layers were separated in a separating funnel and the aqueous layer was extracted three times with ethyl acetate. The combined organic layers were washed with brine, dried over sodium sulfate, filtered and concentrated in vacuo. The resulting solid was dried under vacuum at 50 °C for 4h to give the desired product. Brown powder; 284 mg; 95 % yield. HPLC (A: Water 30 %, B: CH_3CN 70 %) t_R = 6.17 min Purity > 99%. ^1H NMR (400 MHz, DMSO-d_6) δ 10.93 (s, 1H), 10.07 (s, 1H), 8.03 (dd, J = 7.8, 1.7 Hz, 1H), 7.43 (dd, J = 7.7, 1.7 Hz, 1H), 7.40-7.31 (m, 4H), 7.04-6.96 (m, 4H), 6.95-6.85 (m, 2H), 3.77 (s, 3H). ^{13}C NMR (100 MHz, DMSO-d6) δ 159.3, 159.2, 156.3, 155.5, 151.6, 132.2, 131.2, 131.0, 130.7, 126.5, 125.5 (2C), 119.6, 119.1, 117.0, 116.1, 114.5, 114.2 (2C), 113.8, 55.4. The experimental data are in accordance with the literature.[10]

2,2'-(1-(4-hydroxyphenyl)-1H-1,2,4-triazole-3,5-diyl)diphenol 3r: Under argon atmosphere, 2,2'(1-(4-methoxyphenyl)-1H-1,2,4-triazole-3,5-diyl)diphenol **3q** (102 mg, 0.28 mmol) and pyridinium chloride (505 mg, 4.37 mmol) were mixed in a sealed tube. The reaction mixture was heated to 225 °C for 2h and then diluted by ethyl acetate and water. The mixture was extracted three times with ethyl acetate, washed with brine, dried over anhydrous sodium sulfate, filtered and concentrated in vacuo. The crude product was purified by column chromatography on silica gel by using a mixture of dichloromethane/cyclohexane (5:5 v/v) then dichloromethane/methanol (99:1 v/v) as eluent. The resulting solid was dried under vacuum at 60°C during 8h to provide the desired product. Beige powder; 40 mg; 41 %. HPLC (A: Water 30 %, B: CH_3CN 70 %) t_R = 3.95 min Purity > 97 %. ^1H NMR (400 MHz, DMSO-d_6) δ 10.95 (s, 1H), 9.99 (s, 2H), 8.01 (dd, J = 7.8, 1.7, 1H), 7.47 – 7.29 (m, 3H), 7.29 – 7.15 (m, 2H), 7.07 – 6.93 (m, 2H), 6.96 – 6.84 (m, 2H), 6.82 – 6.71 (m, 2H). ^{13}C NMR (100 MHz, DMSO-d6) δ 159.1, 157.7, 156.3, 155.5, 151.5, 132.0, 131.1, 130., 129.2, 126.5, 125.7 (2C), 119.6, 119.0,

116.9, 116.1, 115.5 (2C), 114.5, 113.9. HRMS (ESI) m/z: Calcd for $C_{20}H_{16}N_3O_3$ $[M+H]^+$ 346.1186. Found 346.1187.

4-(3,5-bis(2-hydroxyphenyl)-1H-1,2,4-triazol-1-yl)benzenesulfonamide-3s. The compound is obtained following the general procedure A from 4-hydrazinylbenzenesulfonamide hydrochloride (187 mg, 0.84 mmol) and triethylamine (232 µL, 1.67 mmol) and 2-(2-hydroxyphenyl)-1,3(4H)-benzoxazin-4-one **2** (200 mg, 0.84 mmol. After completion of the reaction, an aqueous solution of saturated aqueous sodium bicarbonate was added to the mixture to reach pH = 7. The two layers were separated in a separating funnel and the aqueous layer was extracted three times with EtOAc. The combined organic layers were washed with brine, dried over sodium sulfate, filtered and concentrated in vacuo to afford the desired product. White product; 290 mg; 85 % yield. HPLC (A: Water 30 % , B: CH_3CN 70 %) t_R = 2.69 min Purity > 99 %.^{1}H NMR (300 MHz, DMSO-d_6) δ 10.76 (s, 1H), 10.11 (s, 1H), 8.05 (dd, J = 7.8, 1.7, 1H), 7.92 – 7.84 (m, 2H), 7.68 – 7.60 (m, 2H), 7.56 (dd, J = 7.6, 1.7, 1H), 7.50 (s, 2H), 7.39 (m, 2H), 7.07 – 6.93 (m, 3H), 6.88 (dd, J = 8.3, 1.0, 1H). Experimental data match with the data reported in the literature.[11]

4-(3,5-bis(2-hydroxyphenyl)-1H-1,2,4-triazol-1-yl)benzenesulfonic acid 3t: The compound is obtained following the general procedure A from 4-hydrazinobenzenesulfonic acid (157 mg, 0.84 mmol) and triethylamine (127 µL, 0.92 mmol) and 2-(2-hydroxyphenyl)-1,3(4H)-benzoxazin-4-one **2** (200 mg, 0.84 mmol). An aqueous solution of 6M HCl was added to the mixture. The resulting solid was filtered, washed with water and diethyl ether and dried under vacuum at 60 °C for 12h to give the desired product. White powder; 231 mg; 68%. HPLC (A: Water 40 % + 0.1 % TFA, B: CH_3CN 60 % + 0.08 % TFA) t_R = 5.90 min Purity > 98 %. ^{1}H NMR (400 MHz, DMSO-d_6) δ 10.10 (s, 1H), 8.04 (dd, J = 7.8, 1.7, 1H), 7.67 – 7.59 (m, 2H), 7.51 (dd, J = 7.7, 1.7, 1H), 7.42 – 7.30 (m, 4H), 6.99 (m, 3H), 6.86 (d, J = 8.2, 1H). Experimental data match with the data reported in the literature.[1]

4-(3,5-bis(2-hydroxyphenyl)-1H-1,2,4-triazol-1-yl)benzamide 3u: 4-(3,5-bis(2-hydroxyphenyl)-1H-1,2,4-triazol-1-yl)benzonitrile **3o** (136 mg, 0.38 mmol) and potassium hydroxide (104 mg, 1.85 mmol) were dissolved in tert-butanol (2.6 mL). The reaction mixture was heated under reflux for 5 h. A solution of brine was added to the mixture followed by acetic acid to reach pH = 5. The two layers were separated in a separating funnel. The aqueous layers were extracted three times with ethyl acetate, dried over anhydrous sodium sulfate,

filtered and concentrated in vacuo to furnish the desired product. White powder; 123 mg; 86 % yield. HPLC (A: Water 30 %, B: CH_3CN 70 %) t_R = 2.42 min Purity > 99 %. ^{1}H NMR (400 MHz, DMSO-d_6) δ 10.82 (s, 1H), 10.06 (s, 1H), 8.05 (dd, J = 7.8, 1.8, 2H), 7.95 – 7.88 (m, 2H), 7.56 – 7.46 (m, 4H), 7.38 (m, 2H), 7.05 – 6.94 (m, 3H), 6.87 (dd, J = 8.3, 1.0, 1H). ^{13}C NMR (100 MHz, DMSO-d_6) δ 166.7, 159.7, 156.3, 155.2, 151.9, 139.9, 134.0, 132.5, 131.4, 131.1, 128.4 (2C), 126.7, 123.1 (2C), 119.6, 119.3, 117.1, 116.1, 114.4, 113.6. The experimental data are in accordance with the literature.[12]

2,2'-(1-(4-(2H-tetrazol-5-yl)phenyl)-1H-1,2,4-triazole-3,5-diyl)diphenol 3v: 4-(3,5-bis(2-hydroxyphenyl)-1H-1,2,4-triazol-1-yl)benzonitrile **3o** (100 mg, 0.28 mmol) was dissolved in anhydrous dimethylformamide (5 mL). Sodium azide (46 mg, 7.05 mmol) and ammonium chloride (38 mg, 7.05 mmol) were added to the reaction mixture. The reaction mixture was heated at 120 °C for 24 h under stirring. After 24 h, a TLC follow-up was carried out showing remaining reagent. Therefore, additional sodium azide (46 mg, 7.05 mmol) and ammonium chloride (38 mg, 7.05 mmol) were added to the mixture. The reaction mixture was heated at 120 °C for 24 h. The mixture was diluted by EtOAc then a solution of brine was added to the mixture and the two layers separated in a separating funnel. The aqueous layer was extracted three times with ethyl acetate, dried over anhydrous sodium sulfate, filtered and concentrated in vacuo. The crude product was purified by column chromatography in silica gel using a mixture of dichloromethane/methanol (100:0 to 90:10, v/v) as eluent. The resulting solid was dried under vacuum at 50°C during 12 h to provide the desired product. White powder; 58 mg; 52 % yield. HPLC (A: Water 40 % + 0.1% TFA, B: CH_3CN 60 % + 0.08% TFA) t_R = 7.06 min Purity > 99 %. ^{1}H NMR (400 MHz, DMSO-d_6) δ 10.89 (s, 1H), 10.24 (s, 1H), 8.17 – 8.00 (m, 3), 7.57 – 7.49 (m, 3H), 7.37 (m, 2H), 7.05 – 6.89 (m, 4H). ^{13}C NMR (100 MHz, DMSO-d6) δ 159.6, 156.3, 155.4, 151.8, 137.9, 132.4, 131.3, 131.0, 129.0, 127.0 (2C), 126.7, 124.0 (2C), 119.7, 119.3, 117.0, 116.2, 114.5, 114.5, 113.8. HRMS (ESI) m/z: Calcd for $C_{21}H_{16}N_7O_2$ $[M+H]^+$ 398.1360. Found 398.1361.

Synthesis of (S)-methyl 2-(4-(3,5-bis(2-hydroxyphenyl)-1H-1,2,4-triazol-1-yl)benzamido)-5-(3-tosylguanidino)pentanoate-3w

(S)-methyl 2-amino-5-(3-tosylguanidino)pentanoate: (S)-2-((tert-butoxycarbonyl)amino)-5-(3-tosylguanidino)pentanoic acid (200 mg, 0.47 mmol) was dissolved in MeOH (3.5 mL). Acetyl chloride (1.66 mL, 0.023 mol) was added dropwise at 0 °C. The reaction was stirred overnight

at r.t. A saturated aqueous $NaHCO_3$ solution was added until reaching pH 9. The mixture was extracted with ethyl acetate. The organic layer was then washed two times with water, dried over Na_2SO_4 and concentrated under reduced pressure to afford the product. Pure colorless oil; 107 mg, 67% yield. ^1H NMR (400 MHz, DMSO-d_6) δ 7.63 (d, J = 8.1 Hz, 2H), 7.28 (d, J = 8.1 Hz, 2H), 7.05 (bs, 1H), 6.79 (bs, 1H), 6.57 (bs, 1H), 3.61 (s, 3H), 3.26 (t, J = 6.0 Hz, 1H), 3.03 (s, 2H), 2.34 (s, 3H), 1.90 – 1.73 (m, 2H), 1.59 – 1.29 (m, 4H). ^{13}C NMR (100 MHz, DMSO-d6) 176.0, 156.6, 141.0, 129.0, 125.5, 53.6, 51.4, 40.5, 20.9. HRMS (ESI) m/z: Calcd. for $C_{14}H_{23}N_4O_4S$ $[M+H]^+$ 343.1435. Found 343.1434.

(S)-methyl 2-(4-(3,5-bis(2-hydroxyphenyl)-1H-1,2,4-triazol-1-yl)benzamido)-5-(3-tosylguanidino)pentanoate 3w: The product was prepared according to the general procedure B from **DFX-3a** (93 mg, 0.25 mmol) and (S)-methyl 2-amino-5-(3-tosylguanidino)pentanoate (102 mg, 0.30 mmol). The crude product was purified via column chromatography on silica gel (0-6% MeOH in DCM) to give the desired compound. Brown powder; 93 mg; 54% yield. ^1H NMR (400 MHz, DMSO-d_6) δ 10.82 (s, 1H), 10.05 (s, 1H), 8.83 (d, J = 7.4 Hz, 1H), 8.06 (dd, J = 7.8, 1.6 Hz, 1H), 7.98 – 7.91 (m, 2H), 7.66 – 7.60 (m, 2H), 7.59 – 7.51 (m, 3H), 7.42 – 7.34 (m, 2H), 7.25 (d, J = 8.0 Hz, 2H), 7.05 – 6.95 (m, 3H), 6.87 (d, J = 7.9 Hz, 1H), 6.81 (bs, 1H), 6.58 (bs, 1H), 4.44 – 4.34 (m, 1H), 3.64 (s, 3H), 3.08 (m, 2H), 2.32 (s, 3H), 1.86 – 1.65 (m, 2H), 1.60 – 1.38 (m, 2H). ^{13}C NMR (100 MHz, DMSO-d6) δ 172.4, 165.5, 159.8, 156.6, 156.3, 155.2, 152.0, 141.5, 140.6, 133.4, 132.5, 131.4, 131.1, 129.0, 128.5, 126.8, 125.5, 123.2, 119.7, 119.4, 117.0, 116.1, 114.9, 114.5, 113.7, 52.5, 51.9, 27.7, 20.8. HRMS (ESI) m/z: Calcd for $C_{35}H_{36}N_7O_7S$ $[M+H]^+$ 698.2391. Found 698.2396.

Sodium benzoyl(2-fluorobenzoyl)amide 5: To a stirred solution of 2-fluoronicotinamide **4** (500 mg, 3.59 mmol) in anhydrous THF (0.07 M), at 0° C, under argon atmosphere, was added NaH (60% in oil dispersion, 316 mg, 7.91 mmol), and the reaction mixture was stirred 1 h at 0° C. Then, the reaction mixture was cool to -78° C, and benzoyl chloride (418 µL, 3.59 mmol) was added dropwise. The reaction was slowly warmed up to r.t and stirred for 16 h. The reaction mixture was filtered on filter paper and concentrated under reduced pressure. The resulting residue was triturated with Et_2O to provide, after filtration on fritted glass 4. The resulting solid was dried under vacuum at 50°C during 2 h to provide the desired product. White powder; 645 mg; 68% yield. ^1H NMR (300 MHz, DMSO-d_6) δ 8.17 – 8.05 (m, 2H), 7.81 (dd, J = 7.6, 1.9, 1H), 7.36 (m, 4H), 7.21 – 7.04 (m, 2H). ^{13}C NMR (75 MHz, DMSO-d6) δ 173.8,

172.8, 160.1, 140.5, 131.0, 130.5, 130.1, 129.8, 128.7 (2C), 127.4 (2C), 123.3, 115.8. ^{19}F NMR (282 MHz, DMSO-d6) δ -113.88. Experimental data match with the data reported in the literature.[13]

Sodium 4-methoxybenzoyl(2-fluorobenzoyl)amide 6: To a stirred solution of 2-fluoronicotinamide **4** (500 mg, 3.59 mmol) in anhydrous THF (0.07 M), at 0° C, under argon atmosphere, was added NaH (60% in oil dispersion, 316 mg, 7.91 mmol), and the reaction mixture was stirred 1 h at 0°C. Then, the reaction mixture was cool to -78° C, and 4-methoxybenzoyl chloride (613 mg, 3.59 mmol) was added portionwise. The reaction was slowly warmed up to r.t. and stirred for 16 h. The reaction mixture was filtered on filter paper and concentrated under reduced pressure. The resulting residue was triturated with Et_2O to provide, after filtration on fritted glass 4. The resulting solid was dried under vacuum at 50°C during 2h to provide the desired product. White powder; 646 mg; 61% yield. ^{1}H NMR (300 MHz, DMSO-d_6) δ 8.13 (d, J = 6.6, 2H), 7.90 (dt, J = 7.8, 2.1 Hz, 1H), 7.36 (m, 1H), 7.15 (m, 1H), 6.89 (d, J = 6.6, 2H). 13C NMR (75 MHz, DMSO-d6) δ 174.3, 172.7, 162.1, 161.0, 158.7, 133.0, 131.3, 130.7(2C), 130.3, 123.3, 116.1, 115.8, 112.7(2C), 55.1**.** HRMS (ESI): m/z $[M+H]^+$ calcd for $C_{15}H_{12}FNNaO_3$: 296.0702; found: 296.0693.

2-phenyl-4H-benzo[e][1,3]oxazin-4-one 7: In a microwave vial, under argon atmosphere, sodium benzoyl(2-fluorobenzoyl)amide **5** (250 mg, 0,94 mmol) was dissolved in anhydrous dimethyl sulfoxide (19 mL). The vial was sealed and the reaction mixture was submitted to microwave irradiation while stirring for 30 min at 180 °C (in very high absorption mode). The mixture was then diluted with ethyl acetate and washed three times with brine. The organic layer was then dried with sodium sulfate, filtered, and concentrated in vacuo to give the desired product. Brown powder; 178 mg; 85 % yield. ^{1}H NMR (400 MHz, Chloroform-d) δ 8.46 – 8.37 (m, 2H), 8.26 – 8.18 (m, 1H), 7.77 (m, 1H), 7.66 (m, 1H), 7.52 (m, 4H). Experimental data match with the data reported in the literature.[13]

2-(4-methoxyphenyl-4H-benzo[e][1,3]oxazin-4-one 8: In a microwave vial, under argon atmosphere, sodium benzoyl(2-fluorobenzoyl)amide **6** (180 mg, 0,61 mmol) was dissolved in anhydrous dimethyl sulfoxide (20 mL). The vial was sealed and the reaction mixture was submitted to microwave irradiation while stirring for 30 min at 180 °C (in very high absorption mode). The mixture was then diluted with ethyl acetate and washed three times with brine. The organic layer was then dried with sodium sulfate, filtered, and concentrated in vacuo to

give the desired product. Brown powder; 110 mg; 71 % yield.^{1}H NMR (300 MHz, Chloroform-d) δ 8.29 (d, J= 9, 2H), 8.02 (dd, J = 7.8, 1.8 1H), 7.89 (dt, J = 7.2, 1.8, 1H), 7.71 (d, J = 8.7, 1H), 7.55 (dt, J = 7.2, 0.9, 1H), 7.18 (d, J= 9, 2H), 3.89 (s, 3H). ^{13}C NMR (75 MHz, DMSO-d6) δ 165.7, 164.1, 163.0, 154.4, 135.5, 131.2 (2C), 126.8, 126.7, 121.6, 117.7, 117.3, 114.6 (2C), 55.7. HRMS (ESI): m/z Calcd for $C_{15}H_{11}NNaO_3$ [M+Na]$^+$ 276.0635; found: 276.0631.

4-(3-(2-hydroxyphenyl)-5-phenyl-1H-1,2,4-triazol-1-yl)benzoic acid 9: The compound is obtained following the general procedure A from 4-hydrazinobenzoic acid (68 mg, 0.45 mmol), triethylamine (68 µL, 0.49 mmol) and 2-phenyl-4H-benzo[e][1,3]oxazin-4-one **7** (100 mg, 0.45 mmol). After addition of 1 M HCl solution to the mixture till pH = 3, the resulting solid was filtered and washed with water and dried under vacuum at 50 °C for 5h to give the desired product. The crude product was purified by column chromatography on silica gel using a mixture of ethyl acetate/cyclohexane (20:80 then 50:50 then 100:0, v/v) as eluent. The obtained solid was dried under vacuum at 60°C during 2h to provide the desired compound. Yellow-green powder; 53 mg; 38% yield. HPLC (A: Water 40 % + 0.1 % TFA, B: CH_3CN 60 % + 0.08 % TFA) t_R = 6.12 min Purity > 97 %. ^{1}H NMR (400 MHz, DMSO-d_6) δ 13.13 (s, 1H), 9.97 (s, 1H), 8.15 – 8.09 (m, 2H), 7.99 – 7.92 (m, 2H), 7.51 (m, 6H), 7.37 (m, 1H), 6.96 (m, 1H), 6.85 (dd, J = 8.2, 1.0, 1H). ^{13}C NMR (101 MHz, DMSO-d_6) δ 166.5, 160.9, 155.1, 153.2, 141.6, 132.1, 130.9, 130.3, 130.2 (2C), 129.6, 128.9 (2C), 126.0 (2C), 123.0 (2C), 119.3, 116.0, 115.4. HRMS (ESI) m/z: Calcd for $C_{21}H_{16}N_3O_3$ [M+H]$^+$ 358.1186. Found: 358.1186.

4-(5-(2-hydroxyphenyl)-3-(4-methoxyphenyl)-1H-1,2,4-triazol-1-yl)benzoic acid 10: The compound is obtained following the general procedure A from 4-hydrazino-benzoic acid (60 mg, 0.38 mmol) and compound **8** (90 mg, 0.35 mmol). The rection mixture was stirred for 30 min. and then concentrated. The residue triturated with water. The resulting solid was purified by column chromatography on silica gel using a mixture of dichloromethane/methanol (92:8, v/v) as eluent. The obtained solid was dried under vacuum at 60°C during 2 h to provide the desired compound. Light brown powder; 106 mg; 77% yield. HPLC (A: Water 30 % + 0.1 % TFA, B: CH_3CN 70 % + 0.08 % TFA) t_R = 3.29 min Purity > 94%**.** ^{1}H NMR (300 MHz, DMSO-d_6) δ 8.05 (d, J = 9, 2H), 7.96 (d, J = 9, 2H), 7.47 (m, 3H), 7.35 (dt, J = 7.2, 1.8, 1H), 7.06 (d, J = 9, 2H), 6.93 (dt, J = 7.5, 0.9, 1H), 6.87 (d, J = 78.1, 1H), 3.81 (s, 3H). ^{13}C NMR (75 MHz, DMSO-d_6) δ 167.5, 160.7, 160.4, 155.4, 153.0, 141.1, 132.0, 130.8, 130.2, 127.6, 122.9, 122.8, 119.2, 116.1, 115.6, 114.3, 55.3. HRMS (ESI): m/z Calcd for $C_{22}H_{17}N_3O_4$ [M-H]$^-$ 386.1158; found: 386.1146.

2-(2-hydroxyphenyl)-7-methoxy-4H-benzo[e][1,3]oxazin-4-one 12: In a round-bottomed flask, salicylamide **1** (410 mg, 3.0 mmol) and 2,4-dimethoxybenzoyl chloride **11** (600 mg, 3.0 mmol) were suspended in xylene (10 mL). The reaction was heated to 130 °C for 4 h, leading to complete dissolution of the starting materials and change in the color of the solution to yellow. The reaction mixture was cooled to room temperature and xylene evaporated under reduced pressure. The obtained crude product was triturated with methanol three times to give the expected product after drying under vacuum at 50 °C for 3 h. Beige powder; 170 mg; 21 % yield. ^{1}H NMR (300 MHz, Chloroform-d) δ 13.06 (s, 1H), 8.19 (dd, J = 7.8, 1.7, 1H), 8.00 (d, J = 8.8, 1H), 7.76 (m, 1H), 7.56 – 7.40 (m, 2H), 6.65 – 6.45 (m, 2H), 3.87 (s, 3H). Experimental data match with the data reported in the literature.[14]

4-(3-(2-hydroxy-4-methoxyphenyl)-5-(2-hydroxyphenyl)-1H-1,2,4-triazol-1-yl)benzoic acid 13: The compound is obtained following the general procedure A from 4-hydrazinobenzoic acid (92 mg, 0.60 mmol), triethylamine (93 µL, 0.66 mmol) and 2-(2-hydroxyphenyl)-7-methoxy-4H-benzo[e][1,3]oxazin-4-one **12** (162 mg, 0.6 mmol). After addition of aqueous solution of 1M HCl to the mixture till pH = 3, the resulting solid was filtered and washed with water. The crude product was purified by column chromatography on silica gel by using a mixture of diethyl ether/cyclohexane as eluant (50/50, 70/30 then 100/0; v/v) as eluent and dried under vacuum at 60°C during 4 h to provide the desired product. Pale yellow solid; 200 mg; 60 % yield. HPLC (A: Water 40 % + 0.1 % TFA, B: CH_3CN 60 % + 0.08 % TFA) t_R = 6.89 min Purity > 98 %. ^{1}H NMR (400 MHz, DMSO-d_6) δ 13.17 (s, 1H), 10.90 (s, 1H), 10.03 (s, 1H), 8.03 – 7.91 (m, 3H), 7.54 (m, 3H), 7.43 – 7.35 (m, 1H), 6.98 (m, 1H), 6.87 (dd, J = 8.3, 1.0 Hz, 1H), 6.64 – 6.55 (m, 2H), 3.80 (s, 3H). ^{13}C NMR (101 MHz, DMSO-d_6) δ 166.4, 161.9, 159.9, 157.9, 155.1, 151.7, 141.2, 132.5, 131.0, 130.4, 130.2 (2C), 127.7, 123.2 (2C), 119.4, 116.1, 114.5, 106.9, 106.6, 101.4, 55.3. HRMS (ESI) m/z: Calcd for $C_{22}H_{18}N_3O_5$ $[M+H]^+$ 404.1241. Found 404.1237.

4-(3-(2,4-dihydroxyphenyl)-5-(2-hydroxyphenyl)-1H-1,2,4-triazol-1-yl)benzoic acid 14: Under argon atmosphere, 4-(3-(2-hydroxy-4-methoxyphenyl)-5-(2-hydroxyphenyl)-1H-1,2,4-triazol-1-yl)benzoic acid **13** (91 mg, 0.23 mmol) and pyridinium chloride (430 mg, 3.72 mmol) were mixed in a sealed tube. The reaction mixture was heated to 225 °C for 2 h and then diluted by ethyl acetate and water. The mixture was extracted three times with ethyl acetate, dried over anhydrous sodium sulfate, filtered and concentrated in vacuo. The crude product was purified by column chromatography on silica gel by using a mixture of

dichloromethane/methanol (99:1, 95:5 to 90:10; v/v) as eluent. The resulting solid was dried under vacuum at 60°C during 8h to provide the desired product. White powder; 51 mg; 58% yield. HPLC (A: Water 40 % + 0.1 % TFA, B: CH_3CN 60 % + 0.08 % TFA) t_R = 2.96 min Purity > 90%. ^1H NMR (400 MHz, DMSO-d6) δ 13.27 (s, 1H), 10.76 (s, 1H), 10.05 (s, 2H), 8.01 – 7.93 (m, 2H), 7.84 (d, J = 8.6 Hz, 1H), 7.55 – 7.48 (m, 3H), 7.38 (m, 1H), 6.97 (m, 1H), 6.87 (dd, J = 8.3, 1.0 Hz, 1H), 6.44 (dd, J = 8.6, 2.4 Hz, 1H), 6.38 (d, J = 2.4 Hz, 1H). ^{13}C NMR (100 MHz, DMSO-d6) δ 166.5, 160.4, 160.2, 157.9, 155.2, 151.6, 141.1, 132.4, 131.0, 130.7, 130.2 (2C), 127.8, 123.1 (2C), 119.4, 116.1, 114.6, 108.1, 105.3, 102.9. HRMS (ESI) m/z: Calcd for $C_{21}H_{16}N_3O_5$ $[M+H]^+$ 390.1084. Found: 390.1079.

4-[3-(2,4-dimethoxyphenyl)-5-(3-hydroxy-4-pyridyl)-1,2,4-triazol-1-yl]benzoic acid 20: In a vial under argon atmosphere, to a solution of 2-(2,4-dimethoxyphenyl)pyrido[4,3-e][1,3]oxazin-4-one **17** (100 mg, 0.35 mmol) in EtOH (10 mL) were added 4-hydrazinobenzoic acid (54 mg , 0.35 mmol). The vial was sealed and the resulting mixture was stirred for 30 min at 110 °C. The mixture was concentrated in vacuo. The resulting residue was then triturated in diethyl ether and filtered to furnish the desired compound. White powder; 103 mg, 71 % yield. ^1H NMR (300 MHz, DMSO-d_6) δ 8.24 (s, 1H), 8.19 (d, J = 4.9 Hz, 1H), 8.01 (d, J = 8.6 Hz, 2H), 7.82 (d, J = 8.5 Hz, 1H), 7.52 (d, J = 8.6 Hz, 2H), 7.48 (d, J = 4.9 Hz, 1H), 6.72 (d, J = 2.3 Hz, 1H), 6.65 (dd, J = 8.6, 2.4 Hz, 1H), 3.85 (s, 3H), 3.84 (s, 3H). ^{13}C NMR (75 MHz, DMSO-d_6) δ 166.5, 161.6, 159.9, 158.6, 150.9, 149.7, 141.3, 140.4, 139.0, 131.4, 130.5, 130.3 (2C), 124.3, 123.3 (2C), 122.0, 111.8, 105.3, 99.1, 55.8, 55.3; IR (film, cm^{-1}) 1689, 1608, 1572, 1291, 1160. HRMS (ESI) m/z: Calcd for $C_{22}H_{19}N_4O_5$ $[M+H]^+$ 419,1350. Found: 419.1340.

4-(3,5-bis(2-methoxyphenyl)-1H-1,2,4-triazol-1-yl)benzoic acid 21: In a sealed tube, under argon atmosphere, 4-(3,5-bis(2-hydroxyphenyl)-1,2,4-triazol-1-yl)benzoic acid **DFX-3a** (200 mg, 0.54 mmol) was dissolved in anhydrous acetonitrile (5 mL). Potassium carbonate (444 mg, 3.21 mmol) and iodomethane (274 mg, 1.93 mmol) were added and the mixture was heated under reflux for 6 h. Water was added to stop the reaction. The suspension was filtered and the solid washed with water to afford a residue which was purified by column chromatography on silica gel using a mixture of ethyl acetate/cyclohexane (20:80; v/v) as eluent. The resulting solid was dried under vacuum at 60°C during 4 h to provide the desired product. White solid; 131 mg; 61% yield. HPLC (A: Water 15 % + 0.1 % TFA, B: CH_3CN 85 % + 0.08 % TFA) t_R = 6.47 min Purity > 95 %.^1H NMR (400 MHz, Chloroform-d) δ 10.84 (s, 1H), 8.18 (dd, J = 7.9, 1.7, 1H), 8.07 – 7.99 (m, 2H), 7.67 (dd, J = 7.6, 1.7, 1H), 7.54 – 7.42 (m, 3H), 7.34 (m, 1H, H_2), 7.14 (m,

1H), 7.06 (dd, J = 8.3, 1.1, 1H), 7.00 – 6.93 (m, 1H), 6.84 (dd, J = 8.4, 0.9, 1H), 3.93 (s, 3H), 3.32 (s, 3H). ^{13}C NMR (100 MHz, Chloroform-d) δ 166.2, 161.4, 157.2, 156.6, 151.6, 142.3, 132.8, 131.4, 130.5 (2C), 129.7, 127.1, 122.9 (2C), 121.4, 119.6, 117.4, 116.9, 114.1, 111.6, 54.9, 52.5. The experimental data are in accordance with literature.[12]

4-(3,5-bis(2-(carboxyoxy)phenyl)-1H-1,2,4-triazol-1-yl)benzoic acid 22: 4-(3,5-bis(2-hydroxyphenyl)-1,2,4-triazol-1-yl)benzoic acid **DFX-3a** (200 mg, 0.54 mmol) was dissolved in acetic anhydride (1.4 mL). Then concentrated H_2SO_4 (67 µL) was added to the mixture. The reaction mixture was heated at 80 °C for 4 h under stirring. The mixture was diluted by EtOAc and water. The two layers were separated in a separating funnel and the aqueous layer was extracted three times with EtOAc. The combined organic layers were washed with brine, dried over anhydrous sodium sulfate, filtered and concentrated in vacuo. The crude product was purified by column chromatography in silica gel using a mixture of dichloromethane/methanol (95.5/0.5 to 90:10, v/v) as eluent. The resulting solid was dried under vacuum at 50°C during 12 h to provide the desired product. White solid; 110 mg, 45% yield. HPLC (A: Water 25 % + 0.1 % TFA, B: CH_3CN 75 % + 0.08 % TFA) t_R = 3.85 min Purity > 98 %**.** ^{1}H NMR (400 MHz, DMSO-d_6) δ 13.25 (s, 1H), 8.24 (dd, J = 7.8, 1.7, 1H), 8.05 – 7.97 (m, 2H), 7.66 – 7.24 (m, 9H), 2.27 (s, 3H), 2.02 (s, 3H). ^{13}C NMR (100 MHz, DMSO-d_6) δ 169.5, 168.4, 166.4, 158.6, 150.7, 148.2, 148.1, 140.6, 132.1, 131.3, 130.9, 130.8, 130.5 (2C), 129.1, 126.4, 126.2, 124.1, 124.0 (2C), 123.4, 122.9, 121.0, 21.1, 20.5. HRMS (ESI) m/z: Calcd for $C_{25}H_{20}N_3O_6$ $[M+H]^+$ 458.1347. Found 458.1341.

2,2'-(1,2,4-oxadiazole-3,5-diyl)diphenol 23: In a sealed tube and under an atmosphere of argon, 2-(2-hydroxyphenyl)-1,3(4H)-benzoxazin-4-one **2** (150 mg, 0.63 mmol)**,** hydroxylamine hydrochloride (44 mg, 0.63 mmol) and sodium acetate (51 mg, 0.63 mmol) were mixed for about 3 hours in absolute EtOH (3.2 mL) at room temperature. After completion reaction, an aqueous solution of saturated sodium chloride was added to the mixture. The two layers were separated in a separating funnel and the aqueous layer was extracted three times with EtOAc. The combined organic layers were dried over sodium sulfate, filtered and concentrated in vacuo. The crude product was purified by column chromatography on silica gel using a mixture of DCM/cyclohexane (50:50 then 90:10, v/v) as eluent to provide the desired product. Beige powder, 81 mg, 51 % yield. HPLC (A: Water 30 % + 0.1 % TFA, B: CH_3CN 70 % + 0.08 % TFA) t_R = 6.26 min Purity > 98%**.** ^{1}H NMR (300 MHz, DMSO-d_6) δ 10.71 (s, 1H), 10.14 (s, 1H), 8.01 (dd,

J = 7.9, 1.7, 1H), 7.91 (dd, J = 7.8, 1.8, 1H), 7.55 (m, 1H), 7.48 – 7.39 (m, 1H), 7.14 (dd, J = 8.4, 1.0, 1H), 7.10 – 6.96 (m, 3H). The experimental data are in accordance with the literature [11].

2.b. Mechanisms of inhibition

Figure S1: Mechanisms of inhibition of hit compounds in absence and presence of 100 µM $FeCl_3$. Dixon plots for KLK1, KLK6 and KLK8 hits

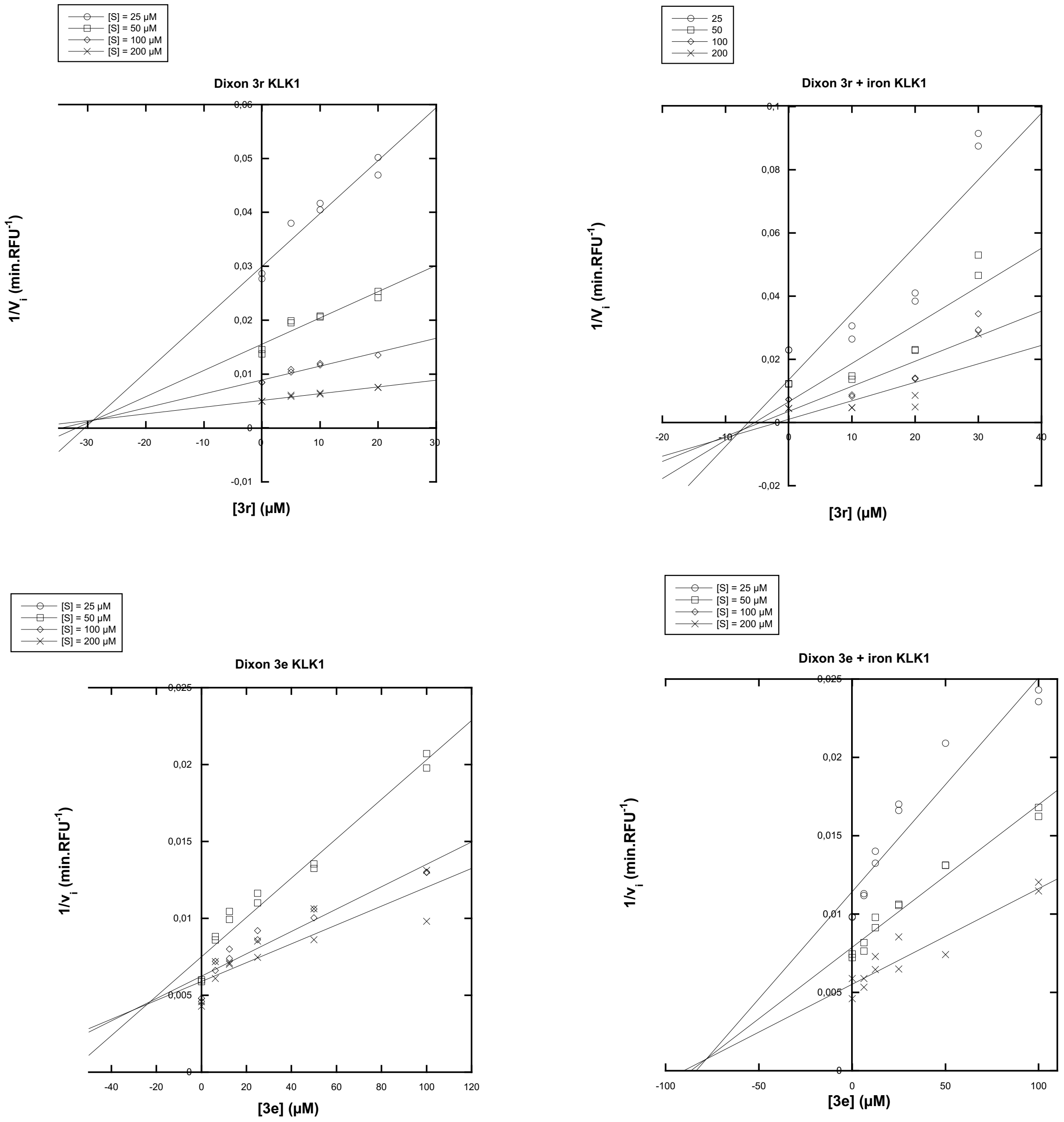

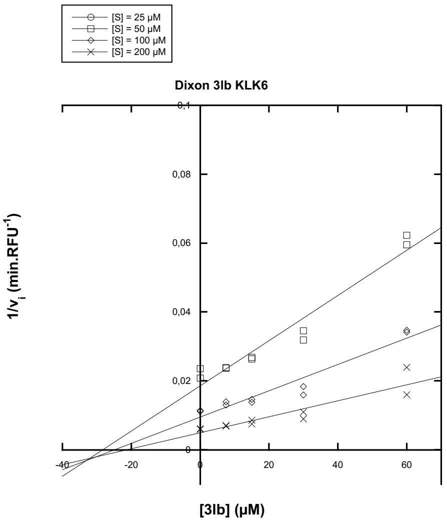
[S] = 25 µM
[S] = 50 µM
[S] = 100 µM
[S] = 200 µM
Dixon 3lb KLK6
1/v_i (min.RFU^-1)
[3lb] (µM)

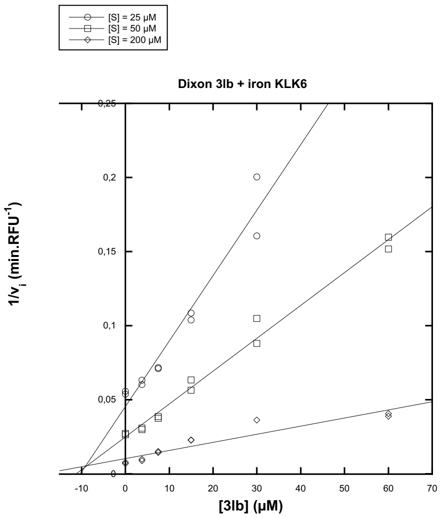
[S] = 25 µM
[S] = 50 µM
[S] = 200 µM
Dixon 3lb + iron KLK6
1/v_i (min.RFU^-1)
[3lb] (µM)

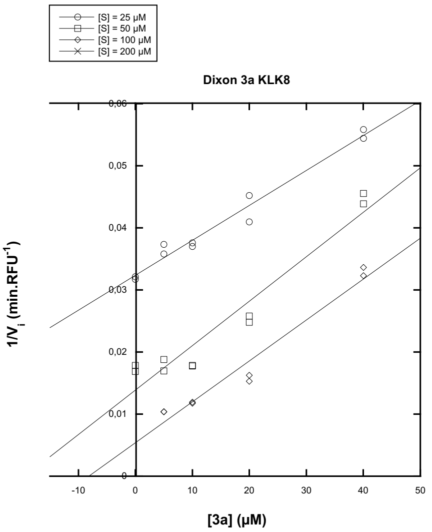
[S] = 25 µM
[S] = 50 µM
[S] = 100 µM
[S] = 200 µM
Dixon 3a KLK8
1/V_i (min.RFU^-1)
[3a] (µM)

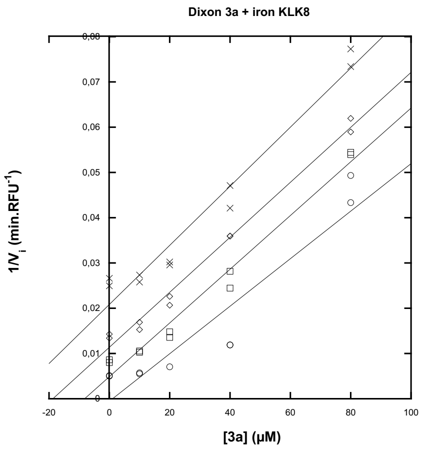
Dixon 3a + iron KLK8
1/V_i (min.RFU^-1)
[3a] (µM)

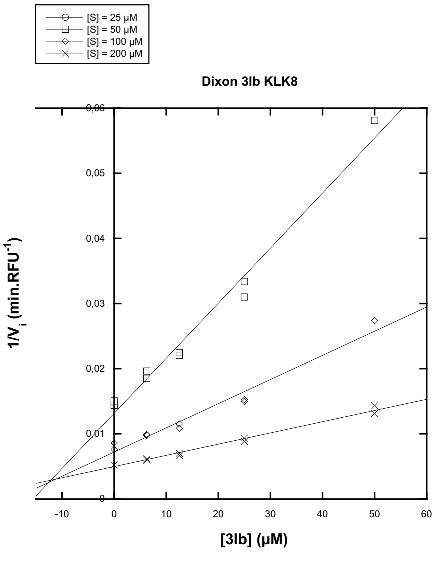
[S] = 25 µM
[S] = 50 µM
[S] = 100 µM
[S] = 200 µM
Dixon 3lb KLK8
1/V_i (min.RFU^-1)
[3lb] (µM)

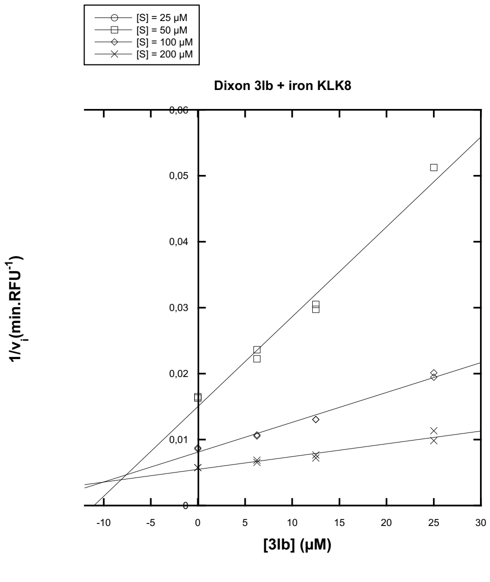
[S] = 25 µM
[S] = 50 µM
[S] = 100 µM
[S] = 200 µM
Dixon 3lb + iron KLK8
1/v_i(min.RFU^-1)
[3lb] (µM)

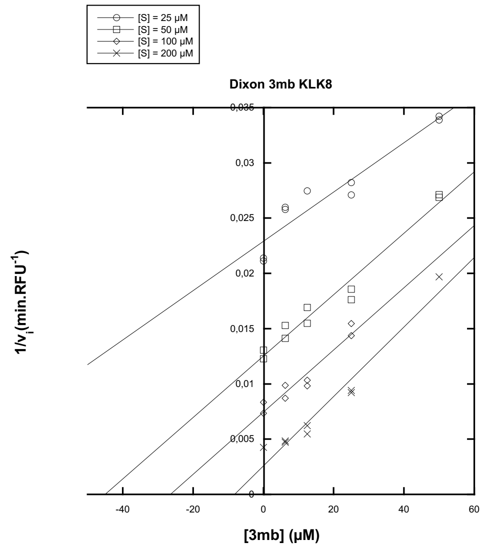

[S] = 25 µM
[S] = 50 µM
[S] = 100 µM
[S] = 200 µM
Dixon 3mb KLK8
1/v_i(min.RFU^-1)
[3mb] (µM)

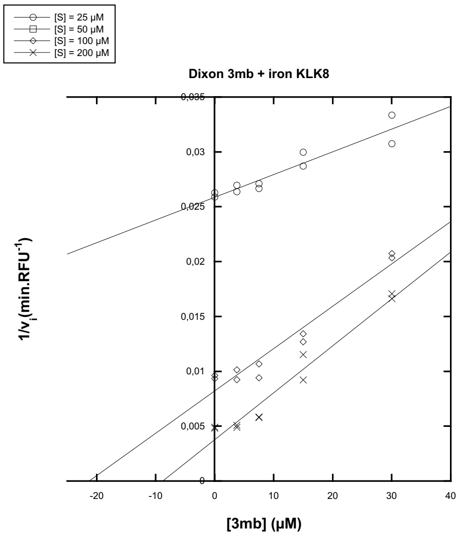

[S] = 25 µM
[S] = 50 µM
[S] = 100 µM
[S] = 200 µM
Dixon 3mb + iron KLK8
1/v_i(min.RFU^-1)
[3mb] (µM)

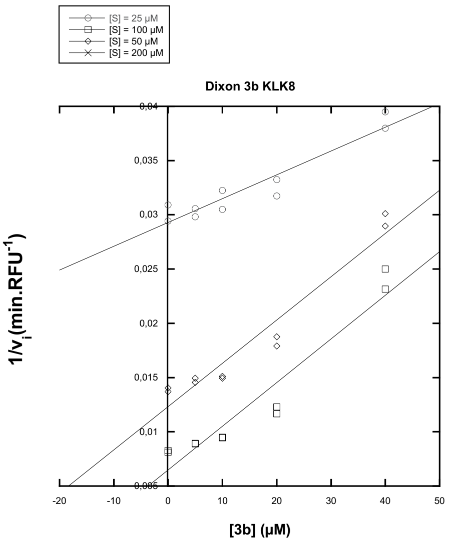

[S] = 25 µM
[S] = 100 µM
[S] = 50 µM
[S] = 200 µM
Dixon 3b KLK8
1/v_i(min.RFU^-1)
[3b] (µM)

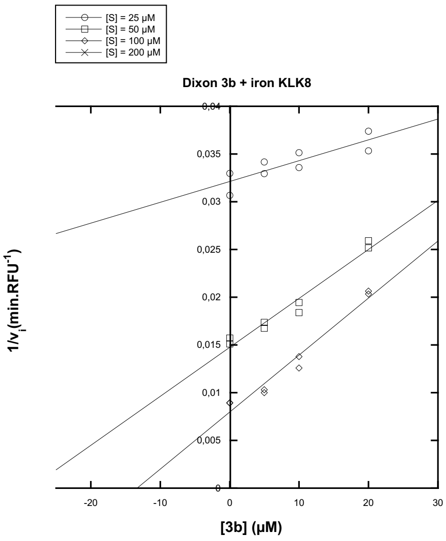

[S] = 25 µM
[S] = 50 µM
[S] = 100 µM
[S] = 200 µM
Dixon 3b + iron KLK8
1/v_i(min.RFU^-1)
[3b] (µM)

2.c. Chelation properties, iron interactions, and selectivity profiles of selected compounds targeting KLK activity.

Figure S2: Stability of the chelated species as monitored by spectrophotometry. UV/VIS spectra of molecules alone using activity buffer at pH 7.4 (in orange at 500 µM) or in the presence of $FeCl_3$ (ratio of 1:1) at different times (t = 0 in red, 30 min in black, 60 min in blue and 240 min in green). The purple trace corresponds to iron only at 500 µM All data are representative of 2 independent experiments performed in duplicate.

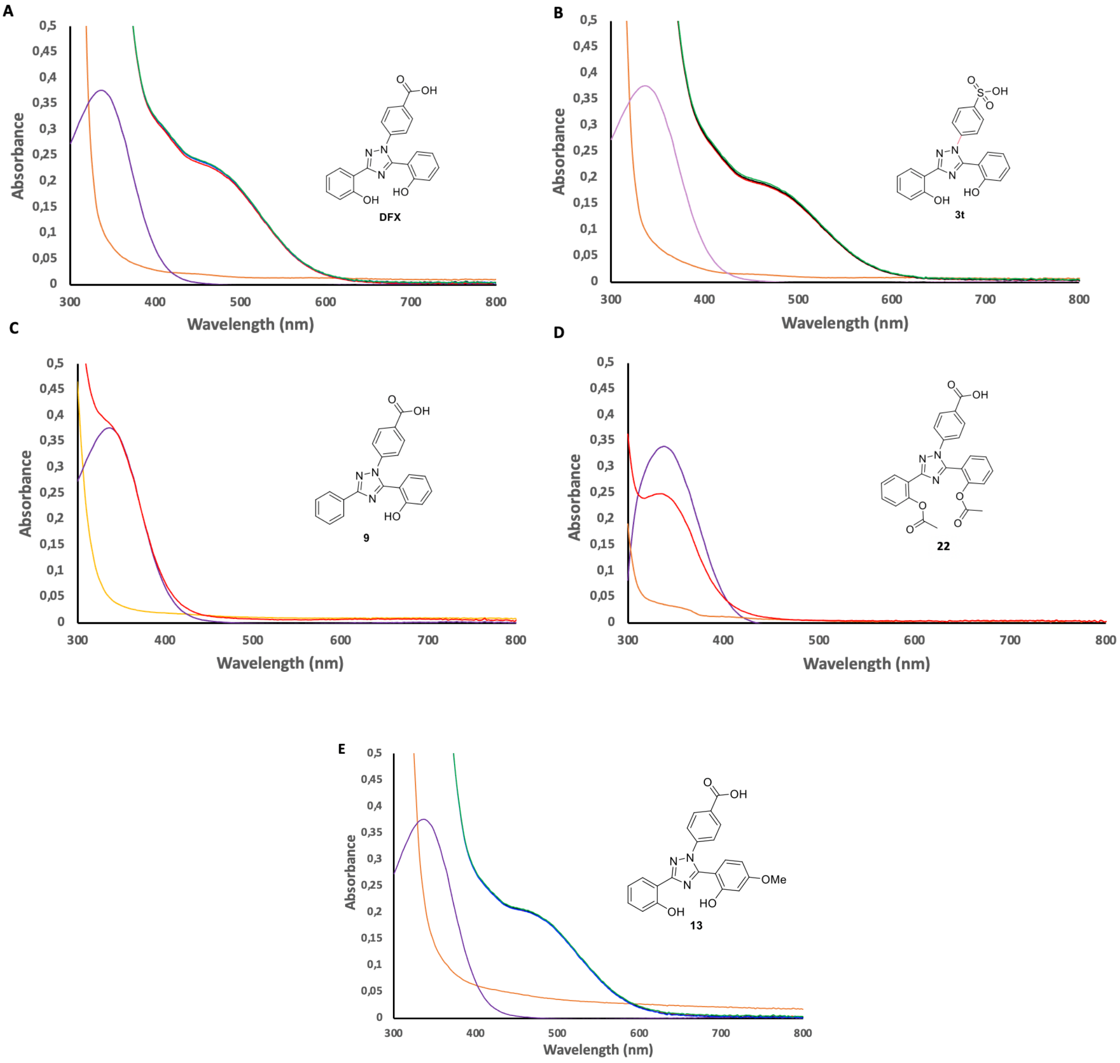

Figure S3 : Trans-chelation test to analyze chelation properties for the newly synthetized compounds in comparison to DFX

A.

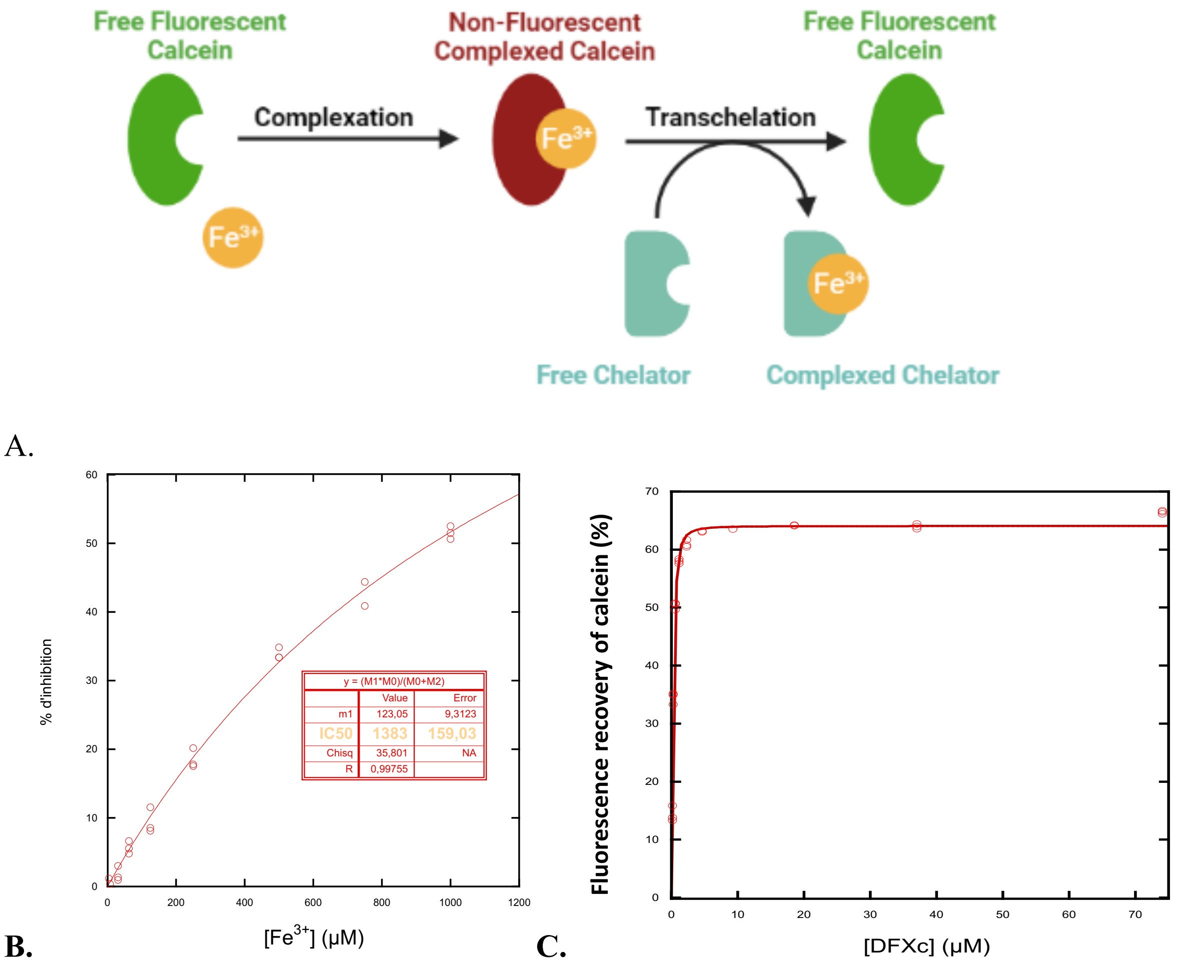

B. C.

A. Schematic representation of the calcein trans-chelation assay. The free chelator here represents analogs from the DFX series. Calcein has fluorescent properties with $\lambda_{excitation}$ = 485 nm and $\lambda_{emission}$ = 520 nm.

B. Example of quantification of the inhibitory potency of [Fe^{3+}] on KLK8, the same procedure was followed for the other KLK's. The IC_{50} of Iron towards KLK's was determined by a range of [Fe^{3+}] from 2 µM to 2 mM. Enzymatic kinetics were monitored using Boc-VPR-AMC substrate (100 µM) in 50 mM Tris, 1 M Citrate, 0.05% Brij35, pH = 7.4 buffer for 30 min at 37°C.

C. Curve showing restoration of calcein fluorescence by DFX acting as competitor. Different compound concentrations were tested (range 0.72 nM to 74 µM) against a fixed concentration of calcein in the wells (100 nM). The inhibitors were brought into contact with calcein for 4 h at room temperature in a buffer consisting of 20 mM HEPES, 150 mM NaCl at pH = 7.3. Fluorescence measurements were made in EndPoint-type readout at 37°C.

Figure S4 : Effect of [Fe^{3+}] on KLK's activity : A. KLK1; B. KLK6; C. KLK8. Curve fits for Pseudo-IC_{50} are shown as insert.

A.

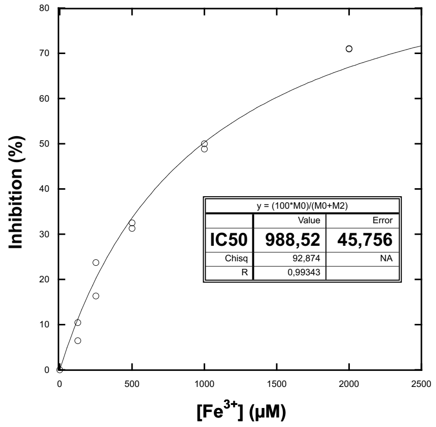

B.

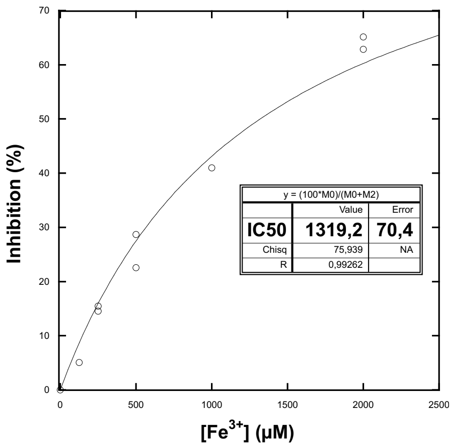

C.

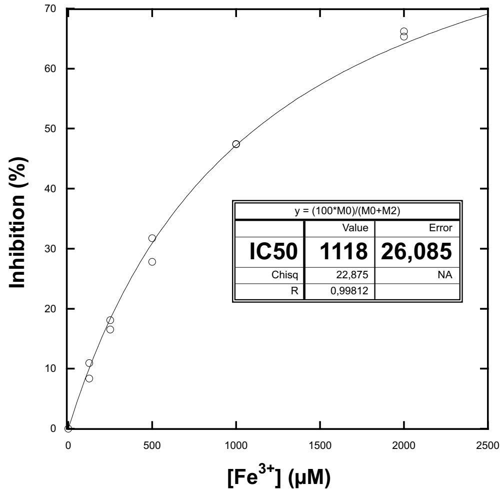

Figure S5: Effect of [Fe^{3+}] on the inhibition potential of DFX-based derivatives. A. KLK1; B. KLK6; C. KLK8.

A. KLK1

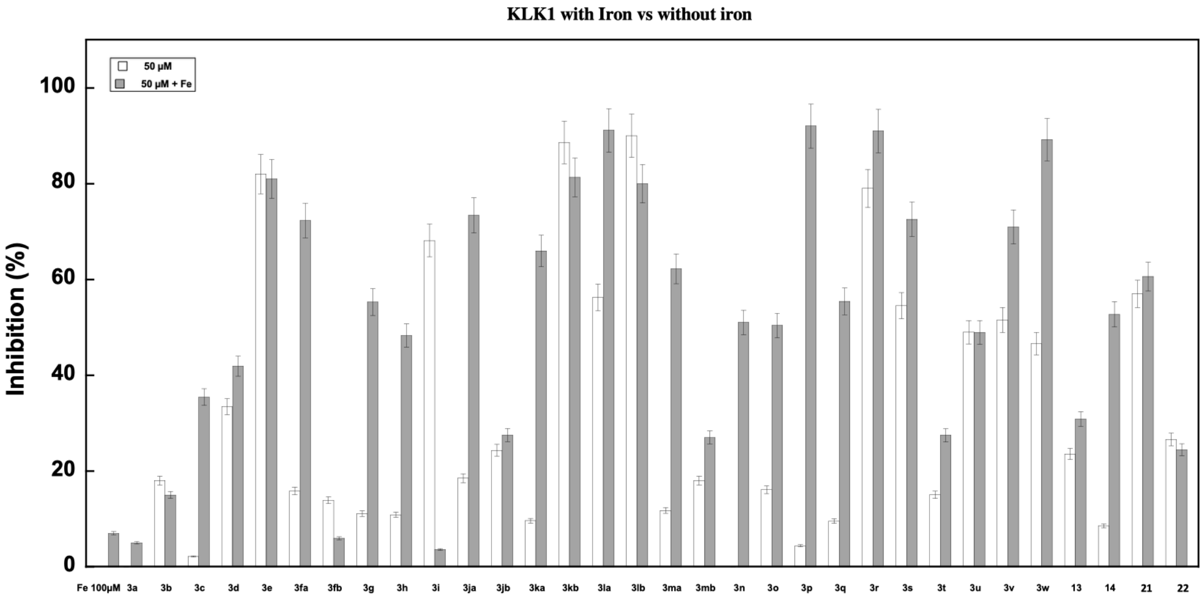

B. KLK6

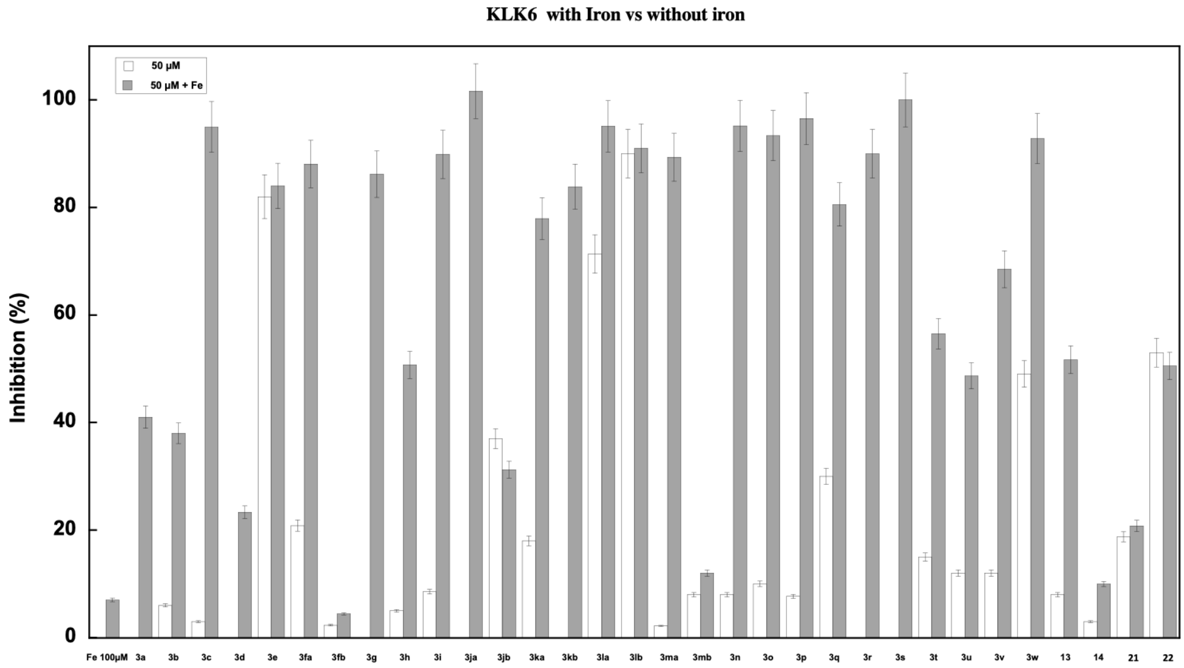

C. KLK8

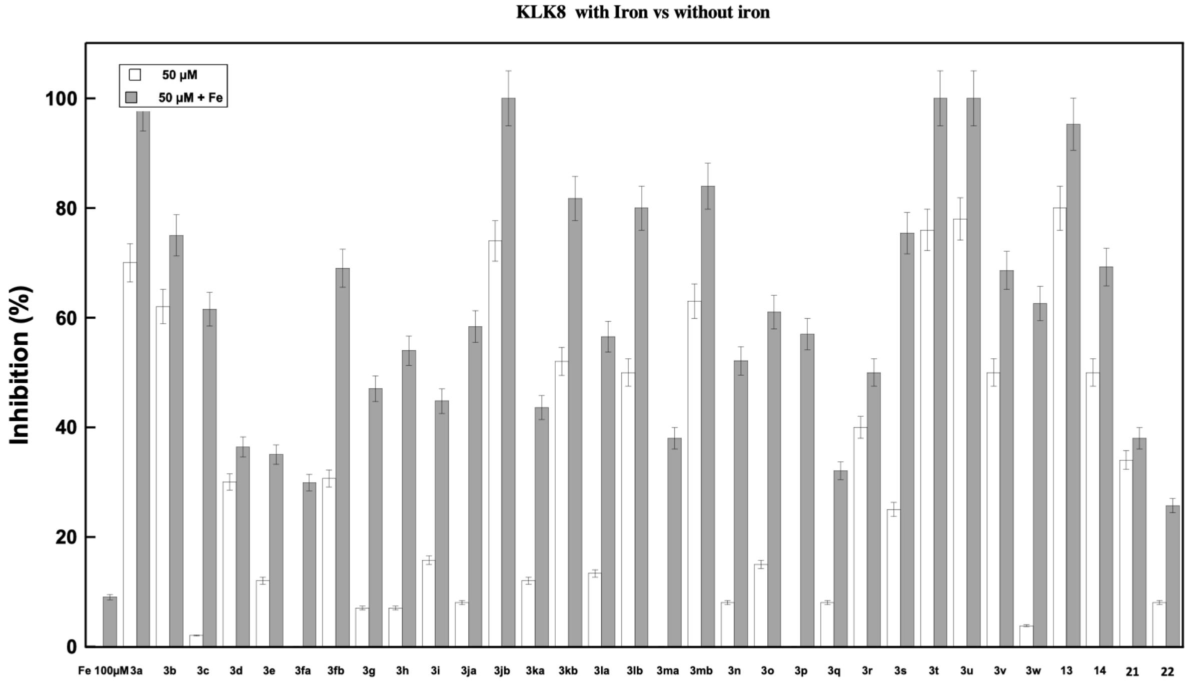

Table S1 : Selectivity profiles of hit compounds towards other members of KLK's family and selected representative proteases in the presence of 100 µM [Fe^{3+}]. The percentages of inhibition were determined with 50 µM of DFX-based derivatives in duplicate, in the optimal conditions of each protease (See material and methods section). NI: not inhibitory, percentage of inhibition below 50%.

	3a	3r	3e	3lb	3mb	3b
**KLK1**	NI	91.3	80.9	80.2	NI	NI
**KLK6**	NI	89.8	84.3	91.1	NI	NI
**KLK8**	99.2	NI	NI	80.3	84.9	75.5
**KLK4**	88.6	96.7	74.8	98.9	86.6	92.4
**KLK5**	92.1	92.7	97.8	95.9	98.2	97.1
**KLK7**	NI	80.4	80.1	90.8	NI	NI
**KLK11**	93.6	NI	NI	74.4	80.2	93.3
**KLK13**	96.1	96.1	98.2	97.2	87.2	99.2
**KLK14**	78.7	94.1	NI	97.9	NI	85.1
**Thrombin**	NI	54.4	NI	NI	85.2	NI
**Plasmin**	NI	NI	NI	65.5	NI	NI
**tPA**	NI	NI	NI	NI	NI	NI
**Matriptase**	NI	NI	NI	NI	NI	NI
**Cathepsin L**	NI	NI	NI	60.1	NI	NI

2.d. Molecular docking

Figure S6 : Molecular docking-interaction model of compound 3b, 3lb and 3mb with KLK8 (PDB: 5MS4). KLK8 is represented in surface mode with representation underlying the catalytic triad H57, D102, S195 (light blue), the different regulatory loops characteristic of KLK8, the calcium binding loop (green), the 99-loop or KLKs-loop (red).

KLK8-3b: **A.** Representation of KLK8 with the most favorable predicted pose of compound **3b** within the serine protease. **B.** First pose of compound **3b** within a pocket at the vicinity of the KLK8 active site of the active site. **C.** Interaction map of compound **3b** within the interaction pocket.

A.

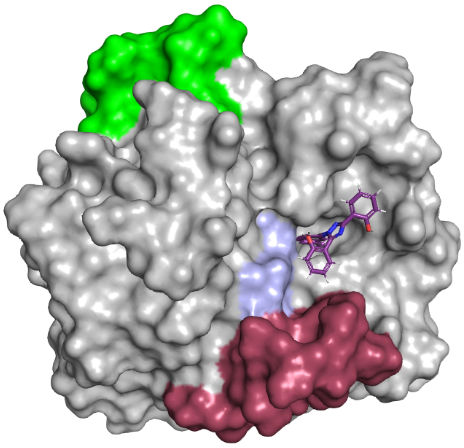

B.

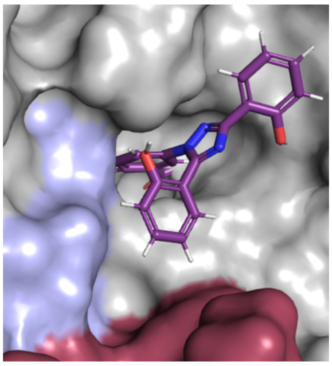

C.

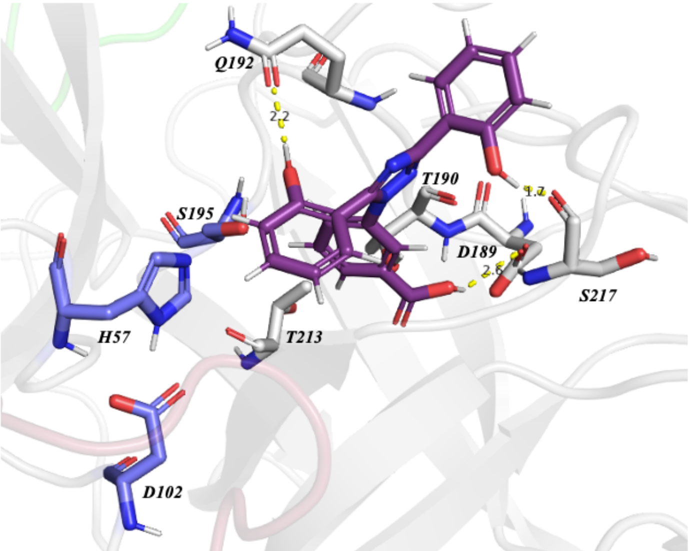

KLK8-3lb: D. Representation of KLK8 with the most favorable predicted pose of compound 3lb within the serine protease**. E.** First pose of compound **3lb** within a pocket at the vicinity of the KLK8 active site of the active site. **F.** Interaction map of compound **3lb** within the interaction pocket.

D.

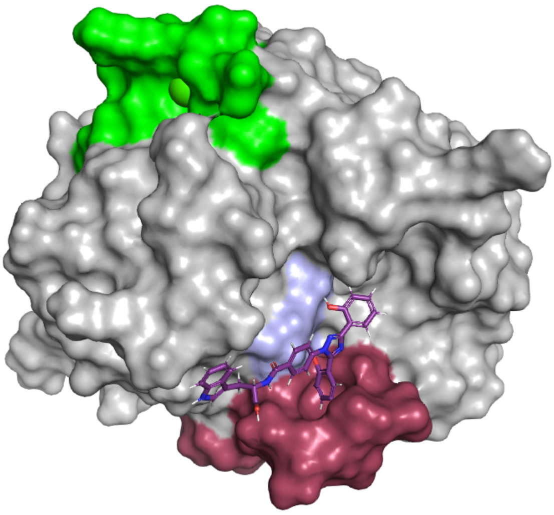

E.

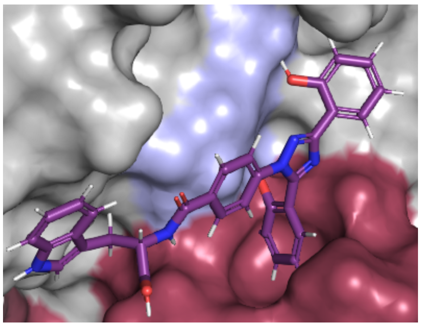

F.

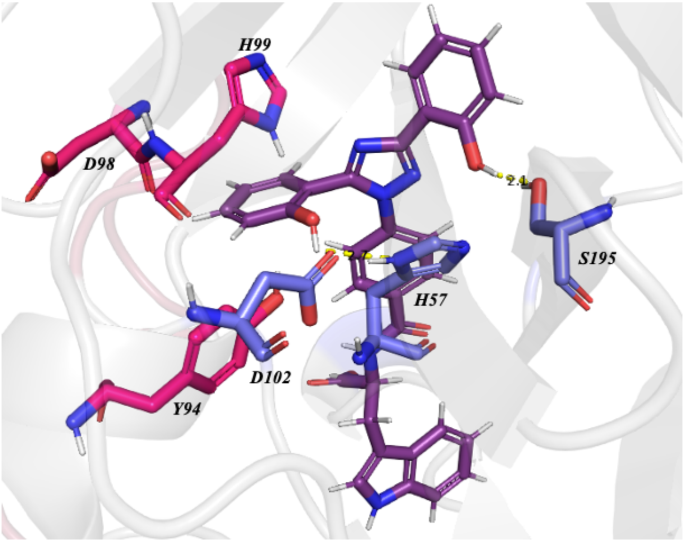

KLK8-3mb : G. Representation of KLK8 with the most favorable predicted pose of compound **3mb** within the serine protease**. H.** First pose of compound **3mb** within a pocket at the vicinity of the KLK8 active site of the active site. **I.** Interaction map of compound **3mb** within the interaction pocket.

G.

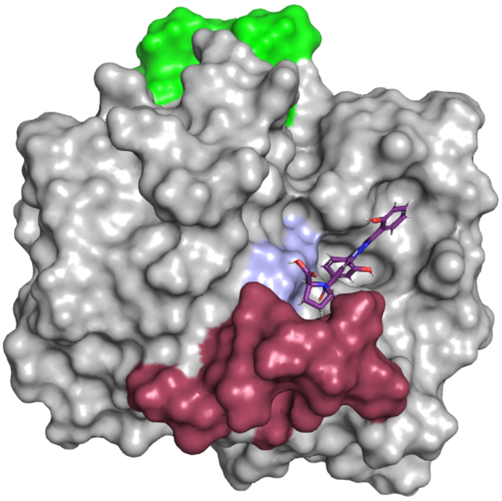

H.

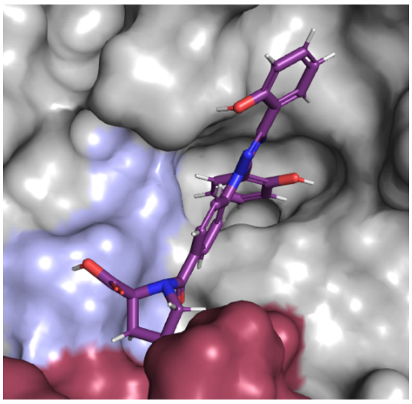

I.

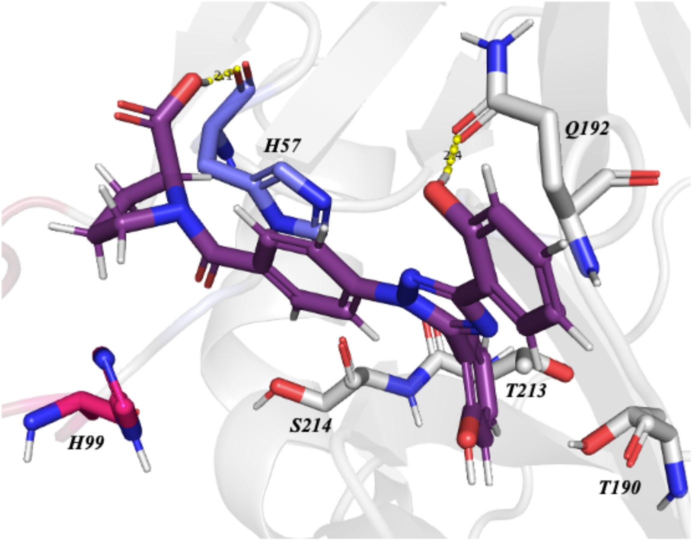

Figure S7 : Molecular docking-interaction model of compound 3e and 3r with KLK1 (PDB: 1SPJ). KLK1 is represented in surface mode with representation underlying the catalytic triad H57, D102, S195 (light blue), the different regulatory loops characteristic of KLK1, the calcium binding loop (green), the 99-loop or KLKs-loop (red).

KLK1 – 3e: A. Representation of KLK1 with the most favorable predicted pose of compound **3e** within the serine protease**. B**. First pose of compound **3e** within a pocket inside of the KLK1's S1' and S2' pockets of the active site. **C.** Interaction map of compound **3e** within the interaction pockets.

A.

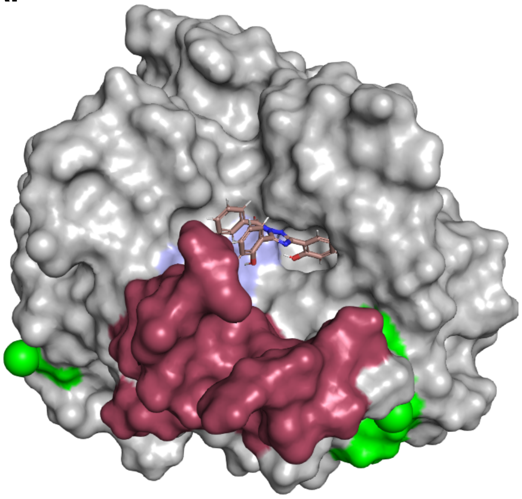

B.

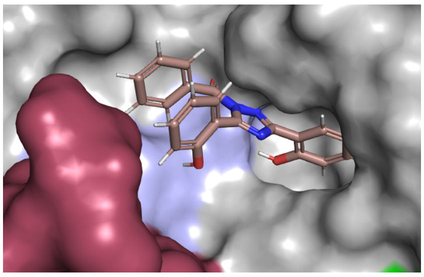

C.

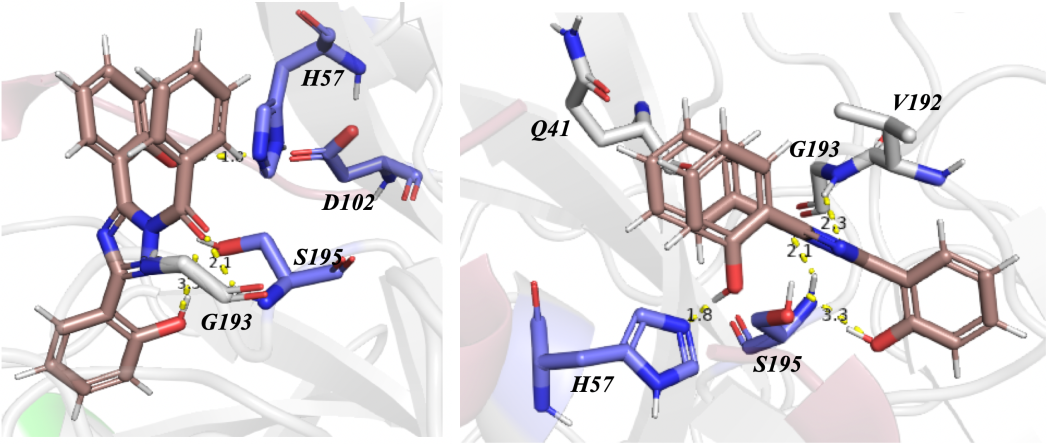

KLK1 – 3r: D. Representation of KLK1 with the most favorable predicted pose of compound **3r** within the serine protease. **E.** First pose of compound **3r** within a pocket inside of the KLK1's S1' and S2' pockets of the active site. **F.** Interaction map of compound **3r** within the interaction pockets. G. Interactive 2D diagram of compound **3r** generated from PoseEdit from the free available ProteinsPLus webserver (https://proteins.plus).

D.

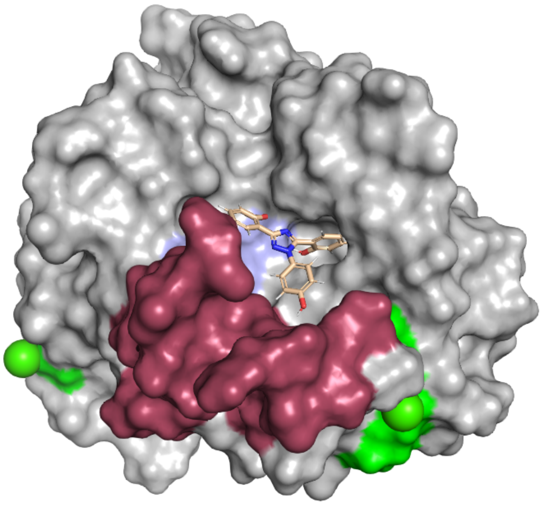

E.

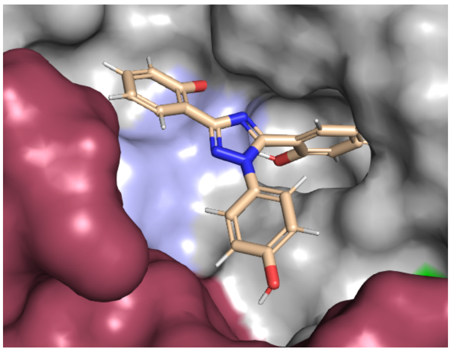

F.

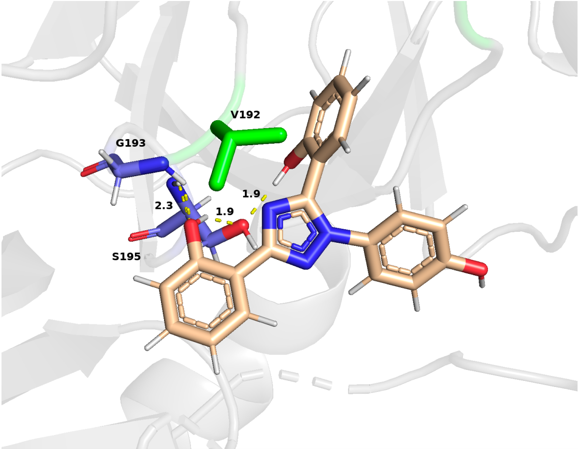

G.

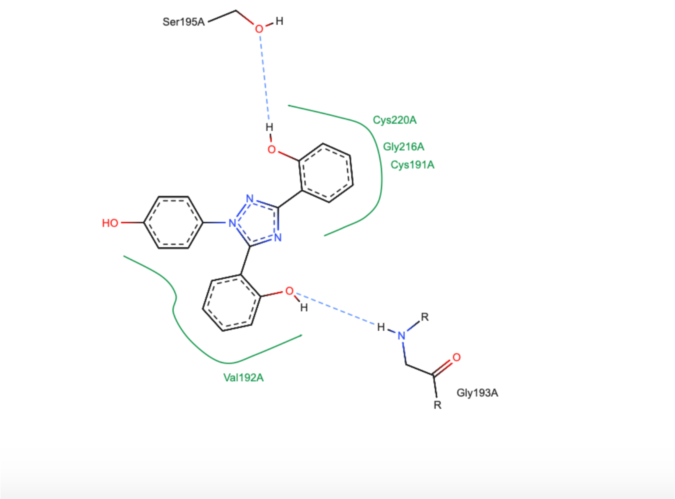

Figure S8 : Molecular docking-interaction model of compound 3lb with KLK6 (PDB: 1LO6). KLK6 is represented in surface mode with representation underlying the catalytic triad H57, D102, S195 (light blue) and the 99-loop or KLKs-loop (red). A. Representation of KLK6 with the most favorable predicted pose of compound **3lb** within the serine protease**.** B. First pose of compound **3lb** within a pocket inside of the KLK6 S1 pocket of the active site. C. Interaction map of compound 3lb within the interaction S1 pocket.

KLK6 – 3lb

A.

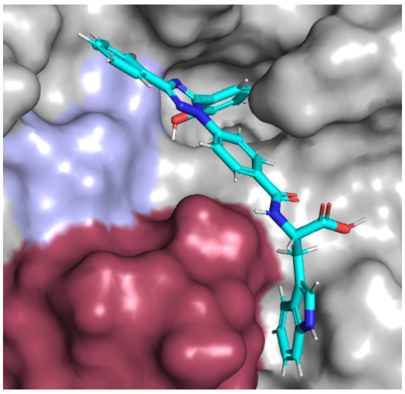

B.

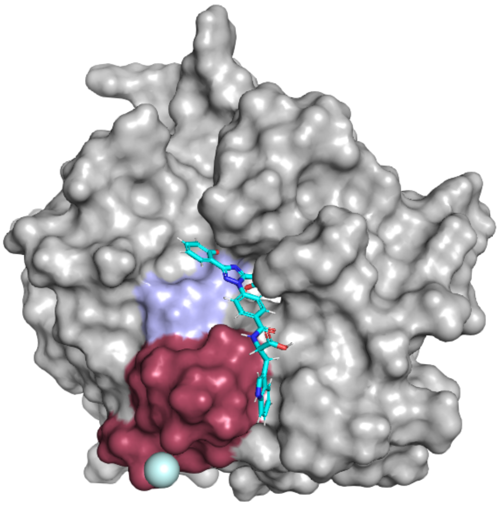

C.

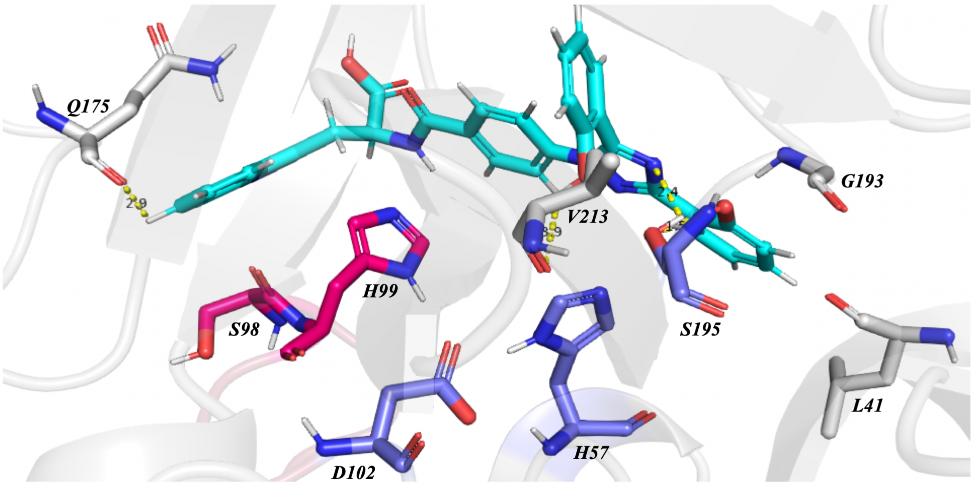

Figure S9: Specific features within the KLK Family – Focus on the Topology of Regulatory 220-Loops in Human KLKs 1, 6, and 8 within active sites
This figure illustrates the 220-loops of human (A) KLK1 (PDB: 1SPJ), (B) KLK6 (PDB: 1LO6), and (C) KLK8 (PDB: 5MS4) in surface representation. Residues that are conserved across all three enzymes are highlighted in beige. The amino acids at position 217 are distinctly colored to underline specific topology of each KLK: Tyr217 in light pink (KLK), Asn217 in yellow (KLK6), and Ser217 in red (KLK8).

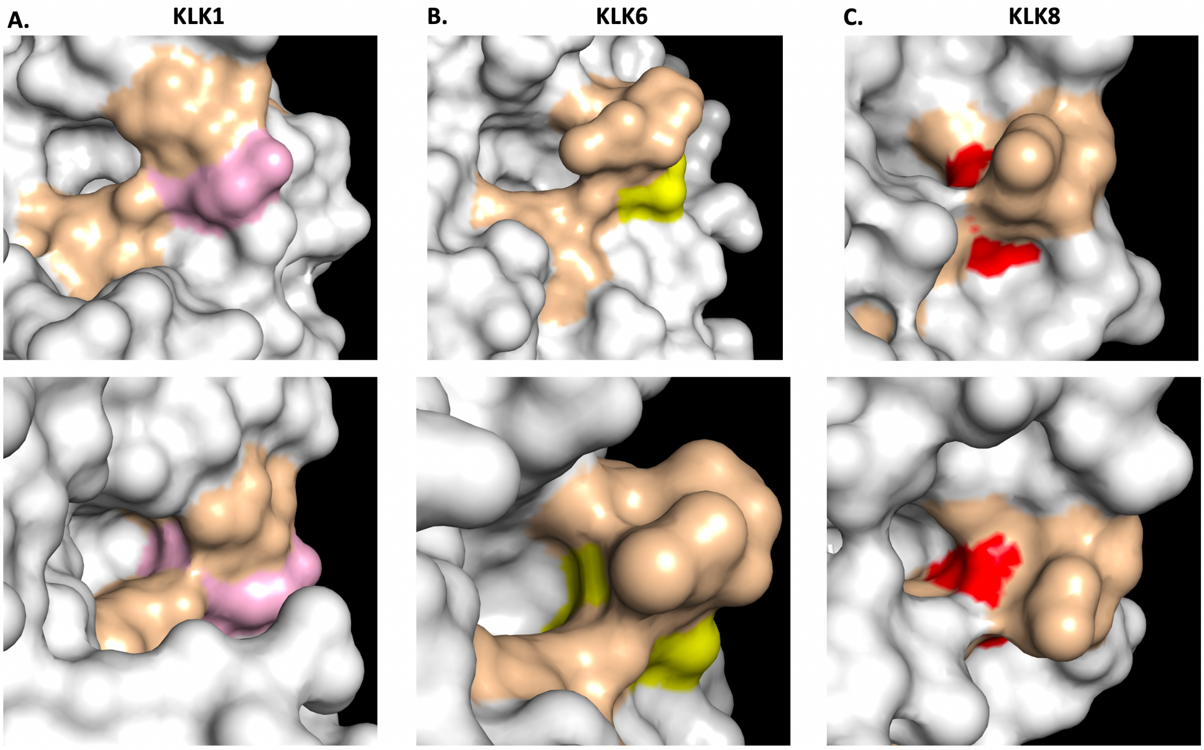

2.e. Cytotoxicity evaluation: experimental procedure

Human neuroblastoma cell line (SH-SY5Y) was purchased at American Type Culture Collection (ATCC, CRL-2266) and human microglial cell line (HMC3) was provided by Dr. El-Habr (IBPS, Paris, France). Both cell lines were cultured in Dulbecco's Modified Eagle Medium (DMEM) containing 10% heat inactivated fetal bovine serum (FBS), 1% Penicillin/Streptomycin, 10 mM Sodium Pyruvate, 2 mM L-Glutamine and 4,5 g/L D-glucose at 37°C, in a 5% humidified incubator. Cells were cultivated until 80% confluence in flasks then were plated in 96-well plates. For this experiment, SH-SY5Y were used at 10th passage and HMC3 at 13th. SH-SY5Y cells were seeded at a density of $3x10^4$ cells/well and HMC3 at a density of 10^4 cells/well. After seeding, cells were left in complete medium overnight to adhere to the plates. After that, cells were treated using inhibitors solubilized in DMSO at stock concentrations of 2,5 mM or 5 mM with or without 10 mM iron. Iron solution was prepared by using FAC (Ferric Ammonium Citrate, Sigma®) at stock concentration of 25 mM. After 24h of treatment, cell viability was assessed with the CCK8 (Cell-Counting Kit 8) from TargetMol. After treatments, 10 µL of CCK8 reagent was diluted in 90 µL of complete medium and added to each well. After 3 hours at 37°C, cell viability was assessed by absorbance measurement at 460 nm. Each viability assay is the mean of two independent experiments performed in triplicates.

Figure S10 : Cell viability assay after exposure of HMC3 (A) and SH-SY5Y (B) cell lines at 25 and 50 µM of hit inhibitor without (White) or with 100 µM of FAC (Ferric ammonium citrate) (Gray).Cell viability was assessed using the WST8 - Cell-Counting Kit 8. Cells were treated for 24h with the two selected concentrations of inhibitor (25 µM or 50 µM) preincubated or not with 100 µM of FAC. The data are the mean of two independent experiments performed in triplicates.

A.

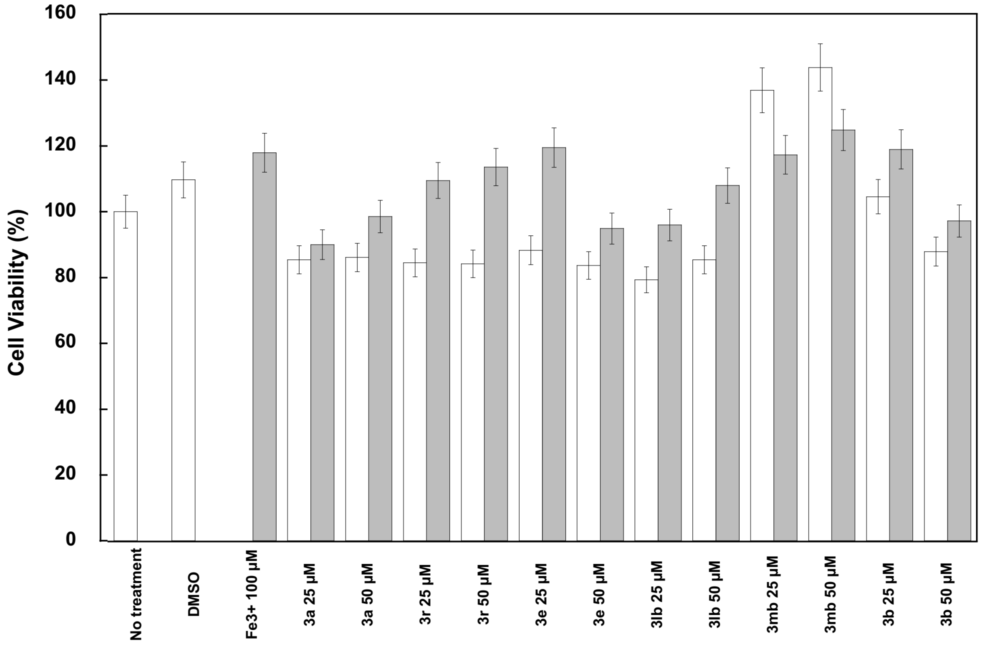

B.

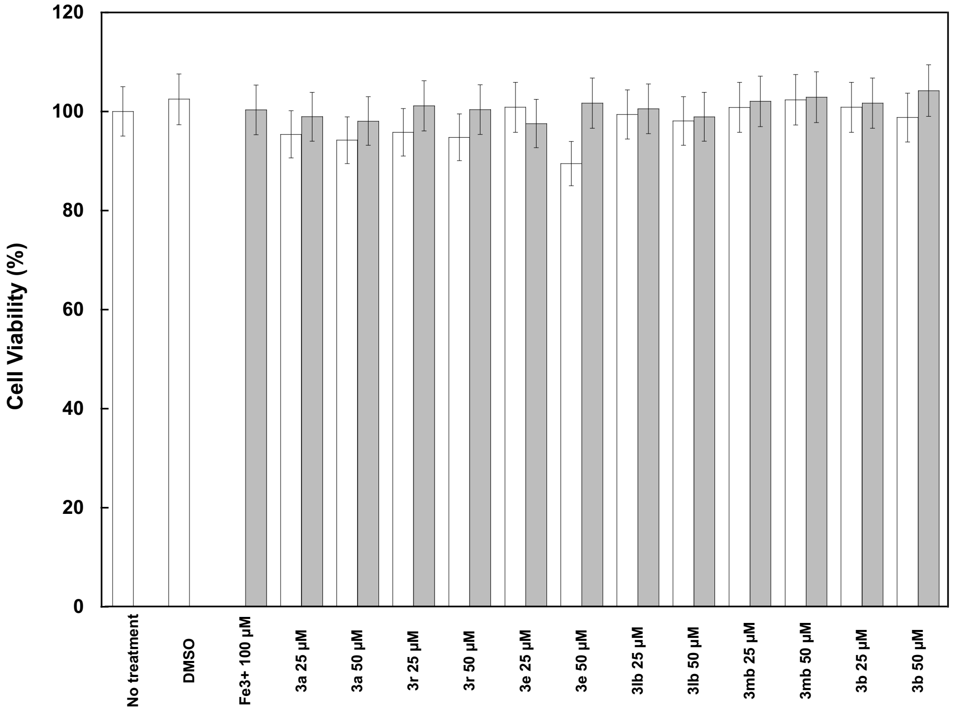

3. ^{1}H and ^{13}C NMR spectra of new compounds

Figure S11 : 1H and 13C NMR spectra of compound 3d

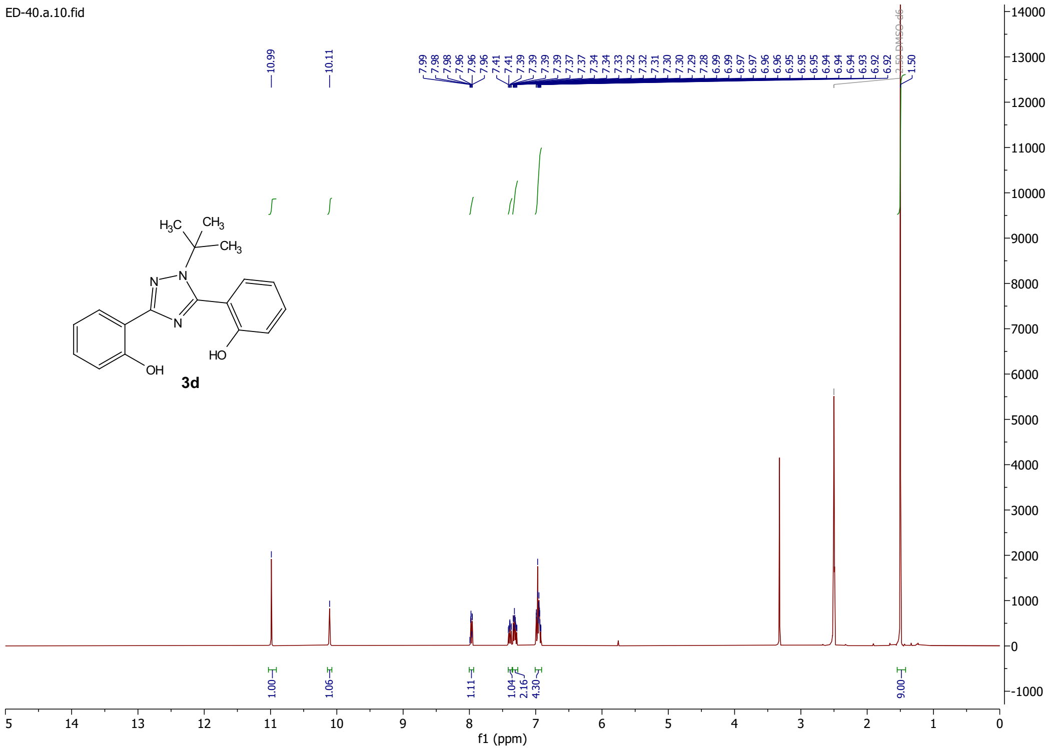

1H NMR spectrum of 3d (400 MHz, DMSO-d6)

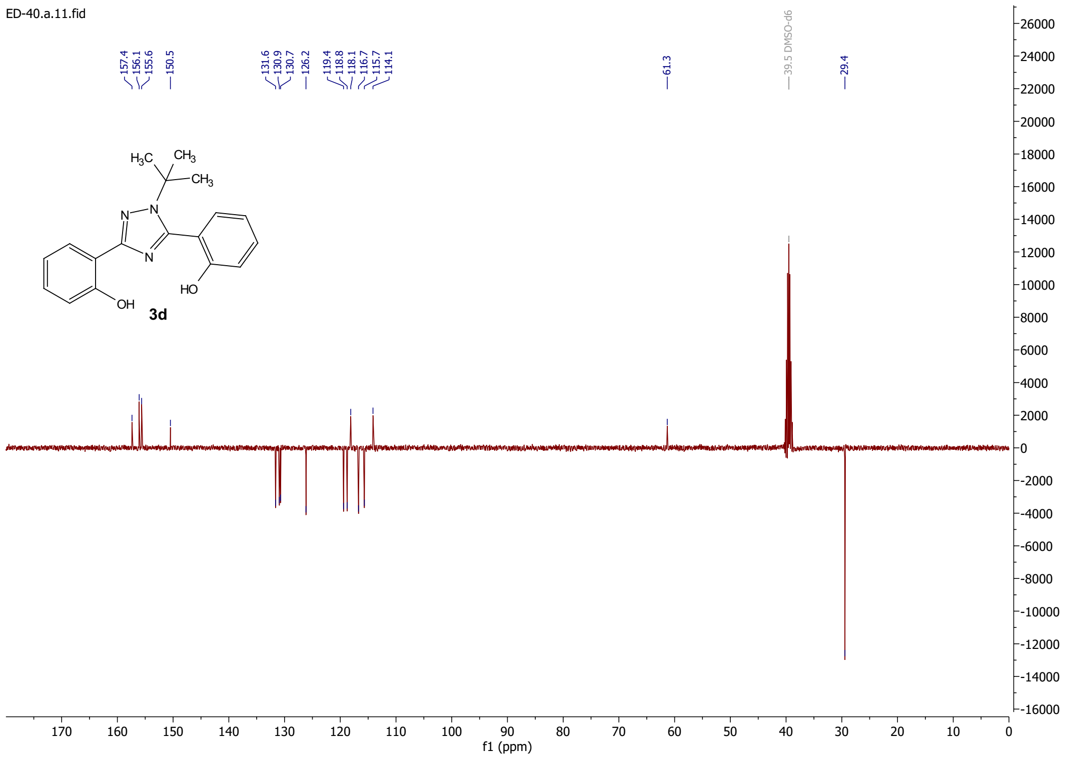

13C (JMOD) NMR spectrum of 3d (100 MHz, DMSO-d6)

Figure S12 : ^{1}H and ^{13}C NMR spectra of compound of (±)-Ethyl 4-[(tert-butyloxycarbonyl)hydrazono]cyclohexanecarboxylate

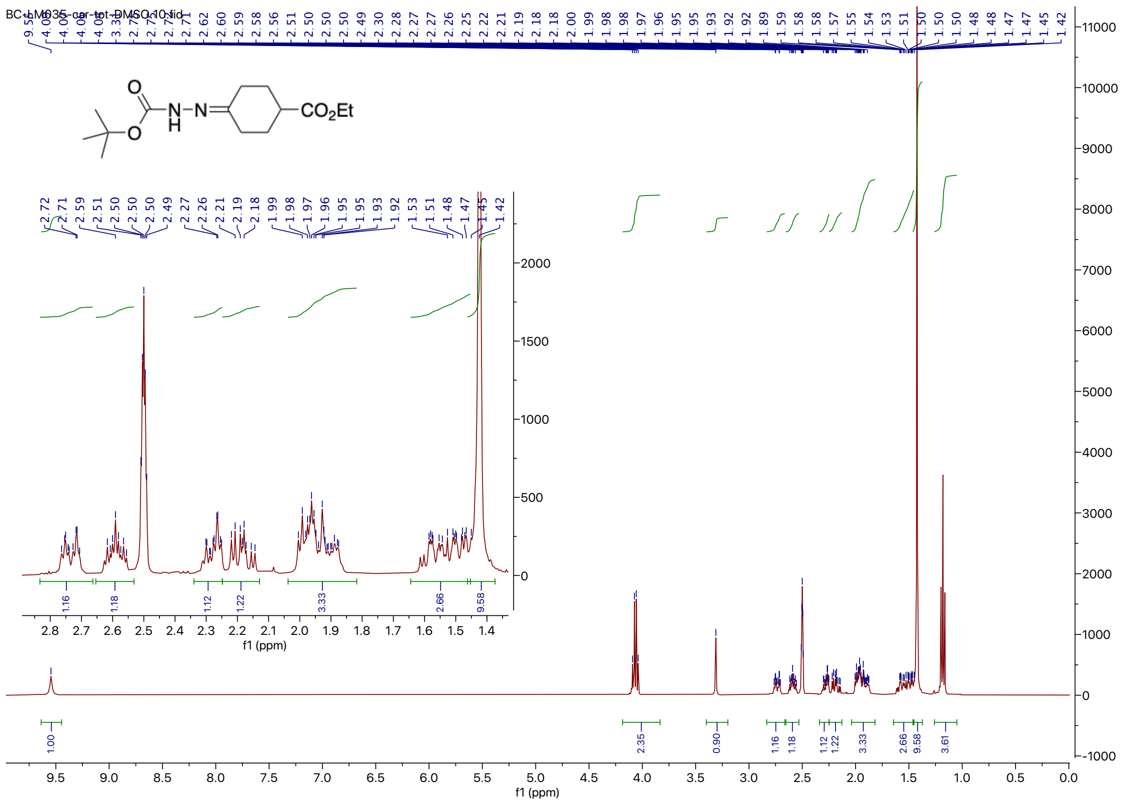

1H NMR spectrum (400 MHz, DMSO-d6)

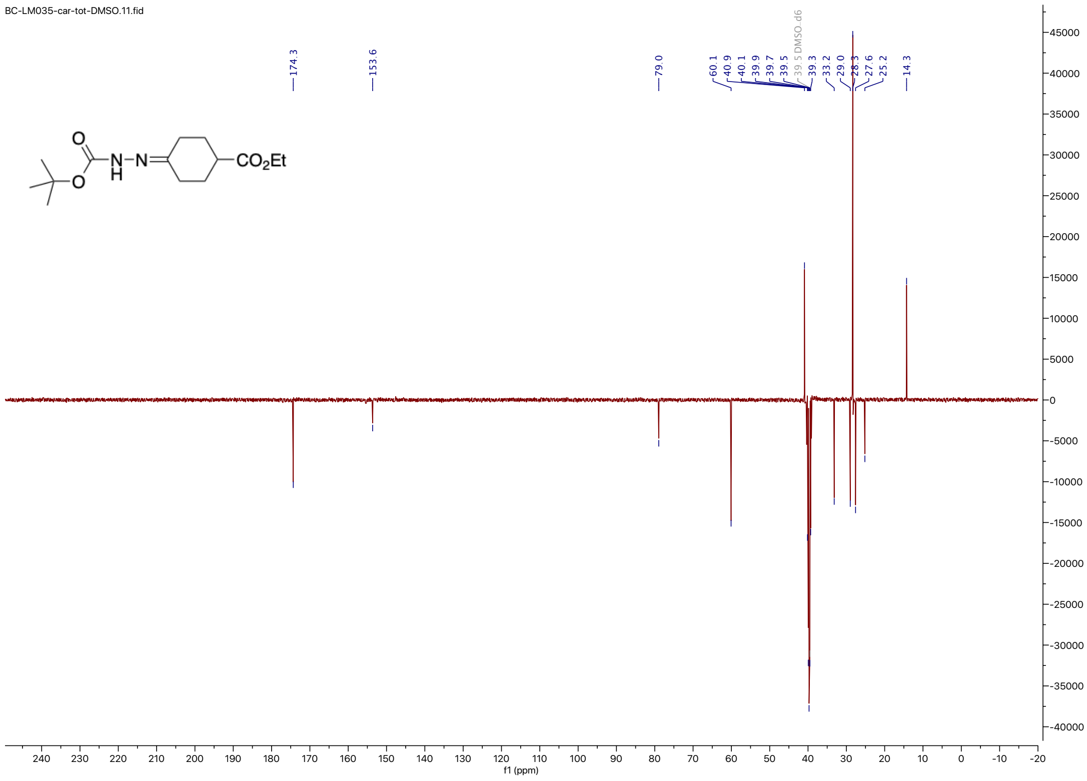

13C (JMOD) NMR spectrum of (100 MHz, DMSO-d6)

Figure S13 : ^{1}H and ^{13}C NMR spectra of (±)-Trans-ethyl 4-[2-(tert-: butyloxycarbonyl)hydrazino]cyclohexanecarboxylate

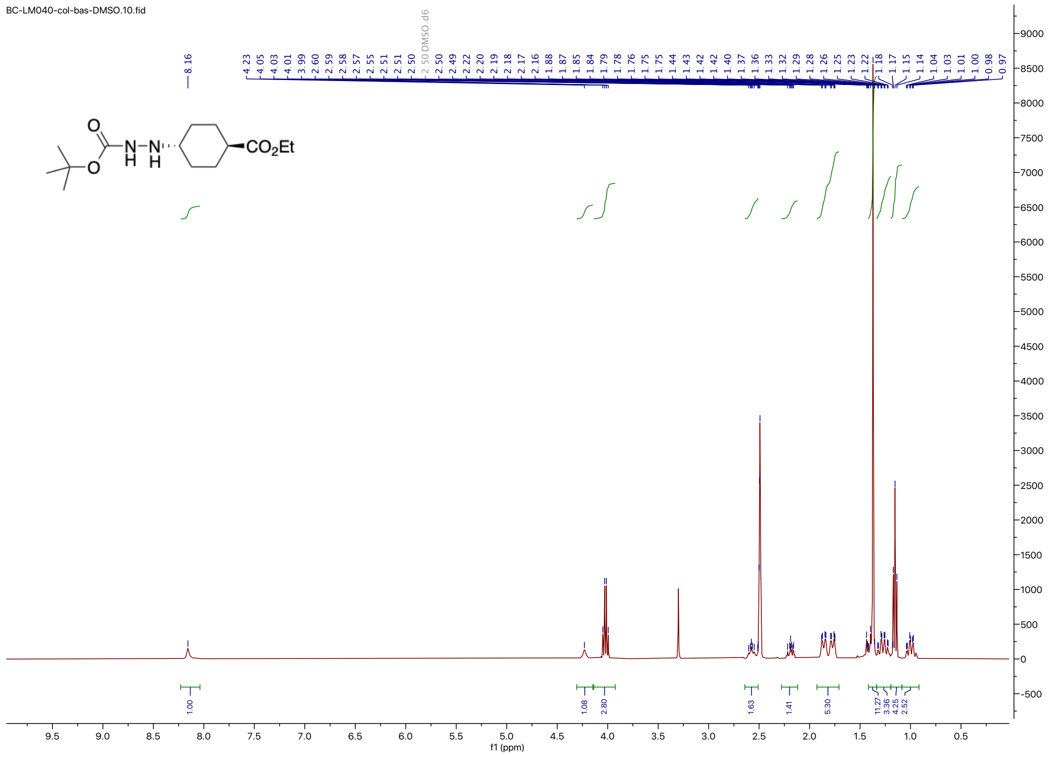

1H NMR spectrum (400 MHz, DMSO-d6)

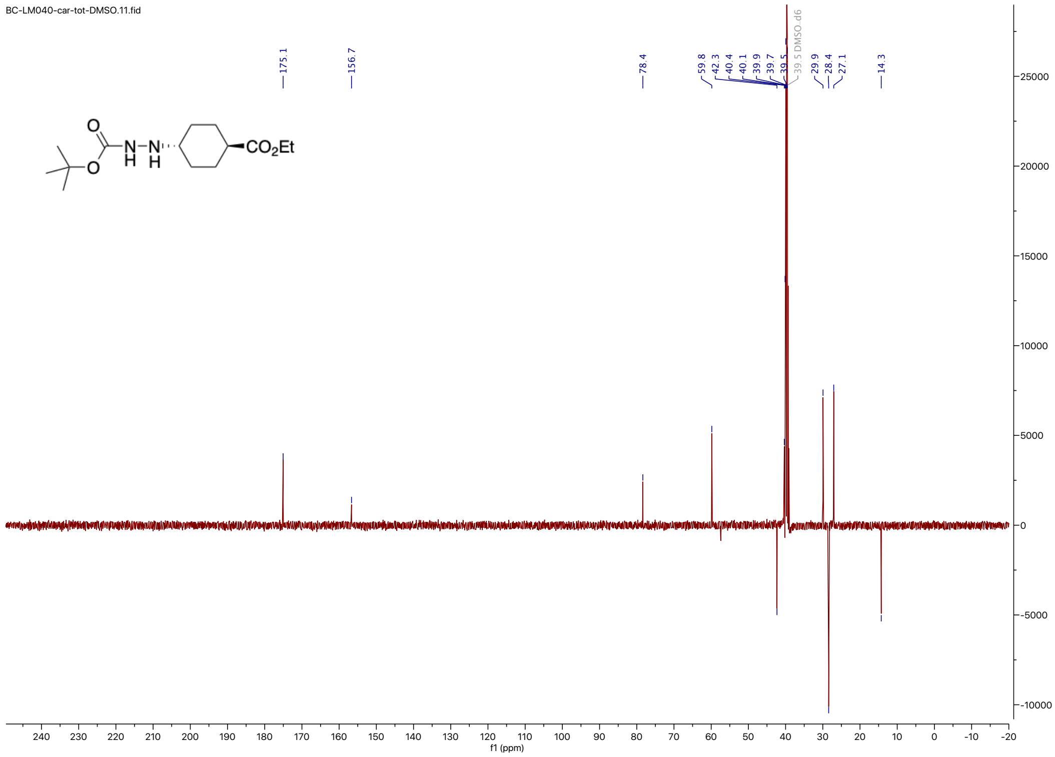

13C (JMOD) NMR spectrum of (100 MHz, DMSO-d6)

Figure S14 : ^{1}H and ^{13}C NMR spectra of (±)-Trans-ethyl-4-hydrazinylcyclohexanecarboxylate hydrochloride

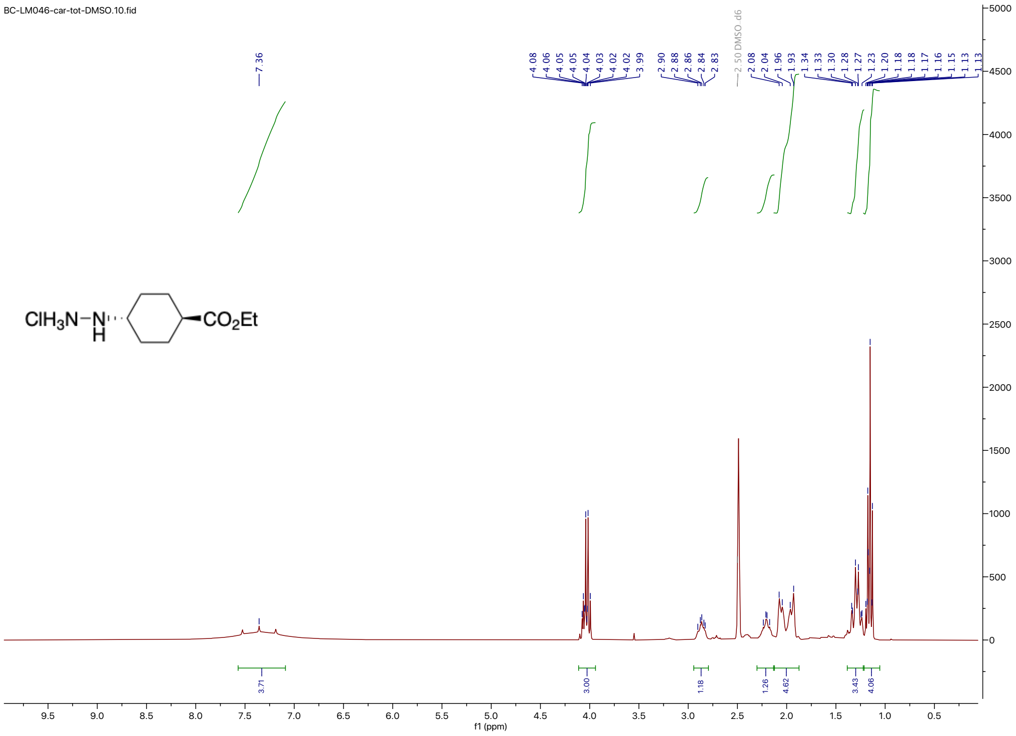

1H NMR spectrum (400 MHz, DMSO-d6)

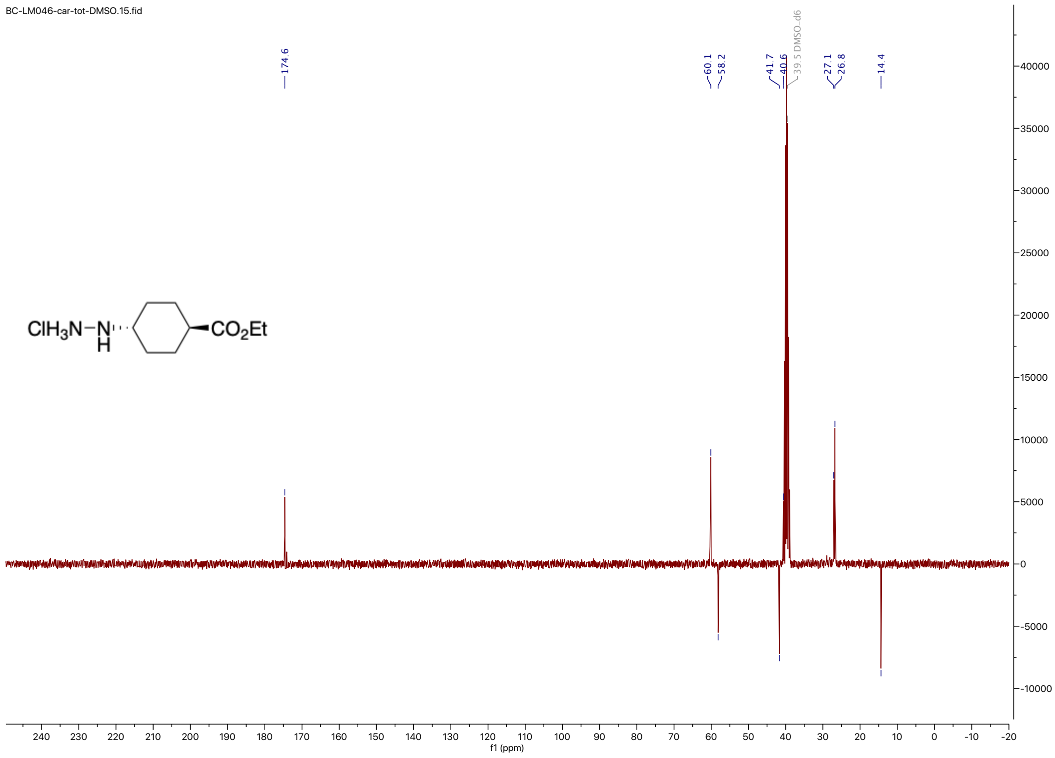

13C (JMOD) NMR spectrum of (100 MHz, DMSO-d6)

Figure S15 : ^{1}H and ^{13}C NMR spectra of 3fa

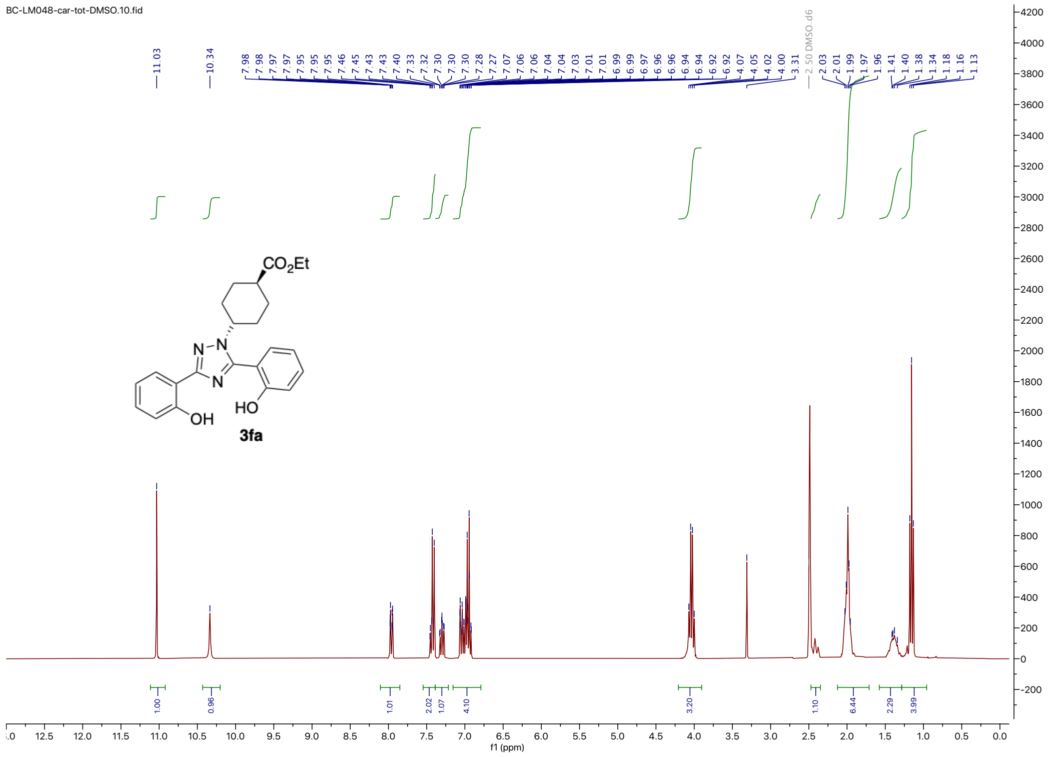

1H NMR spectrum (300 MHz, DMSO-d6) of 3fa

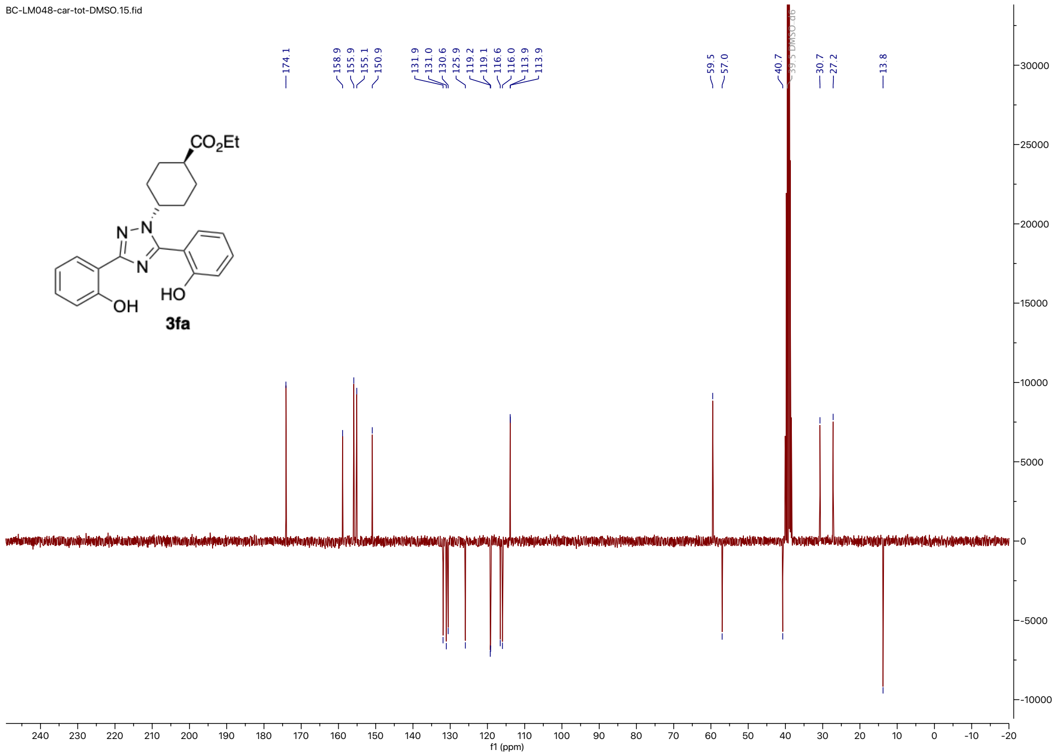

13C (JMOD) NMR spectrum of 3fa (75 MHz, DMSO-d6)

Figure S16 : ^{1}H and ^{13}C NMR spectra of 3fb

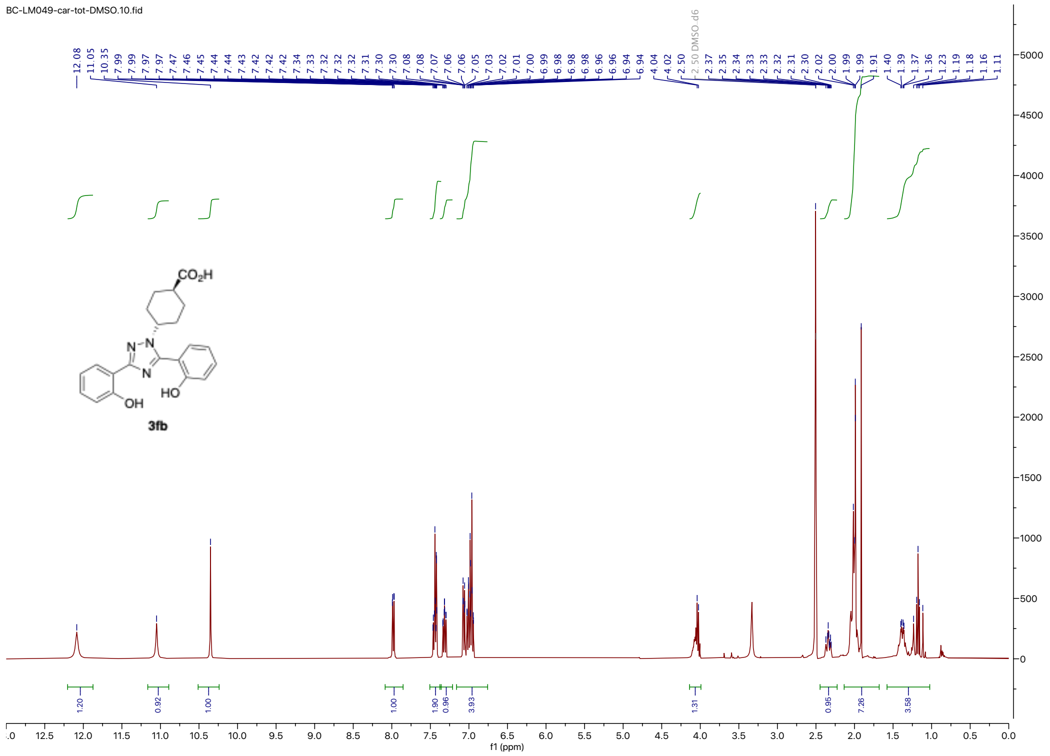

1H NMR spectrum (400 MHz, DMSO-d6) of 3fb

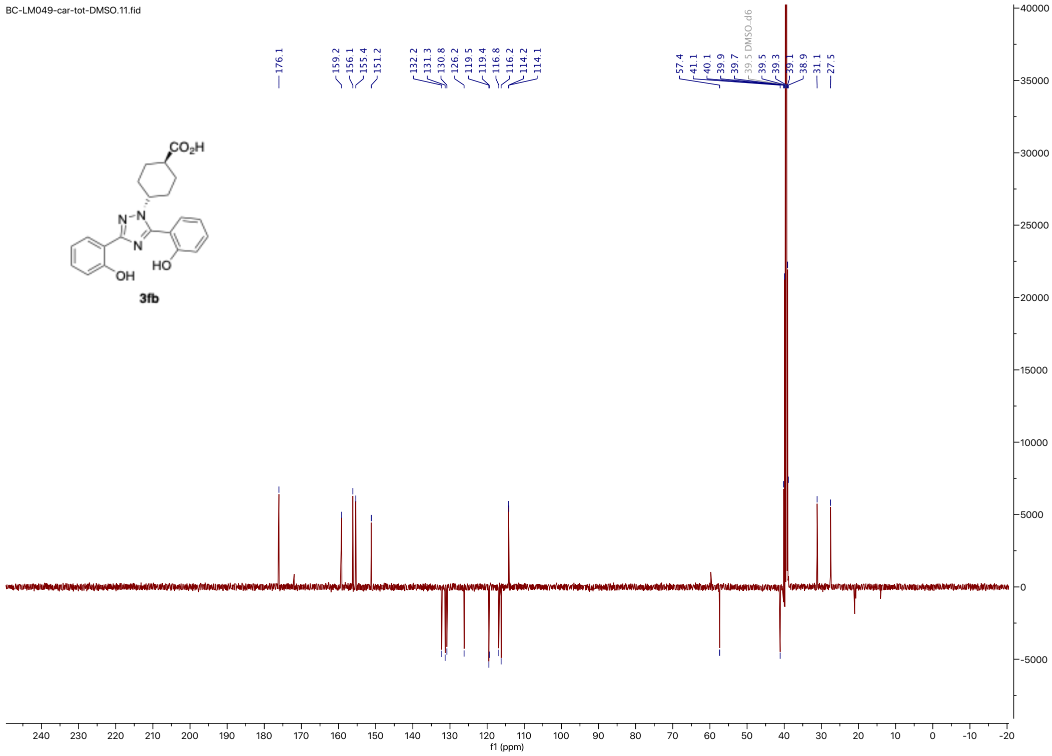

13C (JMOD) NMR spectrum of 3fb (100 MHz, DMSO-d6)

Figure S17 : ^{1}H and ^{13}C NMR spectra of 3g

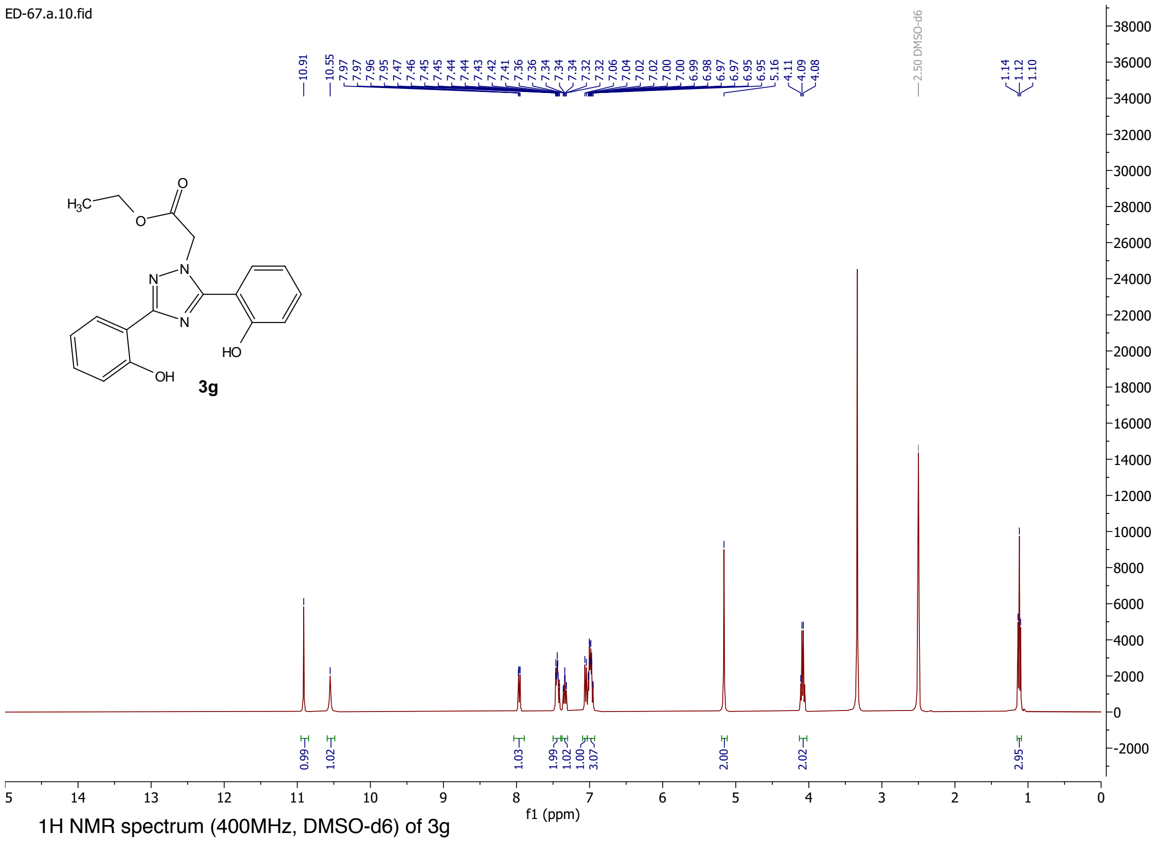

1H NMR spectrum (400MHz, DMSO-d6) of 3g

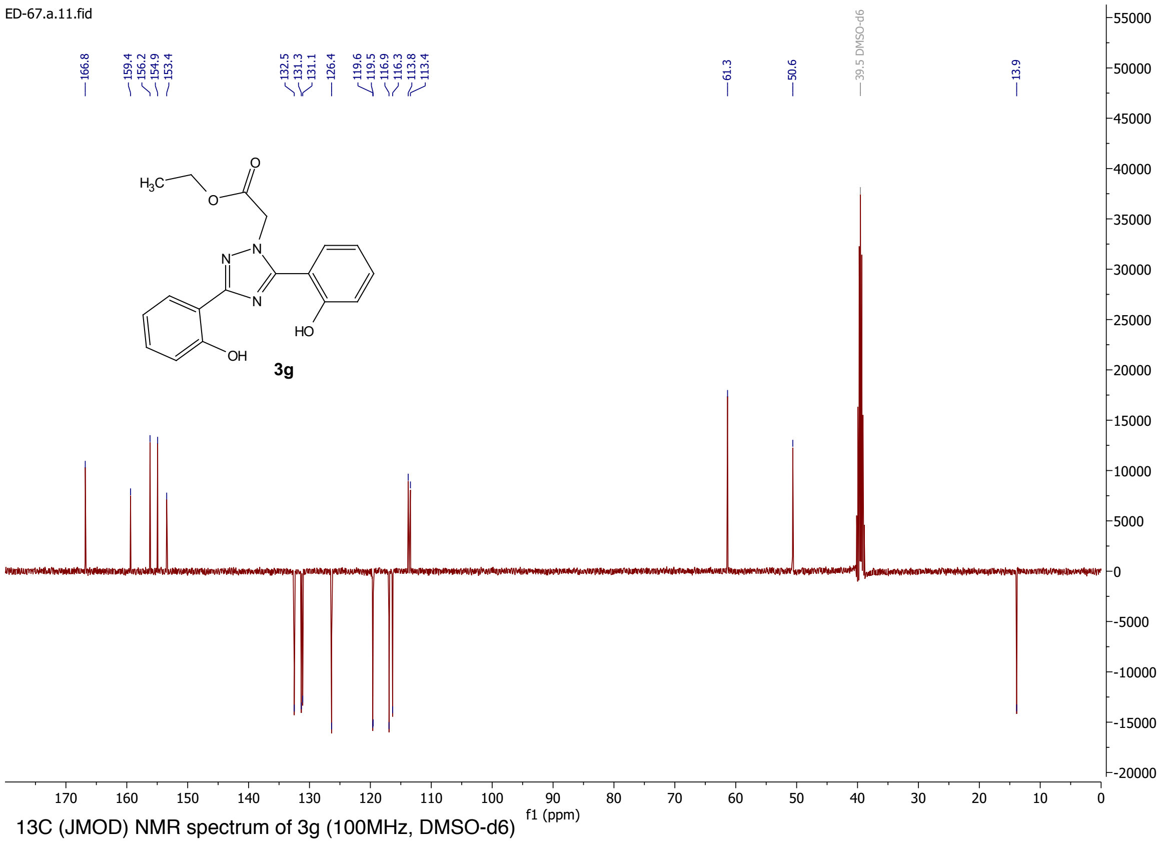

13C (JMOD) NMR spectrum of 3g (100MHz, DMSO-d6)

Figure S18 : ^{1}H and ^{13}C NMR spectra of 3h

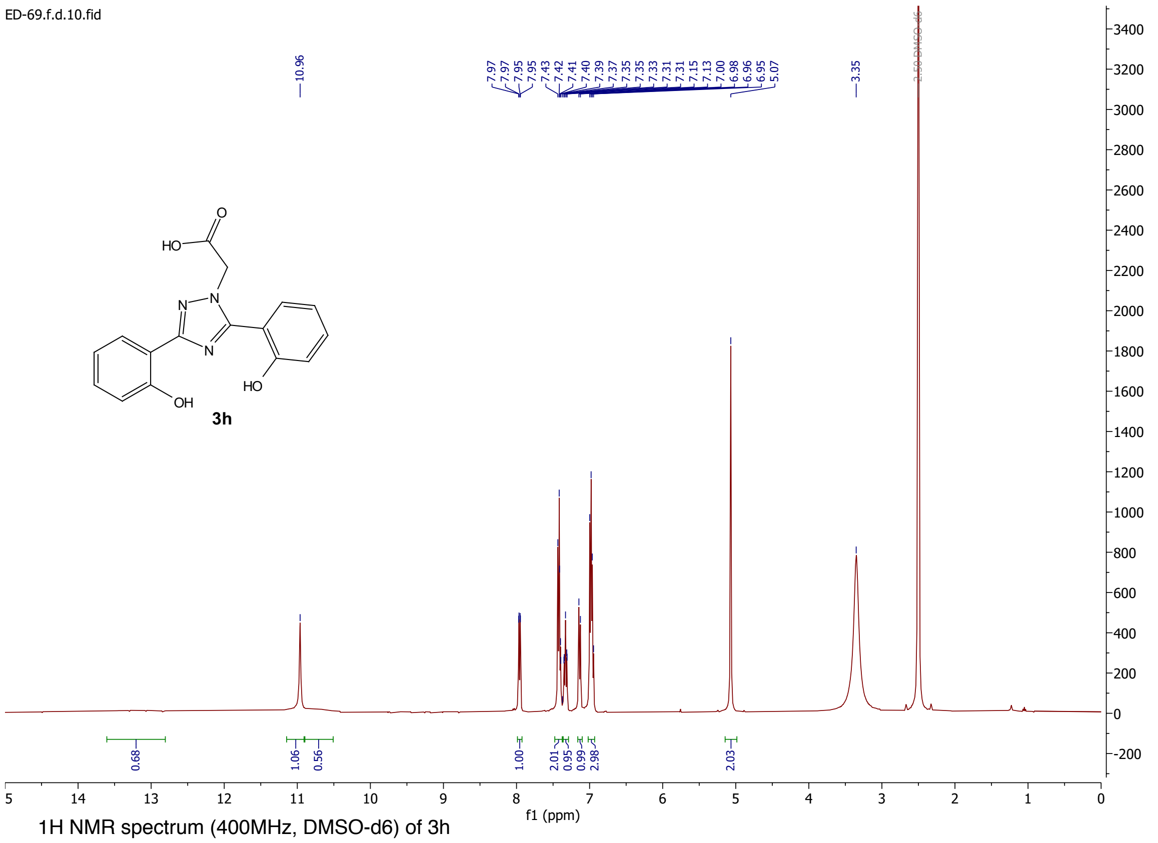

1H NMR spectrum (400MHz, DMSO-d6) of 3h

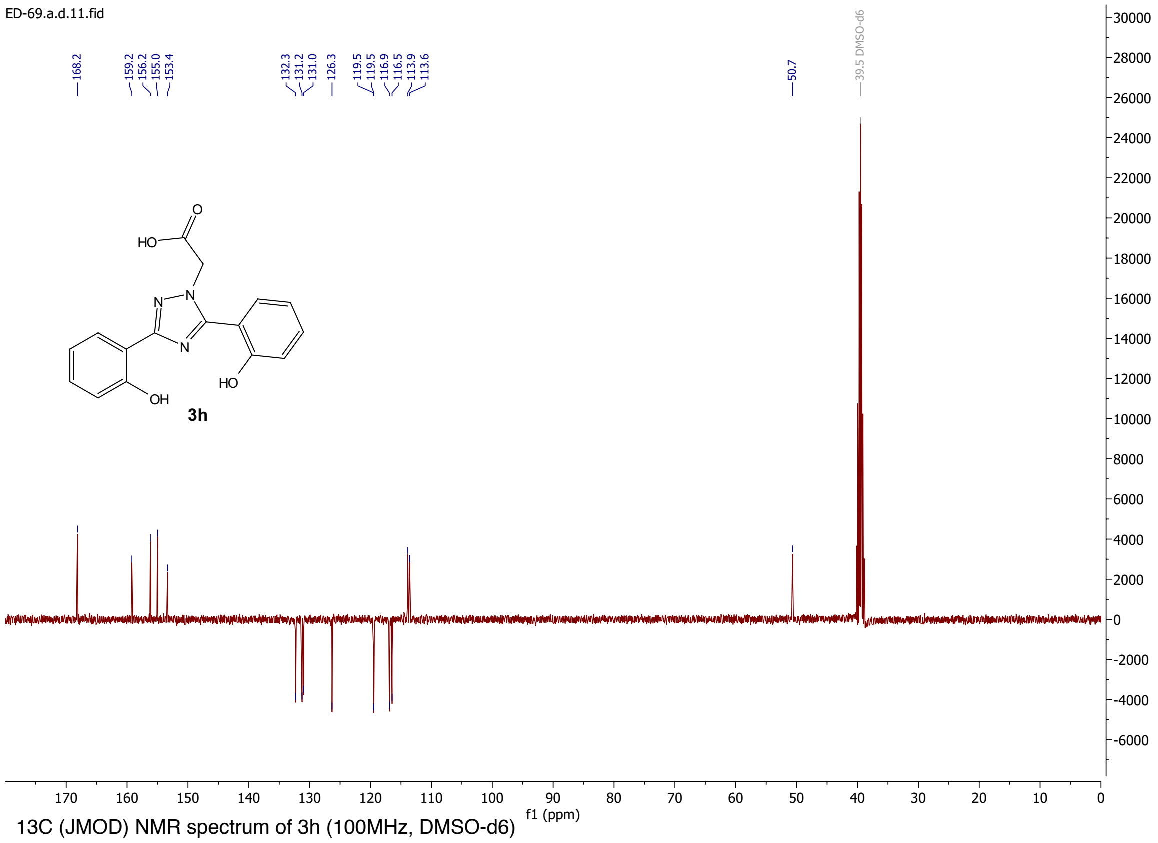

13C (JMOD) NMR spectrum of 3h (100MHz, DMSO-d6)

Figure S19 : ^{1}H and ^{13}C NMR spectra of 3ja

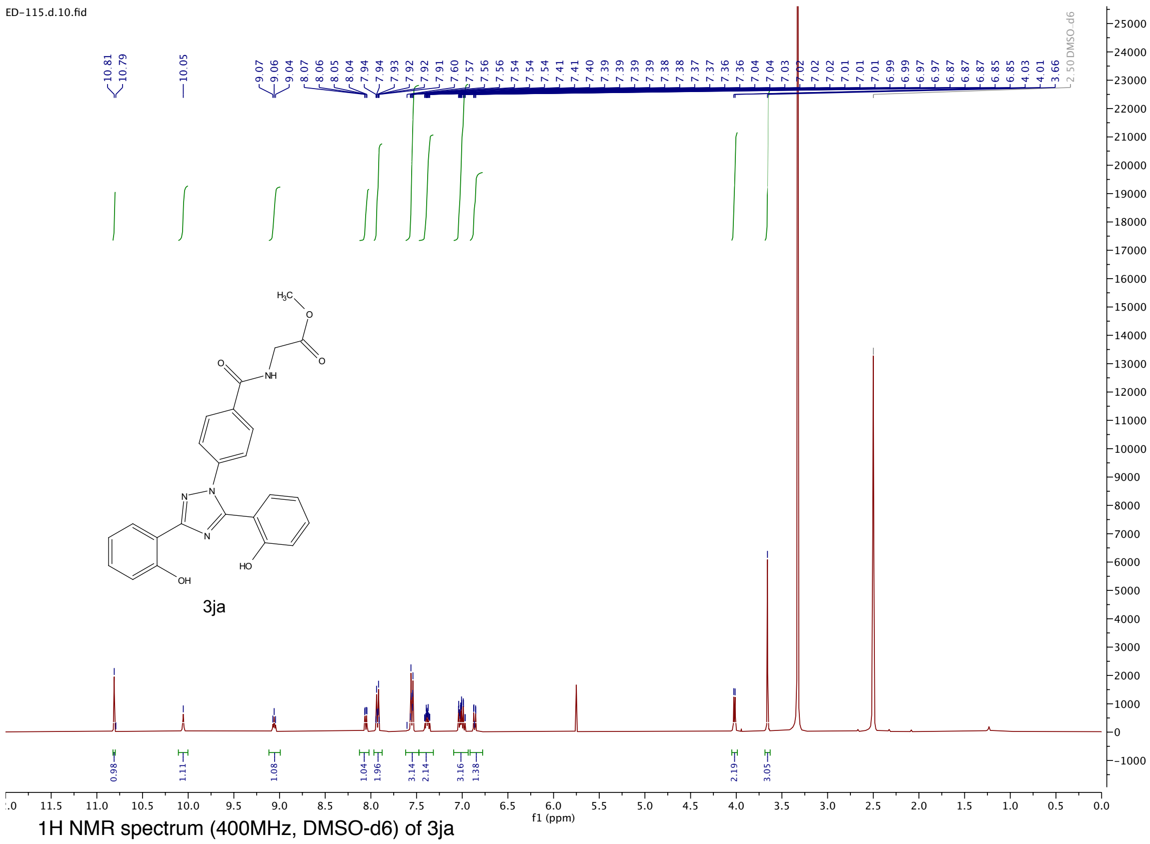

1H NMR spectrum (400MHz, DMSO-d6) of 3ja

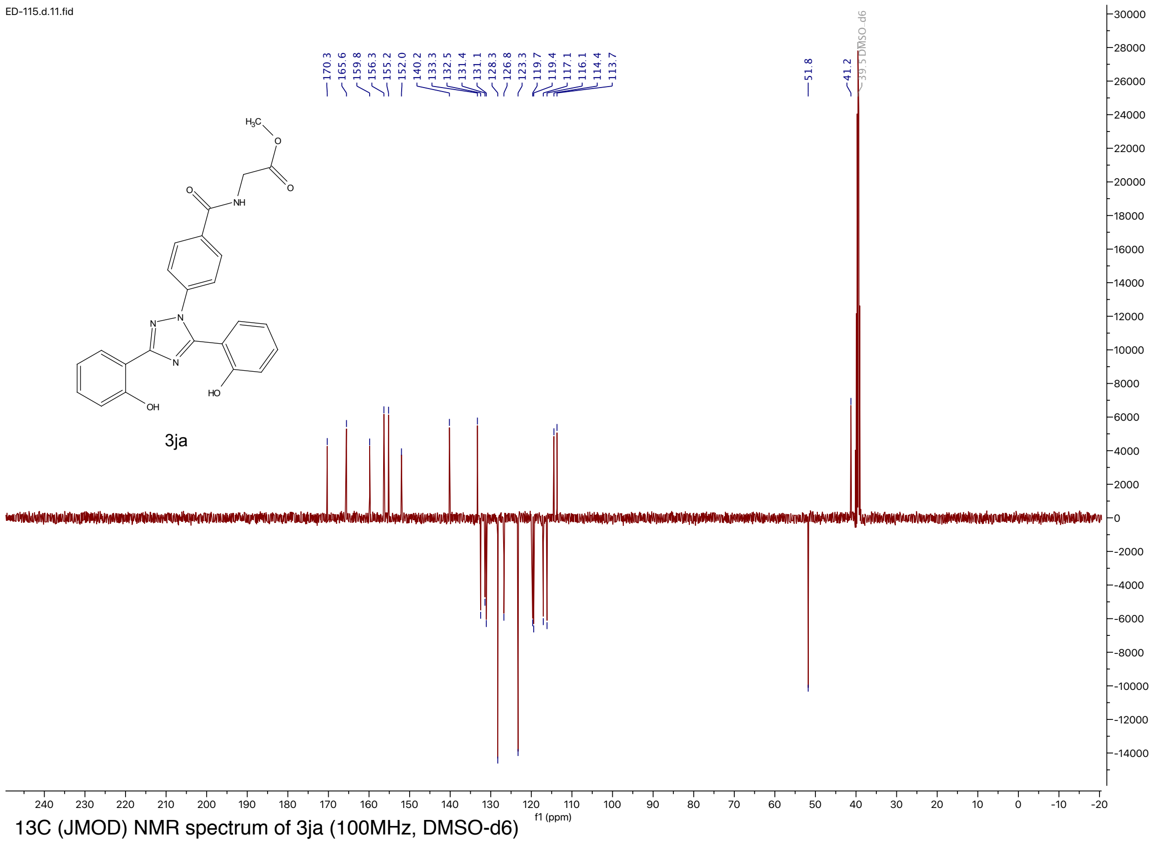

13C (JMOD) NMR spectrum of 3ja (100MHz, DMSO-d6)

Figure S20 : ^{1}H and ^{13}C NMR spectra of 3jb

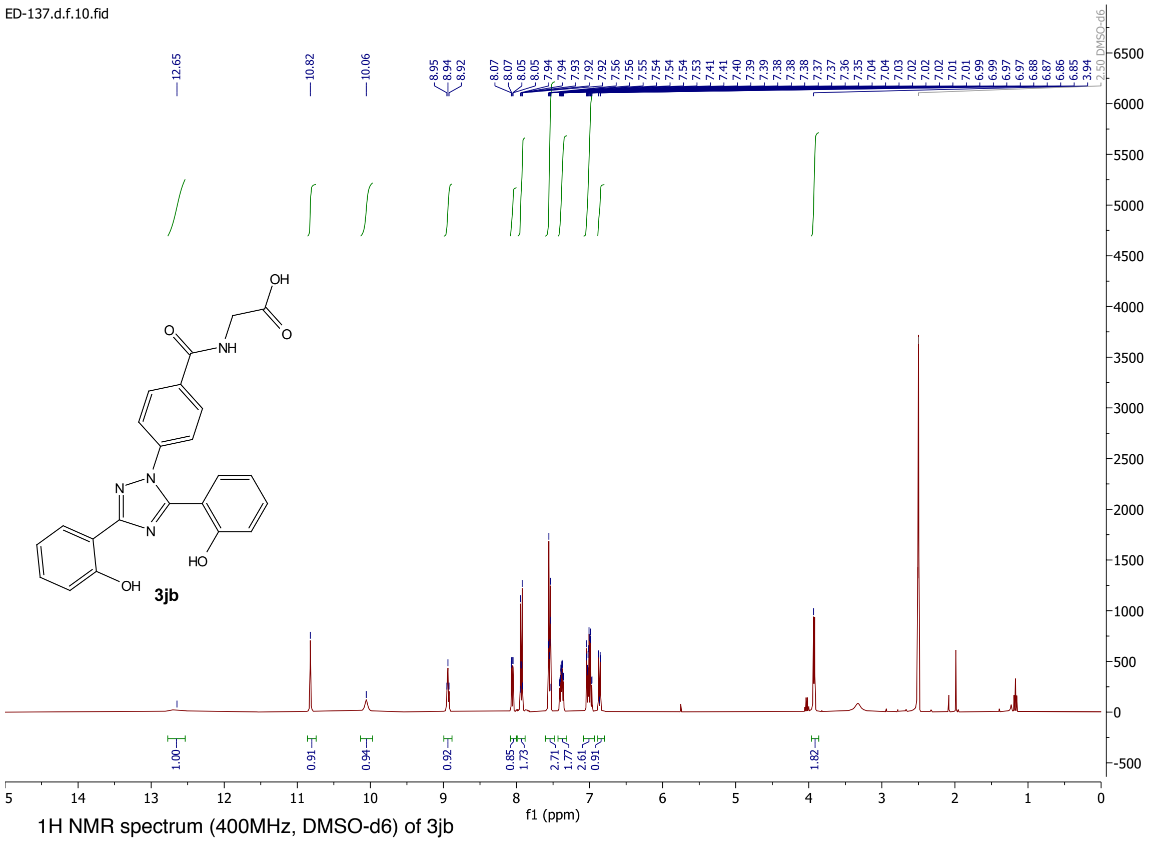

1H NMR spectrum (400MHz, DMSO-d6) of 3jb

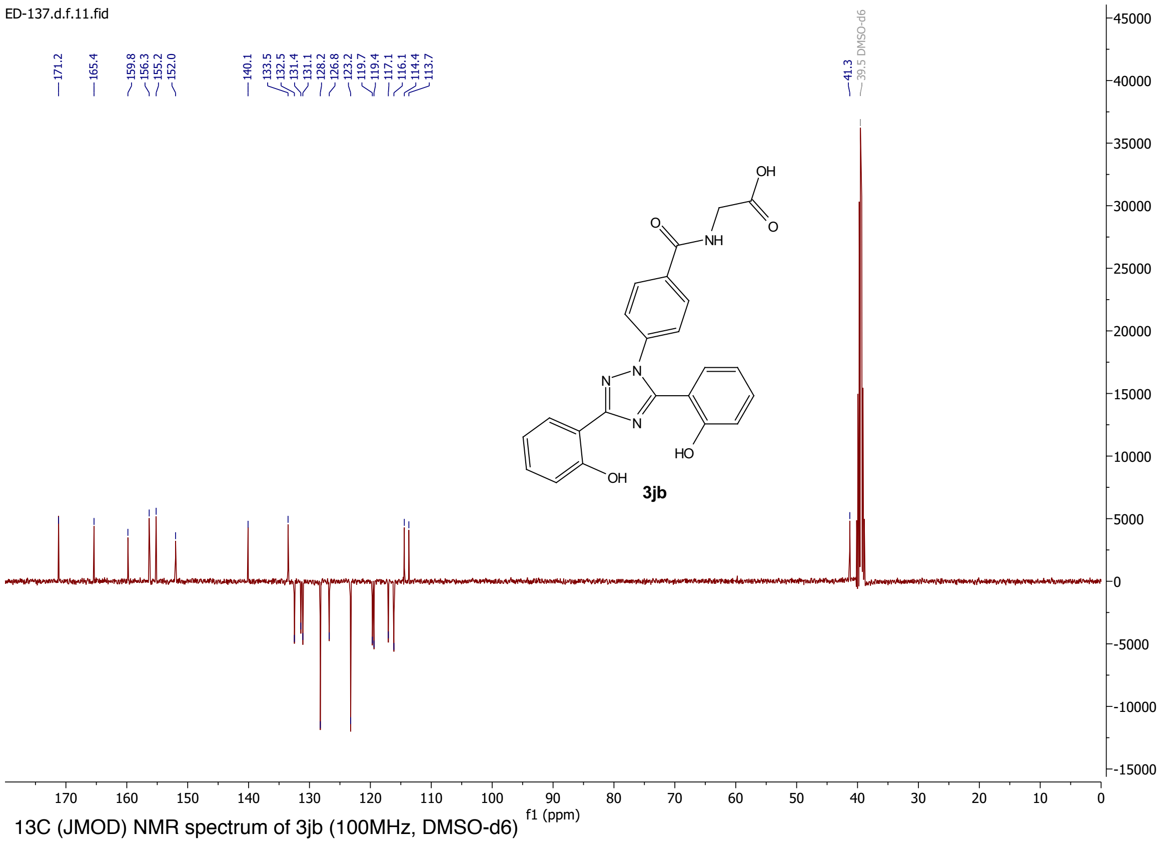

13C (JMOD) NMR spectrum of 3jb (100MHz, DMSO-d6)

Figure S21 : ^{1}H and ^{13}C NMR spectra of 3kb

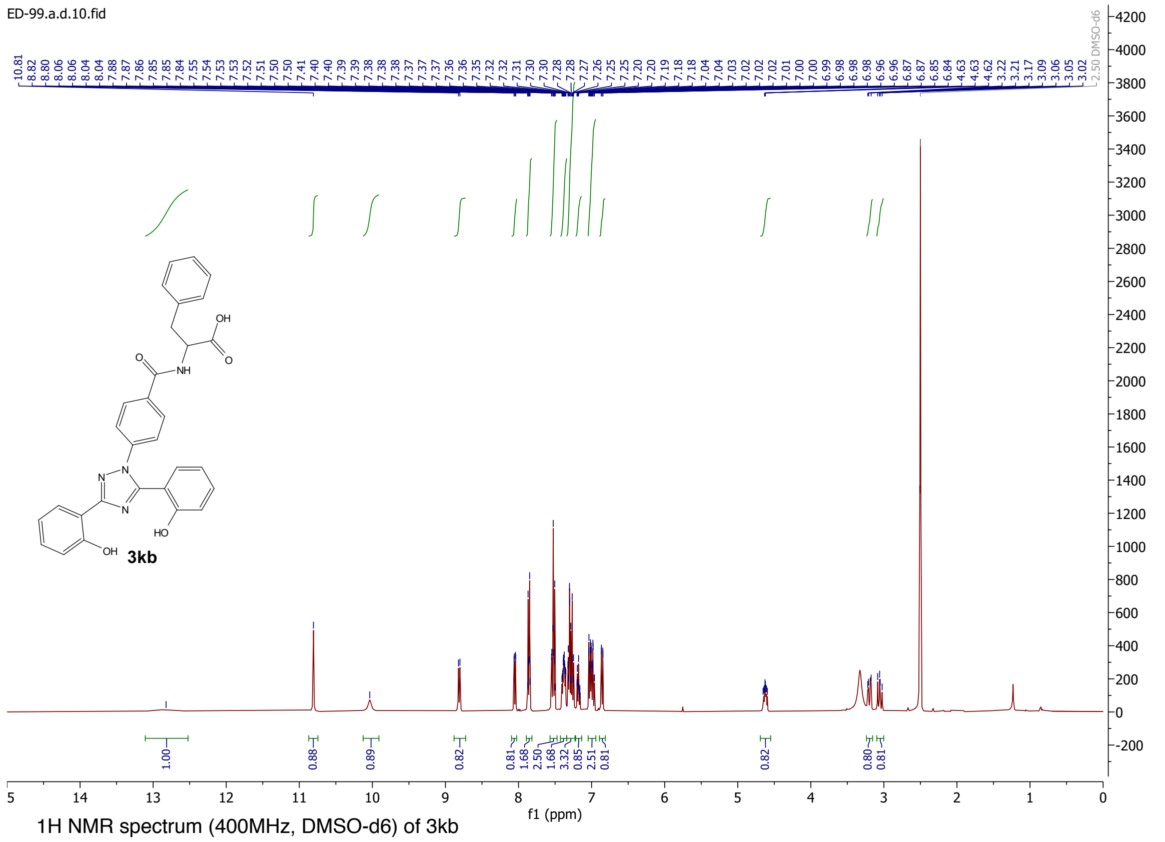

1H NMR spectrum (400MHz, DMSO-d6) of 3kb

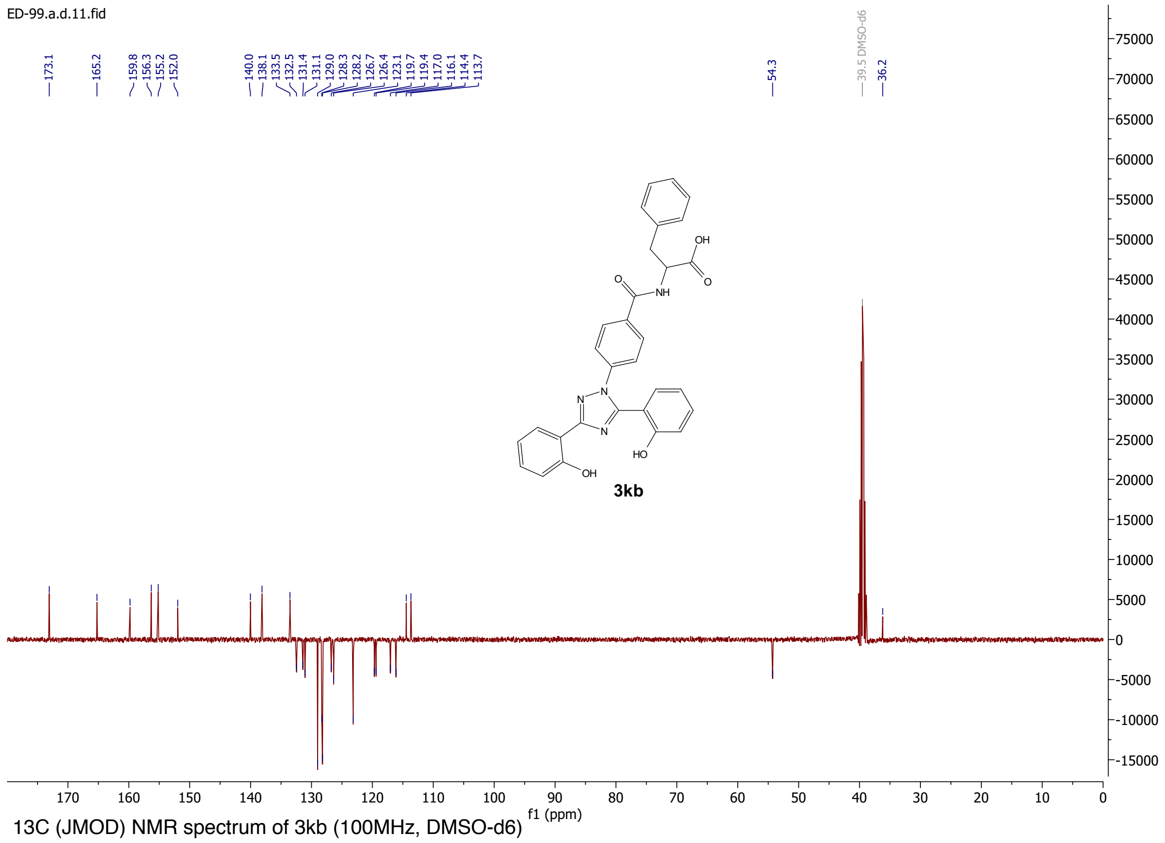

13C (JMOD) NMR spectrum of 3kb (100MHz, DMSO-d6)

Figure S22 : ^{1}H and ^{13}C NMR spectra of 3la

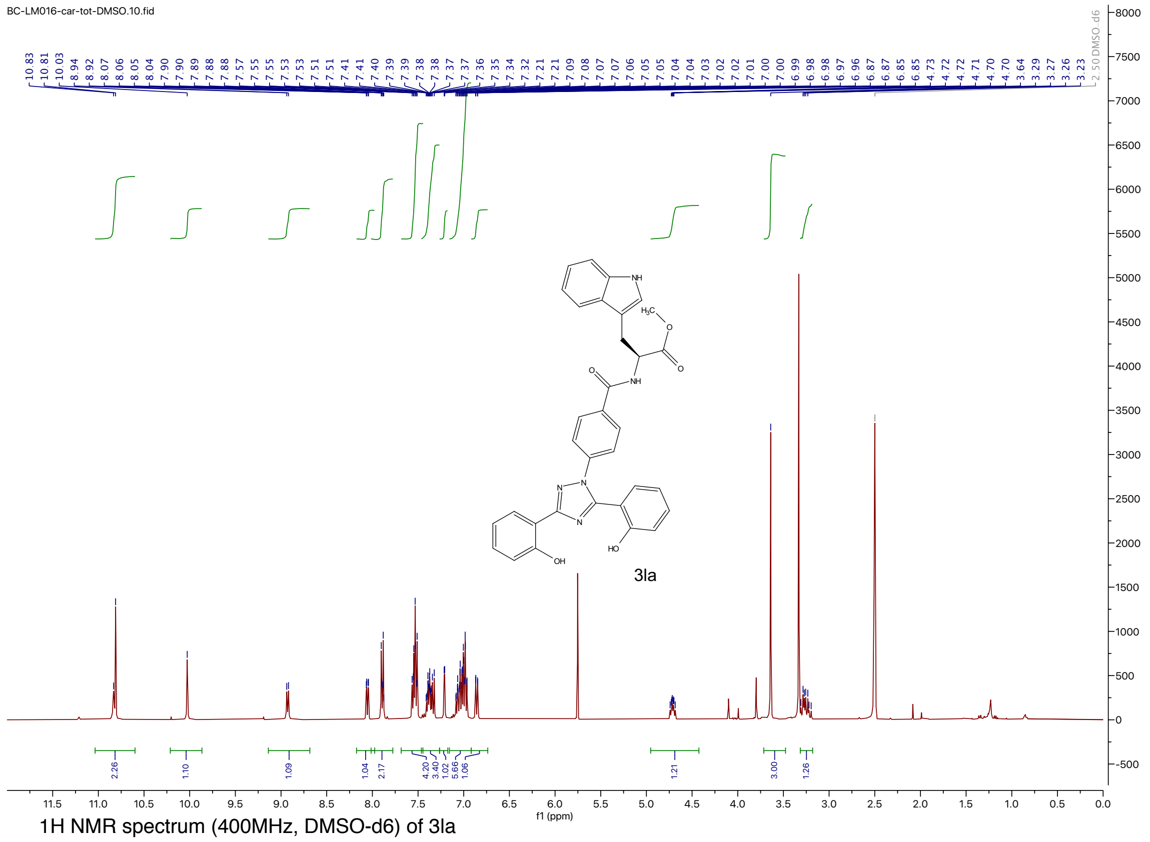

1H NMR spectrum (400MHz, DMSO-d6) of 3la

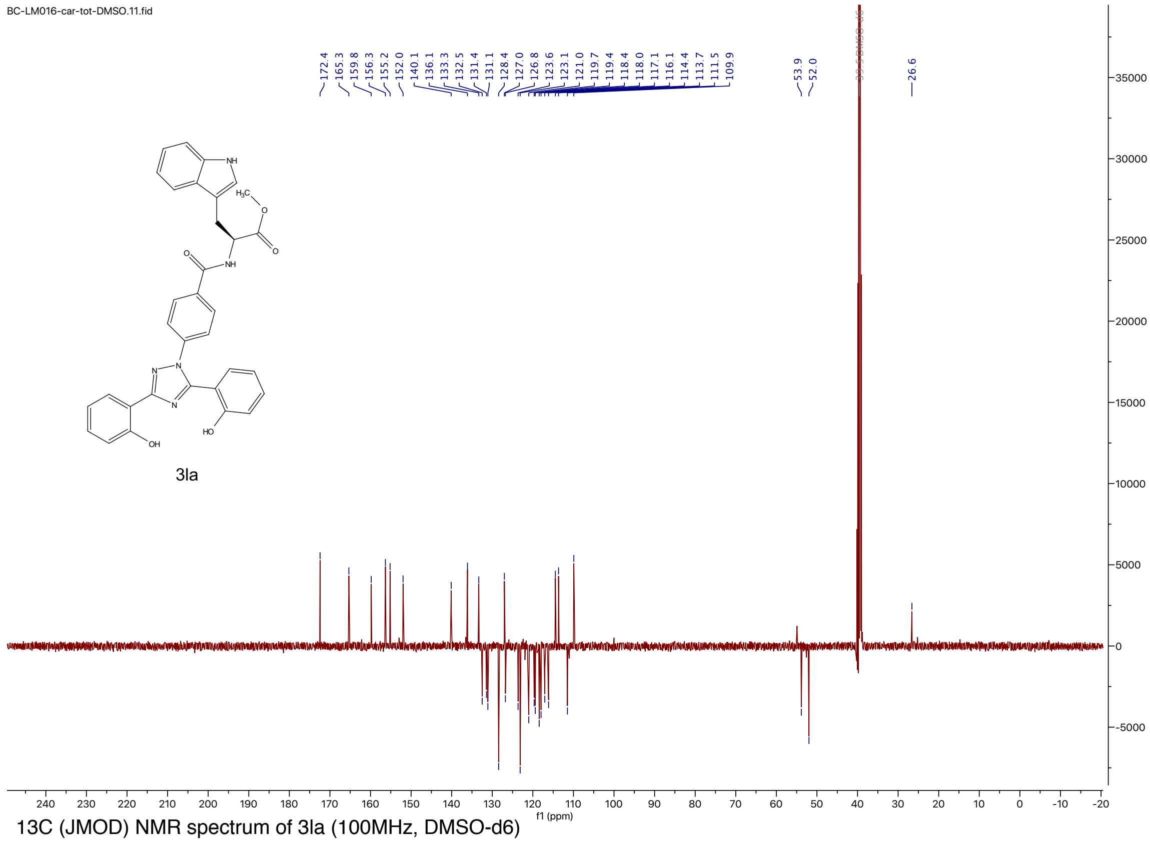

13C (JMOD) NMR spectrum of 3la (100MHz, DMSO-d6)

Figure S23 : ^{1}H and ^{13}C NMR spectra of 3lb

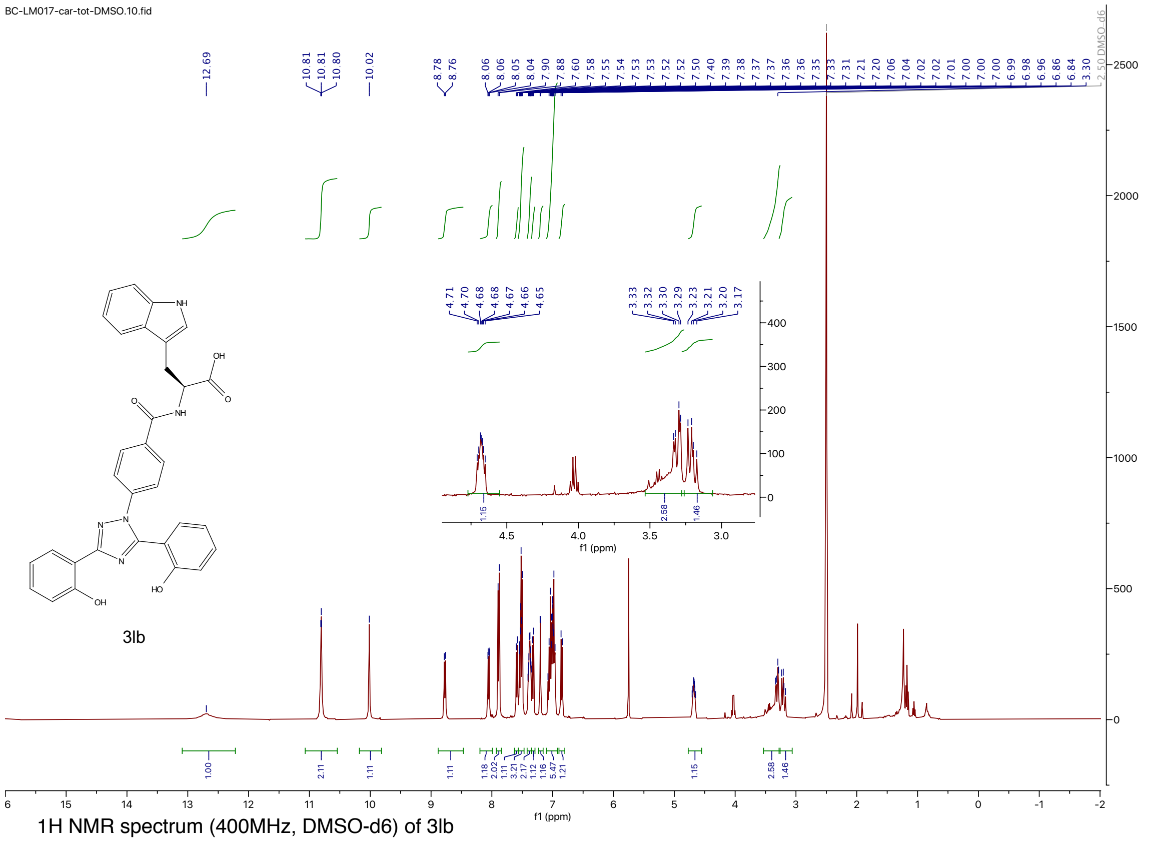

1H NMR spectrum (400MHz, DMSO-d6) of 3lb

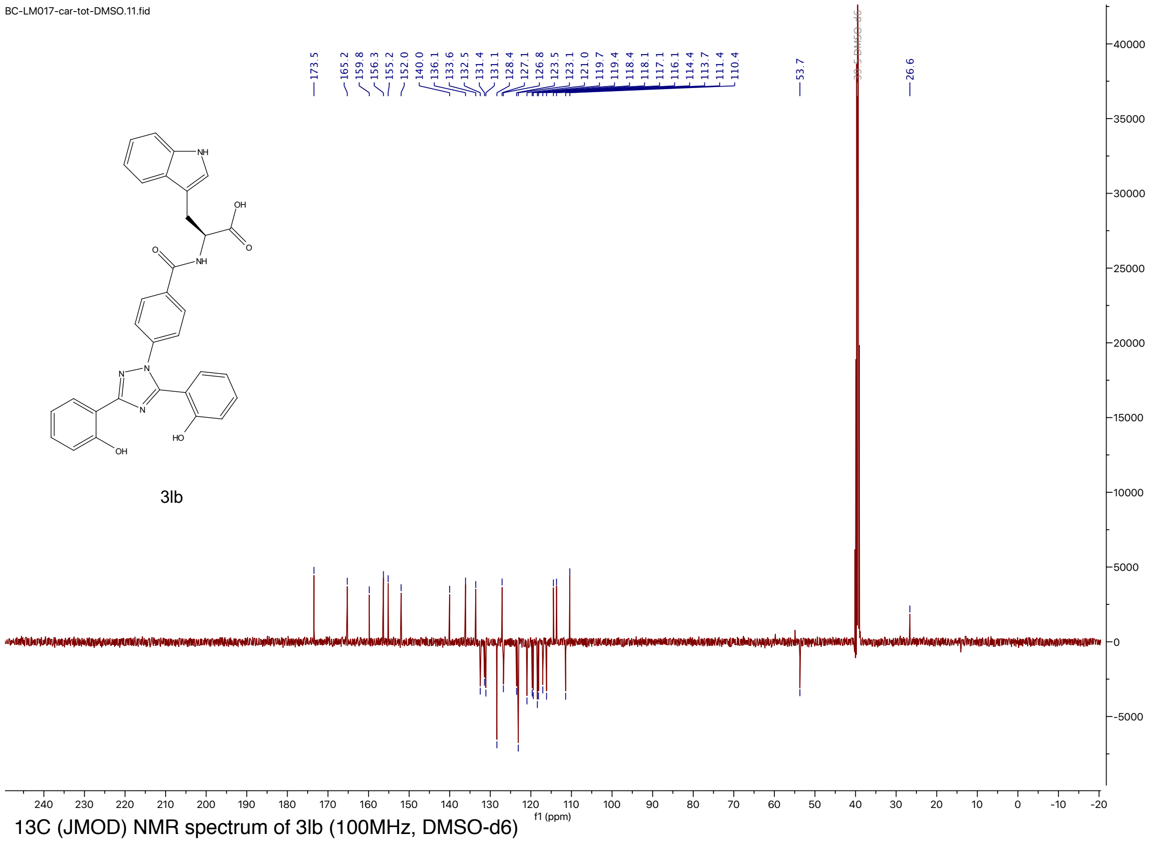

13C (JMOD) NMR spectrum of 3lb (100MHz, DMSO-d6)

Figure S24 : ^{1}H and ^{13}C NMR spectra of 3ma

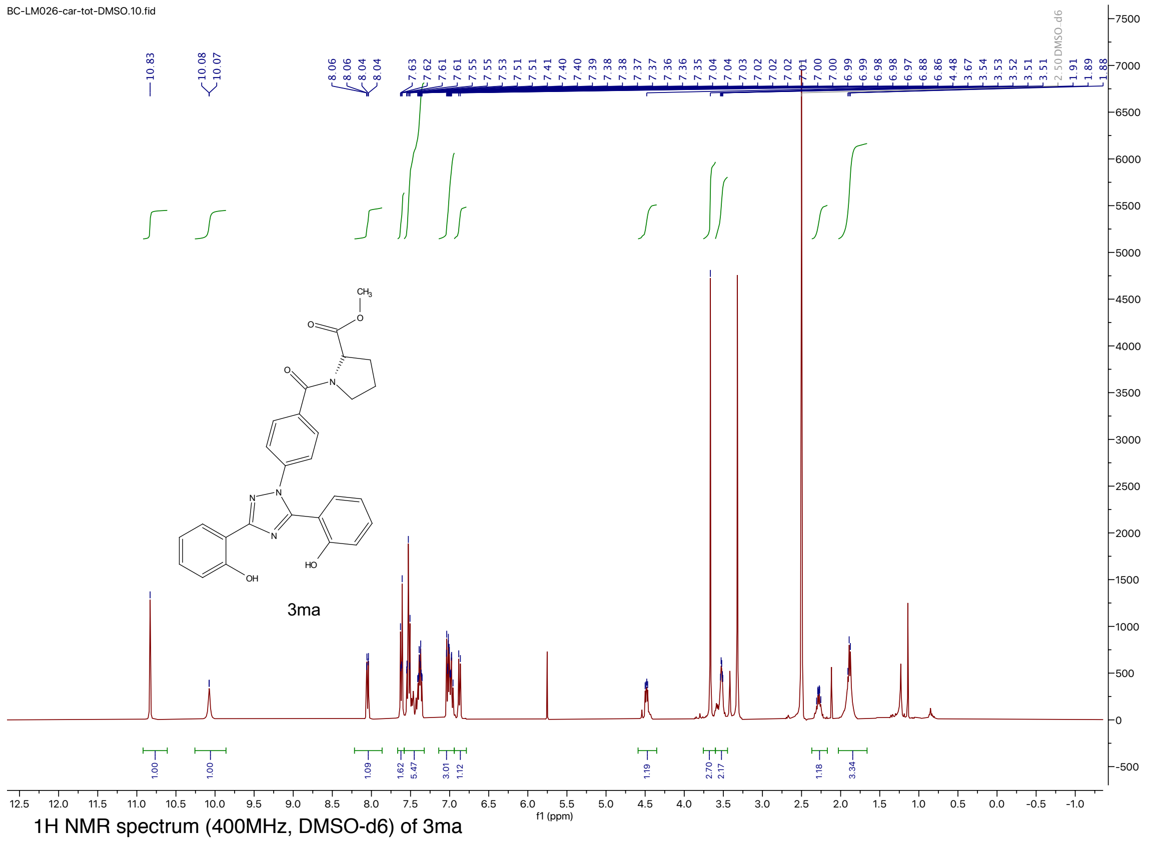

1H NMR spectrum (400MHz, DMSO-d6) of 3ma

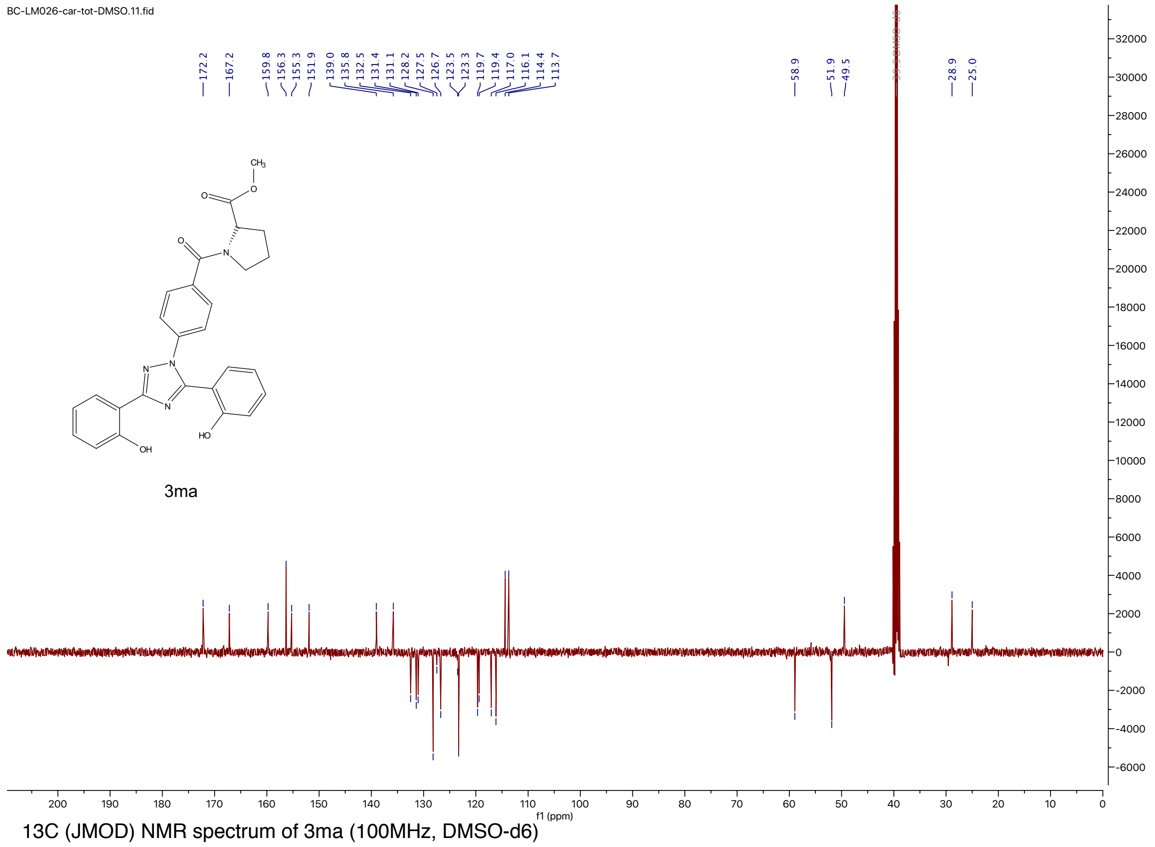

13C (JMOD) NMR spectrum of 3ma (100MHz, DMSO-d6)

Figure S25 : ^{1}H and ^{13}C NMR spectra of 3mb

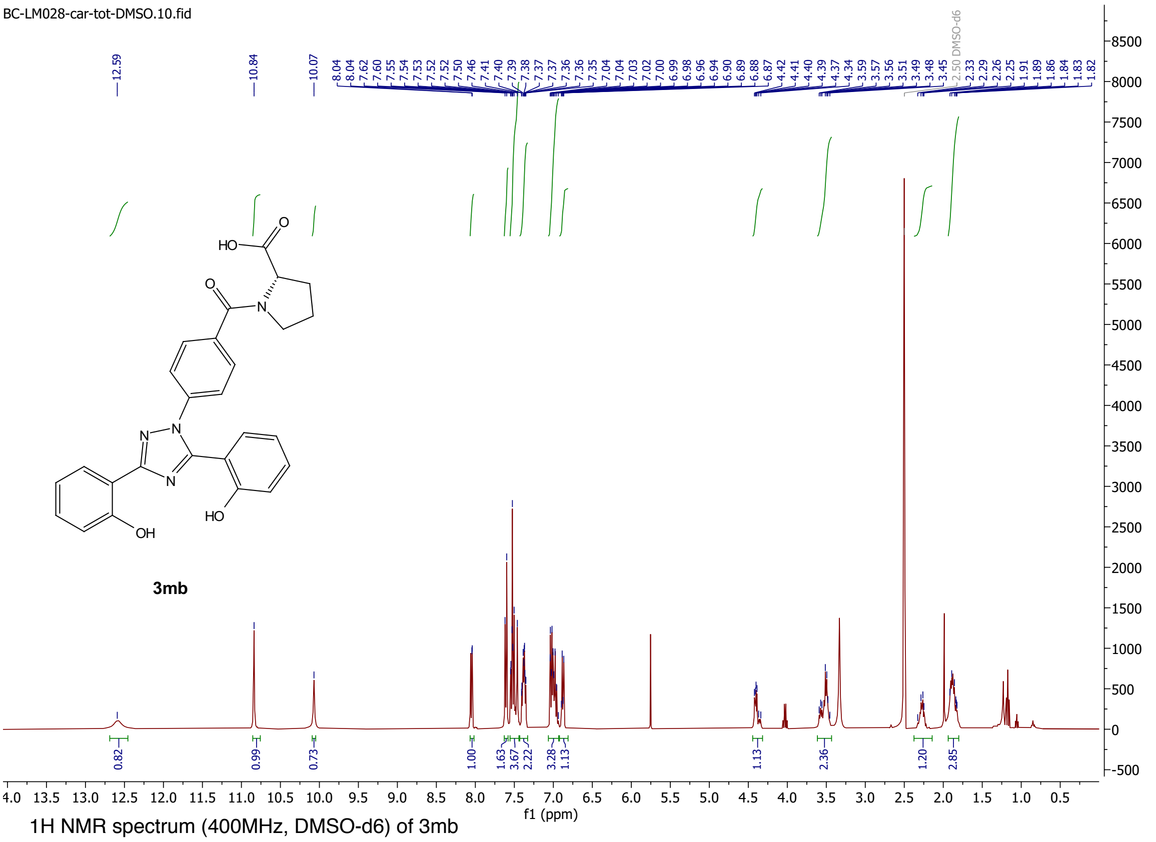

1H NMR spectrum (400MHz, DMSO-d6) of 3mb

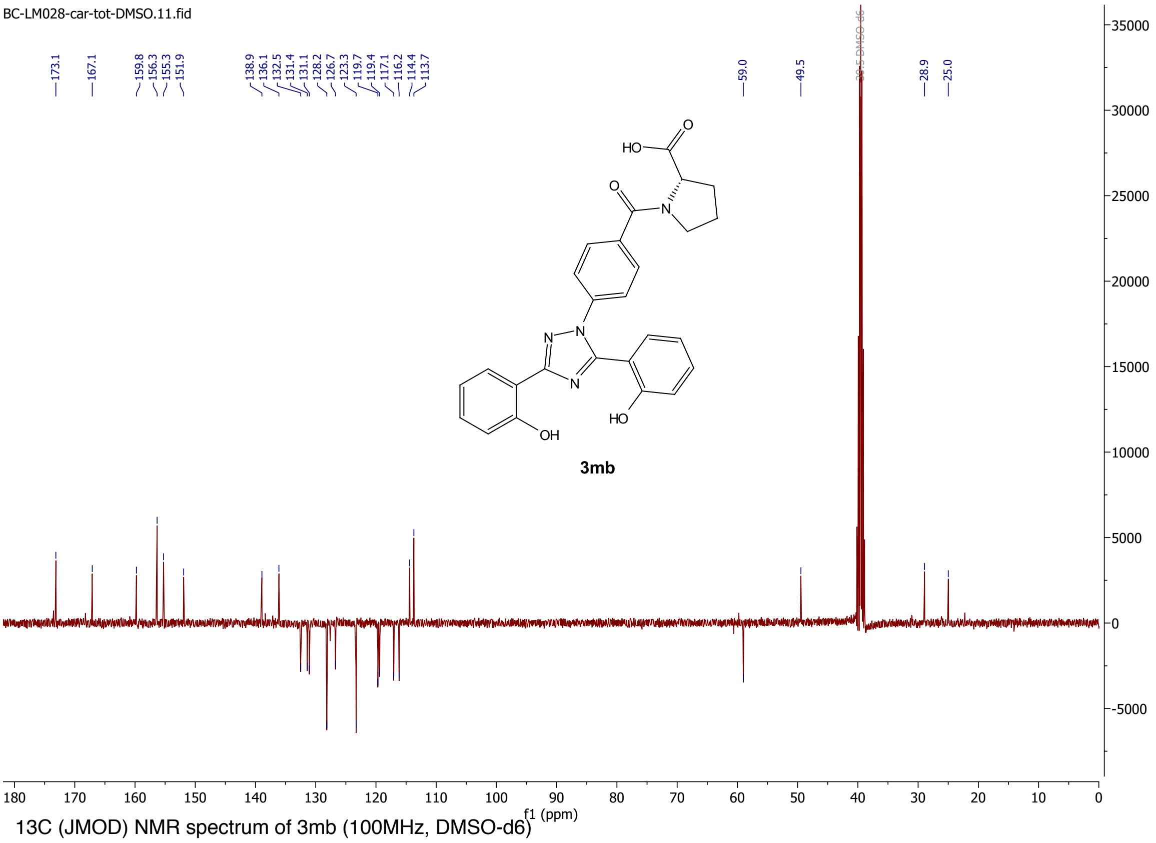

13C (JMOD) NMR spectrum of 3mb (100MHz, DMSO-d6)

Figure S26 : ^{1}H and ^{13}C NMR spectra of 3p

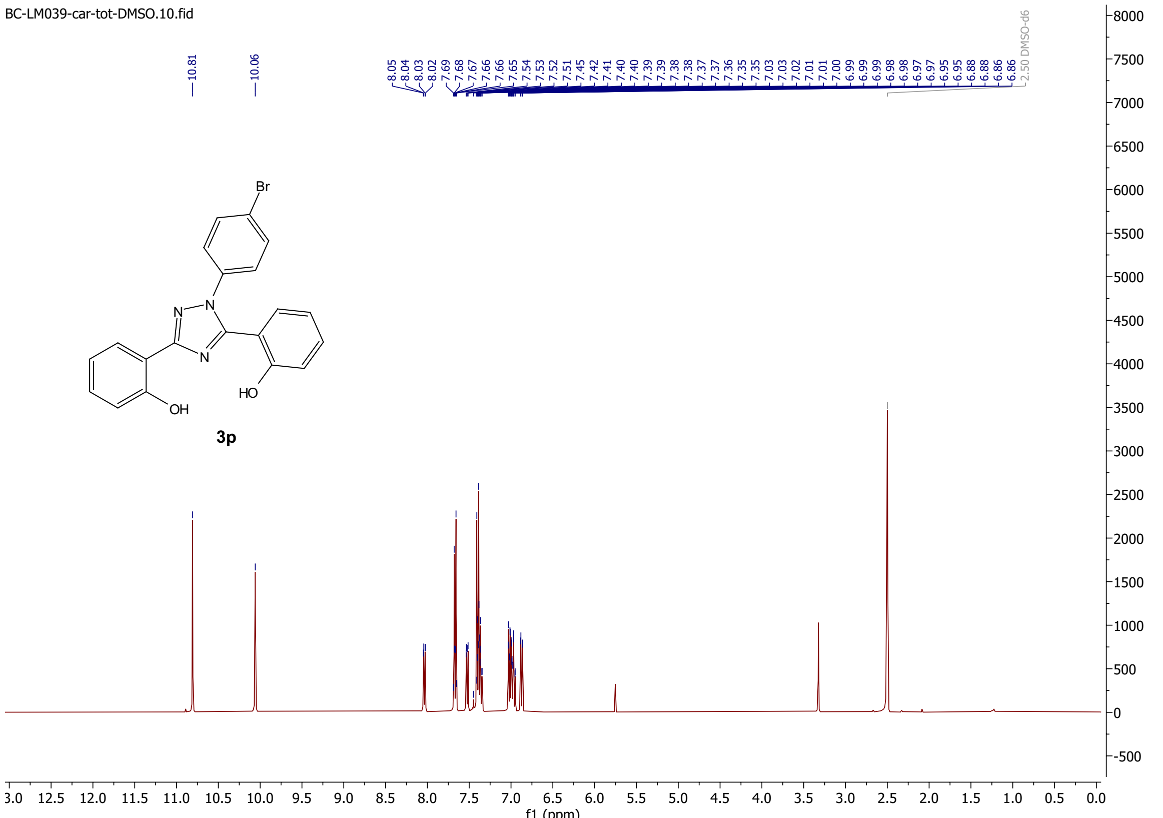

1H NMR spectrum (400MHz, DMSO-d6) of 3p

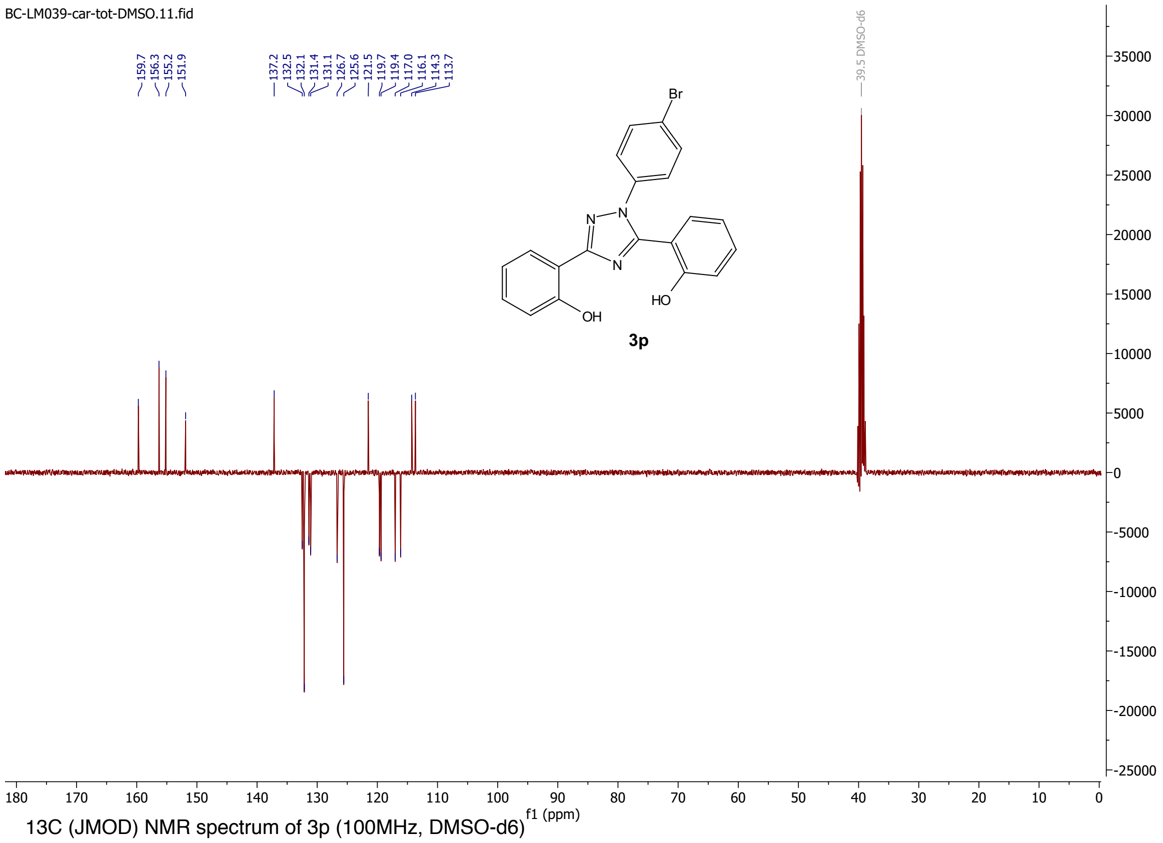

13C (JMOD) NMR spectrum of 3p (100MHz, DMSO-d6)

Figure S27 : ^{1}H and ^{13}C NMR spectra of 3r

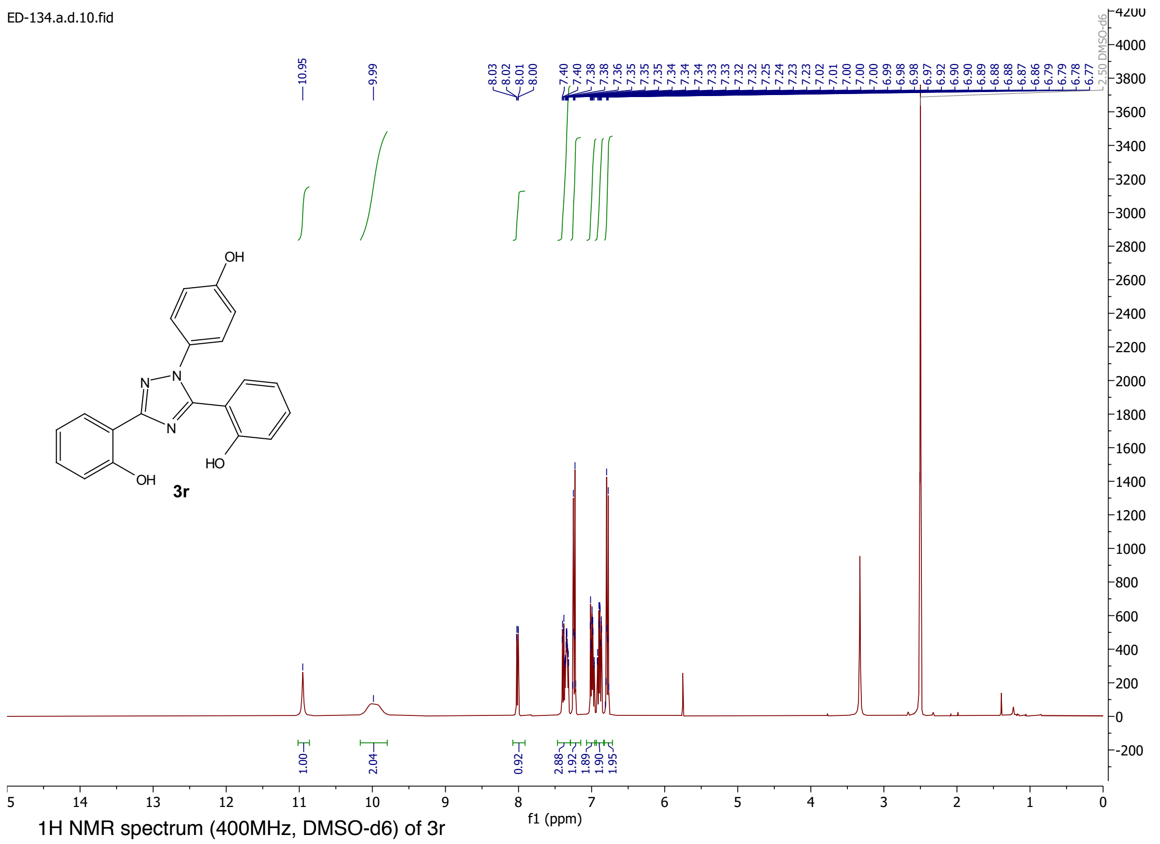

1H NMR spectrum (400MHz, DMSO-d6) of 3r

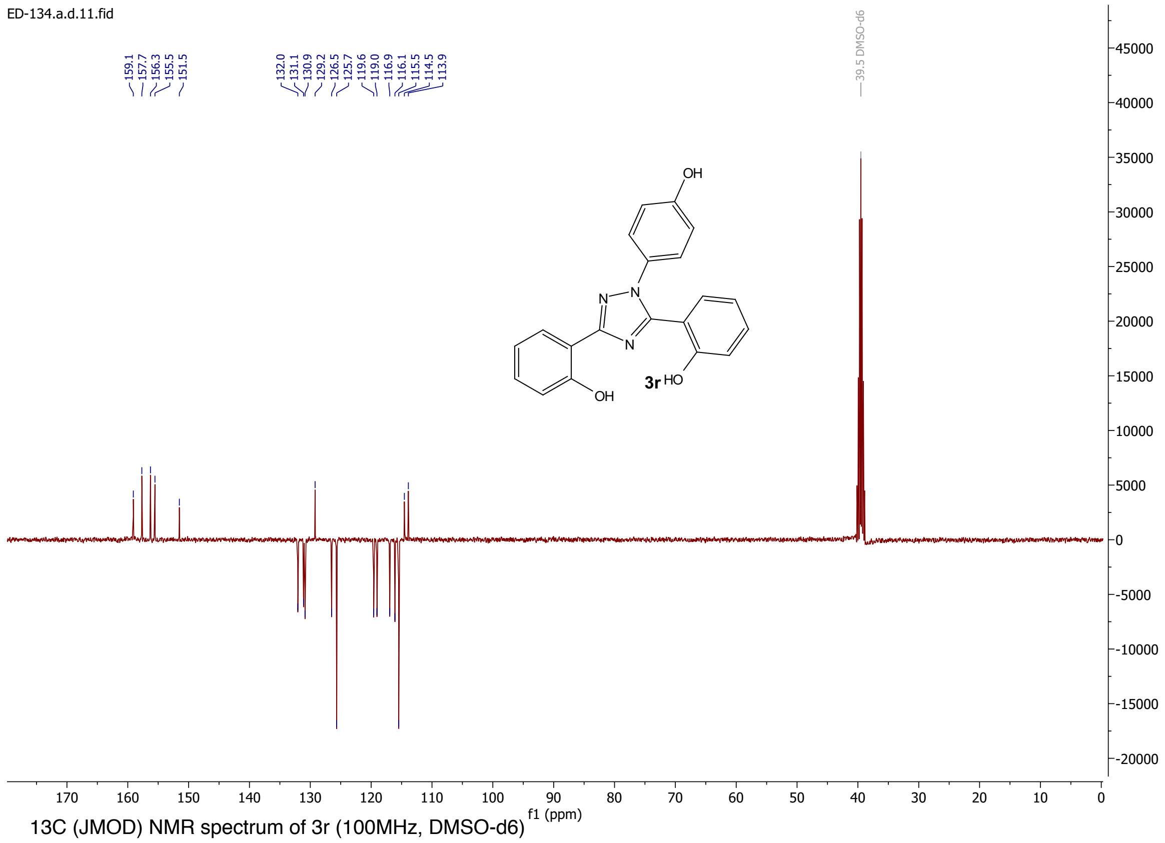

13C (JMOD) NMR spectrum of 3r (100MHz, DMSO-d6)

Figure S28 : ^{1}H and ^{13}C NMR spectra of 3v

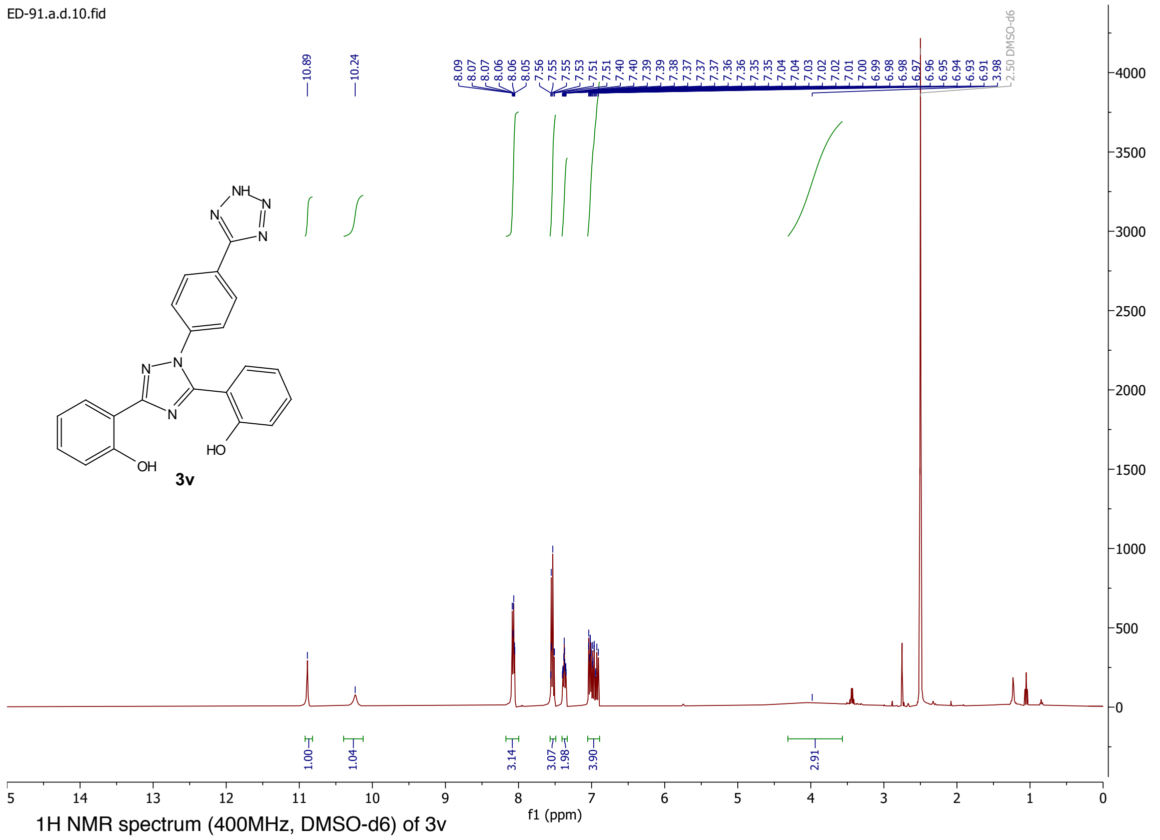

1H NMR spectrum (400MHz, DMSO-d6) of 3v

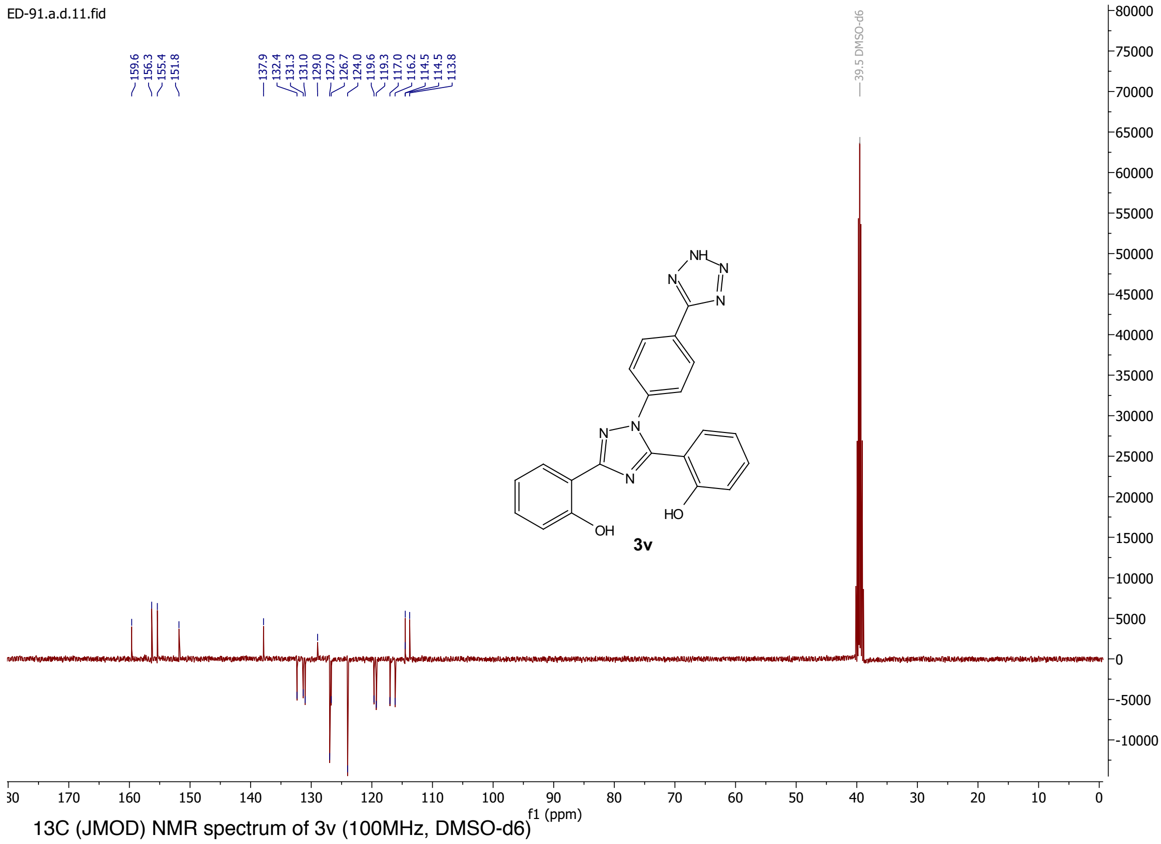

13C (JMOD) NMR spectrum of 3v (100MHz, DMSO-d6)

Figure S29 : ^{1}H and ^{13}C NMR spectra of (S)-methyl 2-amino-5-(3-tosylguanidino)pentanoate

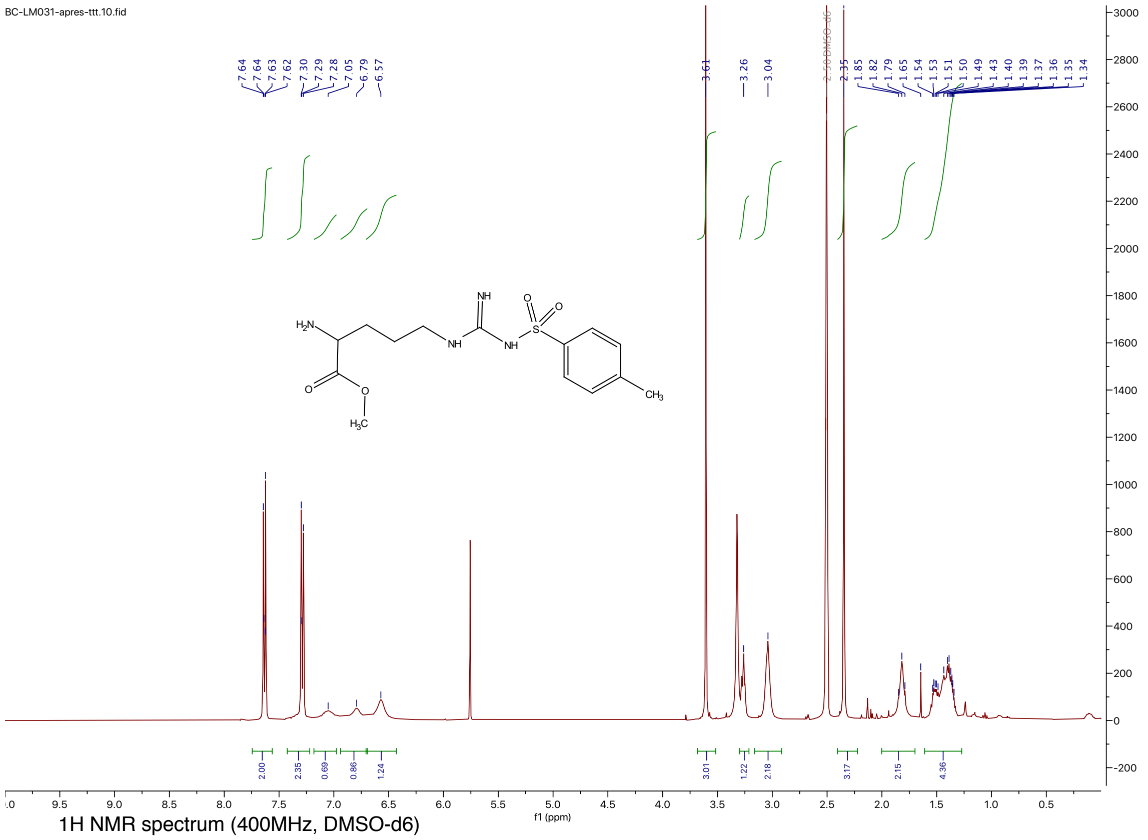

1H NMR spectrum (400MHz, DMSO-d6)

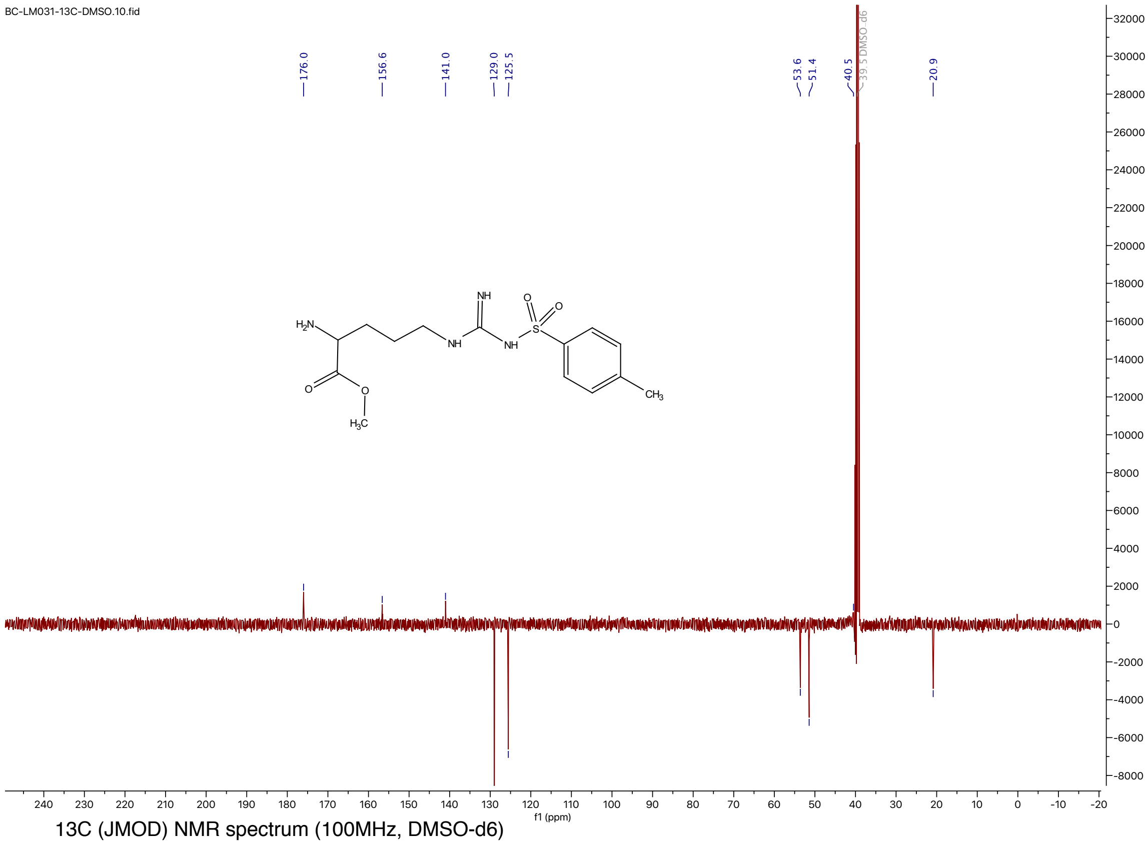

13C (JMOD) NMR spectrum (100MHz, DMSO-d6)

Figure S30 : ^{1}H and ^{13}C NMR spectra of 3w

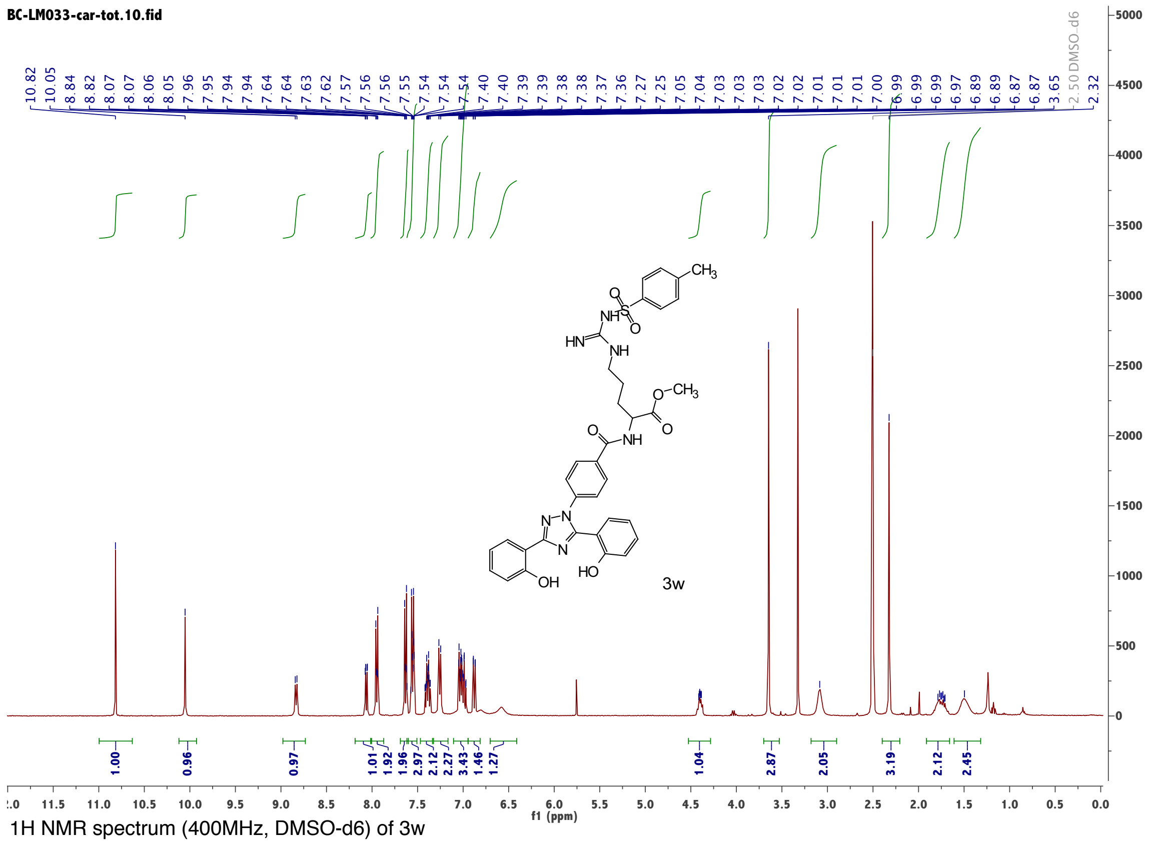

1H NMR spectrum (400MHz, DMSO-d6) of 3w

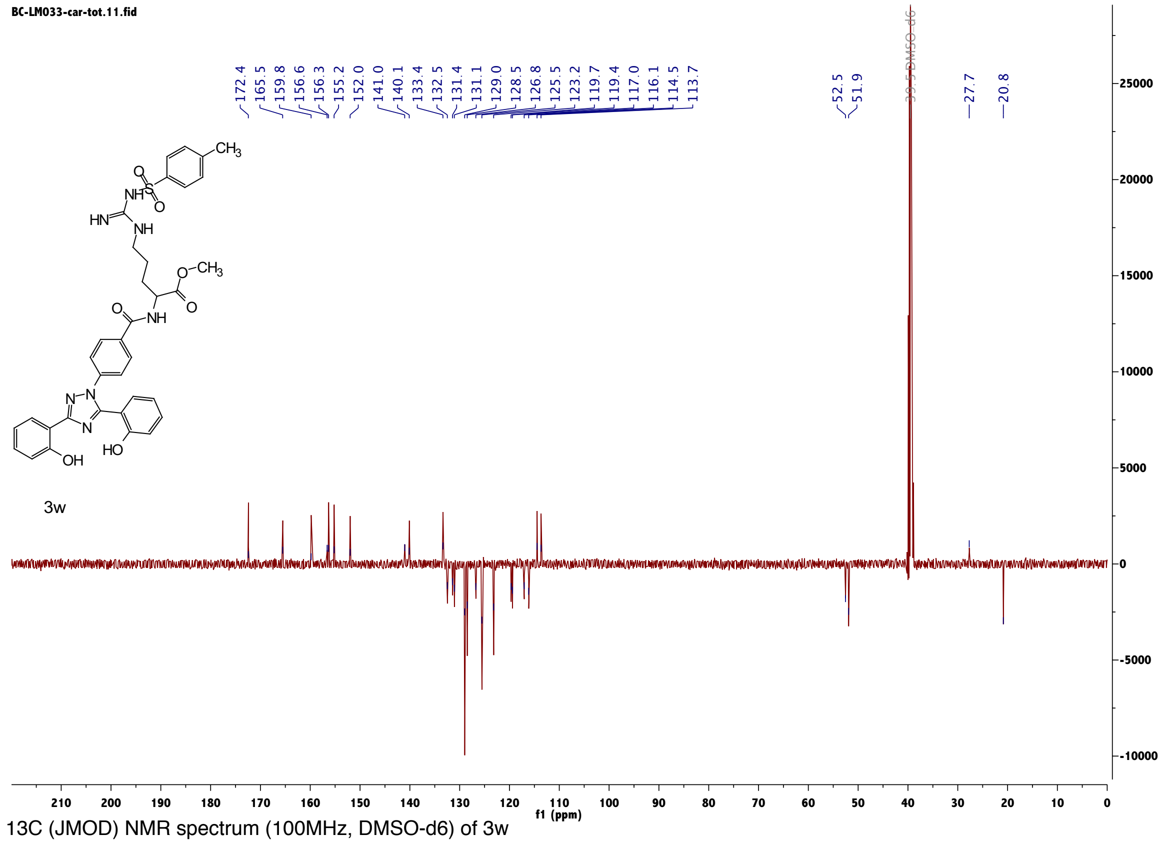

13C (JMOD) NMR spectrum (100MHz, DMSO-d6) of 3w

Figure S31 : ^{1}H and ^{13}C NMR spectra of 6

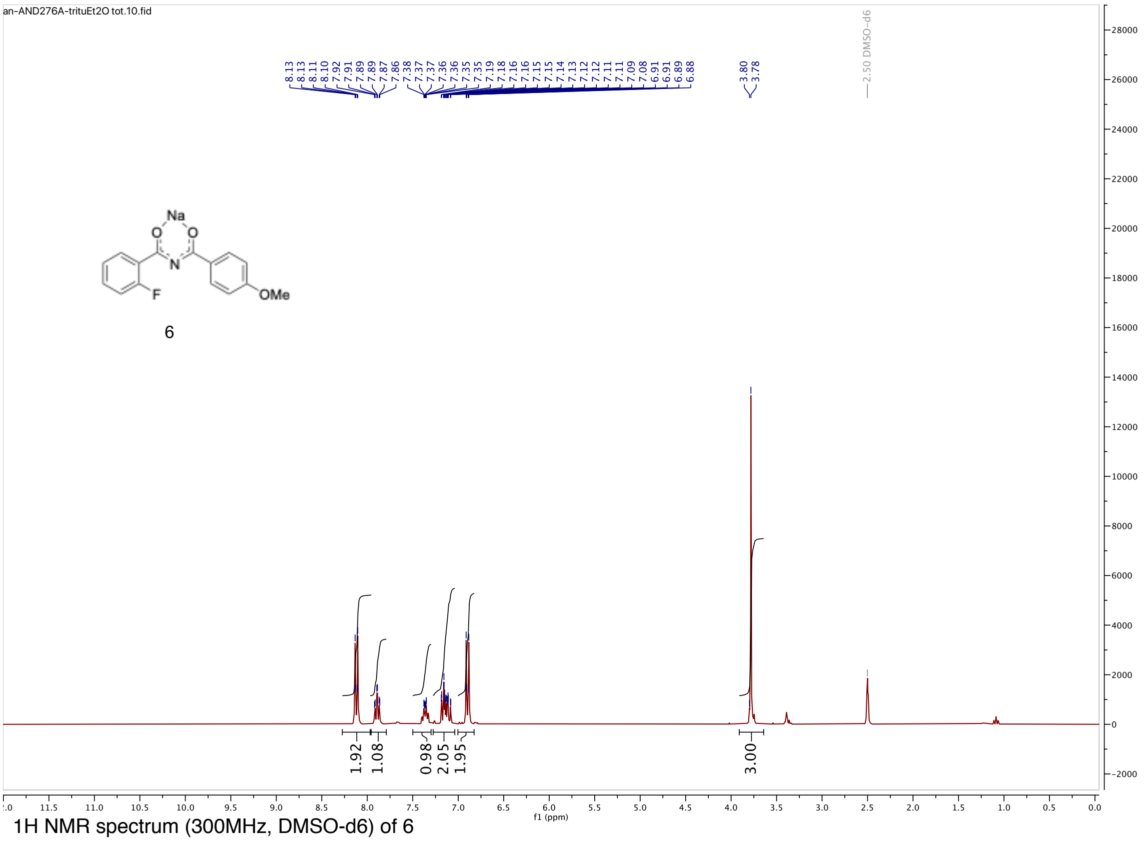

1H NMR spectrum (300MHz, DMSO-d6) of 6

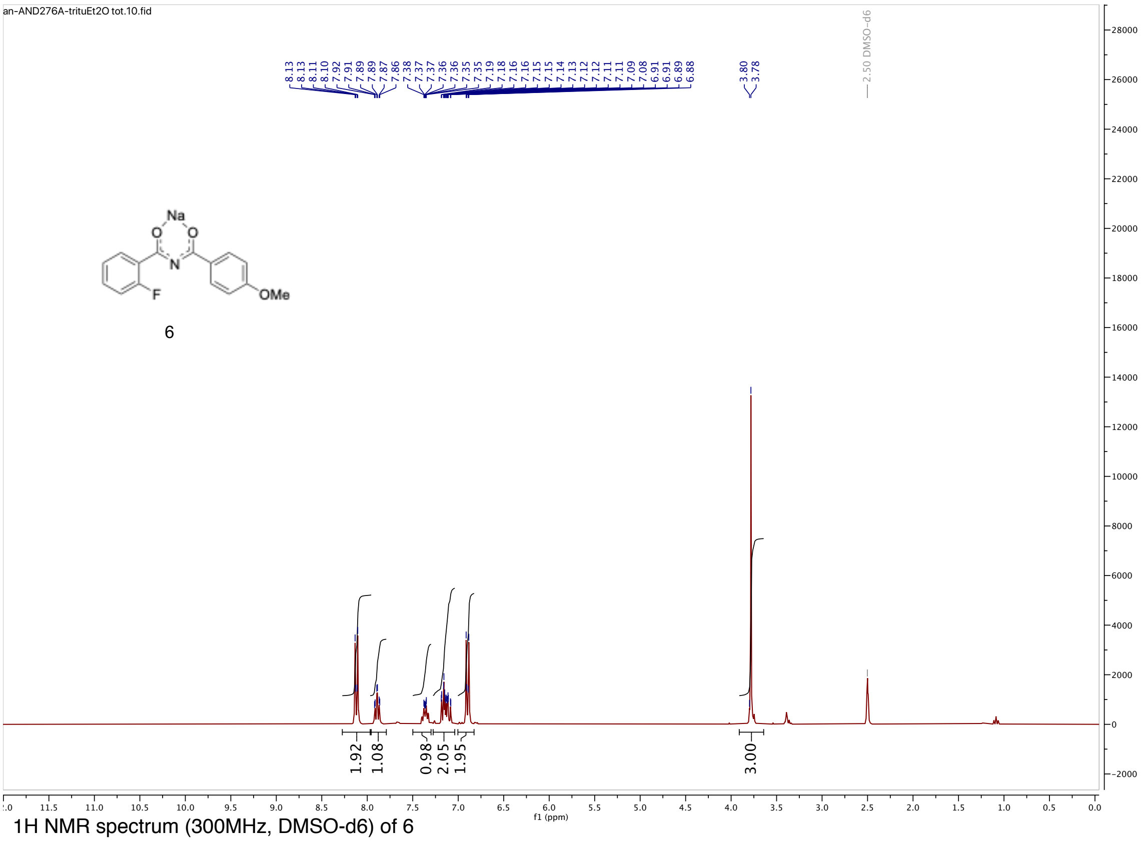

1H NMR spectrum (300MHz, DMSO-d6) of 6

Figure S32 : ^{1}H and ^{13}C NMR spectra of 8

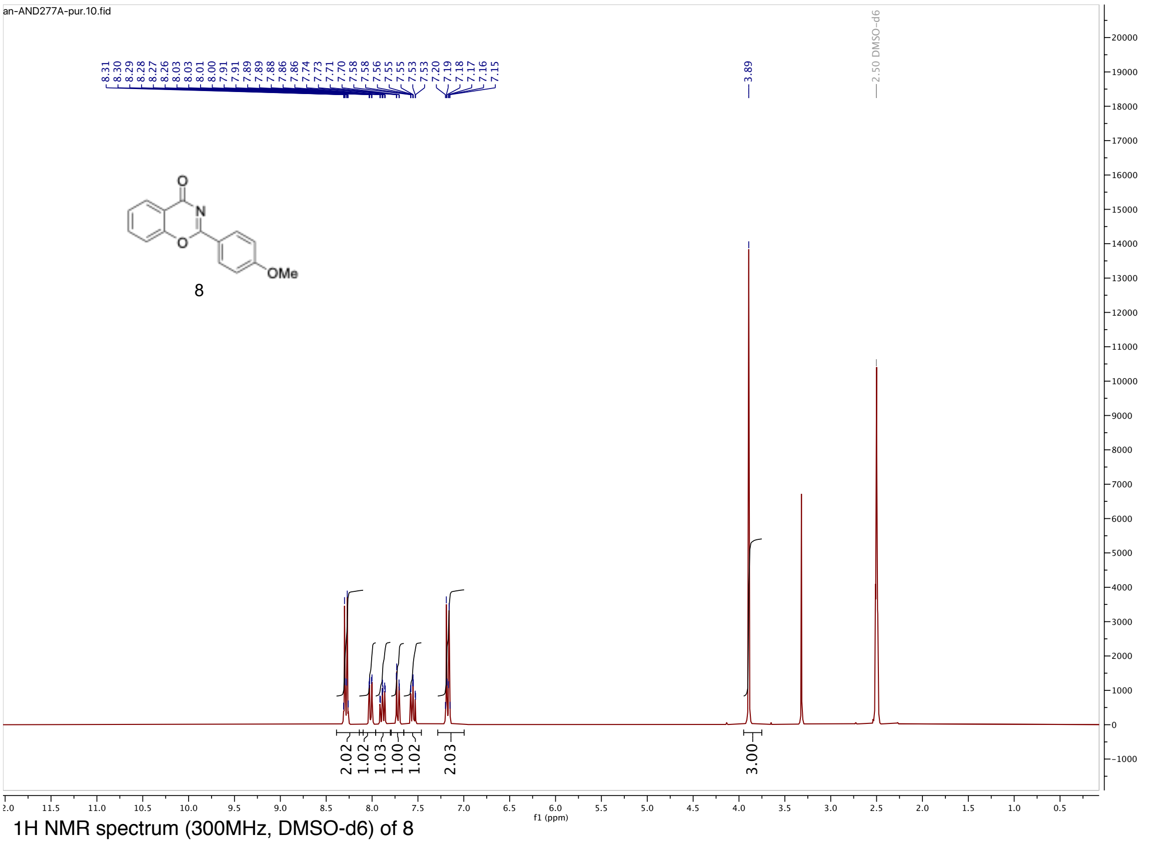

1H NMR spectrum (300MHz, DMSO-d6) of 8

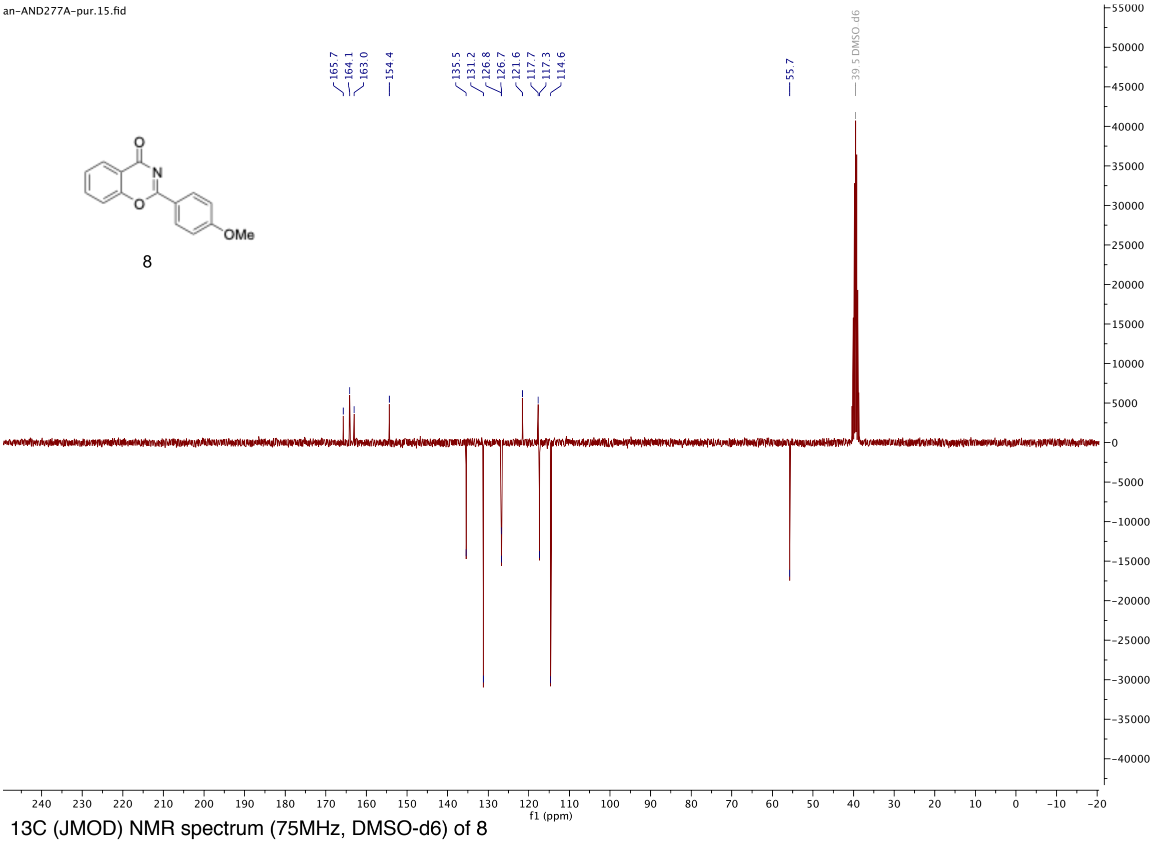

13C (JMOD) NMR spectrum (75MHz, DMSO-d6) of 8

Figure S33 : ^{1}H and ^{13}C NMR spectra of 9

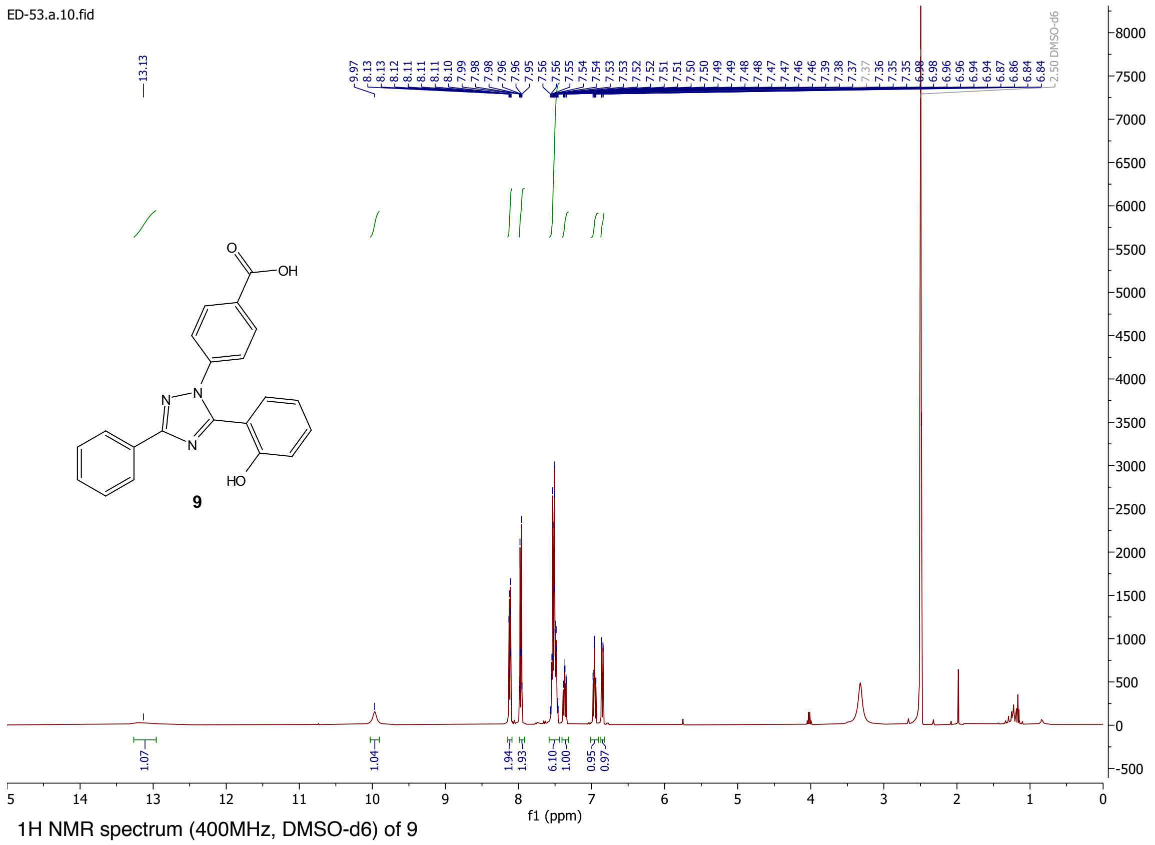

1H NMR spectrum (400MHz, DMSO-d6) of 9

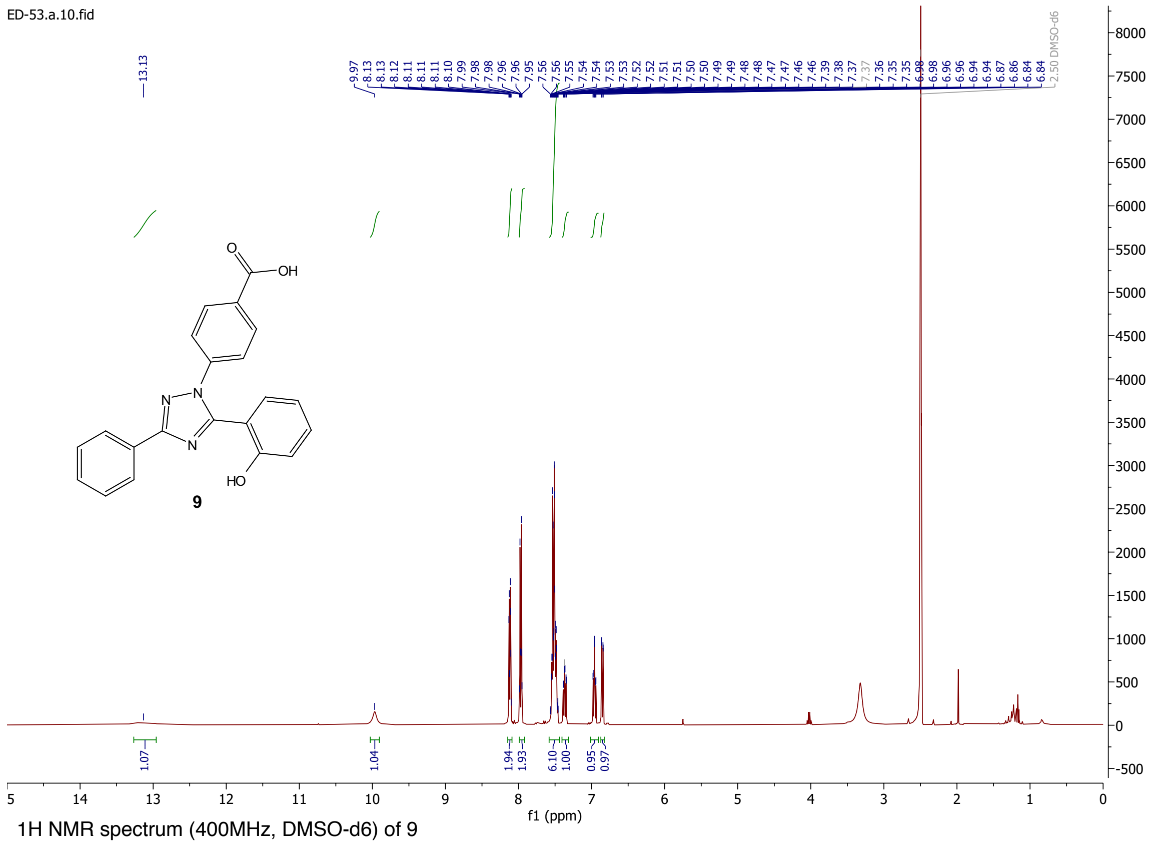

1H NMR spectrum (400MHz, DMSO-d6) of 9

Figure S34 : ^{1}H and ^{13}C NMR spectra of 10

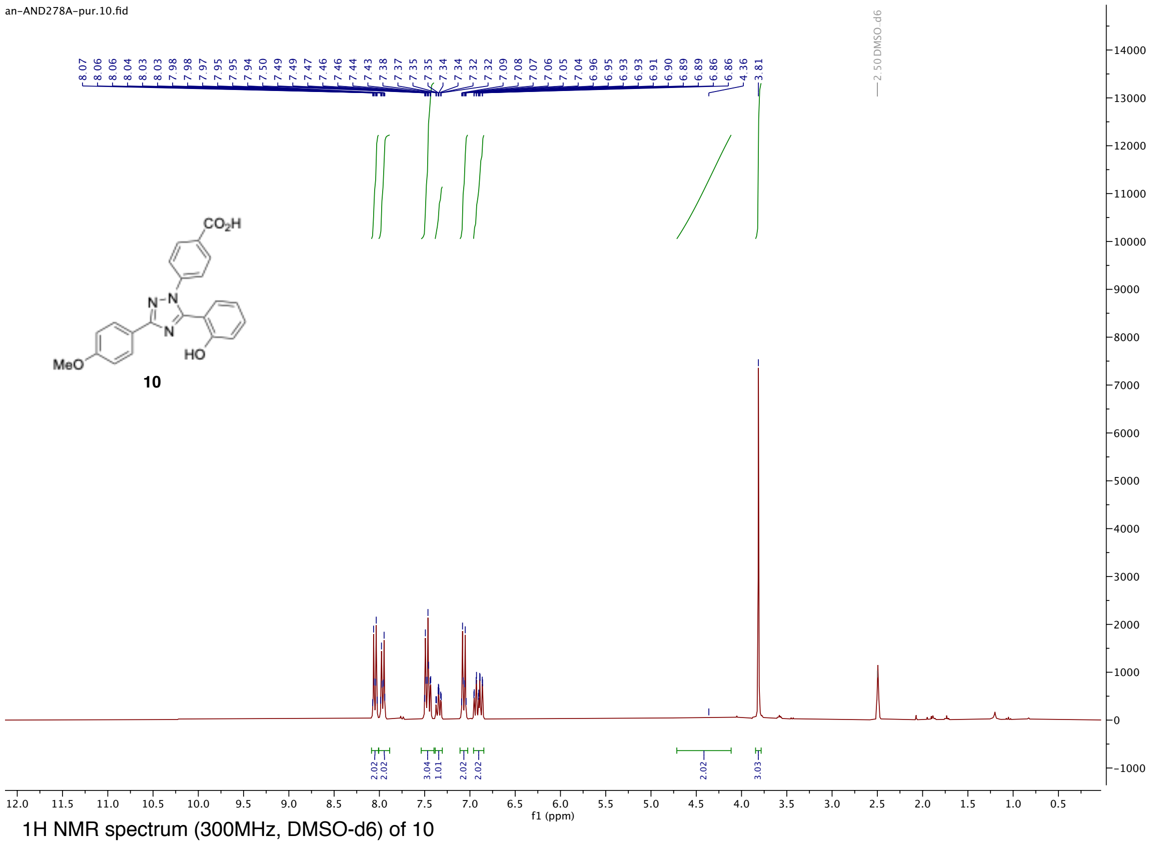

1H NMR spectrum (300MHz, DMSO-d6) of 10

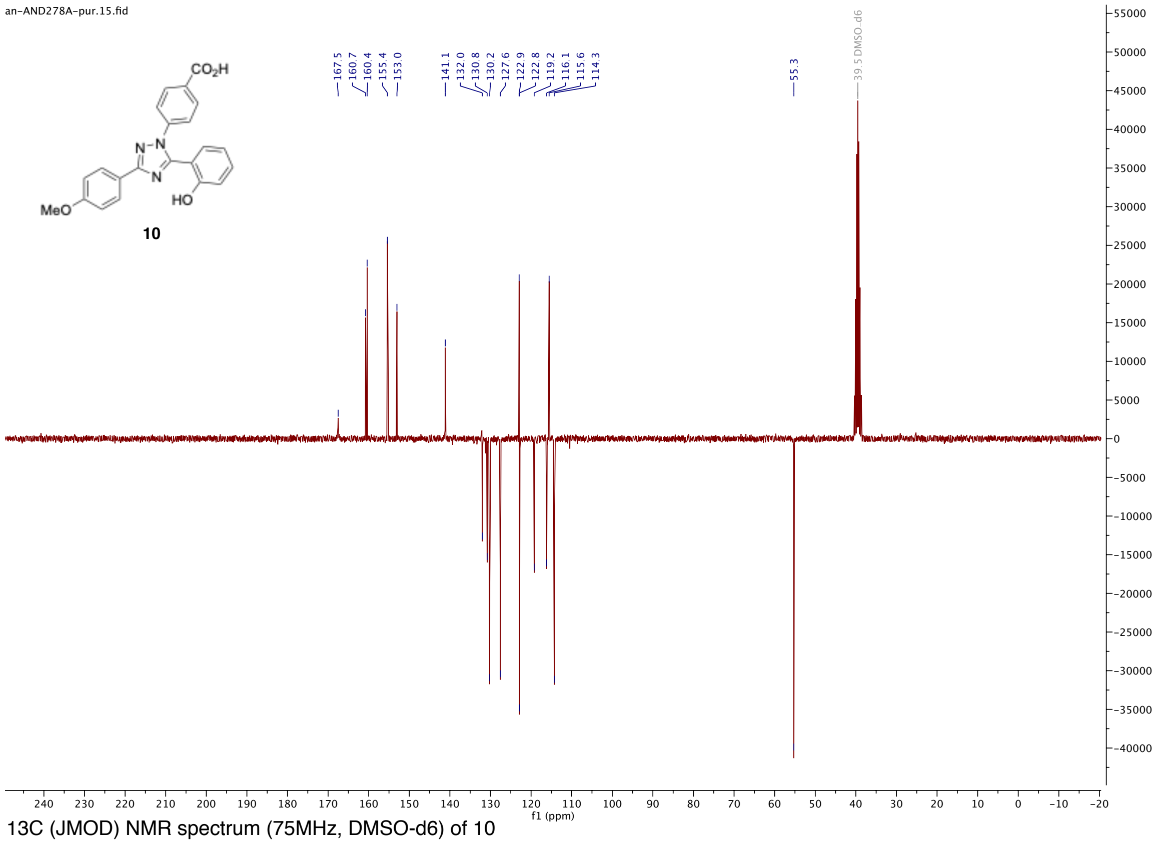

13C (JMOD) NMR spectrum (75MHz, DMSO-d6) of 10

Figure S35 : ^{1}H and ^{13}C NMR spectra of 13

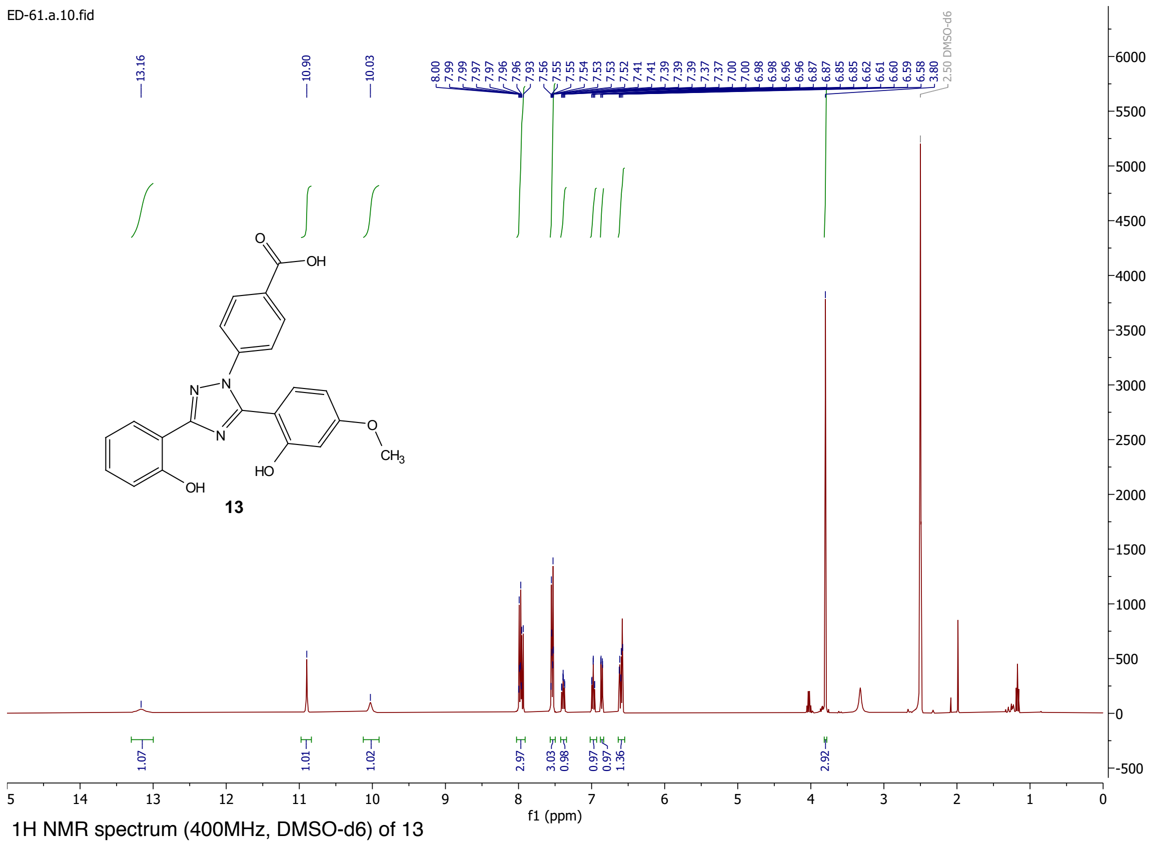

1H NMR spectrum (400MHz, DMSO-d6) of 13

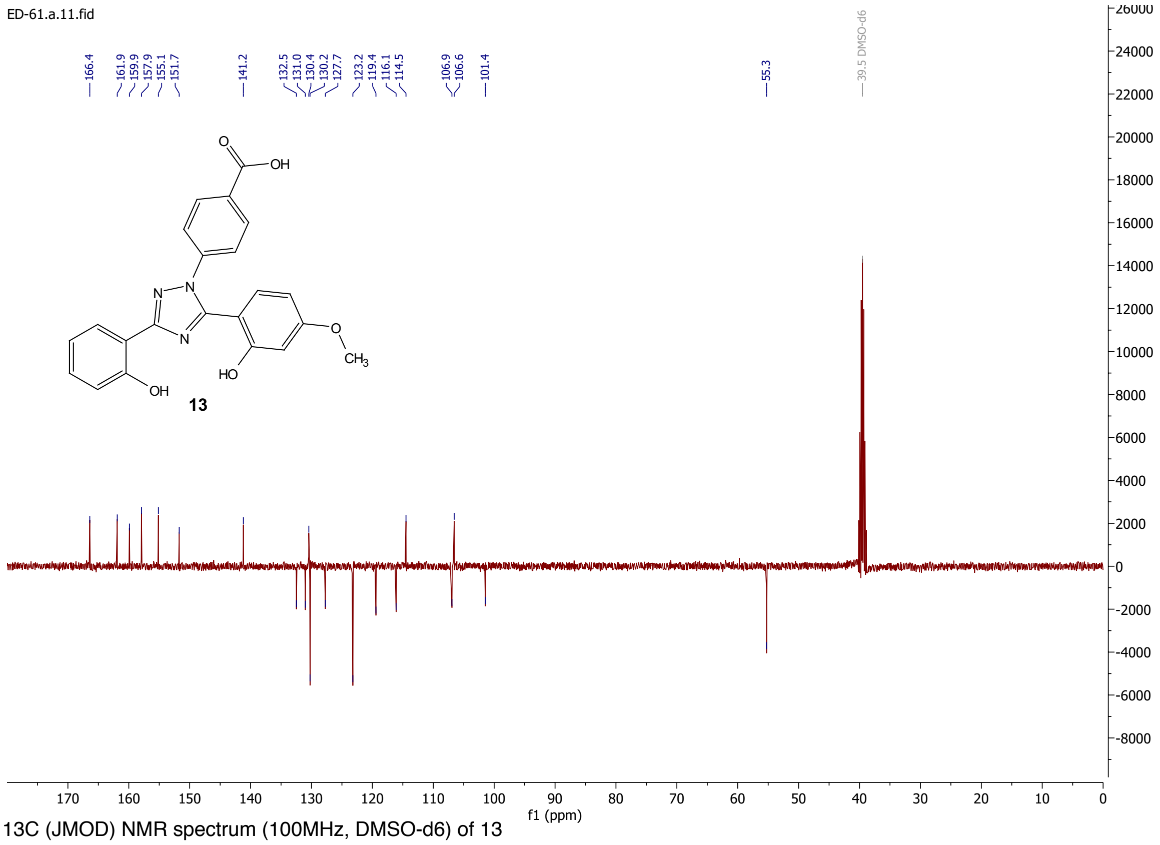

13C (JMOD) NMR spectrum (100MHz, DMSO-d6) of 13

Figure S36 : ^{1}H and ^{13}C NMR spectra of 14

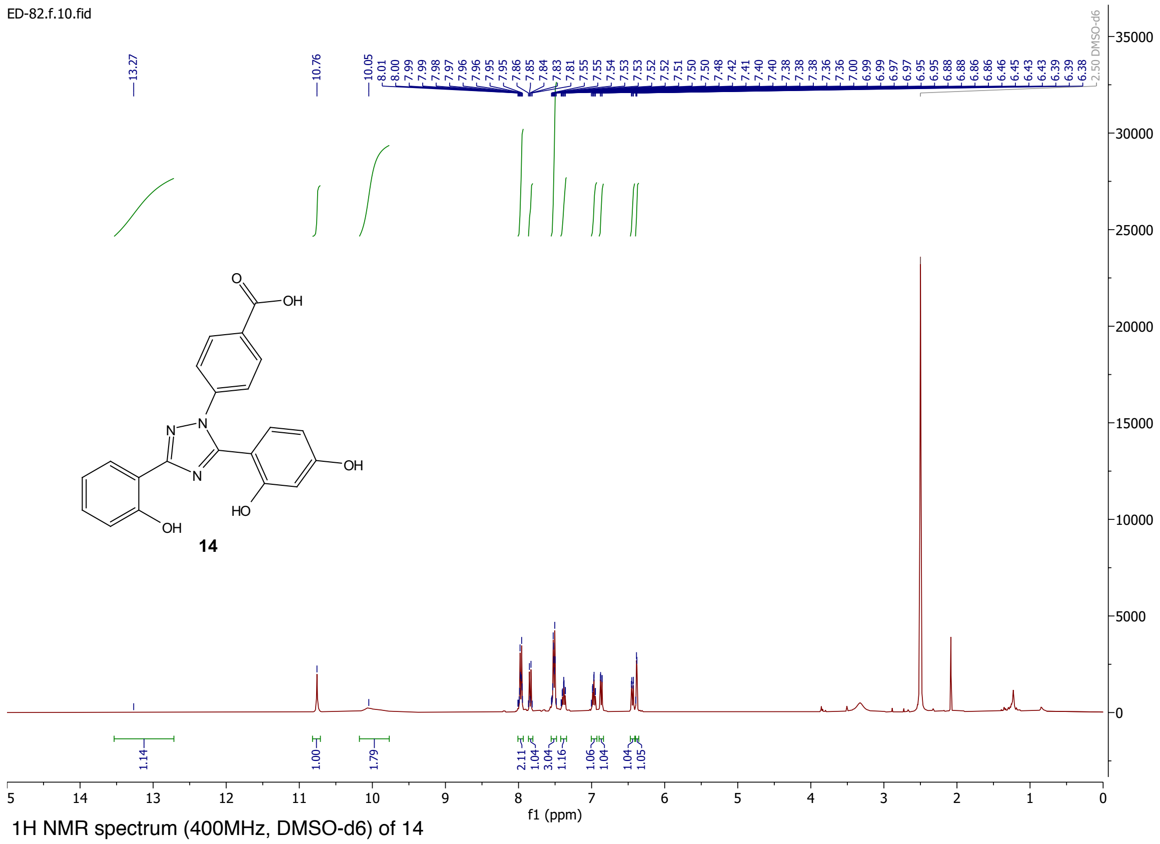

1H NMR spectrum (400MHz, DMSO-d6) of 14

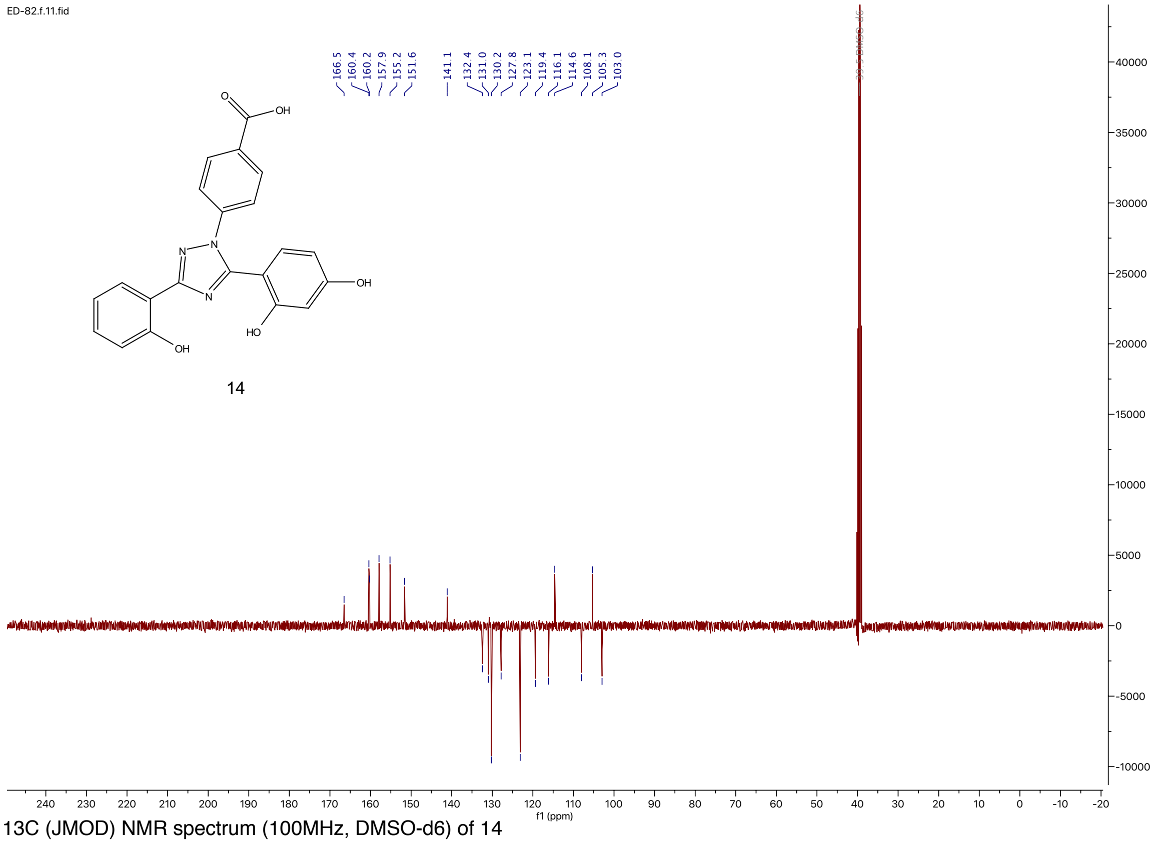

13C (JMOD) NMR spectrum (100MHz, DMSO-d6) of 14

Figure S37 : ^{1}H and ^{13}C NMR spectra of 20

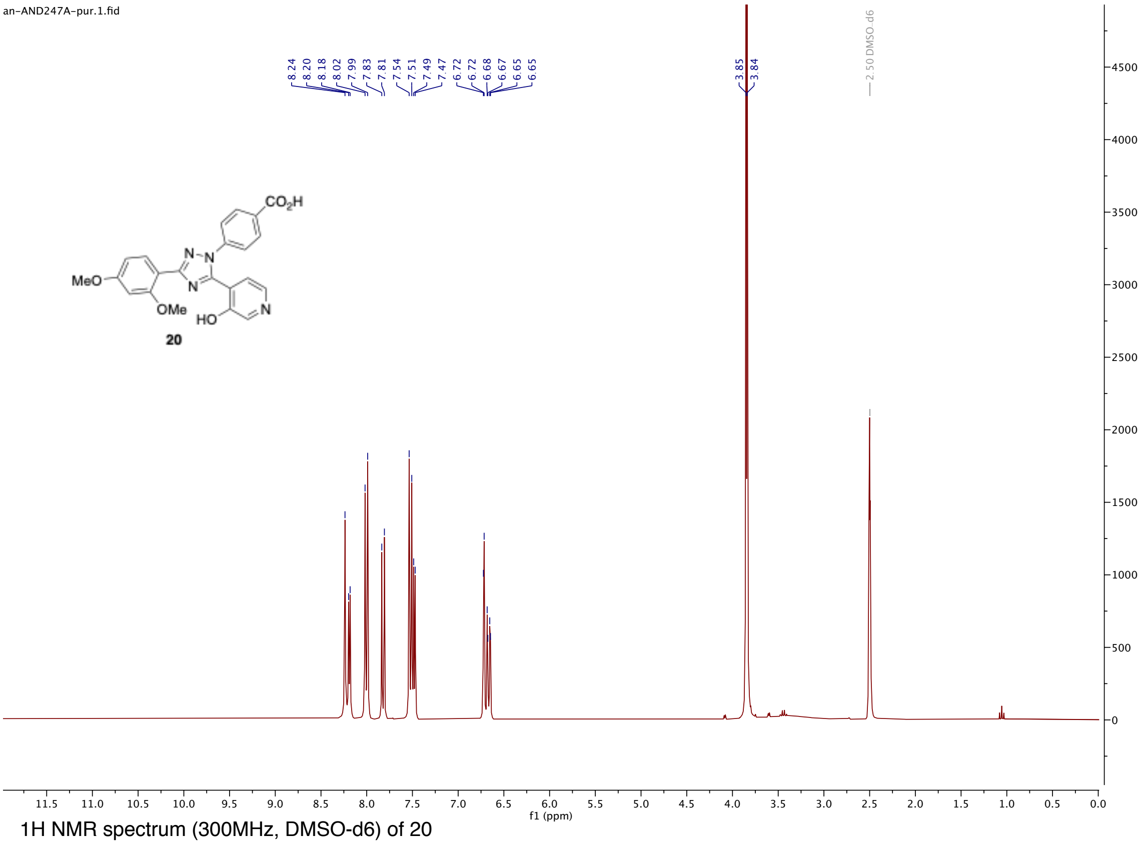

1H NMR spectrum (300MHz, DMSO-d6) of 20

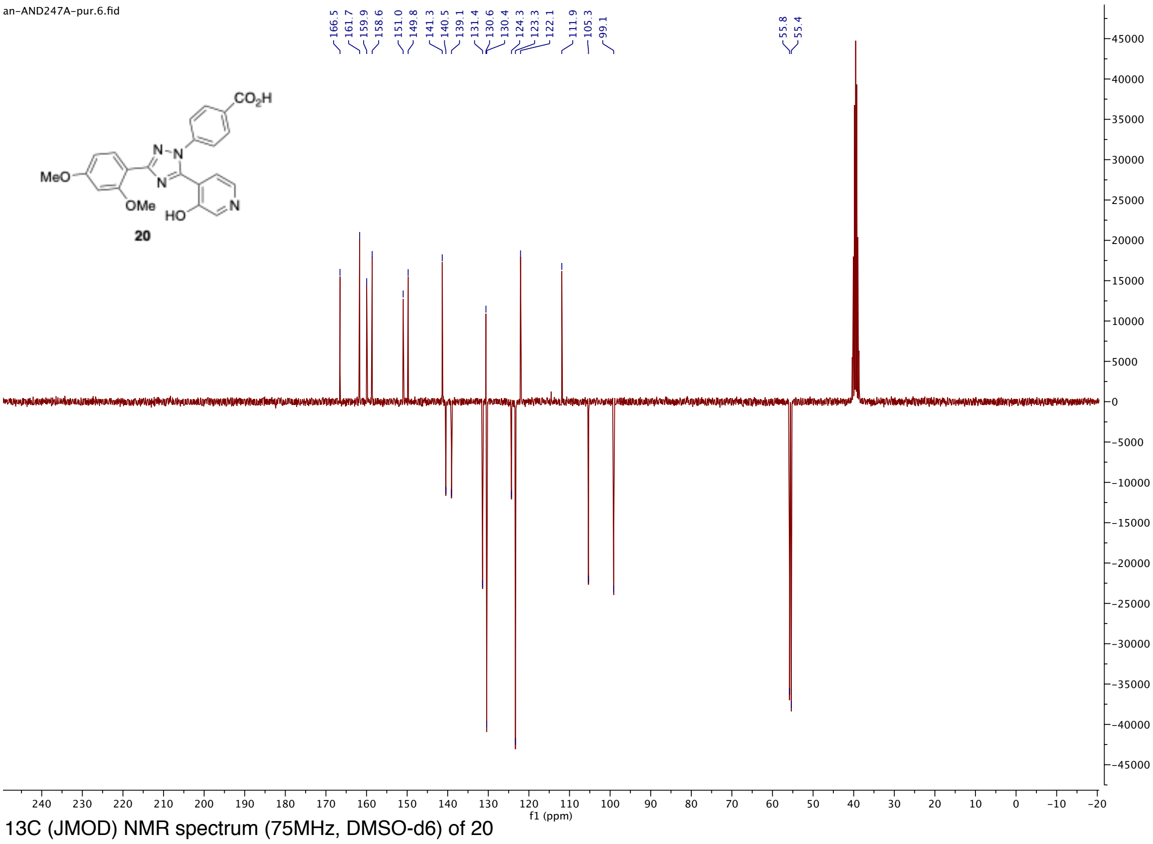

13C (JMOD) NMR spectrum (75MHz, DMSO-d6) of 20

4. HPLC chromatograms of all the tested compounds 3a, 3b, 3c, 3d, 3e, 3fa, 3fb, 3g, 3h, 3i, 3ja, 3jb, 3ka, 3kb, 3la, 3lb, 3ma, 3mb, 3n, 3o, 3q, 3r, 3s, 3t, 3u, 3v, 3w, 9, 10, 13, 14, 18, 19, 21, 22, 23.

Figure S38 : HPLC chromatogram of 3a

Chromatogram molecule 3a

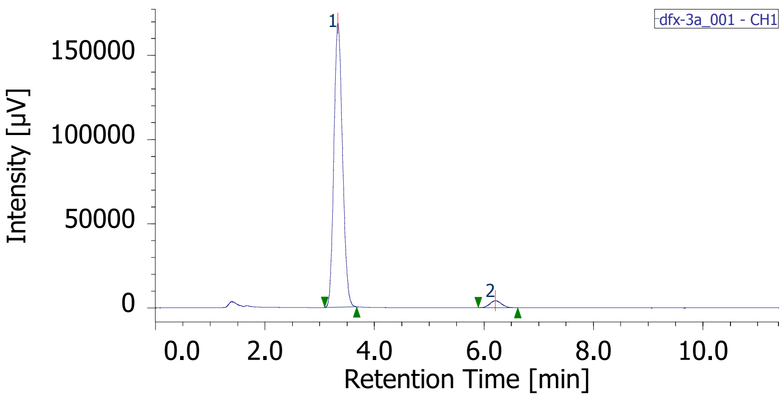

Chromatogram Information	
User Name	COM
Date Modified	3/7/2024 5:40:33 PM
Description	
HPLC System Name	HPLC binaire
Injection Date	3/7/2024 5:29:09 PM
Volume	20.00 [µL]
Sample Number	-
Project Name	HPLC binaire
Acquisition Time	14.9 [min]
Acquisition Sequence	
Control Method	elodie+vincent-70%MeCN-TFA-300nm
Peak ID Table	
Calibration Method	
Additional Information	

Channel & Peak Information Table	
Chromatogram Name	dfx-3a_001-CH1
Sample Name	
Channel Name	UV
Sampling Interval	200 [msec]
Peak Method	(Manual)
Formula	
Decision	

#	Peak Name	CH	tR [min]	Area [µV·sec]	Height [µV]	Area%	Height%	Quantity	NTP	Resolution	Symmetry Factor	Warning
1	Unknown	1	3.33	1834863	168417	96.834	97.634	N/A	2133	8.488	1.145	
2	Unknown	1	6.21	59996	4081	3.166	2.366	N/A	4015	N/A	1.047	

1 / 1

Figure S39 : HPLC chromatogram of 3b

Chromatogram molecule 3b

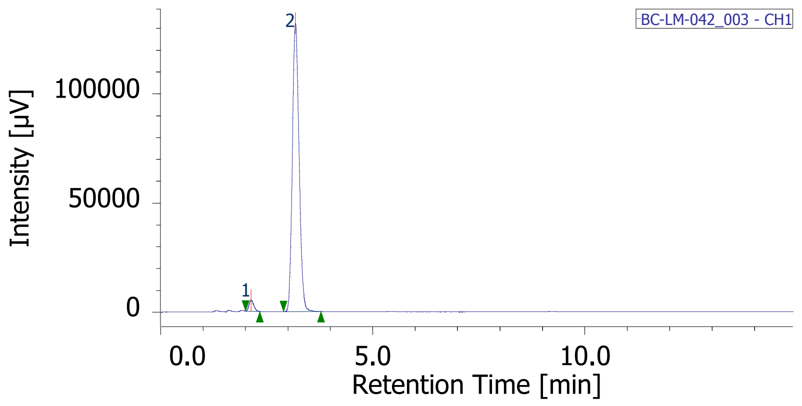

Chromatogram Information

User Name	COM
Date Modified	2/8/2024 5:00:43 PM
Description	
HPLC System Name	HPLC binaire
Injection Date	2/8/2024 10:34:47 AM
Volume	20.00 [µL]
Sample Number	-
Project Name	HPLC binaire
Acquisition Time	14.9 [min]
Acquisition Sequence	
Control Method	elodie+vincent-70%MeCN-TFA-254nm
Peak ID Table	
Calibration Method	
Additional Information	

Channel & Peak Information Table

Chromatogram Name	BC-LM-042_003-CH1
Sample Name	
Channel Name	UV
Sampling Interval	200 [msec]
Peak Method	(Manual)
Formula	
Decision	

#	Peak Name	CH	tR [min]	Area [µV·sec]	Height [µV]	Area%	Height%	Quantity	NTP	Resolution	Symmetry Factor	Warning
1	Unknown	1	2.13	40581	5142	2.722	3.753	N/A	1633	4.196	1.243	
2	Unknown	1	3.18	1450135	131877	97.278	96.247	N/A	1928	N/A	1.149	

1 / 1

Figure S40 : HPLC chromatogram of 3c

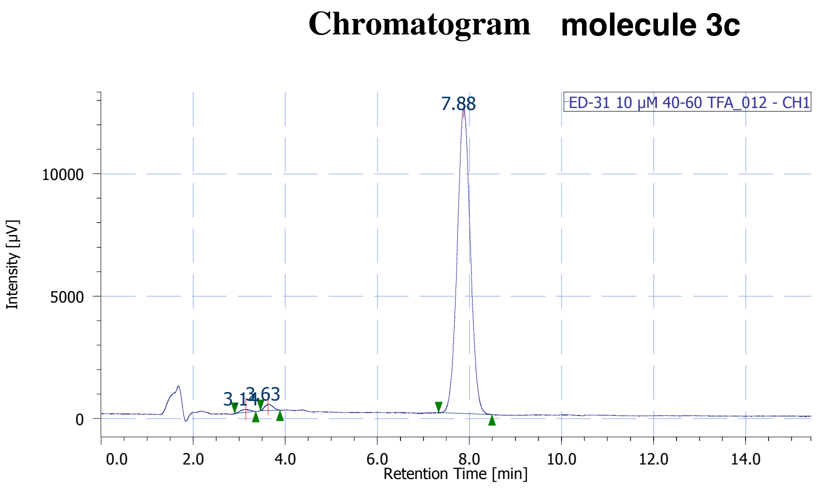

Chromatogram Information	
User Name	COM
Date Modified	3/15/2023 6:20:20 PM
Description	
HPLC System Name	HPLC binaire
Injection Date	3/15/2023 4:40:43 PM
Volume	20.00 [µL]
Sample Number	-
Project Name	HPLC binaire
Acquisition Time	29.9 [min]
Acquisition Sequence	
Control Method	elodie40-60+TFA
Peak ID Table	
Calibration Method	
Additional Information	

Channel & Peak Information Table	
Chromatogram Name	ED-31 10 µM 40-60 TFA_012-CH1
Sample Name	
Channel Name	UV
Sampling Interval	200 [msec]
Peak Method	(Manual)
Formula	
Decision	

#	Peak Name	CH	tR [min]	Area [µV·sec]	Height [µV]	Area%	Height%	Quantity	NTP	Resolution	Symmetry Factor	Warning
1	Unknown	1	3.14	1812	120	0.718	0.931	N/A	850	1.250	0.928	
2	Unknown	1	3.63	3131	256	1.241	1.991	N/A	1778	9.868	1.230	
3	Unknown	1	7.88	247303	12499	98.040	97.077	N/A	3676	N/A	1.020	

1 / 1

Figure S41 : HPLC chromatogram of 3d

Chromatogram molecule 3d

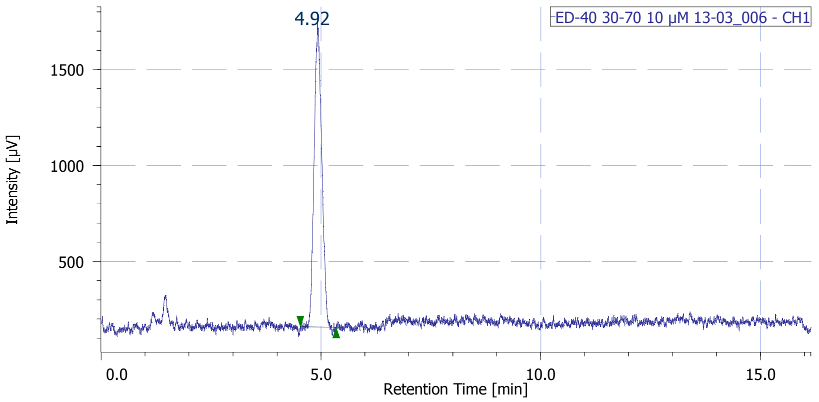

Chromatogram Information	
User Name	COM
Date Modified	3/13/2023 3:51:25 PM
Description	
HPLC System Name	HPLC binaire
Injection Date	3/13/2023 3:35:15 PM
Volume	20.00 [µL]
Sample Number	-
Project Name	HPLC binaire
Acquisition Time	29.9 [min]
Acquisition Sequence	
Control Method	elodie30-70
Peak ID Table	
Calibration Method	
Additional Information	

Channel & Peak Information Table	
Chromatogram Name	ED-40 30-70 10 µM 13-03_006-CH1
Sample Name	
Channel Name	UV
Sampling Interval	200 [msec]
Peak Method	(Manual)
Formula	
Decision	

#	Peak Name	CH	tR [min]	Area [µV·sec]	Height [µV]	Area%	Height%	Quantity	NTP	Resolution	Symmetry Factor
1	Unknown	1	4.92	19172	1586	100.000	100.000	N/A	3502	N/A	1.166

#	Warning
1	

1 / 1

Figure S42 : HPLC chromatogram of 3e

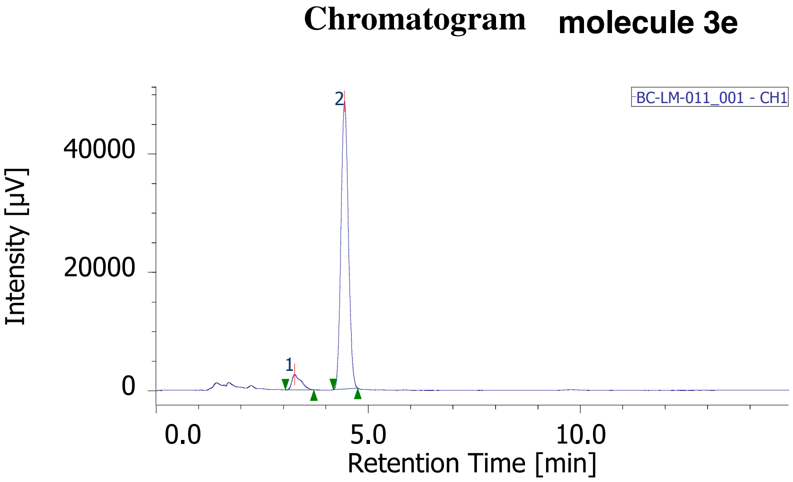

Chromatogram Information

User Name	COM
Date Modified	2/8/2024 5:00:41 PM
Description	
HPLC System Name	HPLC binaire
Injection Date	2/8/2024 9:51:43 AM
Volume	20.00 [µL]
Sample Number	-
Project Name	HPLC binaire
Acquisition Time	14.9 [min]
Acquisition Sequence	
Control Method	elodie+vincent-70%MeCN-TFA-254nm
Peak ID Table	
Calibration Method	
Additional Information	

Channel & Peak Information Table

Chromatogram Name	BC-LM-011_001-CH1
Sample Name	
Channel Name	UV
Sampling Interval	200 [msec]
Peak Method	(Manual)
Formula	
Decision	

#	Peak Name	CH	tR [min]	Area [µV·sec]	Height [µV]	Area%	Height%	Quantity	NTP	Resolution	Symmetry Factor	Warning
1	Unknown	1	3.26	38516	2586	6.181	5.059	N/A	945	3.167	1.764	
2	Unknown	1	4.44	584590	48521	93.819	94.941	N/A	3068	N/A	1.091	

1 / 1

Figure S43 : HPLC chromatogram of 3fa

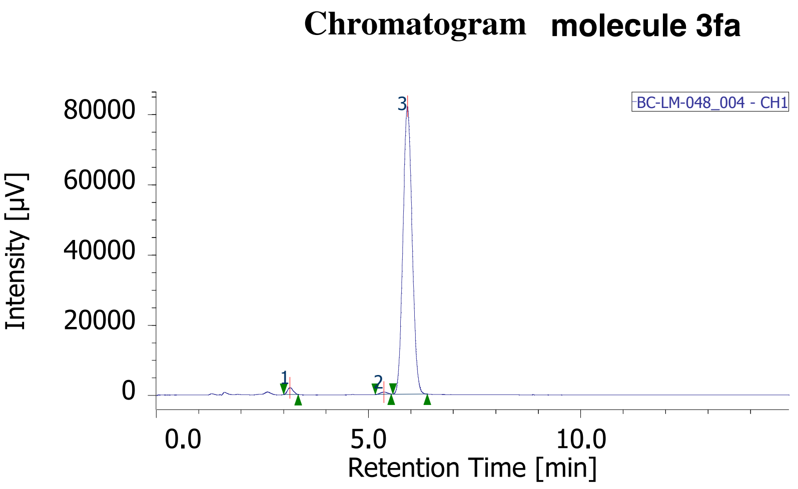

Chromatogram Information	
User Name	COM
Date Modified	2/8/2024 11:06:06 AM
Description	
HPLC System Name	HPLC binaire
Injection Date	2/8/2024 10:51:12 AM
Volume	20.00 [µL]
Sample Number	-
Project Name	HPLC binaire
Acquisition Time	14.9 [min]
Acquisition Sequence	
Control Method	elodie+vincent-70%MeCN-TFA-254nm
Peak ID Table	
Calibration Method	
Additional Information	

Channel & Peak Information Table	
Chromatogram Name	BC-LM-048_004-CH1
Sample Name	
Channel Name	UV
Sampling Interval	200 [msec]
Peak Method	(Manual)
Formula	
Decision	

#	Peak Name	CH	tR [min]	Area [µV·sec]	Height [µV]	Area%	Height%	Quantity	NTP	Resolution	Symmetry Factor	Warning
1	Unknown	1	3.15	18189	1928	1.452	2.284	N/A	2407	7.574	1.169	
2	Unknown	1	5.36	7175	613	0.573	0.725	N/A	4234	1.534	0.959	
3	Unknown	1	5.92	1227530	81900	97.976	96.991	N/A	3535	N/A	1.064	

1 / 1

Figure S44 : HPLC chromatogram of 3fb

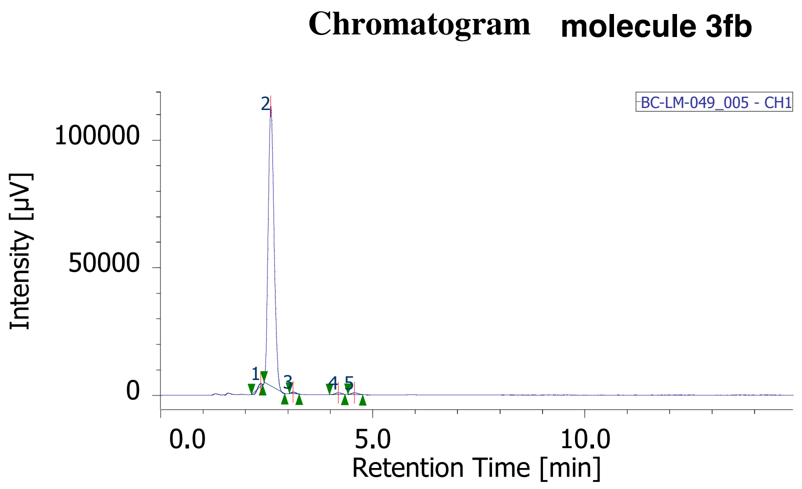

Chromatogram Information
User Name COM
Date Modified 2/8/2024 11:22:20 AM
Description
HPLC System Name HPLC binaire
Injection Date 2/8/2024 11:07:26 AM
Volume 20.00 [µL]
Sample Number -
Project Name HPLC binaire
Acquisition Time 14.9 [min]
Acquisition Sequence
Control Method elodie+vincent-70%MeCN-TFA-254nm
Peak ID Table
Calibration Method
Additional Information

Channel & Peak Information Table
Chromatogram Name BC-LM-049_005-CH1
Sample Name
Channel Name UV
Sampling Interval 200 [msec]
Peak Method (Manual)
Formula
Decision

#	Peak Name	CH	tR [min]	Area [µV·sec]	Height [µV]	Area%	Height%	Quantity	NTP	Resolution	Symmetry Factor	Warning
1	Unknown	1	2.36	5788	962	0.541	0.857	N/A	3457	1.140	0.715	
2	Unknown	1	2.60	1046130	109386	97.764	97.441	N/A	1648	2.285	1.194	
3	Unknown	1	3.12	4463	613	0.417	0.546	N/A	3668	4.312	1.321	
4	Unknown	1	4.19	6807	663	0.636	0.591	N/A	3377	1.289	0.950	
5	Unknown	1	4.57	6872	635	0.642	0.566	N/A	3640	N/A	1.151	

1 / 1

Figure S45 : HPLC chromatogram of 3g

Chromatogram molecule 3g

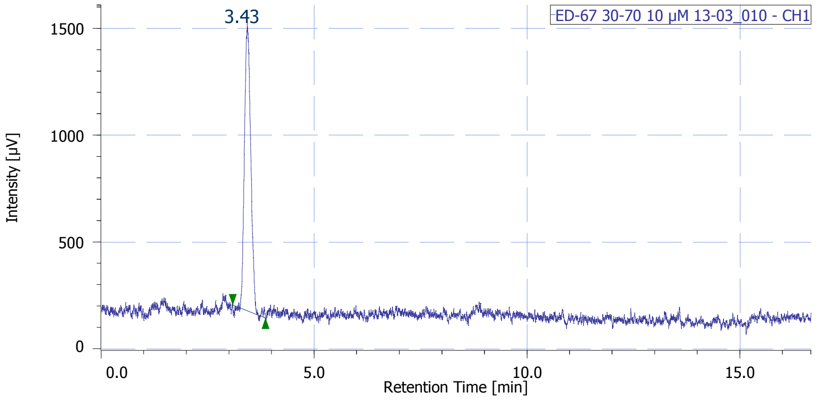

Chromatogram Information

User Name	COM
Date Modified	3/13/2023 5:01:53 PM
Description	
HPLC System Name	HPLC binaire
Injection Date	3/13/2023 4:45:11 PM
Volume	20.00 [µL]
Sample Number	-
Project Name	HPLC binaire
Acquisition Time	29.9 [min]
Acquisition Sequence	
Control Method	elodie30-70
Peak ID Table	
Calibration Method	
Additional Information	

Channel & Peak Information Table

Chromatogram Name	ED-67 30-70 10 µM 13-03_010-CH1
Sample Name	
Channel Name	UV
Sampling Interval	200 [msec]
Peak Method	(Manual)
Formula	
Decision	

#	Peak Name	CH	tR [min]	Area [µV·sec]	Height [µV]	Area%	Height%	Quantity	NTP	Resolution	Symmetry Factor
1	Unknown	1	3.43	13959	1357	100.000	100.000	N/A	2454	N/A	1.175

#	Warning
1	

1 / 1

Figure S46 : HPLC chromatogram of 3h

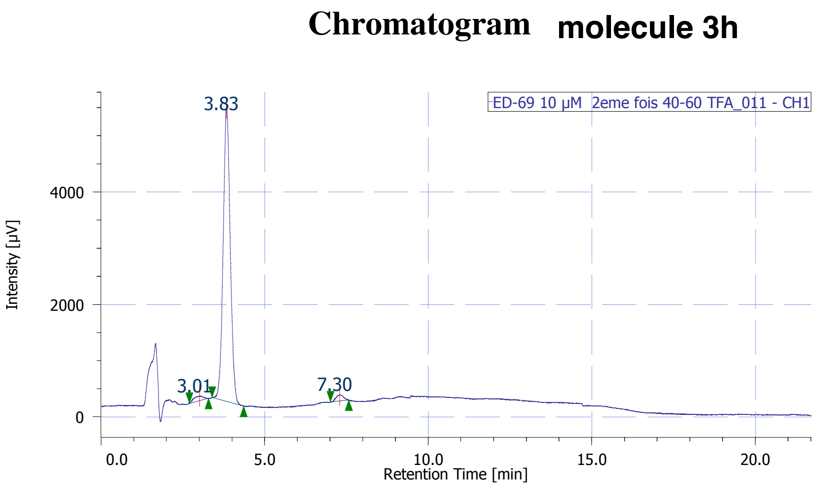

Chromatogram Information

User Name	COM
Date Modified	3/15/2023 6:20:19 PM
Description	
HPLC System Name	HPLC binaire
Injection Date	3/15/2023 4:16:30 PM
Volume	20.00 [µL]
Sample Number	-
Project Name	HPLC binaire
Acquisition Time	29.9 [min]
Acquisition Sequence	
Control Method	elodie40-60+TFA
Peak ID Table	
Calibration Method	
Additional Information	

Channel & Peak Information Table

Chromatogram Name	ED-69 10 µM 2eme fois 40-60 TFA_011-CH1
Sample Name	
Channel Name	UV
Sampling Interval	200 [msec]
Peak Method	(Manual)
Formula	
Decision	

#	Peak Name	CH	tR [min]	Area [µV·sec]	Height [µV]	Area%	Height%	Quantity	NTP	Resolution	Symmetry Factor	Warning
1	Unknown	1	3.01	1748	82	2.129	1.517	N/A	375	1.624	0.863	
2	Unknown	1	3.83	78568	5219	95.716	96.436	N/A	1546	8.355	1.059	
3	Unknown	1	7.30	1768	111	2.154	2.047	N/A	4376	N/A	1.025	

1 / 1

Figure S47 : HPLC chromatogram of 3i

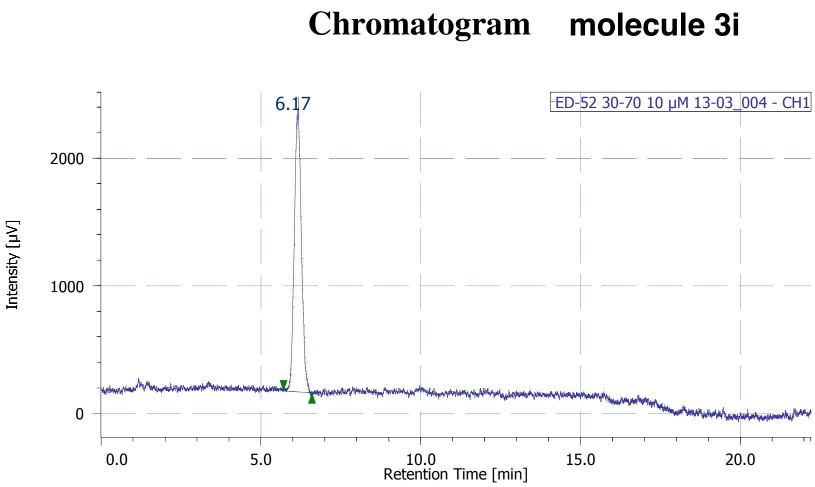

Chromatogram Information

User Name	COM
Date Modified	3/13/2023 3:10:03 PM
Description	
HPLC System Name	HPLC binaire
Injection Date	3/13/2023 2:47:48 PM
Volume	20.00 [µL]
Sample Number	-
Project Name	HPLC binaire
Acquisition Time	29.9 [min]
Acquisition Sequence	
Control Method	elodie30-70
Peak ID Table	
Calibration Method	
Additional Information	

Channel & Peak Information Table

Chromatogram Name	ED-52 30-70 10 µM 13-03_004-CH1
Sample Name	
Channel Name	UV
Sampling Interval	200 [msec]
Peak Method	(Manual)
Formula	
Decision	

#	Peak Name	CH	tR [min]	Area [µV·sec]	Height [µV]	Area%	Height%	Quantity	NTP	Resolution	Symmetry Factor
1	Unknown	1	6.17	34165	2227	100.000	100.000	N/A	3801	N/A	1.032

#	Warning
1	

1 / 1

Figure S48 : HPLC chromatogram of 3ja

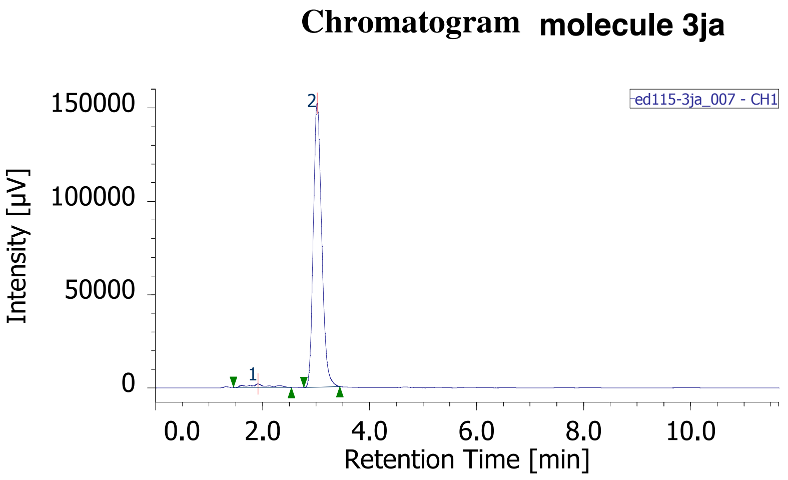

Chromatogram Information	
User Name	COM
Date Modified	3/7/2024 7:22:38 PM
Description	
HPLC System Name	HPLC binaire
Injection Date	3/7/2024 7:10:58 PM
Volume	20.00 [µL]
Sample Number	-
Project Name	HPLC binaire
Acquisition Time	14.9 [min]
Acquisition Sequence	
Control Method	elodie+vincent-70%MeCN-TFA-300nm
Peak ID Table	
Calibration Method	
Additional Information	

Channel & Peak Information Table	
Chromatogram Name	ed115-3ja_007-CH1
Sample Name	
Channel Name	UV
Sampling Interval	200 [msec]
Peak Method	(Manual)
Formula	
Decision	

#	Peak Name	CH	tR [min]	Area [µV·sec]	Height [µV]	Area%	Height%	Quantity	NTP	Resolution	Symmetry Factor	Warning
1	Unknown	1	1.91	47738	1938	2.792	1.260	N/A	121	2.263	1.149	
2	Unknown	1	3.02	1661990	151924	97.208	98.740	N/A	1797	N/A	1.198	

1 / 1

Figure S49 : HPLC chromatogram of 3jb

Chromatogram molecule 3jb

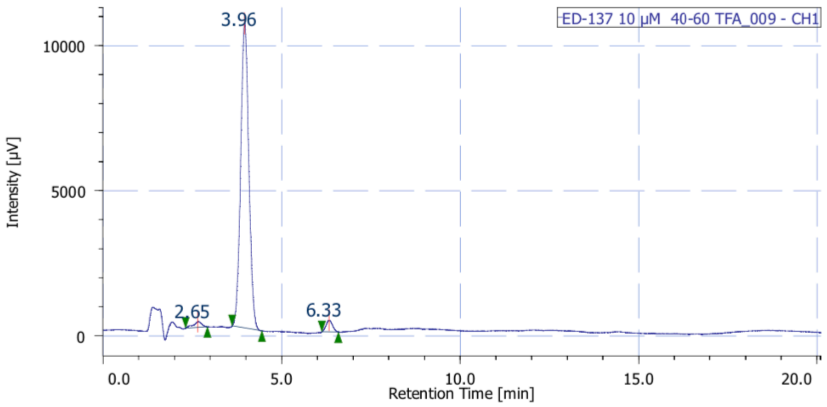

Chromatogram Information	
User Name	COM
Date Modified	3/15/2023 6:20:16 PM
Description	
HPLC System Name	HPLC binaire
Injection Date	3/15/2023 2:54:03 PM
Volume	20.00 [µL]
Sample Number	-
Project Name	HPLC binaire
Acquisition Time	29.9 [min]
Acquisition Sequence	
Control Method	elodie40-60+TFA
Peak ID Table	
Calibration Method	
Additional Information	

Channel & Peak Information Table	
Chromatogram Name	ED-137 10 µM 40-60 TFA_009-CH1
Sample Name	
Channel Name	UV
Sampling Interval	200 [msec]
Peak Method	(Manual)
Formula	
Decision	

#	Peak Name	CH	tR [min]	Area [µV·sec]	Height [µV]	Area%	Height%	Quantity	NTP	Resolution	Symmetry Factor	Warning
1	Unknown	1	2.65	3236	199	1.853	1.795	N/A	892	3.408	0.842	
2	Unknown	1	3.96	166263	10484	95.216	94.581	N/A	1457	6.259	1.046	
3	Unknown	1	6.33	5117	402	2.931	3.624	N/A	5483	N/A	1.093	

1 / 1

Figure S50 : HPLC chromatogram of 3ka

Chromatogram molecule 3ka

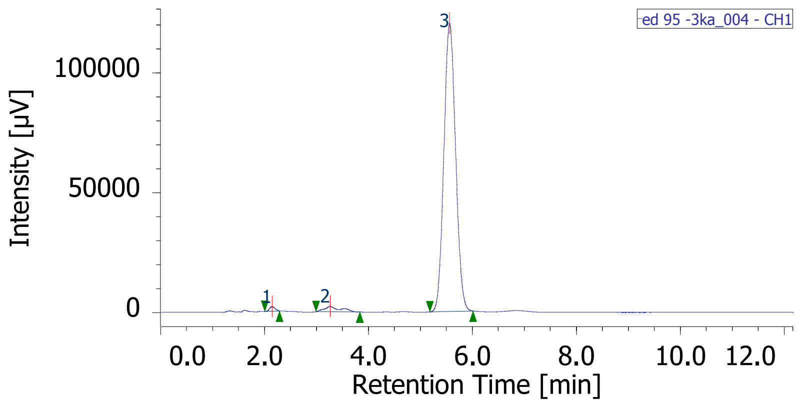

Chromatogram Information

User Name	COM
Date Modified	3/7/2024 6:26:44 PM
Description	
HPLC System Name	HPLC binaire
Injection Date	3/7/2024 6:14:32 PM
Volume	20.00 [µL]
Sample Number	-
Project Name	HPLC binaire
Acquisition Time	14.9 [min]
Acquisition Sequence	
Control Method	elodie+vincent-70%MeCN-TFA-300nm
Peak ID Table	
Calibration Method	
Additional Information	

Channel & Peak Information Table

Chromatogram Name	ed 95 -3ka_004-CH1
Sample Name	
Channel Name	UV
Sampling Interval	200 [msec]
Peak Method	(Manual)
Formula	
Decision	

#	Peak Name	CH	tR [min]	Area [µV·sec]	Height [µV]	Area%	Height%	Quantity	NTP	Resolution	Symmetry Factor	Warning
1	Unknown	1	2.14	14684	2000	0.755	1.609	N/A	1815	2.353	1.109	
2	Unknown	1	3.26	49155	2244	2.527	1.805	N/A	300	3.938	1.438	
3	Unknown	1	5.56	1881396	120074	96.718	96.587	N/A	2853	N/A	1.063	

1 / 1

Figure S51 : HPLC chromatogram of 3kb

Chromatogram molecule 3kb

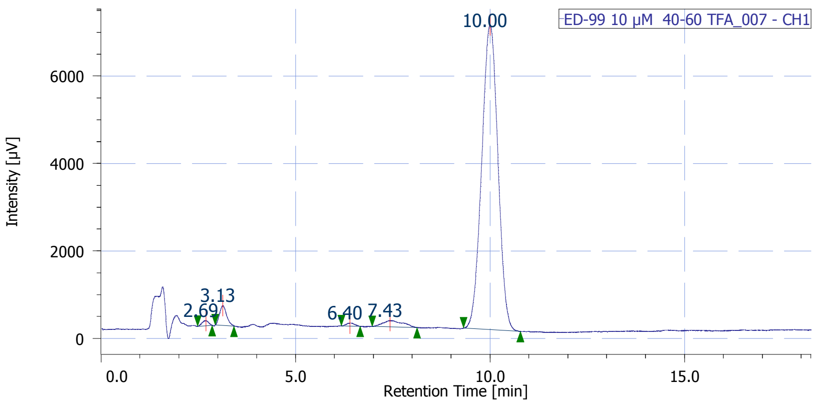

Chromatogram Information

User Name	COM
Date Modified	3/15/2023 6:20:18 PM
Description	
HPLC System Name	HPLC binaire
Injection Date	3/15/2023 12:35:33 PM
Volume	20.00 [µL]
Sample Number	-
Project Name	HPLC binaire
Acquisition Time	29.9 [min]
Acquisition Sequence	
Control Method	elodie40-60+TFA
Peak ID Table	
Calibration Method	
Additional Information	

Channel & Peak Information Table

Chromatogram Name	ED-99 10 µM 40-60 TFA_007-CH1
Sample Name	
Channel Name	UV
Sampling Interval	200 [msec]
Peak Method	(Manual)
Formula	
Decision	

#	Peak Name	CH	tR [min]	Area [µV·sec]	Height [µV]	Area%	Height%	Quantity	NTP	Resolution	Symmetry Factor	Warning
1	Unknown	1	2.69	1383	121	0.643	1.566	N/A	1111	1.326	0.913	
2	Unknown	1	3.13	5739	451	2.667	5.820	N/A	1377	8.644	1.208	
3	Unknown	1	6.40	1012	69	0.471	0.884	N/A	3693	1.345	1.119	
4	Unknown	1	7.43	5248	145	2.439	1.874	N/A	712	2.732	1.222	
5	Unknown	1	10.00	201759	6969	93.780	89.857	N/A	2680	N/A	0.984	

1 / 1

Figure S52 : HPLC chromatogram of 3la

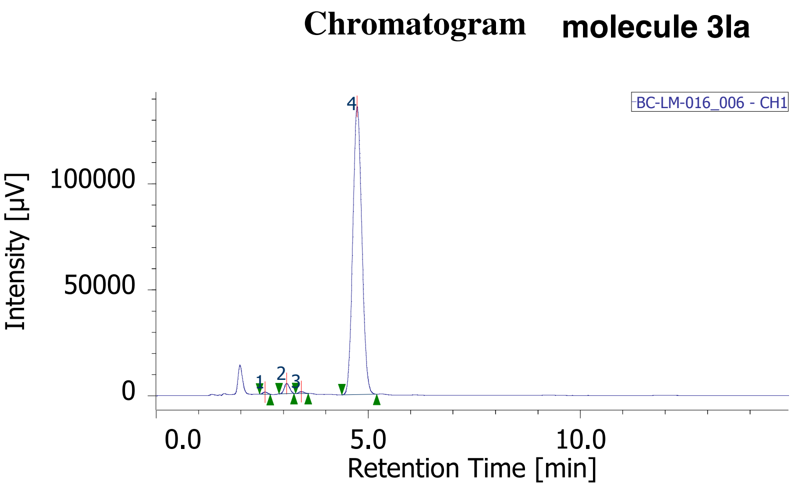

Chromatogram Information

User Name	COM
Date Modified	2/8/2024 11:41:12 AM
Description	
HPLC System Name	HPLC binaire
Injection Date	2/8/2024 11:26:16 AM
Volume	20.00 [µL]
Sample Number	-
Project Name	HPLC binaire
Acquisition Time	14.9 [min]
Acquisition Sequence	
Control Method	elodie+vincent-70%MeCN-TFA-254nm
Peak ID Table	
Calibration Method	
Additional Information	

Channel & Peak Information Table

Chromatogram Name	BC-LM-016_006-CH1
Sample Name	
Channel Name	UV
Sampling Interval	200 [msec]
Peak Method	(Manual)
Formula	
Decision	

#	Peak Name	CH	tR [min]	Area [µV·sec]	Height [µV]	Area%	Height%	Quantity	NTP	Resolution	Symmetry Factor	Warning
1	Unknown	1	2.57	7608	988	0.367	0.693	N/A	2272	2.250	1.040	
2	Unknown	1	3.08	42615	4824	2.058	3.384	N/A	2650	1.478	1.089	
3	Unknown	1	3.42	8227	995	0.397	0.698	N/A	3639	4.254	1.109	
4	Unknown	1	4.74	2012016	135748	97.177	95.225	N/A	2313	N/A	1.084	

1 / 1

Figure S53 : HPLC chromatogram of 3lb

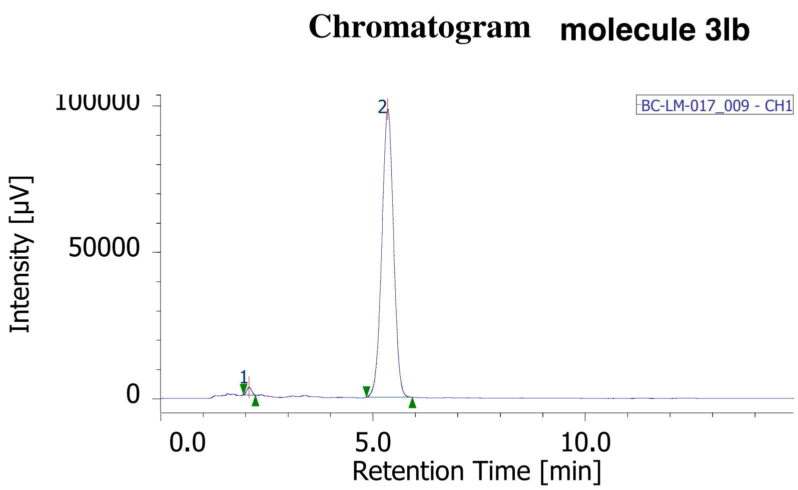

Chromatogram Information	
User Name	COM
Date Modified	2/8/2024 4:38:32 PM
Description	
HPLC System Name	HPLC binaire
Injection Date	2/8/2024 4:23:38 PM
Volume	20.00 [µL]
Sample Number	-
Project Name	HPLC binaire
Acquisition Time	14.9 [min]
Acquisition Sequence	
Control Method	elodie+vincent-70%MeCN-TFA-254nm
Peak ID Table	
Calibration Method	
Additional Information	

Channel & Peak Information Table	
Chromatogram Name	BC-LM-017_009-CH1
Sample Name	
Channel Name	UV
Sampling Interval	200 [msec]
Peak Method	(Manual)
Formula	
Decision	

#	Peak Name	CH	tR [min]	Area [µV·sec]	Height [µV]	Area%	Height%	Quantity	NTP	Resolution	Symmetry Factor	Warning
1	Unknown	1	2.08	21441	2714	1.125	2.689	N/A	1495	9.104	1.067	
2	Unknown	1	5.35	1883823	98215	98.875	97.311	N/A	1798	N/A	0.987	

1 / 1

Figure S54 : HPLC chromatogram of 3ma

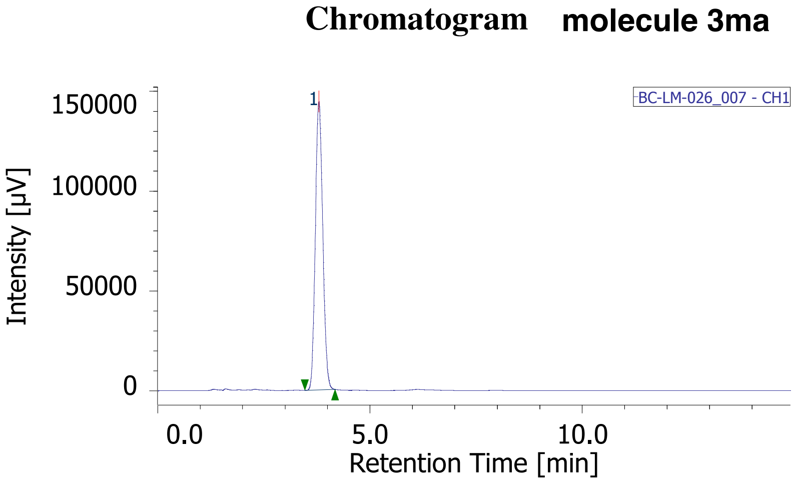

Chromatogram Information

User Name	COM
Date Modified	2/8/2024 11:57:32 AM
Description	
HPLC System Name	HPLC binaire
Injection Date	2/8/2024 11:42:37 AM
Volume	20.00 [µL]
Sample Number	-
Project Name	HPLC binaire
Acquisition Time	14.9 [min]
Acquisition Sequence	
Control Method	elodie+vincent-70%MeCN-TFA-254nm
Peak ID Table	
Calibration Method	
Additional Information	

Channel & Peak Information Table

Chromatogram Name	BC-LM-026_007-CH1
Sample Name	
Channel Name	UV
Sampling Interval	200 [msec]
Peak Method	(Manual)
Formula	
Decision	

#	Peak Name	CH	tR [min]	Area [µV·sec]	Height [µV]	Area%	Height%	Quantity	NTP	Resolution	Symmetry Factor
1	Unknown	1	3.79	1754527	144287	100.000	100.000	N/A	2216	N/A	1.120

#	Warning
1	

1 / 1

Figure S55 : HPLC chromatogram of 3mb

Chromatogram **molecule 3mb**

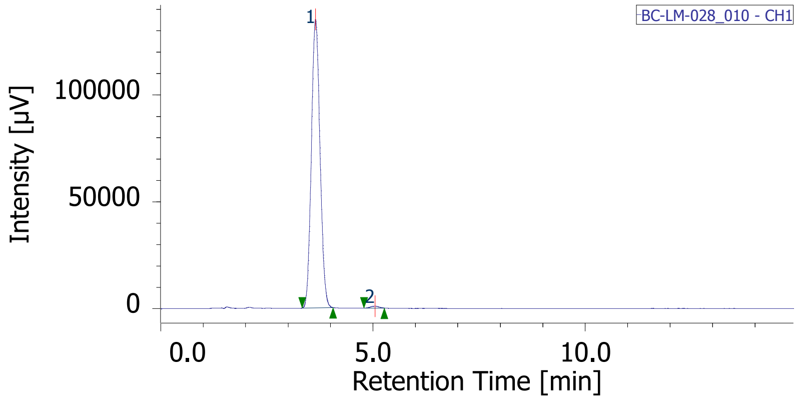

Chromatogram Information

User Name	COM
Date Modified	2/8/2024 4:54:48 PM
Description	
HPLC System Name	HPLC binaire
Injection Date	2/8/2024 4:39:53 PM
Volume	20.00 [µL]
Sample Number	-
Project Name	HPLC binaire
Acquisition Time	14.9 [min]
Acquisition Sequence	
Control Method	elodie+vincent-70%MeCN-TFA-254nm
Peak ID Table	
Calibration Method	
Additional Information	

Channel & Peak Information Table

Chromatogram Name	BC-LM-028_010-CH1
Sample Name	
Channel Name	UV
Sampling Interval	200 [msec]
Peak Method	(Manual)
Formula	
Decision	

#	Peak Name	CH	tR [min]	Area [µV·sec]	Height [µV]	Area%	Height%	Quantity	NTP	Resolution	Symmetry Factor	Warning
1	Unknown	1	3.65	1905026	134751	99.385	99.371	N/A	1518	3.704	1.077	
2	Unknown	1	5.04	11786	852	0.615	0.629	N/A	2793	N/A	0.971	

1 / 1

Figure S56 : HPLC chromatogram of 3n

Chromatogram molecule 3n

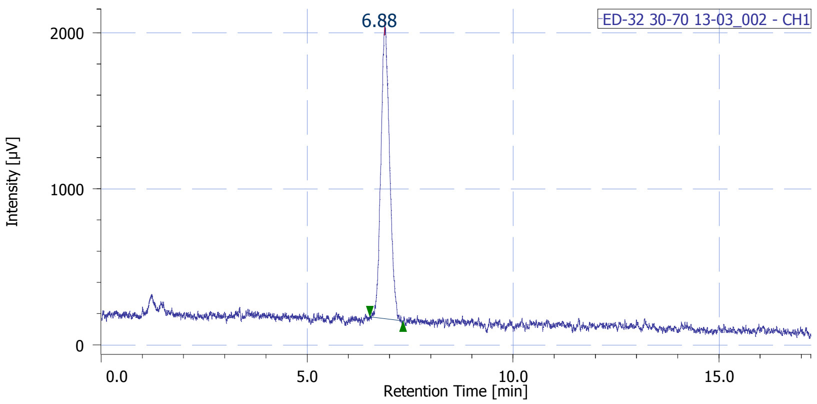

Chromatogram Information

User Name	COM
Date Modified	3/13/2023 2:23:56 PM
Description	
HPLC System Name	HPLC binaire
Injection Date	3/13/2023 2:06:41 PM
Volume	20.00 [µL]
Sample Number	-
Project Name	HPLC binaire
Acquisition Time	29.9 [min]
Acquisition Sequence	
Control Method	elodie30-70
Peak ID Table	
Calibration Method	
Additional Information	

Channel & Peak Information Table

Chromatogram Name	ED-32 30-70 13-03_002-CH1
Sample Name	
Channel Name	UV
Sampling Interval	200 [msec]
Peak Method	(Manual)
Formula	
Decision	

#	Peak Name	CH	tR [min]	Area [µV·sec]	Height [µV]	Area%	Height%	Quantity	NTP	Resolution	Symmetry Factor
1	Unknown	1	6.88	27599	1882	100.000	100.000	N/A	5043	N/A	1.175

#	Warning
1	

1 / 1

Figure S57 : HPLC chromatogram of 3o

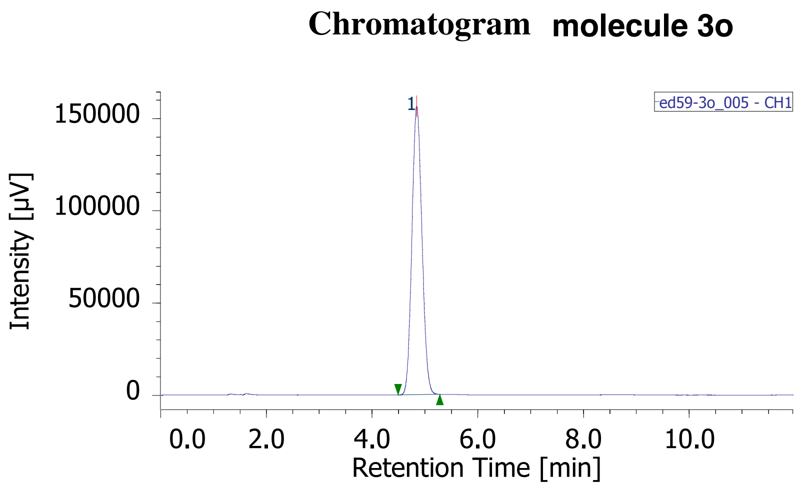

Chromatogram Information	
User Name	COM
Date Modified	3/7/2024 6:43:17 PM
Description	
HPLC System Name	HPLC binaire
Injection Date	3/7/2024 6:31:17 PM
Volume	20.00 [µL]
Sample Number	-
Project Name	HPLC binaire
Acquisition Time	14.9 [min]
Acquisition Sequence	
Control Method	elodie+vincent-70%MeCN-TFA-300nm
Peak ID Table	
Calibration Method	
Additional Information	

Channel & Peak Information Table	
Chromatogram Name	ed59-3o_005-CH1
Sample Name	
Channel Name	UV
Sampling Interval	200 [msec]
Peak Method	(Manual)
Formula	
Decision	

#	Peak Name	CH	tR [min]	Area [µV·sec]	Height [µV]	Area%	Height%	Quantity	NTP	Resolution	Symmetry Factor
1	Unknown	1	4.84	2059107	156041	100.000	100.000	N/A	3054	N/A	1.087

#	Warning
1	

1 / 1

Figure S58 : HPLC chromatogram of 3p

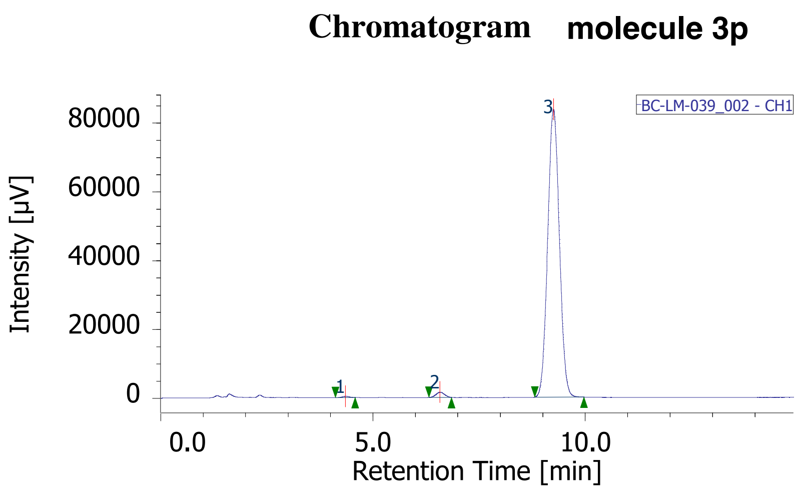

Chromatogram Information	
User Name	COM
Date Modified	2/8/2024 5:00:42 PM
Description	
HPLC System Name	HPLC binaire
Injection Date	2/8/2024 10:15:21 AM
Volume	20.00 [µL]
Sample Number	-
Project Name	HPLC binaire
Acquisition Time	14.9 [min]
Acquisition Sequence	
Control Method	elodie+vincent-70%MeCN-TFA-254nm
Peak ID Table	
Calibration Method	
Additional Information	

Channel & Peak Information Table	
Chromatogram Name	BC-LM-039_002-CH1
Sample Name	
Channel Name	UV
Sampling Interval	200 [msec]
Peak Method	(Manual)
Formula	
Decision	

#	Peak Name	CH	tR [min]	Area [µV·sec]	Height [µV]	Area%	Height%	Quantity	NTP	Resolution	Symmetry Factor	Warning
1	Unknown	1	4.35	3650	334	0.225	0.392	N/A	3544	6.627	1.074	
2	Unknown	1	6.57	21284	1513	1.315	1.772	N/A	4774	6.051	1.056	
3	Unknown	1	9.25	1594130	83533	98.460	97.836	N/A	5335	N/A	1.041	

1 / 1

Figure S59 : HPLC chromatogram of 3q

Chromatogram **molecule 3q**

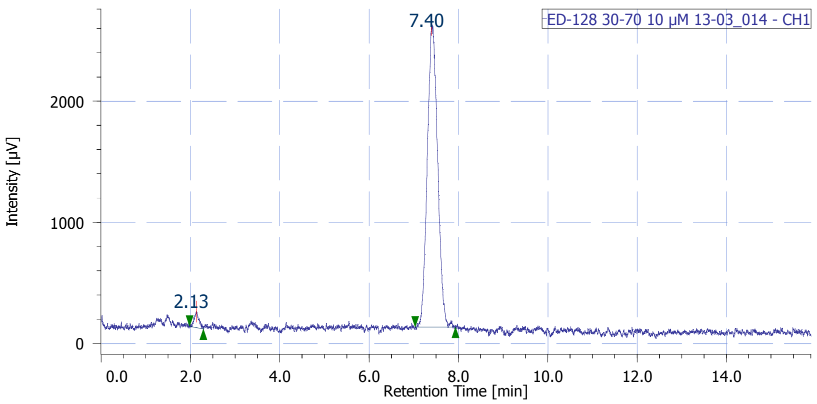

Chromatogram Information	
User Name	COM
Date Modified	3/13/2023 6:10:43 PM
Description	
HPLC System Name	HPLC binaire
Injection Date	3/13/2023 5:54:48 PM
Volume	20.00 [µL]
Sample Number	-
Project Name	HPLC binaire
Acquisition Time	29.9 [min]
Acquisition Sequence	
Control Method	elodie30-70
Peak ID Table	
Calibration Method	
Additional Information	

Channel & Peak Information Table	
Chromatogram Name	ED-128 30-70 10 µM 13-03_014-CH1
Sample Name	
Channel Name	UV
Sampling Interval	200 [msec]
Peak Method	(Manual)
Formula	
Decision	

#	Peak Name	CH	tR [min]	Area [µV·sec]	Height [µV]	Area%	Height%	Quantity	NTP	Resolution	Symmetry Factor	Warning
1	Unknown	1	2.13	984	132	2.389	5.004	N/A	1545	16.135	1.080	
2	Unknown	1	7.40	40194	2497	97.611	94.996	N/A	4567	N/A	1.160	

1 / 1

Figure S60 : HPLC chromatogram of 3r

Chromatogram **molecule 3r**

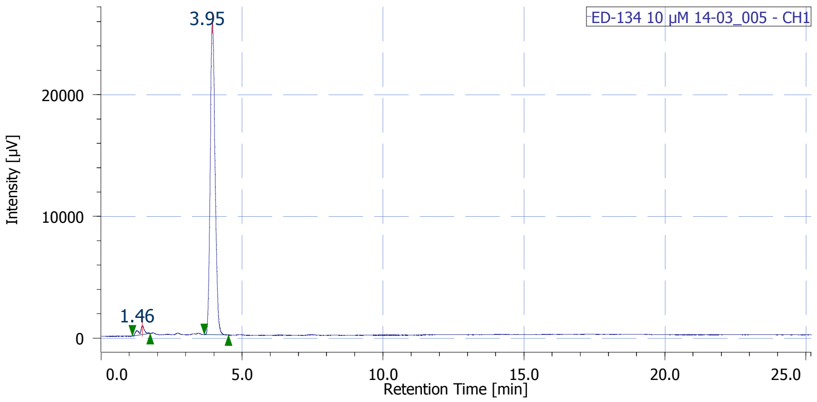

Chromatogram Information	
User Name	COM
Date Modified	3/14/2023 11:28:11 AM
Description	
HPLC System Name	HPLC binaire
Injection Date	3/14/2023 11:02:58 AM
Volume	20.00 [μL]
Sample Number	-
Project Name	HPLC binaire
Acquisition Time	29.9 [min]
Acquisition Sequence	
Control Method	elodie30-70
Peak ID Table	
Calibration Method	
Additional Information	

Channel & Peak Information Table	
Chromatogram Name	ED-134 10 μM 14-03_005-CH1
Sample Name	
Channel Name	UV
Sampling Interval	200 [msec]
Peak Method	(Manual)
Formula	
Decision	

#	Peak Name	CH	tR [min]	Area [μV·sec]	Height [μV]	Area%	Height%	Quantity	NTP	Resolution	Symmetry Factor	Warning
1	Unknown	1	1.46	9457	725	2.981	2.750	N/A	136	6.081	0.910	
2	Unknown	1	3.95	307752	25631	97.019	97.250	N/A	2477	N/A	1.179	

1 / 1

Figure S61 : HPLC chromatogram of 3s

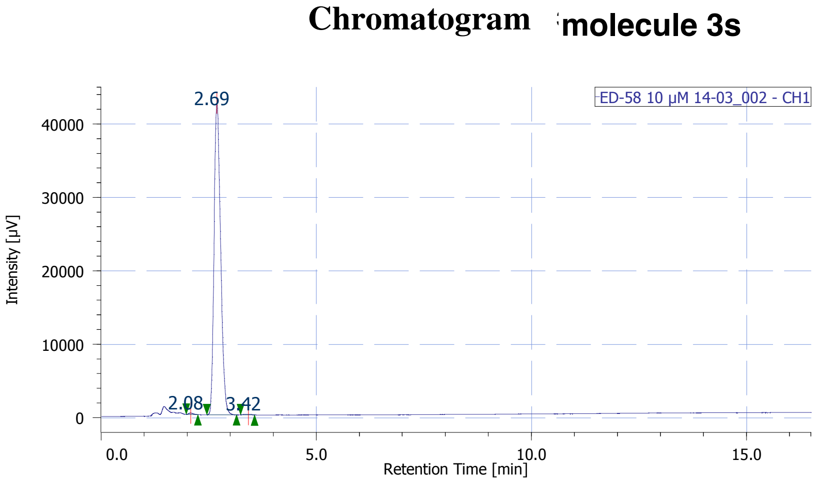

Chromatogram Information	
User Name	COM
Date Modified	3/14/2023 10:34:05 AM
Description	
HPLC System Name	HPLC binaire
Injection Date	3/14/2023 10:17:34 AM
Volume	20.00 [µL]
Sample Number	-
Project Name	HPLC binaire
Acquisition Time	29.9 [min]
Acquisition Sequence	
Control Method	elodie30-70
Peak ID Table	
Calibration Method	
Additional Information	

Channel & Peak Information Table	
Chromatogram Name	ED-58 10 µM 14-03_002-CH1
Sample Name	
Channel Name	UV
Sampling Interval	200 [msec]
Peak Method	(Manual)
Formula	
Decision	

#	Peak Name	CH	tR [min]	Area [µV·sec]	Height [µV]	Area%	Height%	Quantity	NTP	Resolution	Symmetry Factor	Warning
1	Unknown	1	2.08	1426	189	0.317	0.441	N/A	1586	2.493	1.278	
2	Unknown	1	2.69	447934	42452	99.462	99.365	N/A	1490	2.268	1.257	
3	Unknown	1	3.42	998	83	0.222	0.193	N/A	1373	N/A	0.896	

1 / 1

Figure S62 : HPLC chromatogram of 3t

Chromatogram molecule 3t

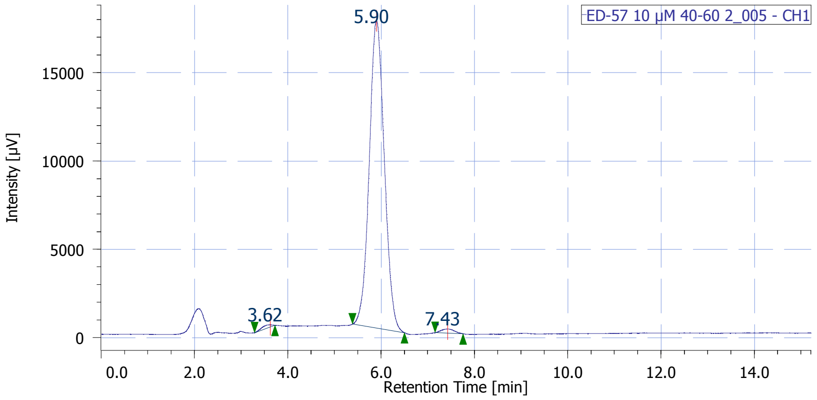

Chromatogram Information	
User Name	COM
Date Modified	3/16/2023 7:29:10 PM
Description	
HPLC System Name	HPLC binaire
Injection Date	3/16/2023 5:53:55 PM
Volume	20.00 [µL]
Sample Number	-
Project Name	HPLC binaire
Acquisition Time	29.9 [min]
Acquisition Sequence	
Control Method	elodie40-60+TFA
Peak ID Table	
Calibration Method	
Additional Information	

Channel & Peak Information Table	
Chromatogram Name	ED-57 10 µM 40-60 2_005-CH1
Sample Name	
Channel Name	UV
Sampling Interval	200 [msec]
Peak Method	(Manual)
Formula	
Decision	

#	Peak Name	CH	tR [min]	Area [µV·sec]	Height [µV]	Area%	Height%	Quantity	NTP	Resolution	Symmetry Factor	Warning
1	Unknown	1	3.62	2628	127	0.661	0.717	N/A	817	4.175	0.665	
2	Unknown	1	5.90	390715	17394	98.194	97.928	N/A	1608	2.712	1.049	
3	Unknown	1	7.43	4558	241	1.146	1.355	N/A	3024	N/A	1.097	

1 / 1

Figure S63 : HPLC chromatogram of 3u

Chromatogram molecule 3u

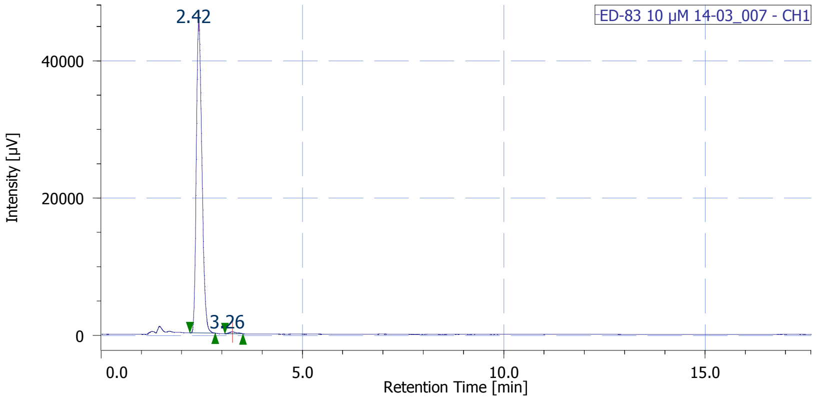

Chromatogram Information

User Name	COM
Date Modified	3/14/2023 12:02:41 PM
Description	
HPLC System Name	HPLC binaire
Injection Date	3/14/2023 11:45:02 AM
Volume	20.00 [µL]
Sample Number	-
Project Name	HPLC binaire
Acquisition Time	29.9 [min]
Acquisition Sequence	
Control Method	elodie30-70
Peak ID Table	
Calibration Method	
Additional Information	

Channel & Peak Information Table

Chromatogram Name	ED-83 10 µM 14-03_007-CH1
Sample Name	
Channel Name	UV
Sampling Interval	200 [msec]
Peak Method	(Manual)
Formula	
Decision	

#	Peak Name	CH	tR [min]	Area [µV·sec]	Height [µV]	Area%	Height%	Quantity	NTP	Resolution	Symmetry Factor	Warning
1	Unknown	1	2.42	440537	45464	99.087	99.367	N/A	1451	2.488	1.274	
2	Unknown	1	3.26	4060	290	0.913	0.633	N/A	965	N/A	1.257	

1 / 1

Figure S64 : HPLC chromatogram of 3v

Chromatogram molecule 3v

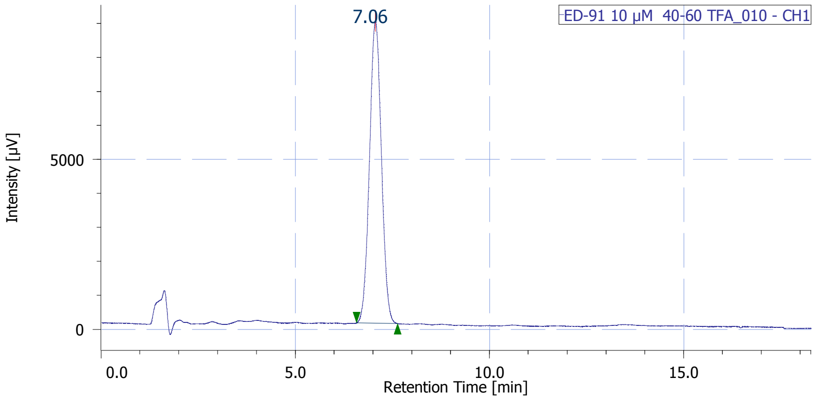

Chromatogram Information	
User Name	COM
Date Modified	3/15/2023 6:20:17 PM
Description	
HPLC System Name	HPLC binaire
Injection Date	3/15/2023 3:17:09 PM
Volume	20.00 [µL]
Sample Number	-
Project Name	HPLC binaire
Acquisition Time	29.9 [min]
Acquisition Sequence	
Control Method	elodie40-60+TFA
Peak ID Table	
Calibration Method	
Additional Information	

Channel & Peak Information Table	
Chromatogram Name	ED-91 10 µM 40-60 TFA_010-CH1
Sample Name	
Channel Name	UV
Sampling Interval	200 [msec]
Peak Method	(Manual)
Formula	
Decision	

#	Peak Name	CH	tR [min]	Area [µV·sec]	Height [µV]	Area%	Height%	Quantity	NTP	Resolution	Symmetry Factor
1	Unknown	1	7.06	189392	8897	100.000	100.000	N/A	2526	N/A	1.029

#	Warning
1	

1 / 1

Figure S65 : HPLC chromatogram of 3w

Chromatogram molecule 3w

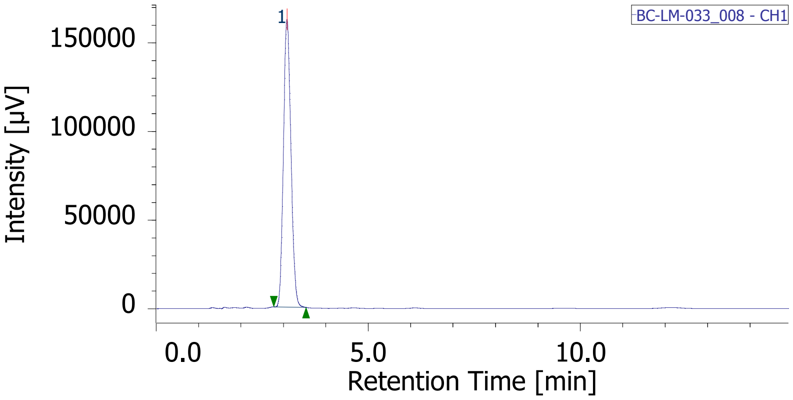

Chromatogram Information

User Name	COM
Date Modified	2/8/2024 12:19:36 PM
Description	
HPLC System Name	HPLC binaire
Injection Date	2/8/2024 12:04:41 PM
Volume	20.00 [µL]
Sample Number	-
Project Name	HPLC binaire
Acquisition Time	14.9 [min]
Acquisition Sequence	
Control Method	elodie+vincent-70%MeCN-TFA-254nm
Peak ID Table	
Calibration Method	
Additional Information	

Channel & Peak Information Table

Chromatogram Name	BC-LM-033_008-CH1
Sample Name	
Channel Name	UV
Sampling Interval	200 [msec]
Peak Method	(Manual)
Formula	
Decision	

#	Peak Name	CH	tR [min]	Area [µV·sec]	Height [µV]	Area%	Height%	Quantity	NTP	Resolution	Symmetry Factor
1	Unknown	1	3.08	1989107	162079	100.000	100.000	N/A	1444	N/A	1.125

#	Warning
1	

1 / 1

Figure S66 : HPLC chromatogram of 9

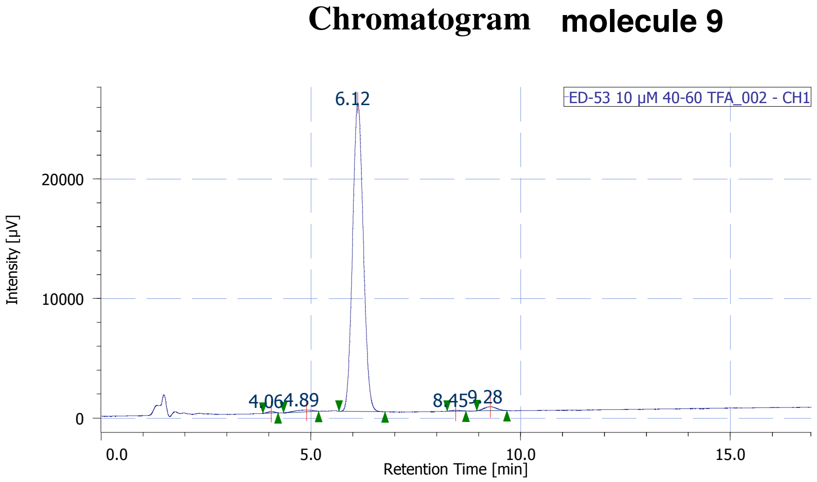

Chromatogram Information

User Name	COM
Date Modified	3/15/2023 6:20:04 PM
Description	
HPLC System Name	HPLC binaire
Injection Date	3/15/2023 10:53:15 AM
Volume	20.00 [µL]
Sample Number	-
Project Name	HPLC binaire
Acquisition Time	29.9 [min]
Acquisition Sequence	
Control Method	elodie40-60+TFA
Peak ID Table	
Calibration Method	
Additional Information	

Channel & Peak Information Table

Chromatogram Name	ED-53 10 µM 40-60 TFA_002-CH1
Sample Name	
Channel Name	UV
Sampling Interval	200 [msec]
Peak Method	(Manual)
Formula	
Decision	

#	Peak Name	CH	tR [min]	Area [µV·sec]	Height [µV]	Area%	Height%	Quantity	NTP	Resolution	Symmetry Factor	Warning
1	Unknown	1	4.06	1511	128	0.321	0.483	N/A	2193	1.362	0.911	
2	Unknown	1	4.89	4668	150	0.990	0.566	N/A	489	1.809	0.771	
3	Unknown	1	6.12	457539	25816	97.037	97.465	N/A	2698	5.012	1.075	
4	Unknown	1	8.45	1074	69	0.228	0.262	N/A	5338	1.609	1.072	
5	Unknown	1	9.28	6715	324	1.424	1.224	N/A	4270	N/A	1.058	

1 / 1

Figure S67 : HPLC chromatogram of 10

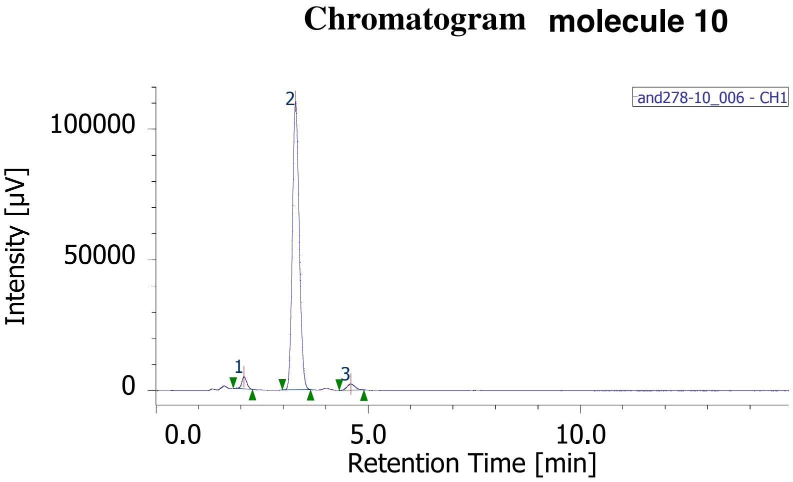

Chromatogram Information	
User Name	COM
Date Modified	3/7/2024 6:58:29 PM
Description	
HPLC System Name	HPLC binaire
Injection Date	3/7/2024 6:43:34 PM
Volume	20.00 [µL]
Sample Number	-
Project Name	HPLC binaire
Acquisition Time	14.9 [min]
Acquisition Sequence	
Control Method	elodie+vincent-70%MeCN-TFA-300nm
Peak ID Table	
Calibration Method	
Additional Information	

Channel & Peak Information Table	
Chromatogram Name	and278-10_006-CH1
Sample Name	
Channel Name	UV
Sampling Interval	200 [msec]
Peak Method	(Manual)
Formula	
Decision	

#	Peak Name	CH	tR [min]	Area [µV·sec]	Height [µV]	Area%	Height%	Quantity	NTP	Resolution	Symmetry Factor	Warning
1	Unknown	1	2.07	38408	4670	2.964	3.990	N/A	1522	4.809	1.002	
2	Unknown	1	3.29	1227391	110056	94.706	94.030	N/A	1984	4.079	1.130	
3	Unknown	1	4.59	30199	2317	2.330	1.980	N/A	2822	N/A	1.146	

1 / 1

Figure S68 : HPLC chromatogram of 13

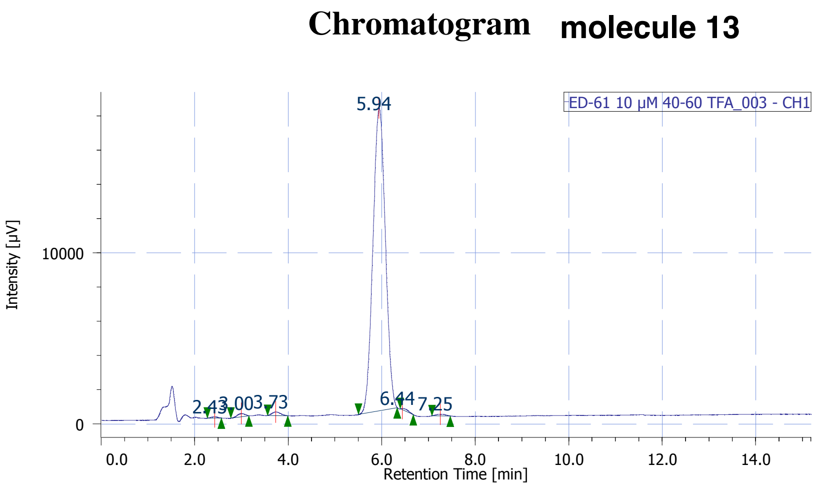

Chromatogram Information	
User Name	COM
Date Modified	3/15/2023 6:20:04 PM
Description	
HPLC System Name	HPLC binaire
Injection Date	3/15/2023 11:13:39 AM
Volume	20.00 [µL]
Sample Number	-
Project Name	HPLC binaire
Acquisition Time	29.9 [min]
Acquisition Sequence	
Control Method	elodie40-60+TFA
Peak ID Table	
Calibration Method	
Additional Information	

Channel & Peak Information Table	
Chromatogram Name	ED-61 10 µM 40-60 TFA_003-CH1
Sample Name	
Channel Name	UV
Sampling Interval	200 [msec]
Peak Method	(Manual)
Formula	
Decision	

#	Peak Name	CH	tR [min]	Area [µV·sec]	Height [µV]	Area%	Height%	Quantity	NTP	Resolution	Symmetry Factor	Warning
1	Unknown	1	2.43	710	76	0.212	0.416	N/A	1309	1.903	0.917	
2	Unknown	1	3.00	2232	188	0.667	1.026	N/A	1307	2.135	0.881	
3	Unknown	1	3.73	2717	209	0.811	1.137	N/A	1751	5.194	1.238	
4	Unknown	1	5.94	326763	17705	97.595	96.523	N/A	2294	1.192	1.023	
5	Unknown	1	6.44	1119	75	0.334	0.407	N/A	5768	2.160	3.119	
6	Unknown	1	7.25	1274	90	0.381	0.491	N/A	4868	N/A	1.119	

1 / 1

Figure S69 : HPLC chromatogram of 14

Chromatogram molecule 14

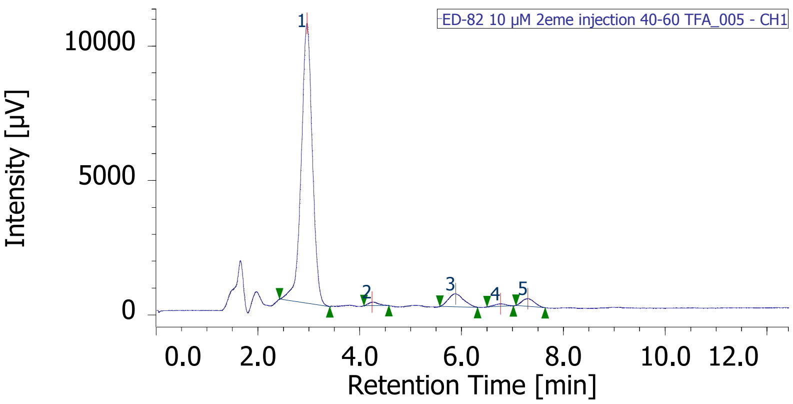

Chromatogram Information

User Name	COM
Date Modified	3/15/2023 6:20:06 PM
Description	
HPLC System Name	HPLC binaire
Injection Date	3/15/2023 12:03:24 PM
Volume	20.00 [µL]
Sample Number	-
Project Name	HPLC binaire
Acquisition Time	29.9 [min]
Acquisition Sequence	
Control Method	elodie40-60+TFA
Peak ID Table	
Calibration Method	
Additional Information	

Channel & Peak Information Table

Chromatogram Name	ED-82 10 µM 2eme injection 40-60 TFA_005-CH1
Sample Name	
Channel Name	UV
Sampling Interval	200 [msec]
Peak Method	(Manual)
Formula	
Decision	

#	Peak Name	CH	tR [min]	Area [µV·sec]	Height [µV]	Area%	Height%	Quantity	NTP	Resolution	Symmetry Factor	Warning
1	Unknown	1	2.96	158383	10400	89.606	91.166	N/A	943	3.386	0.959	
2	Unknown	1	4.24	2009	143	1.137	1.257	N/A	2073	3.492	1.549	
3	Unknown	1	5.88	10147	485	5.741	4.254	N/A	1707	1.763	1.161	
4	Unknown	1	6.76	1479	96	0.837	0.838	N/A	3860	1.182	0.935	
5	Unknown	1	7.30	4736	284	2.680	2.485	N/A	3779	N/A	1.137	

1 / 1

Figure S70 : HPLC chromatogram of 18

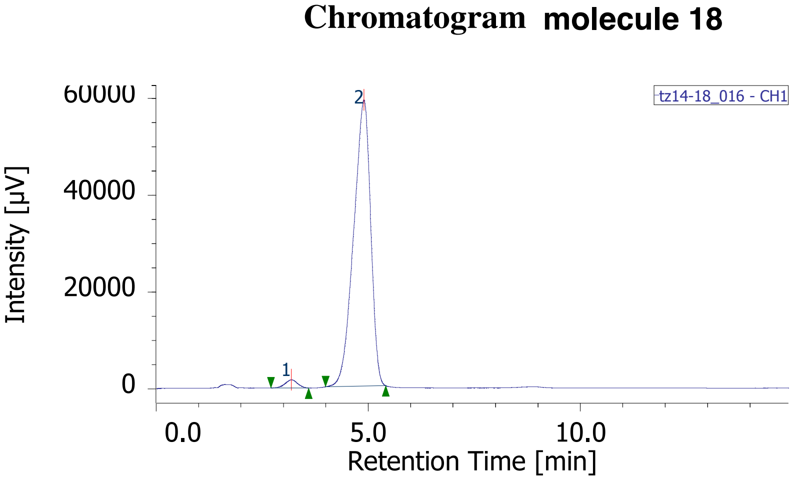

Chromatogram Information	
User Name	COM
Date Modified	3/8/2024 1:57:17 PM
Description	
HPLC System Name	HPLC binaire
Injection Date	3/8/2024 1:42:22 PM
Volume	20.00 [µL]
Sample Number	-
Project Name	HPLC binaire
Acquisition Time	14.9 [min]
Acquisition Sequence	
Control Method	candice-35%MeCN-TFA-300nm
Peak ID Table	
Calibration Method	
Additional Information	

Channel & Peak Information Table	
Chromatogram Name	tz14-18_016-CH1
Sample Name	
Channel Name	UV
Sampling Interval	200 [msec]
Peak Method	(Manual)
Formula	
Decision	

#	Peak Name	CH	tR [min]	Area [µV·sec]	Height [µV]	Area%	Height%	Quantity	NTP	Resolution	Symmetry Factor	Warning
1	Unknown	1	3.19	34142	1687	2.033	2.776	N/A	565	2.676	0.975	
2	Unknown	1	4.90	1645505	59060	97.967	97.224	N/A	691	N/A	0.827	

1 / 1

Figure S71 : HPLC chromatogram of 19

Chromatogram **molecule 19**

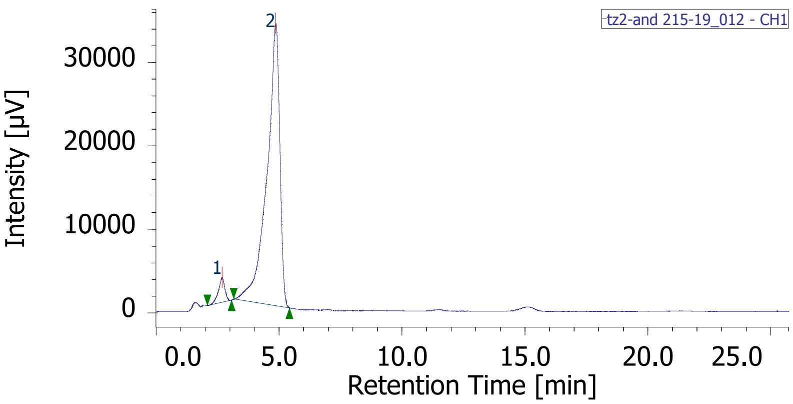

Chromatogram Information

User Name	COM
Date Modified	3/8/2024 1:05:49 PM
Description	
HPLC System Name	HPLC binaire
Injection Date	3/8/2024 12:40:05 PM
Volume	20.00 [µL]
Sample Number	-
Project Name	HPLC binaire
Acquisition Time	14.9 [min] + 20.0 [min]
Acquisition Sequence	
Control Method	candice-35%MeCN-TFA-300nm
Peak ID Table	
Calibration Method	
Additional Information	

Channel & Peak Information Table

Chromatogram Name	tz2-and 215-19_012-CH1
Sample Name	
Channel Name	UV
Sampling Interval	200 [msec]
Peak Method	(Manual)
Formula	
Decision	

#	Peak Name	CH	tR [min]	Area [µV·sec]	Height [µV]	Area%	Height%	Quantity	NTP	Resolution	Symmetry Factor	Warning
1	Unknown	1	2.68	53572	2951	4.183	8.034	N/A	553	3.357	0.823	
2	Unknown	1	4.86	1227119	33785	95.817	91.966	N/A	526	N/A	0.666	

Figure S72 : HPLC chromatogram of 21

Chromatogram **molecule 21**

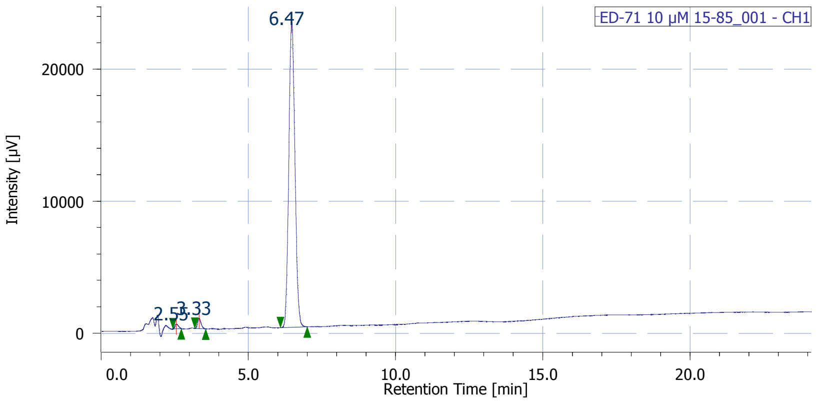

Chromatogram Information
User Name COM
Date Modified 3/16/2023 7:29:09 PM
Description
HPLC System Name HPLC binaire
Injection Date 3/16/2023 2:47:27 PM
Volume 20.00 [µL]
Sample Number -
Project Name HPLC binaire
Acquisition Time 29.9 [min]
Acquisition Sequence
Control Method elodie-15-85+TFA
Peak ID Table
Calibration Method
Additional Information

Channel & Peak Information Table
Chromatogram Name ED-71 10 µM 15-85_001-CH1
Sample Name
Channel Name UV
Sampling Interval 200 [msec]
Peak Method (Manual)
Formula
Decision

#	Peak Name	CH	tR [min]	Area [µV·sec]	Height [µV]	Area%	Height%	Quantity	NTP	Resolution	Symmetry Factor	Warning
1	Unknown	1	2.55	3042	358	0.886	1.478	N/A	1867	3.406	1.313	
2	Unknown	1	3.33	6492	781	1.891	3.227	N/A	3619	10.435	1.199	
3	Unknown	1	6.47	333746	23072	97.223	95.295	N/A	4558	N/A	1.084	

1 / 1

Figure S73 : HPLC chromatogram of 22

Chromatogram molecule 22

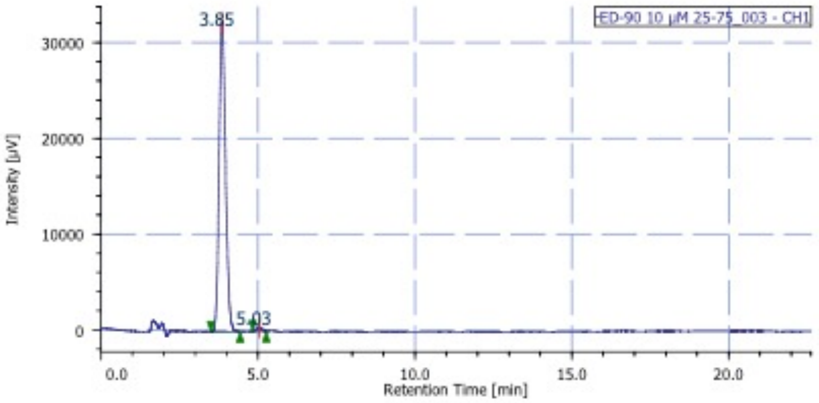

Chromatogram Information

User Name	COM
Date Modified	3/16/2023 7:29:09 PM
Description	
HPLC System Name	HPLC binaire
Injection Date	3/16/2023 4:25:32 PM
Volume	20.00 [µL]
Sample Number	-
Project Name	HPLC binaire
Acquisition Time	29.9 [min]
Acquisition Sequence	
Control Method	elodie-25-75+TFA
Peak ID Table	
Calibration Method	
Additional Information	

Channel & Peak Information Table

Chromatogram Name	ED-90 10 µM 25-75_003-CH1
Sample Name	
Channel Name	UV
Sampling Interval	200 [msec]
Peak Method	(Manual)
Formula	
Decision	

#	Peak Name	CH	tR [min]	Area [µV·sec]	Height [µV]	Area%	Height%	Quantity	NTP	Resolution	Symmetry Factor	Warning
1	Unknown	1	3.85	463192	32278	98.894	98.614	N/A	1688	3.462	1.167	
2	Unknown	1	5.03	5180	454	1.106	1.386	N/A	4313	N/A	1.098	

1 / 1

Figure S74 : HPLC chromatogram of 23

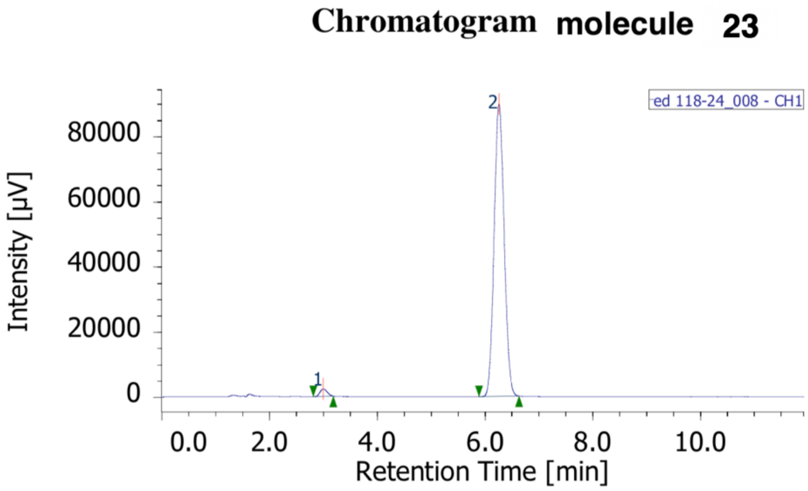

Chromatogram Information	
User Name	COM
Date Modified	3/7/2024 7:35:52 PM
Description	
HPLC System Name	HPLC binaire
Injection Date	3/7/2024 7:23:56 PM
Volume	20.00 [µL]
Sample Number	-
Project Name	HPLC binaire
Acquisition Time	14.9 [min]
Acquisition Sequence	
Control Method	elodie+vincent-70%MeCN-TFA-300nm
Peak ID Table	
Calibration Method	
Additional Information	

Channel & Peak Information Table	
Chromatogram Name	ed 118-24_008-CH1
Sample Name	
Channel Name	UV
Sampling Interval	200 [msec]
Peak Method	(Manual)
Formula	
Decision	

#	Peak Name	CH	tR [min]	Area [µV·sec]	Height [µV]	Area%	Height%	Quantity	NTP	Resolution	Symmetry Factor	Warning
1	Unknown	1	2.99	21190	2295	1.791	2.498	N/A	2255	10.978	1.144	
2	Unknown	1	6.26	1162159	89602	98.209	97.502	N/A	5295	N/A	1.068	

3. References

[1] S. Steinhauser, U. Heinz, M. Bartholomä, T. Weyhermüller, H. Nick, K. Hegetschweiler, *Eur. J. Inorg. Chem.* **2004**, *21*, 4177-4192.

[2] F. Slowinski, O. Ben Ayad, O. Ziyaret, C. Botuha, L. Le Falher, K. Aouane, S. Thorimbert, *Org. Lett*. **2013**, *15*, 3494-3497.

[3] A. Nina-Diogo, B. Bertrand, S. Thorimbert, G. Gontard, S. Nassem-Kahn, A. Echeverri, J. Contreras-Garcia, C. Allain, G. Lemercier, E. Luppi, C. Botuha, *Adv. Opt. Mat*. **2023**, *11*, 2300336.

[4] S. Stucky, N. J. Koch, U. Heinz, K. Hegetschweiler, *Org. Pap*. **2008**, *62*, 388-397.

[5] U. Heinz, K. Hegetschweiler, P. Acklin, B. Faller, R. Lattmann, H. P. Schnebli, *Angew. Chem. Int. Ed. Engl*. **1999**, 38, 2568-2570.

[6] Y. Hu, S. Jiao, Y. Wang, R. Chen, G. Li, Z. Zou, *ChemistrySelect,* **2021**, *6*, 12914-12920.

[7] L. C. C. Coetzee, A. J. Muller, A. S. Adeyinka, M. S. Sonopo, D. B. G. Williams, *Results Chem*. **2021**, *3*, 100165.

[8] V. J. Rao, K. Mukkanti, N. A. Vekariya, P. B. Gupta, A. Islam, *Synth. Commun*. **2012**, *42*, 3200-3210.

[9] S. Salehi, A. Saljooghi, A. Shiri, *Eur. J. Pharmacol.* **2016**, *78*1, 209-217.

[10] X. L. Hu, A. C. Sedgwick, D. N. Mangel, Y. Shang, A. Steinbrueck, K. C. Yan, L. Zhu, D. W. Snelson, S. Sen, C. V. Chau, G. Juarez, V. M. Lynch, X. P. He, J. L. Sessler, *J. Am. Chem. Soc.* **2022**, *144*, 7382-7390.

[11] J. Shi, Y. Wang, J. Chen, Y. Lao, P. Huang, L. Liao, C. Jiang, X. Li, J. Wen, S. Zhou, J. Zhang, *Neurochem. Int.* **2021**, *148*, 105103.

[12] A. Steinbrueck, A. C. Sedgwick, H. H. Han, M. Y. Zhao, S. Sen, D. Y. Huang, Y. Zang, J. Li, X. P. He, J. L. Sessler, *Chem. Commun*. **2021**, *57*, 5678-5681.

[13] L. Le Falher, O. Ben Ayad, O. Ziyaret, C. Botuha, S. Thorimbert, F. Slowinski, *Eur. J. Org. Chem*. **2015**, *17*, 3830-3840.

[14] B. Bertrand, C. Botuha, J. Forte, H. Dossmann, M. Salmain, *Chem. Eur. J.* **2020**, *26*, 12846-12861.
